# Forecasting the Potential Effects of Climate Change on Malaria in the Lake Victoria Basin Using Regionalized Climate Projections

**DOI:** 10.1007/s11686-022-00588-4

**Published:** 2022-08-12

**Authors:** Ednah N. Ototo, Joseph O. Ogutu, Andrew Githeko, Mohammed Y. Said, Lucy Kamau, Didacus Namanya, Stella Simiyu, Stephen Mutimba

**Affiliations:** 1 Kenyatta University, P.O. Box 43844, Nairobi, Kenya; 2 Kenya Medical Research Institute, Centre for Global Health Research, Climate and Human Health Research Unit, P.O. Box 1578, Kisumu, Kenya; 3 Institute for Crop Science-340, University of Hohenheim, 70599 Stuttgart, Germany; 4 C&E Advisory Kenya, P.O. Box 76406-00508, Nairobi, Kenya; 5 Uganda Ministry of Health, P.O. Box 7272, Kampala, Uganda; 6 Institute of Climate Change and Adaptation, University of Nairobi, P.O. Box 30197, Nairobi, 00100 Kenya

**Keywords:** Climate change, Malaria, Regionalized climate projections

## Abstract

**Background:**

Malaria epidemics are increasing in East Africa since the 1980s, coincident with rising temperature and widening climate variability. A projected 1–3.5 °C rise in average global temperatures by 2100 could exacerbate the epidemics by modifying disease transmission thresholds. Future malaria scenarios for the Lake Victoria Basin (LVB) are quantified for projected climate scenarios spanning 2006–2100.

**Methods:**

Regression relationships are established between historical (1995–2010) clinical malaria and anaemia cases and rainfall and temperature for four East African malaria hotspots. The vector autoregressive moving average processes model, VARMAX (*p,q,s*), is then used to forecast malaria and anaemia responses to rainfall and temperatures projected with an ensemble of eight General Circulation Models (GCMs) for climate change scenarios defined by three Representative Concentration Pathways (RCPs 2.6, 4.5 and 8.5).

**Results:**

Maximum temperatures in the long rainy (March–May) and dry (June–September) seasons will likely increase by over 2.0 °C by 2070, relative to 1971–2000, under RCPs 4.5 and 8.5. Minimum temperatures (June–September) will likely increase by over 1.5–3.0 °C under RCPs 2.6, 4.5 and 8.5. The short rains (OND) will likely increase more than the long rains (MAM) by the 2050s and 2070s under RCPs 4.5 and 8.5. Historical malaria cases are positively and linearly related to the 3–6-month running means of monthly rainfall and maximum temperature. Marked variation characterizes the patterns projected for each of the three scenarios across the eight General Circulation Models, reaffirming the importance of using an ensemble of models for projections.

**Conclusions:**

The short rains (OND), wet season (MAM) temperatures and clinical malaria cases will likely increase in the Lake Victoria Basin. Climate change adaptation and mitigation strategies, including malaria control interventions could reduce the projected epidemics and cases. Interventions should reduce emerging risks, human vulnerability and environmental suitability for malaria transmission.

**Supplementary Information:**

The online version contains supplementary material available at 10.1007/s11686-022-00588-4.

## Introduction

Malaria is a major health concern in large parts of the world. Roughly half of the world's population is at risk of malaria [1]. Within Africa, one in every five (20%) childhood deaths are due to the effects of the disease. In East Africa, the probability of malaria deaths is high, but all the East African Community (EAC) Partner States are still at the level of control, and achieving elimination will likely be impeded by widening climatic variability and change. Due to scale up of malaria prevention measures, malaria prevalence has declined significantly in the EAC region since the 2000s [1]. However, since the 1980s, malaria epidemics have been increasing in the East African highlands. This trend has been attributed to climatic variability and change, antimalarial drug resistance, land use change, vector migration [2, 3] and epidemiology of the disease.

The epidemiology of the vector borne disease is largely influenced by climate variability and change as evidenced by recent outbreaks and disease patterns [4]. Notably, both malaria parasites and vectors are highly sensitive to changes in rainfall, humidity, air and water temperatures [5]. Rising temperatures shorten the sporogonic cycle of the parasites and accelerate the vector growth and development [6]. Malaria transmission threshold is 18 °C [7] but 20.8 °C is the optimal global temperature at which malaria mortality increases for all ages [8]. It follows that the projected increase in annual global temperatures by up to 3.5 °C by 2100 will likely elevate both malaria transmission and mortality [9, 10].

Temperature is an important predictor of malaria transmission in different regions [6, 11]. Consequently, future scenarios of malaria risk under changing climatic conditions have been explored using multiple modelling approaches [12]. Results of these models suggest that due to precipitation and temperature changes, the geographical distribution of malaria in Africa will likely change by 2100 [13]. The anticipated changes differ markedly geographically. Thus, by 2050 and 2080, for example, some regions will become more suitable, whereas others, such as southern central Africa, may become unsuitable for malaria transmission [14].

Similarly, because of climate change, changes can be expected in the distribution, range, prevalence, incidence and seasonality of vector borne and waterborne diseases in East Africa. However, the nature and magnitude of the anticipated future changes in the Lake Victoria Basin have not yet been quantified. Lake Victoria basin has a high poverty index and poor health infrastructure and hence its residents are exposed to a high risk of being affected by these diseases [15]. Climatic extremes, such as the El Niño-Southern Oscillation and Indian Dipole events, can be expected to increase the frequency and intensity of disease outbreaks based on historic patterns [16, 17]. The projected future climate change and variability under various scenarios and the anticipated effects of these changes on the pattern and intensity of disease outbreaks should thus form the basis for developing effective and far-sighted control methods and policies.

We examined future malaria scenarios for the Lake Victoria Basin in East Africa in response to climate variability based on projected climate scenarios for 2006–2100 defined by the Representative Concentration Pathways (RCPs) RCP2.6, RCP4.5, and RCP8.5.

## Materials and Methods

### Study Area

**Fig. 1:**
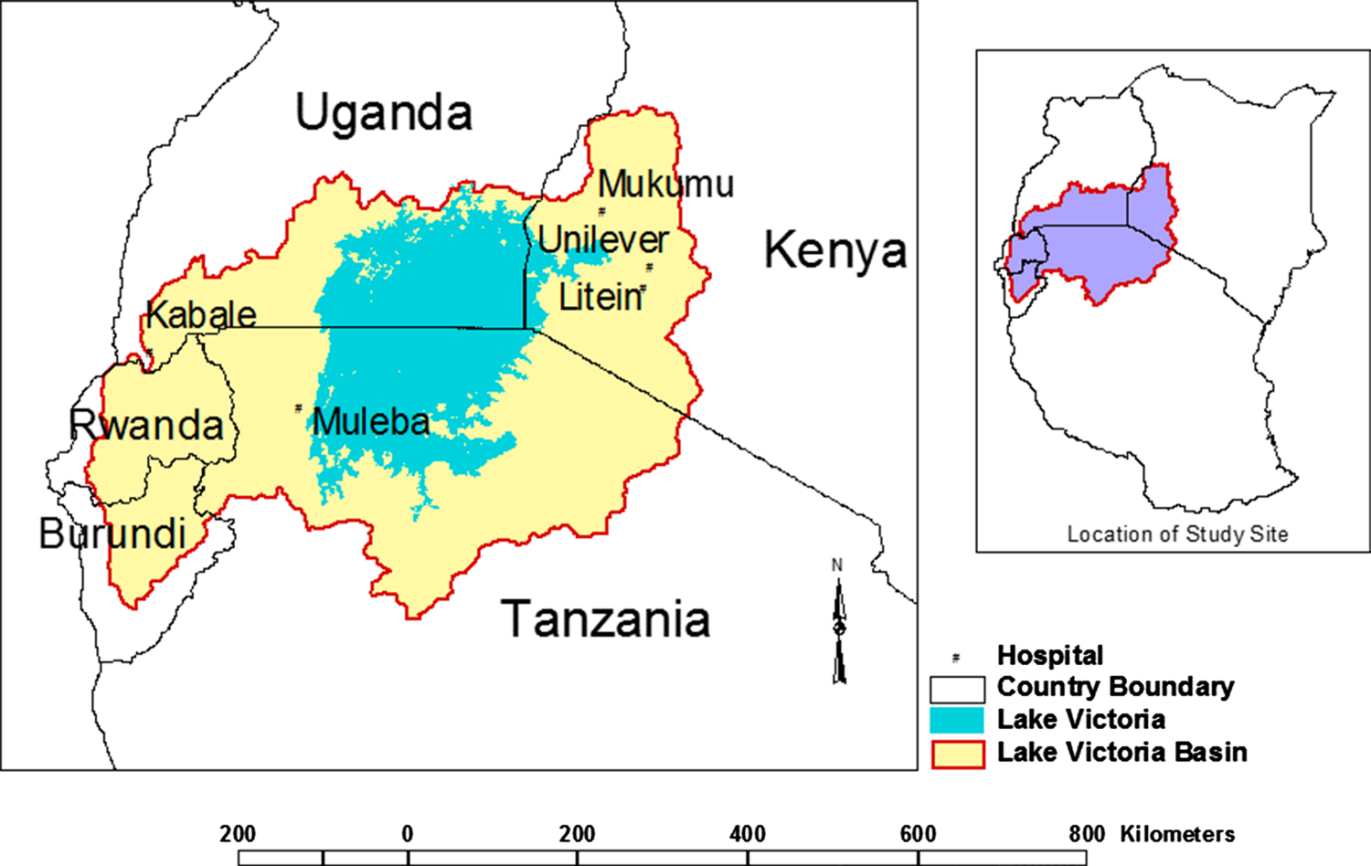
Map showing the location of the study sites in the Lake Victoria Basin

Malaria transmission hotspots in the Lake Victoria Basin were chosen based on the following characteristics: (1) highland regions at altitudes exceeding 1500 m; (2) rainfall during the wet season months of March to May exceeding 150 mm; (3) minimum temperature of 18 °C, the temperature threshold that determines malaria vector breeding sites; and (4) occurrence of infective vectors which can be measured by calculating entomological inoculation rate (EIR), and have clusters of clinical malaria episodes [7, 18]. Based on geospatial data on rainfall, temperature, altitude and slopes, a hot spot map was derived (Fig. 1). We selected four hotspot sites with long-term clinical malaria data, comprising Muleba (Tanzania), Kericho and Mukumu (Kenya) and all hospitals, including at Kabale, in Uganda. Anaemia was also considered for Muleba in Tanzania (Table 1).

**Table 1:** Characteristics of the selected study sites in the Lake Victoria Basin

Country	Study site	Characteristics
Tanzania	Muleba District	Unstable seasonal malaria occurs in the highland areas, with low malaria transmission of not more than three months a year [52, 53]
Kenya	Kericho	The area has been a hot spot for malaria epidemics since 1918, with early epidemics occurring in 1928, 1931, 1932, 1934, 1937, and 1940. The latest epidemic was recorded in 1997/1998 [2, 3, 54]
	Mukumu	Has flat shaped valleys that form suitable habitats for the malaria vectors, making these regions hot spots for malaria transmission [39, 55]
Uganda	Kabale	Malaria has been investigated in the area since 1930s and malaria epidemics have been noted since 1948; including in 1998, 2005 and 2006 [54, 56]. Most epidemics occurred in the highlands [2]

### Data and Analysis

#### Historical Rainfall and Temperature Data

We used the historical rainfall data based on the gridded observation/satellite blended Climate Hazards Group InfraRed Precipitation with Station data (CHIRPS). CHIRPS is a 30+ year quasi-global rainfall dataset that starts in 1981. The temperature data were downloaded from the Climate Hazards Group website (http://chg.geog.ucsb.edu/tools/geoclim/index.html) and covered the period 1995–2010. The GeoCLIM software tool was used to extract the rainfall and temperature data for each of the study sites. Details of the GeoCLIM tool can be found at http://chg-wiki.geog.ucsb.edu/wiki/GeoCLIM.

#### Projected Climate Data

Climate change analysis and projections were based on the regional downscaled climate model. The downscaling is performed using multiple regional climate models (RCMs) as well as statistical downscaling (SD) techniques. We used the simulated data from the Rossby Center Regional Atmospheric Model (RCA4) driven by the Earth system version of the Max Planck Institute for Meteorology (MPI-ESM-LR) coupled with the global climate model from the Coordinated Regional Downscaling Experiment (CORDEX) program [19]. This was supplemented with simulated data from seven additional General Circulation Models (GCMs).

The regional climate models (RCMs) work at finer spatial resolutions over limited geographic regions and are presumed to perform better at regional scales. The eight RCMs from the Coordinated Regional Climate Downscaling Experiment program (CORDEX) that we used in the analyses were sourced from the Swedish Meteorological and Hydrologic Institute (SMHI), Koninklijk Nederlands Meteorologisch Instituut (KNMI) and Max Plank Institute (MPI) Climate Service Centre (CSC) at ~ 50 km spatial resolution. The data were downscaled by the Rossby Centre regional climate model-RCA4 model and Climate Model Intercomparison Project (CMIP5). The GCM’s and RCMs we considered, their names and associated references are summarized in Table 2.

**Table 2:** The number and name of the ensemble of eight general circulation models (GCMs), the institutions that used the model to generate the climate projections and the reference for each model

Number	Model	GCM	RCM	Institutions	References
1	AFR44_MPI_M_MPI_ESM_LR_MPI_SMHI_REMO	MPI-ESM-LR (MPI-M)	REMO (SMHI)	Max Planck—Institute for Meteorology, Germany	[19]
2	AFR-44_ICHEC_EC_EARTH_SMHI-RCA4	EC-EARTH (ICHEC)	RCA4 (SMHI)	Environmental Protection Agency, Ireland	[57]
3	AFR-44_MIROC_MIROC5_SMHI-RCA4	MIROC5 (MIROC)	RCA4 (SMHI)	Japan Agency for Marine-Earth Science and Technology, Atmosphere and Ocean Research Institute (The University of Tokyo), Japan	[58]
4	AFR-44_MOHC_HadGEM2_ES_KNMI_RACMO2	HadGEM2-ES (MOHC)	RACM02 (KNMI)	Koninklijk Nederlands Meteorologisch Instituut (KNMI)	[59]
5	AFR-44_MOHC_HadGEM2_ES_SMHI_RCA4	HadGEM2-ES (MOHC)	RCA4 (SMHI)	Met Office Hadley Centre, United Kingdom	[60]
6	AFR44_MPI_M_MPI_ESM_LR_MPI_CSC_REMO2009	MPI-ESM-LR (MPI-M)	REM02009 (MPI-CSC)	Max Planck—Institute for Meteorology, Germany	[61]
7	AFR-44_MPI_M_MPI_ESM_LR_SMHI_RCA4	MPI-ESM-LR (MPI-M)	RCA4 (SMHI)	Max Planck—Institute for Meteorology, Germany	[61]
8	AFR-44_NCC_NorESM1_M_SMHI_RCA4	NorESMI-M (NCC)	RCA4 (SMHI)	Norwegian Climate Centre, Norway	[62]

Three climate change scenarios were used to project rainfall and temperatures. These correspond with three Representative Concentration Pathways (RCPs) RCP2.6, RCP4.5, and RCP8.5, with the numeric suffixes referencing radiative forcings (global energy imbalances), measured in watts/m^2^, by the year 2100. The three Representative Concentration Pathways give various possibilities of rainfall and temperature changes based on global initiatives to limit gaseous emissions. RCP 2.6 represents an optimistic projection characterized by a very low concentration and emission levels of greenhouse gases. RCP 4.5 scenario represents medium emission scenario in which the international communities are working on limiting emissions with limited implementation of climate change policies. RCP 8.5 scenario represents a pessimistic projection with high levels of concentrations of gases emitted and assumes no implementation of climate change policies [20].

**Fig. 2:**
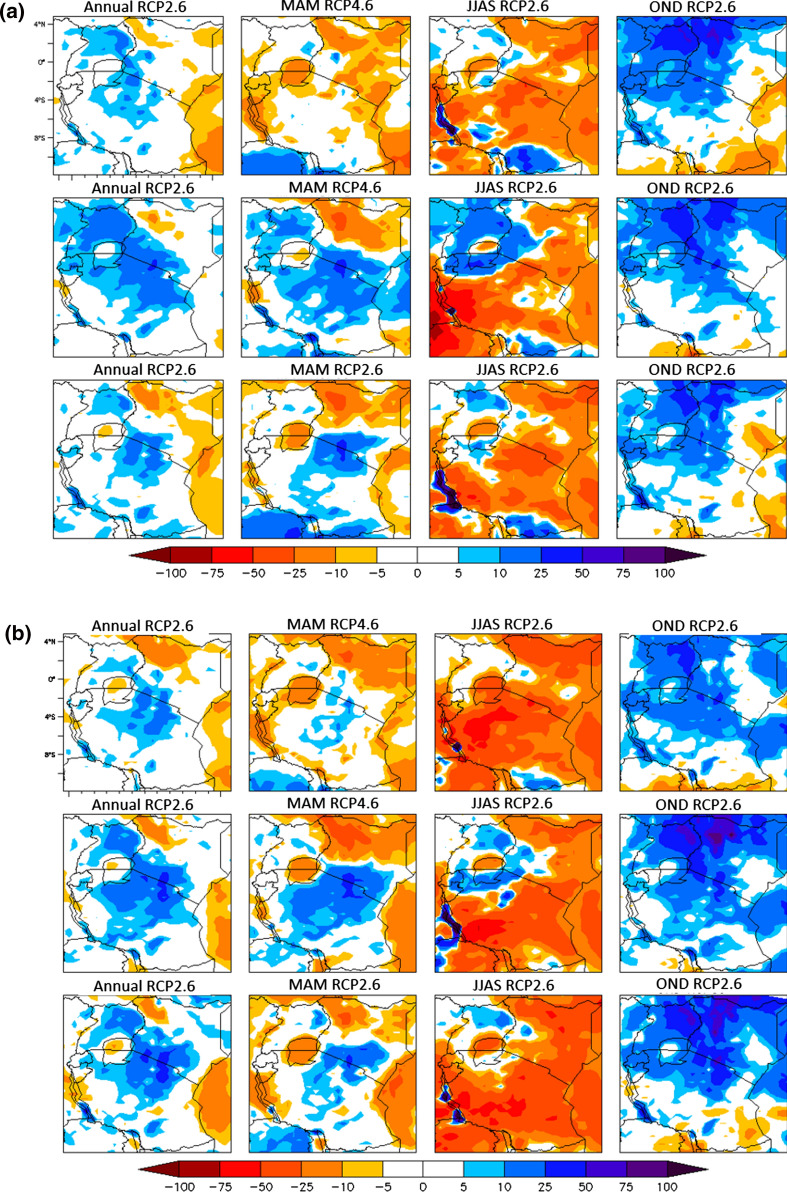
**a** Projected rainfall changes over EAC by 2030s in the annual (1st column), MAM (2nd column), JJAS (3rd column), and OND (4th column) components. Each row corresponds to emission scenarios: RCP2.6 (1st row), RCP4.5 (2nd row) and RCP8.5 (3rd row). **b** Projected rainfall changes over EAC by 2050s in the annual (1st column), MAM (2nd column), JJAS (3rd column), and OND (4th column) components. Each row corresponds to emission scenarios: RCP2.6 (1st row), RCP4.5 (2nd row) and RCP8.5 (3rd row)

**Fig. 3:**
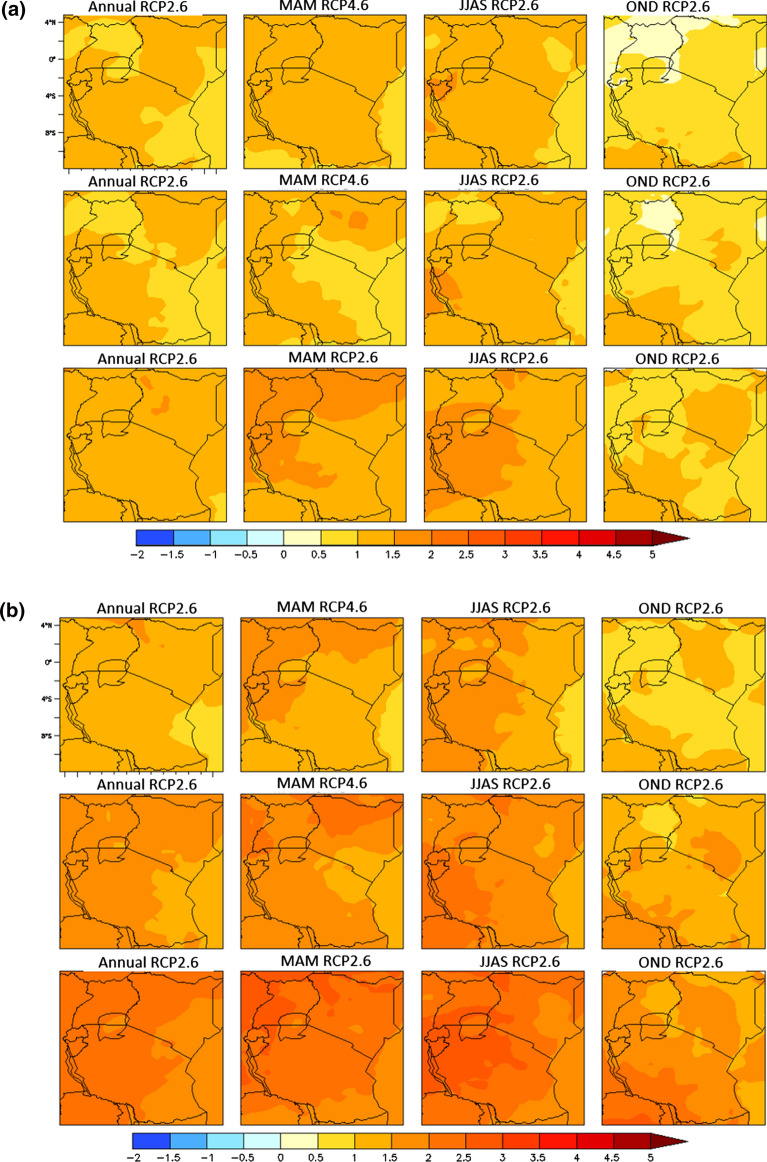

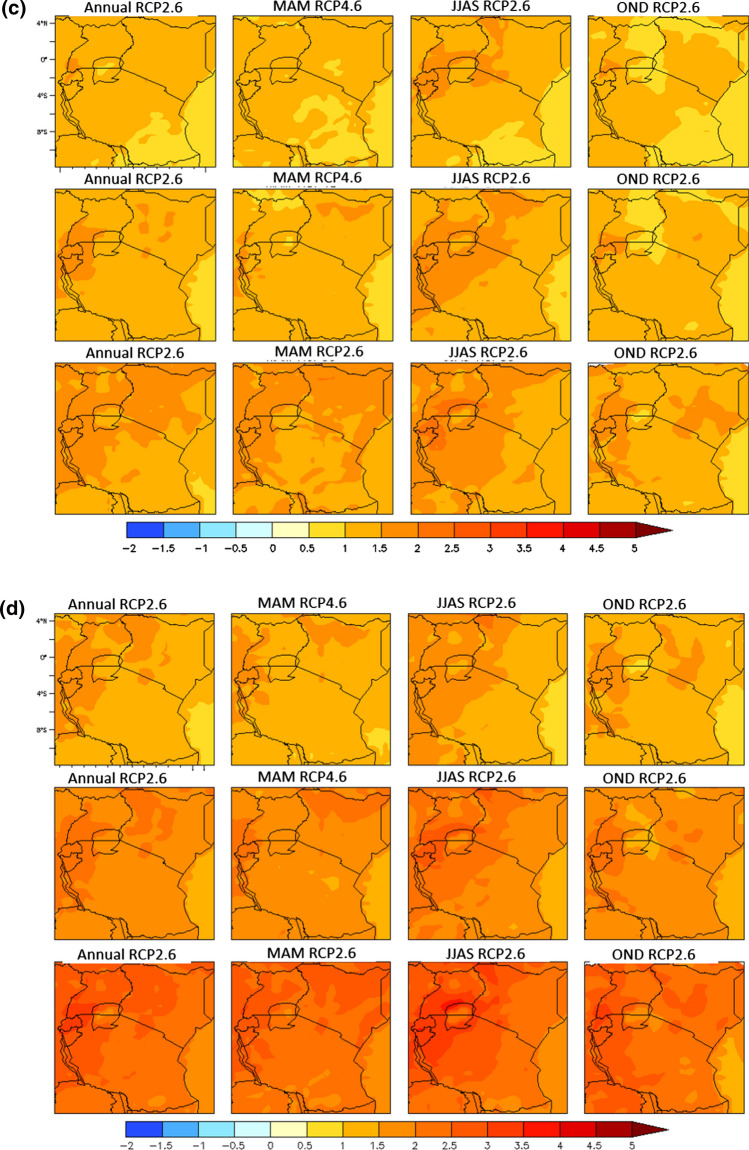
**a** Projected maximum temperature changes over EAC by 2030s in the annual (1st column), MAM (2nd column), JJAS (3rd column), and OND (4th column) components. Each row corresponds to emission scenarios: RCP2.6 (1st row), RCP4.5 (2nd row) and RCP8.5 (3rd row). **b** Projected maximum temperature changes over EAC by 2050s in the annual (1st column), MAM (2nd column), JJAS (3rd column), and OND (4th column) components. Each row corresponds to emission scenarios: RCP2.6 (1st row), RCP4.5 (2nd row) and RCP8.5 (3rd row). **c** Projected minimum temperature changes over EAC by 2030s in the annual (1st column), MAM (2nd column), JJAS (3rd column), and OND (4th column) components. Each row corresponds to emission scenarios: RCP2.6 (1st row), RCP4.5 (2nd row) and RCP8.5 (3rd row). **d** Projected minimum temperature changes over EAC by 2050s in the annual (1st column), MAM (2nd column), JJAS (3rd column), and OND (4th column) components. Each row corresponds to emission scenarios: RCP2.6 (1st row), RCP4.5 (2nd row) and RCP8.5 (3rd row)

The projected rainfall and temperature changes were analyzed for the three scenarios for the five countries bordering Lake Victoria for the 2030s (2016–2045), 2050s (2036–2065) and 2070s (2055–2085) to provide information on the expected magnitude of the climate changes over each time window [21]. The period 1971–2000 is considered as a reference for the present climate (Figs. 2, 3). The projected climate change signals for each time window are calculated as the difference between the projection for the future time window and the reference period. Rainfall in the LVB is divided into three distinct seasons, the main long rains (March–April–May (MAM), short rains (October–November–December (OND) and the long dry season (June–July–August–September (JJAS). We also consider the annual rainfall, the total cumulative rainfall from January to December.

### Statistical Analysis and Forecasting

Monthly malaria data from selected sites from three countries, Kenya, Uganda and Tanzania, and spanning the period 1995–2010 were used to develop regression relationships with historic rainfall and temperature data. The established regression relationships were then used with projected rainfall and temperature data from eight Global Circulation Models provided by the Intergovernmental Authority on Development (IGAD) Climate Prediction and Applications Centre (ICPAC) for the period 2006–2100 for each of the three scenarios. The relationships between malaria incidences and cases and historic rainfall or temperature were carefully statistically modelled. This involved relating the incidences or cases to various lagged values and cumulative moving averages of rainfall, minimum and maximum temperatures using the Pearson product moment correlation that assumes bivariate normality of the two variables being correlated and linearity of the relationship between them. The Spearman rank correlation, which is more appropriate if the relationship between two variables is nonlinear and monotonically increasing or decreasing was also computed. Lastly, the Akaike information criterion (AIC) and its corrected variant, the corrected Akaike information criterion (AICc) [22], were computed to rank contending models (linear and nonlinear regression models) in terms of the weight of evidence in support of each. The best AICc-supported models and rainfall or temperature components from the preceding step were then used to build final regression models. These were then used to estimate the associated intercept and slope coefficients and their standard errors and assess statistical significance of the parameter estimates.

The VARMARX (*p,q,s*) model used for forecasting can accommodate the following features. (1) Modelling of several time series simultaneously (vector). (2) Accounting for relationships among the individual component series with current and past values of the other series (X). (3) Feedback from the response series and cross-correlated explanatory series. (4) Cointegration of component series to achieve stationarity. (5) Autoregressive errors (of order *p*). (6) Moving average errors (of order *q*). (7) Distributed lags in the explanatory series (of order *s*). (8) Seasonality. (9) Mixed autoregressive and moving average errors. (10) Unequal or heteroscedastic covariances for the residuals using various generalized autoregressive conditional heteroscedasticity (GARCH) models [23]. (11) The modelling framework used also allows for testing the dependence of one response series on another (testing for weak exogeneity). (12) Moreover, under certain special regularity conditions, the model allows for testing for Granger causality between two specified groups of variables. For example, the Granger causality test may be used by defining one group consisting of the response series and the other of climatic predictor series. (13) Lastly, the modelling framework allows for putting various restrictions on the estimated parameter coefficients of the model or their linear combinations and testing hypotheses on linear combinations of the parameter coefficients.

The second model used for forecasting the response series is the unobserved components model (UCMs). This model was only used for those response series that were nonlinearly related to one of the explanatory series. The UCM is a structural time series model and a special case of the general state space model, appropriate for modelling and forecasting time series. The UCM model can accommodate nonlinear relationships between the response and the explanatory series. Nonlinearity was modelled using penalized cubic basis splines, or equivalently, random regression or varying coefficients regression models. In addition, the UCM used can accommodate autoregression, seasonality, multiple cyclical patterns, dynamic level shifts, dynamic trend and other features. The UCMs can thus also be viewed as dynamic regression models with multiple predictors. The UCM can provide smoothed trend and cyclical patterns. For more complex univariate or multivariate models that cannot be handled by the UCM, the general state space model, which is much more general and versatile than the UCM and can accommodate more general univariate models and multivariate time series, among other features, can be used for forecasting [23–26].

More detailed descriptions of the statistical modelling and forecasting methodology are provided in SI 1–9. The historic data and the future forecasts under the three emission scenarios for the ensemble of eight projection models are provided in SI 2–4 and in S1–S3 data. SI 10 provides the SAS program codes used to fit the forecasting models and produce plots of the forecast trajectories.

## Results

For brevity, we summarize here only the projected rainfall and temperature data simulated by the Rossby Center Regional Atmospheric Model driven by the Earth system version of the Max Planck Institute for Meteorology (Model 1 in Table 2) coupled with the global climate model from the Coordinated Regional Downscaling Experiment (CORDEX) program. Additionally, we present the projected trajectories of malaria and anaemia cases for the ensemble of eight models and the associated historic series.

### Projected Temperatures Change in LVB

**Fig. 4:**
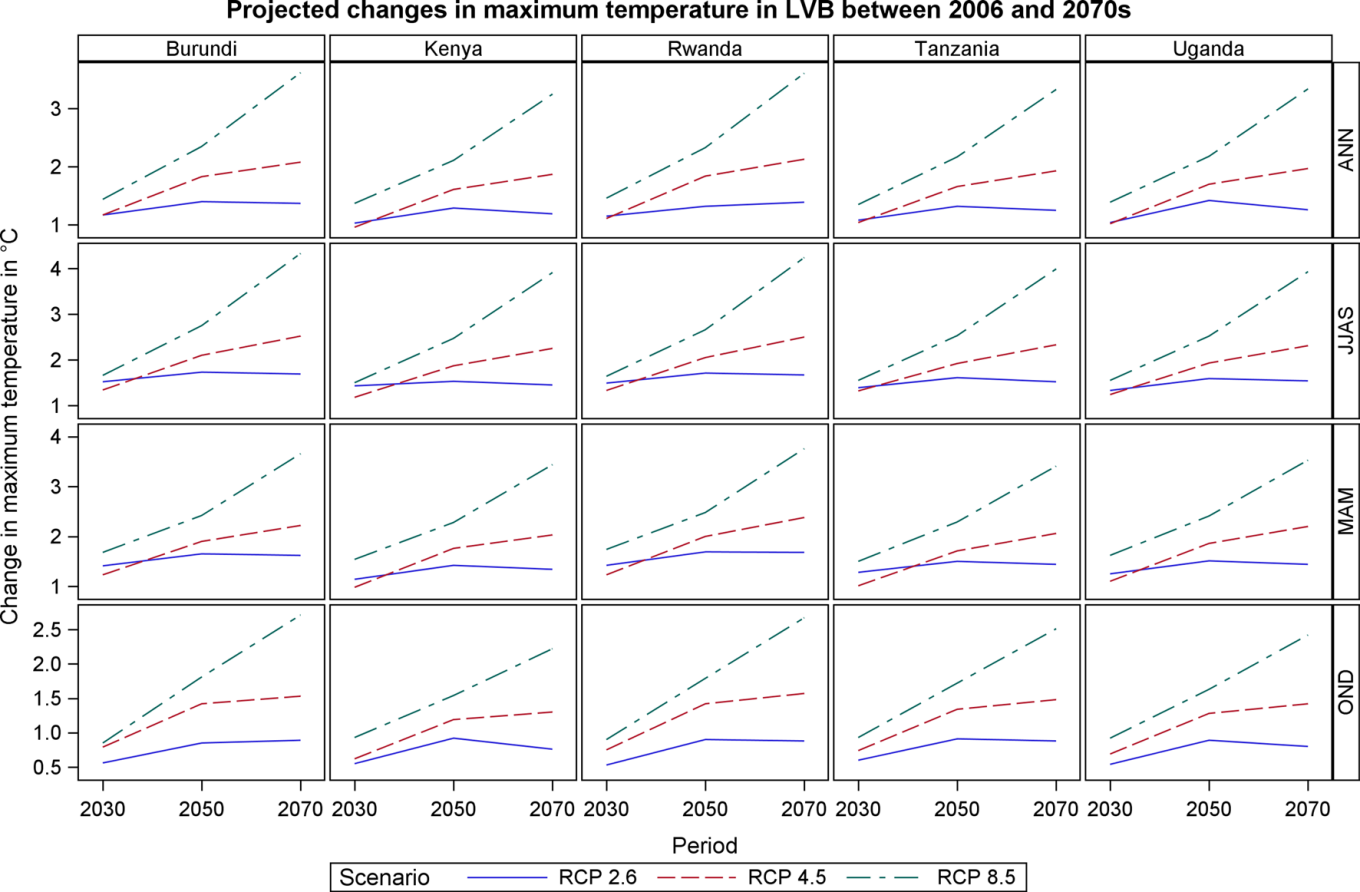
Projected changes in maximum temperature in LVB between 2006 and 2070s

**Fig. 5:**
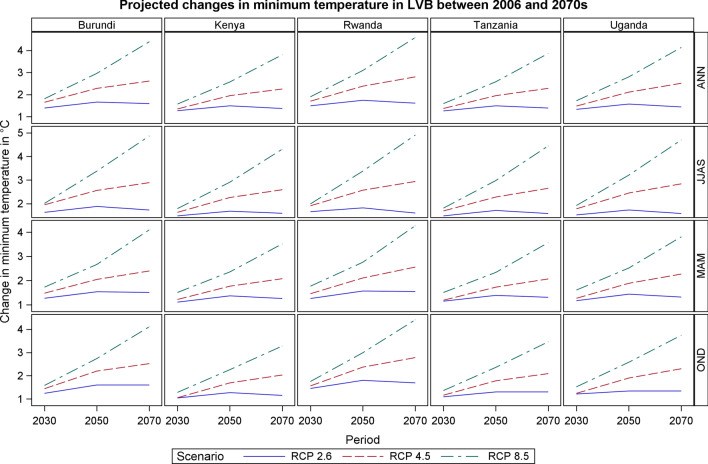
Projected changes in minimum temperature in LVB between 2006 and 2070s

The maximum temperature is projected to increase by at least 1.5 °C by 2050s in the rainy season months of March–May (MAM) under all the three scenarios across all the countries in the Lake Victoria Basin. Notably, the maximum temperature during MAM is expected to rise by up to 3.76 °C by 2050s under RCP 8.5. The highest extent of warming under all the scenarios is projected for the dry season months of June–September. During the dry season, maximum temperatures will likely rise by 1.5 °C under RCP 2.6 in 2030s and by up to 4.33 °C in 2070s under RCP 8.5. Maximum temperature warming in the short rainy season (October–December) is projected to be up to 2.7 °C under RCP 8.5 but to be minimal for all the other scenarios and time windows (Figs. 4, 5) (Table 3).

**Table 3:** Projected mean annual maximum temperature changes in LVB for the periods 2030s, 2050s, and 2070s for RCP2.6, 4.5 and 8.5

	RCP2.6			RCP4.5			RCP8.5		
	2030s	2050s	2070s	2030s	2050s	2070s	2030s	2050s	2070s
Annual									
Burundi	1.17	1.40	1.37	1.17	1.83	2.08	1.44	2.35	3.62
Kenya	1.03	1.29	1.19	0.96	1.61	1.87	1.37	2.11	3.25
Rwanda	1.15	1.32	1.39	1.11	1.84	2.13	1.46	2.33	3.61
Uganda	1.04	1.42	1.26	1.02	1.70	1.97	1.39	2.18	3.34
Tanzania	1.08	1.32	1.25	1.04	1.66	1.93	1.35	2.17	3.33
MAM									
Burundi	1.41	1.65	1.62	1.23	1.90	2.22	1.68	2.42	3.66
Kenya	1.14	1.42	1.34	0.98	1.76	2.03	1.54	2.28	3.44
Rwanda	1.42	1.69	1.68	1.23	2.00	2.38	1.74	2.48	3.76
Uganda	1.25	1.51	1.44	1.10	1.86	2.20	1.62	2.41	3.53
Tanzania	1.28	1.50	1.44	1.01	1.71	2.06	1.50	2.29	3.41
JJAS									
Burundi	1.52	1.73	1.69	1.34	2.10	2.52	1.66	2.75	4.33
Kenya	1.43	1.53	1.45	1.18	1.87	2.25	1.50	2.47	3.91
Rwanda	1.49	1.71	1.67	1.33	2.05	2.50	1.64	2.66	4.24
Uganda	1.33	1.59	1.54	1.24	1.93	2.31	1.55	2.52	3.93
Tanzania	1.39	1.61	1.52	1.32	1.92	2.33	1.55	2.53	3.99
OND									
Burundi	0.56	0.85	0.89	0.79	1.42	1.53	0.85	1.81	2.71
Kenya	0.55	0.92	0.76	0.62	1.19	1.30	0.93	1.54	2.22
Rwanda	0.53	0.90	0.88	0.75	1.42	1.57	0.90	1.79	2.67
Uganda	0.54	0.89	0.80	0.69	1.28	1.42	0.92	1.63	2.42
Tanzania	0.60	0.91	0.88	0.74	1.34	1.48	0.93	1.72	2.51

The projected changes in the minimum temperatures through time for the three scenarios (Table 4) suggest a larger increase in the minimum than the maximum temperature component in future. By 2030, almost all the EAC region will likely be 1.0–2.5 °C warmer than the base period, with the greatest warming expected during the dry season months (JJAS). JJAS is projected to have the highest increase in minimum temperatures, above 1.5 °C under RCP 2.6, above 2.5 °C in RCP 4.5 and above 3.0 °C in RCP 8.5. In the rainy season months of MAM and OND, RCP 4.5 and RCP 8.5 scenarios are projected to have an increase in minimum temperatures of above 2.0 °C. By 2070, the projected increase in the annual minimum temperatures will likely be 4–5 °C higher under the RCP 8.5 scenarios relative to the base period (Table 4).

**Table 4:** Projected mean annual minimum temperature changes in the Lake Victoria Basin for the periods 2030s, 2050s, and 2070s for RCPs 2.6, 4.5 and 8.5

	RCP2.6			RCP4.5			RCP8.5		
	2030s	2050s	2070s	2030s	2050s	2070s	2030s	2050s	2070s
Annual									
Burundi	1.39	1.66	1.59	1.65	2.28	2.61	1.81	2.96	4.39
Kenya	1.27	1.49	1.37	1.35	1.95	2.25	1.57	2.57	3.80
Rwanda	1.49	1.74	1.61	1.70	2.38	2.80	1.90	3.09	4.58
Uganda	1.33	1.57	1.44	1.48	2.11	2.51	1.72	2.80	4.13
Tanzania	1.26	1.49	1.39	1.37	1.95	2.28	1.59	2.58	3.86
MAM									
Burundi	1.27	1.54	1.51	1.48	2.05	2.40	1.73	2.67	4.10
Kenya	1.11	1.37	1.26	1.22	1.77	2.08	1.51	2.36	3.52
Rwanda	1.26	1.57	1.55	1.46	2.11	2.56	1.77	2.76	4.27
Uganda	1.17	1.44	1.32	1.27	1.89	2.27	1.61	2.52	3.81
Tanzania	1.15	1.39	1.31	1.19	1.73	2.07	1.51	2.34	3.57
JJAS									
Burundi	1.63	1.88	1.73	1.95	2.56	2.89	2.01	3.38	4.87
Kenya	1.48	1.68	1.59	1.63	2.26	2.59	1.79	2.91	4.31
Rwanda	1.66	1.82	1.60	1.91	2.57	2.94	1.99	3.38	4.91
Uganda	1.52	1.73	1.58	1.78	2.45	2.84	1.92	3.21	4.70
Tanzania	1.48	1.71	1.58	1.69	2.28	2.65	1.81	2.98	4.45
OND									
Burundi	1.24	1.60	1.60	1.44	2.20	2.52	1.58	2.74	4.11
Kenya	1.04	1.27	1.15	1.05	1.69	2.03	1.28	2.26	3.28
Rwanda	1.45	1.80	1.69	1.56	2.37	2.78	1.75	2.99	4.41
Uganda	1.21	1.34	1.34	1.24	1.90	2.30	1.52	2.58	3.74
Tanzania	1.09	1.30	1.30	1.16	1.78	2.09	1.36	2.36	3.46

### Projected Rainfall Changes in LVB

**Fig. 6:**
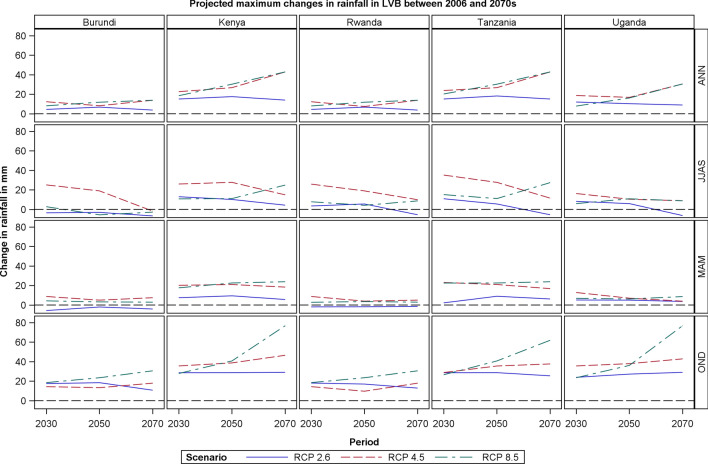
Projected changes in maximum rainfall in LVB between 2006 and 2070s

Rainfall projections vary across the five LVB countries both spatially and temporarily. In general, the OND short rains will likely increase compared to the long rains falling in MAM for all the three RCPs (Fig. 6). The JJAS season will likely be much drier for RCP 2.6 compared to RCP 4.5 and RCP 8.5 for all the periods. The increase in rainfall during OND, especially under RCP 4.5 and RCP 8.5 for the periods 2050s and 2070s, makes a large contribution to the anticipated increase in the annual rainfall in the LVB especially on its eastern section.

### Relationship Between Malaria and Historical Climate Fluctuations

**Fig. 7:**
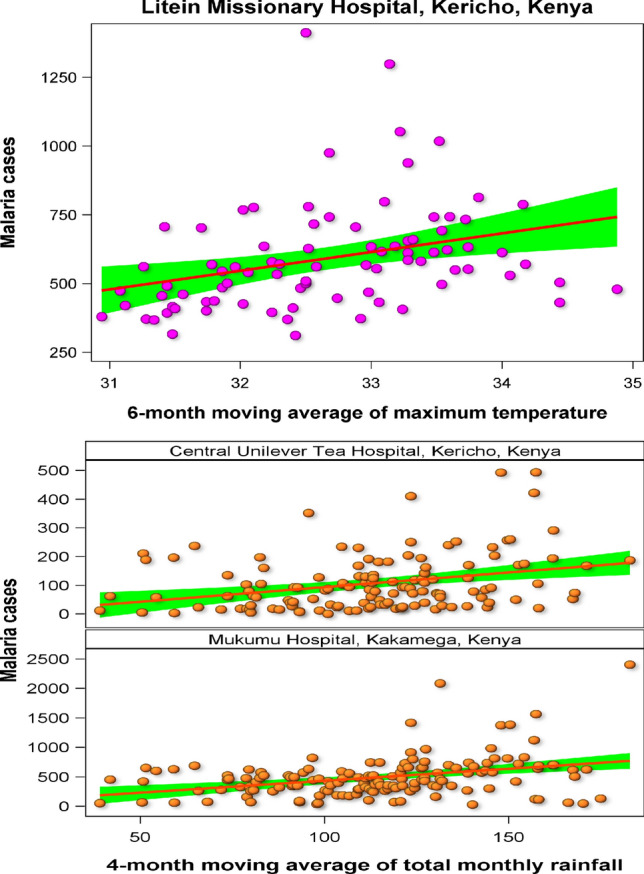
Regression relationships between the 6-month moving average of the average monthly maximum temperature and reported malaria cases in Litein hospital located in Kericho and the 4-month moving average of the total monthly rainfall and reported malaria cases in Central Unilever hospital located in Kericho and Mukumu hospital in Kakamega in Kenya

Extreme rainfall events reported in Kenya in 1997–1998 were related to the upsurge of malaria cases in Central Unilever, Litein and Mukumu hospitals (SI 11). We established linear relationships between malaria incidences and rainfall and 4-month running means of the total monthly rainfall in Unilever and Mukumu hospitals. These relationships suggest that malaria incidences increase linearly with increase in the 4-month running mean of the total monthly rainfall. In contrast, malaria incidences in Litein hospital increased linearly with increase in the 6-month running mean of the average monthly maximum temperature (Fig. 7, Table 5).

**Table 5:** Results of the linear regression of malaria cases on running means of rainfall and temperature for three hospitals in Kenya

Hospital	Effect	Estimate	SE	*df*	*T*	*P* >|*T*|
Litein Missionary Hospital, Kericho	Intercept	− 1628.3929	737.6111	82	− 2.208	0.030062
	Mavmaxtemp6	67.9544	22.5784	82	3.010	0.003474
Mukumu Hospital, Kakamega	Intercept	25.2664	106.4551	143	0.237	0.81273
	Mavrain4	4.0528	0.9025	143	4.491	1.45 × 10^–5^
Central Unilever Tea Hospital, Kericho	Intercept	− 9.0479	32.4533	129	− 0.279	0.780847
	Mavrain4	1.0193	0.2781	129	3.666	0.000359

A numeric suffix in rainfall or temperature component name denotes the time window in months over which the running average was computed

Data from Tanzania showed that the 1997–1998 El Niño episode, one of the strongest on instrumental record, was associated with a pronounced upsurge in reported malaria incidences. The analyses show that more malaria cases were reported in Muleba for all ages and 5-year-olds and above during 1997–1998 than for any other year, providing direct evidence that high rainfall is associated with increased malaria incidences (SI 12). Higher incidences of anaemia in the under-fives were also reported for that period than for the other years. Anaemia cases are related to malaria infections (SI 13).

**Fig. 8:**
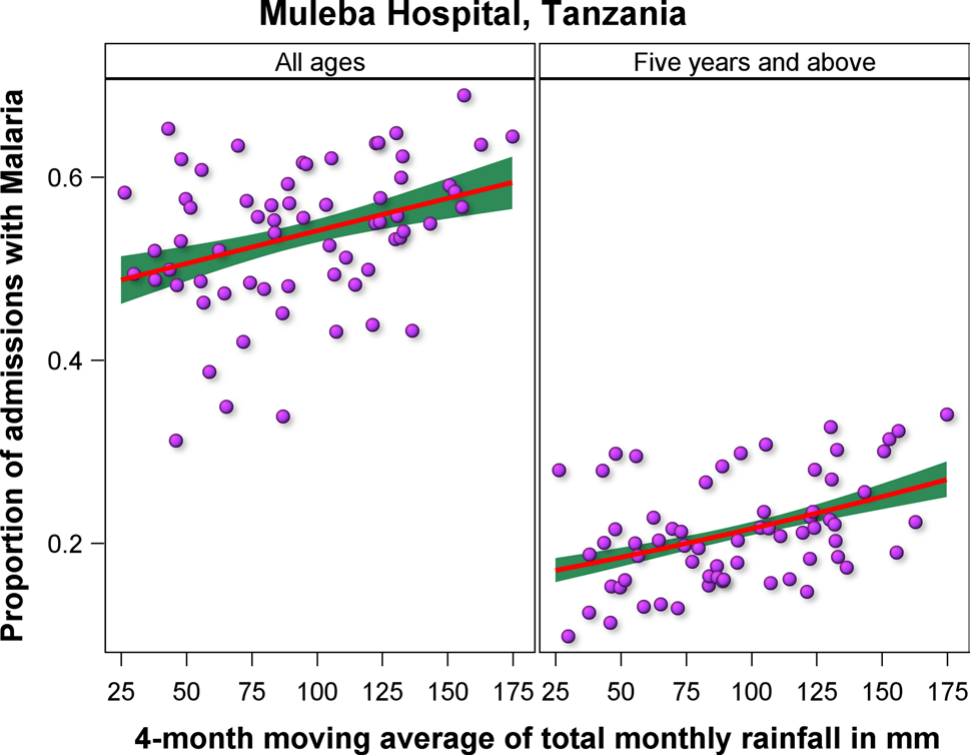
Regression relationships between reported malaria cases and the 4-month moving average of the total monthly rainfall for all ages and 5 years and above for Muleba hospital in Tanzania

Further, statistical analyses of the data from Muleba hospital established positive and significant correlations and regression relationships between malaria incidence and the 4–5-month running means of the total monthly rainfall and 3–5-month running means of the average monthly maximum temperature (Fig. 8). This suggests that upsurges in malaria cases will likely not occur immediately after high rainfall or maximum temperature events but rather will also respond to the carry-over effects of prior rainfall conditions experienced up to 4 months earlier. This delayed or lagged effect of rainfall on malaria cases reflects a delayed response linked to the vector life cycle and disease transmission cycle (Table 6).

**Table 6:** Results of the linear regression of malaria cases on running means of rainfall and temperature for Muleba hospital, Tanzania

Age	Effect	Estimate	SE	DF	T	P >|T|
All ages	Intercept	− 0.1227	0.0674	63.0	− 1.821	0.07339
All ages	Mavrain4	0.0029	0.0007	63.0	4.316	5.71 × 10^–5^
5 years and above	Intercept	− 1.6848	0.0598	63.0	− 28.176	2.04 × 10^–37^
5 years and above	Mavrain4	0.0039	0.0006	63.0	6.841	3.77 × 10^–9^
Under 5 years	Intercept	− 3.3353	1.4992	52.0	− 2.225	0.030467
Under 5 years	Mavmaxtemp4	0.0910	0.0523	62.8	1.739	0.087013

A numeric suffix in rainfall or temperature component name denotes the time window in months over which the running average was computed

**Fig. 9:**
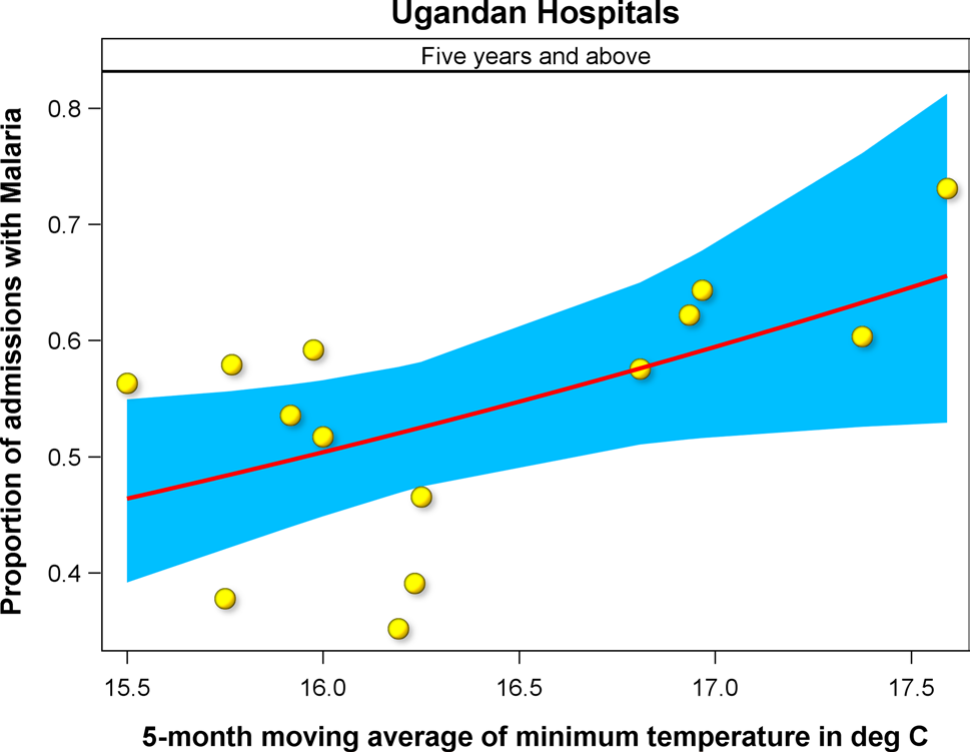
Regression relationship between reported malaria cases and the 5-month moving average of the average monthly minimum temperature for 5 years and above in Uganda

Country-wide data on malaria cases in Uganda spanning 1997–2010 show that far more children aged 5 years and above were affected than children below 5 years of age (SI 14). The trends for both age classes demonstrate a persistent decline in the number of cases following the 1997–1998 El Niño floods up to a low in 2003 followed by a persistent rise in the number of cases thereafter. It seems likely that a high investment in prevention measures, such as use of nets, following the 1997–1998 rains had a positive effect for both age classes but this was not maintained after about 5 years hence compromising the overall benefits of the measures. Figure 9 shows a significant regression relationship between malaria cases and the 5-month running mean of the average monthly minimum temperature.

The relationship shows that malaria cases recorded in the various districts of Uganda increased with either increasing minimum temperatures or rainfall depending on age class. Thus, malaria cases increased with increasing rainfall for the under-five age group, but increased with increase in the 5-month moving average of the minimum temperature for the 5 years and above age group (Fig. 9, Table 7, SI 14).

**Table 7:** Results of the regression of malaria cases reported for Uganda on rainfall and temperature

Age	Effect	Estimate	SE	*df*	*T*	*P* >|*T*|
Under 5 years	Intercept	− 1.7430	0.5901	12	− 2.954	0.012057
Under 5 years	Mavannual2	0.0006	0.0005	12	1.178	0.261772
5 years and above	Intercept	− 3.3344	1.1720	12	− 2.845	0.014757
5 years and above	Lagmintemp5	0.1655	0.0715	12	2.315	0.039130

A numeric suffix in rainfall or temperature component name denotes the time window in months over which the running average was computed

### Malaria Projections for the Kenyan Section of the LVB over 2006–2100

**Fig. 10:**
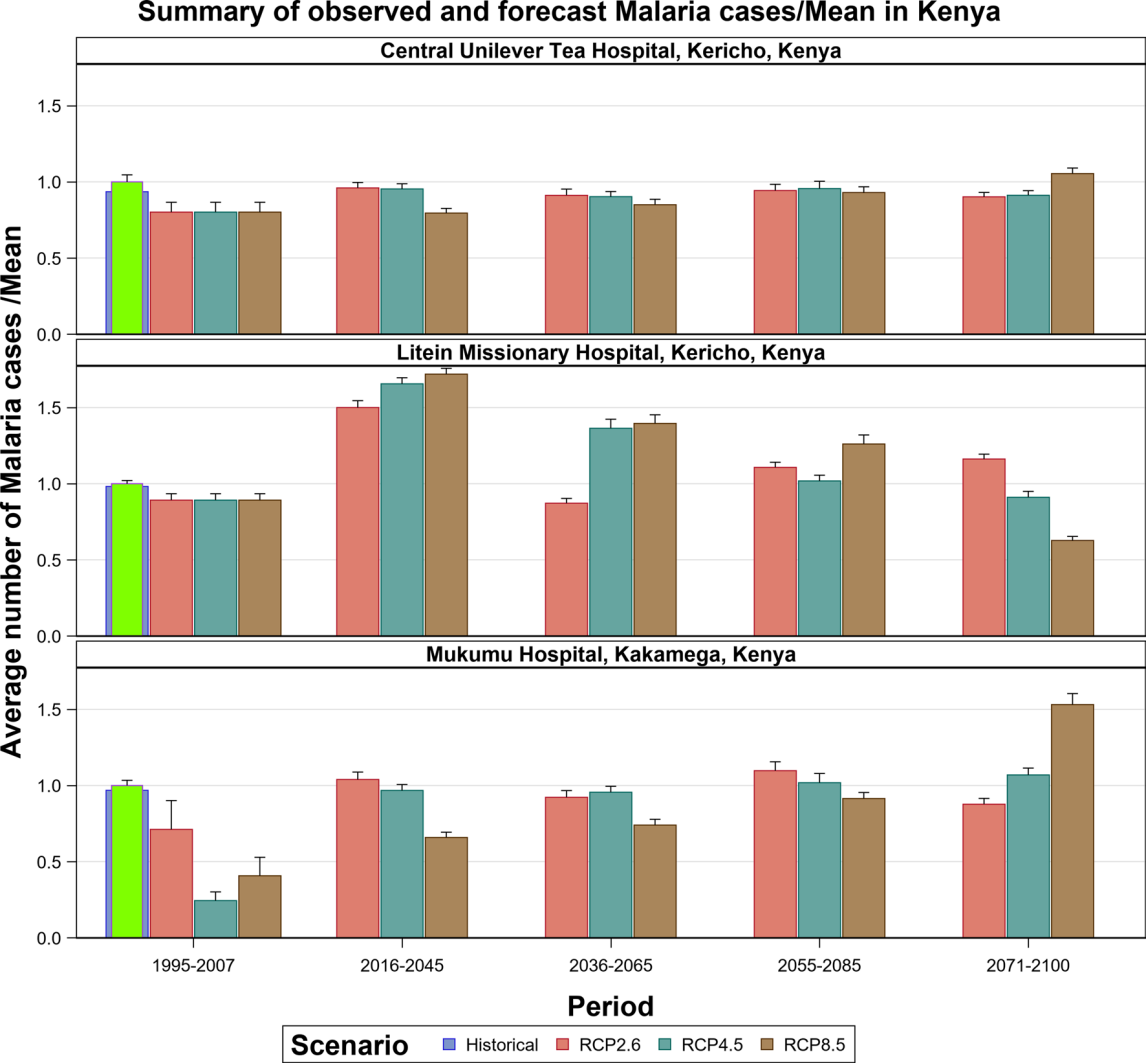
Summary of observed and forecast malaria cases/mean in Kenya for the period 1995–2100 under RCP2.6, RCP4.5 and RCP8.5 scenarios based on GCM model 1 in Table 2

**Fig. 11:**
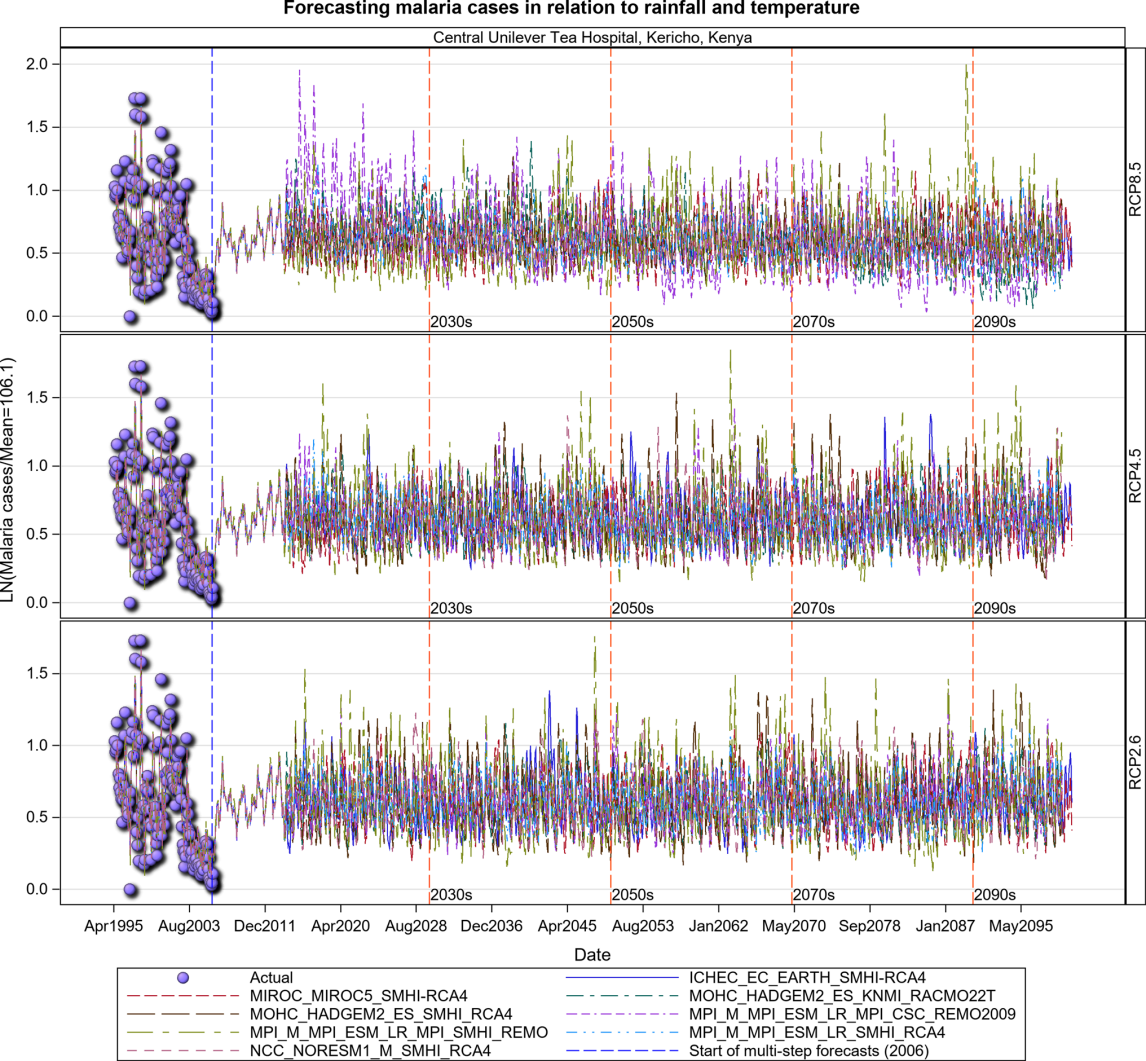
Malaria cases projected for Central Unilever hospital in Kericho, Kenya, for the period 2008–2100 under the RCP2.6, RCP4.5 and RCP8.5 scenarios, using an ensemble of 8 general circulation models

**Fig. 12:**
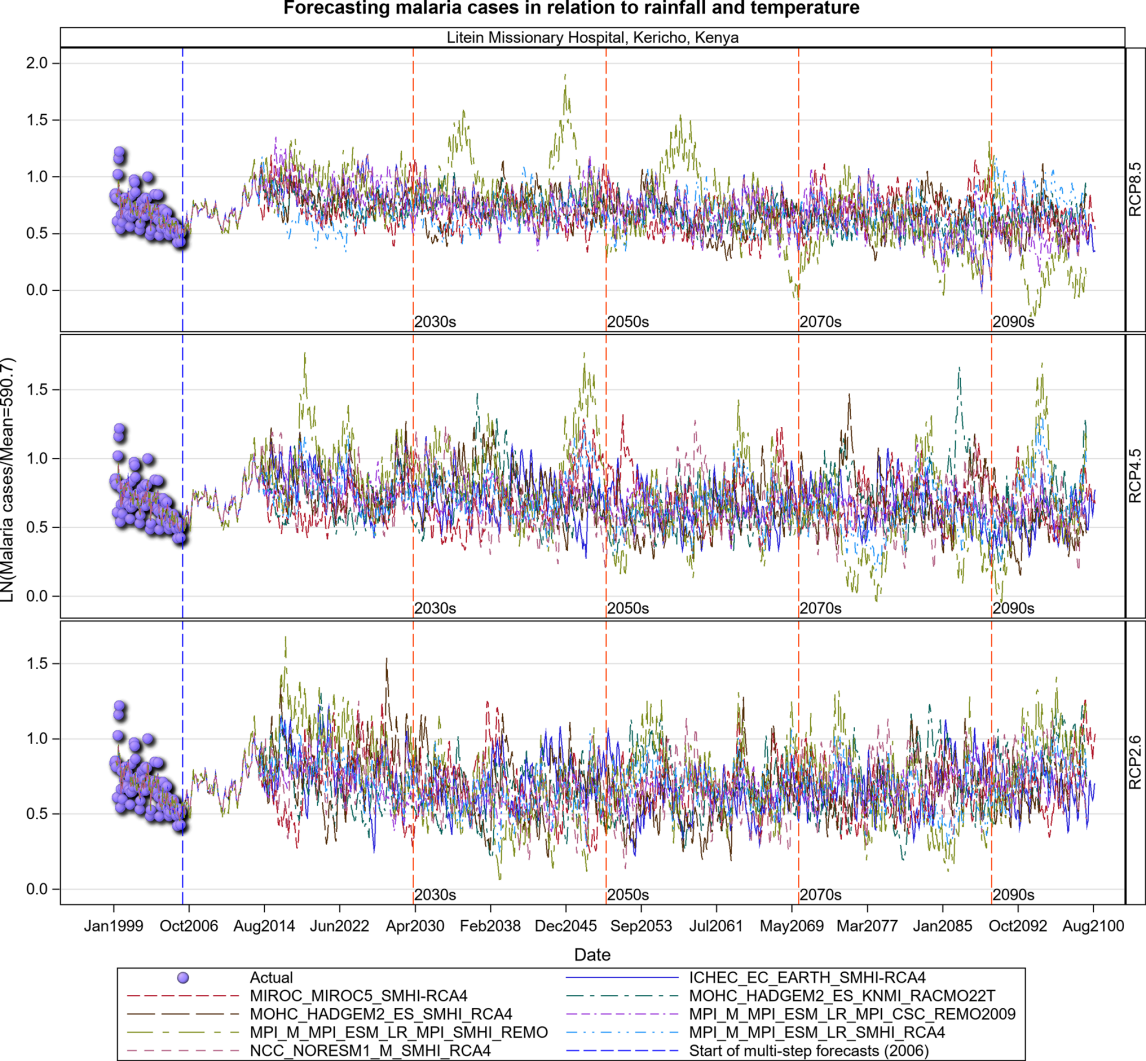
Malaria cases projected for Litein hospital in Kericho, Kenya, for the period 2008–2100 under the RCP2.6, RCP4.5 and RCP8.5 scenarios, using an ensemble of 8 general circulation models

**Fig. 13:**
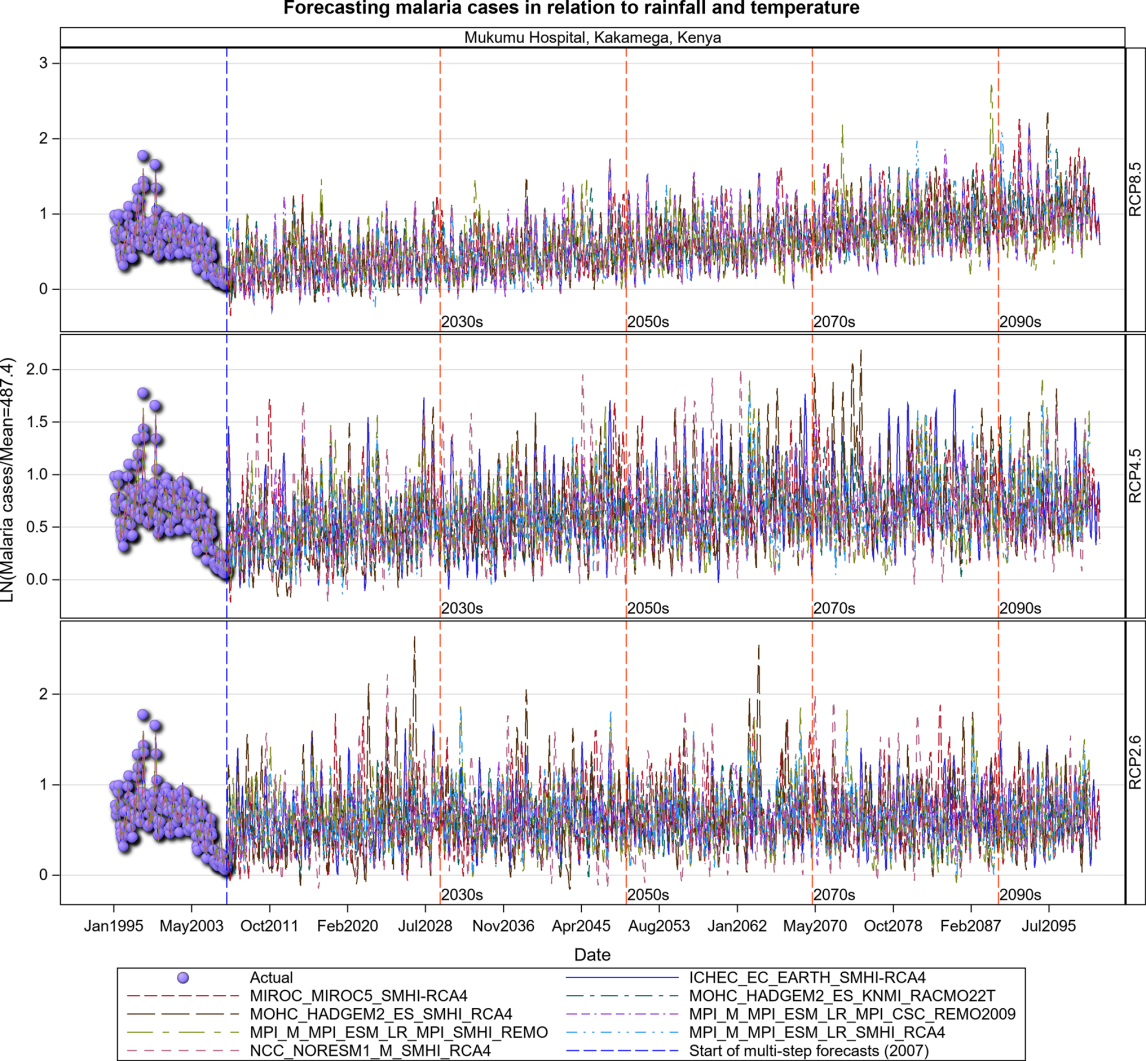
Malaria cases projected for Mukumu hospital in Kakamega, Kenya, for the period 2008–2100 under the RCP2.6, RCP4.5 and RCP8.5 scenarios, using an ensemble of 8 general circulation models

The transmission of the malaria parasite in Central Unilever and Litein Missionary hospitals showed strong quasi-cyclic fluctuations in all the three scenarios, with evidently time varying amplitudes and phases. Moreover, there were evident variations in the timings and amplitudes of the oscillations across the eight different GCM trajectories but no apparent temporal trend in the projected malaria or anaemia cases. Historical cases in Litein Missionary Hospital responded to changes in temperature. In Mukumu, the trajectory of the projected transmission portrays an increase in transmission, implying more cases can be expected in the future, especially under the RCP 4.5 and RCP 8.5 scenarios (Figs. 10, 11, 12, 13, SI 15–18). Under RCP 8.5, the observed cases were high in 1995–2007, the period when the 1997–1998 El Niño was experienced. During the 2008–2030 period, malaria cases will likely be reduced due to the efforts of integrated malaria control that are currently underway. There will likely be a marked variability in both the incidence and prevalence of malaria cases under the three scenarios in the future. In the worst-case scenario (RCP 8.5), malaria cases are projected to increase (Figs. 10, 11, 12, 13). The RCP 8.5 scenario shows that there will likely be a steady increase in the mean malaria cases reported at the hospitals over time as a result of the anticipated widening variability in rainfall and temperature (Figs. 10, 11, 12, 13).

### Malaria Projections for the Tanzanian Section of the LVB over 2016–2100

**Fig. 14:**
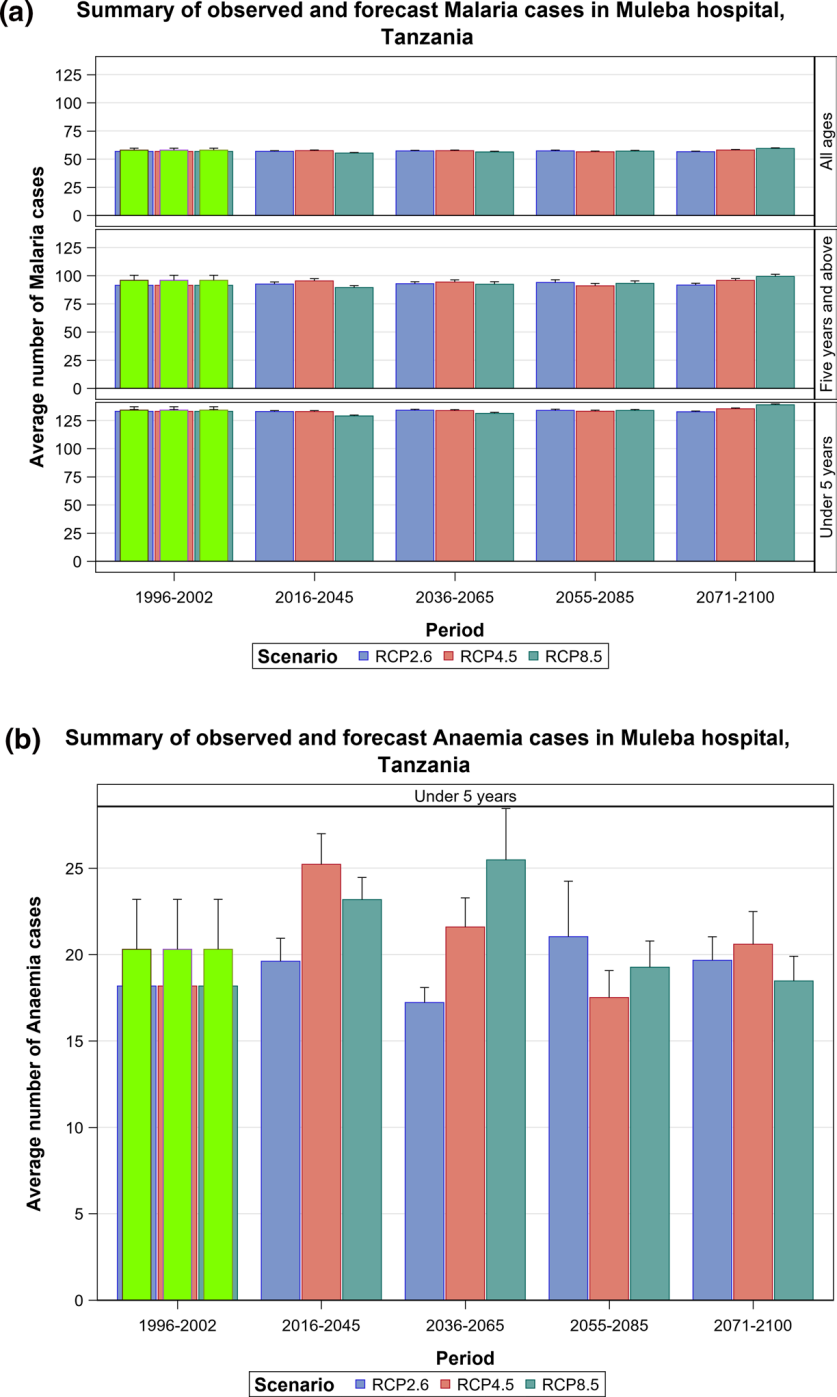
Summary of observed and forecast **a** malaria cases/mean and **b** anaemia cases/mean in Tanzania for the period 1995–2100 under RCP2.6, RCP4.5 and RCP8.5 scenarios based on GCM

**Fig. 15:**
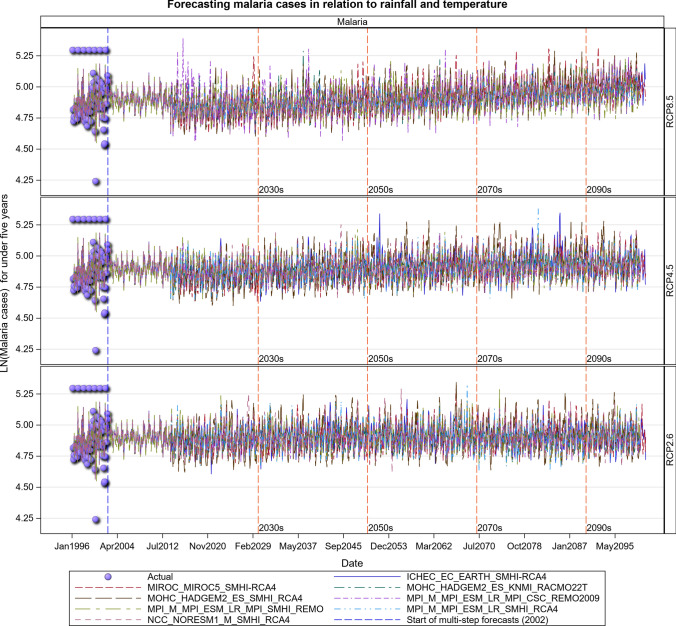
Malaria cases among under 5-year old’s projected for Muleba hospital, Tanzania, for the period 2006–2100 under the RCP2.6, RCP4.5 and RCP8.5 scenarios, using an ensemble of 8 general circulation models

**Fig. 16:**
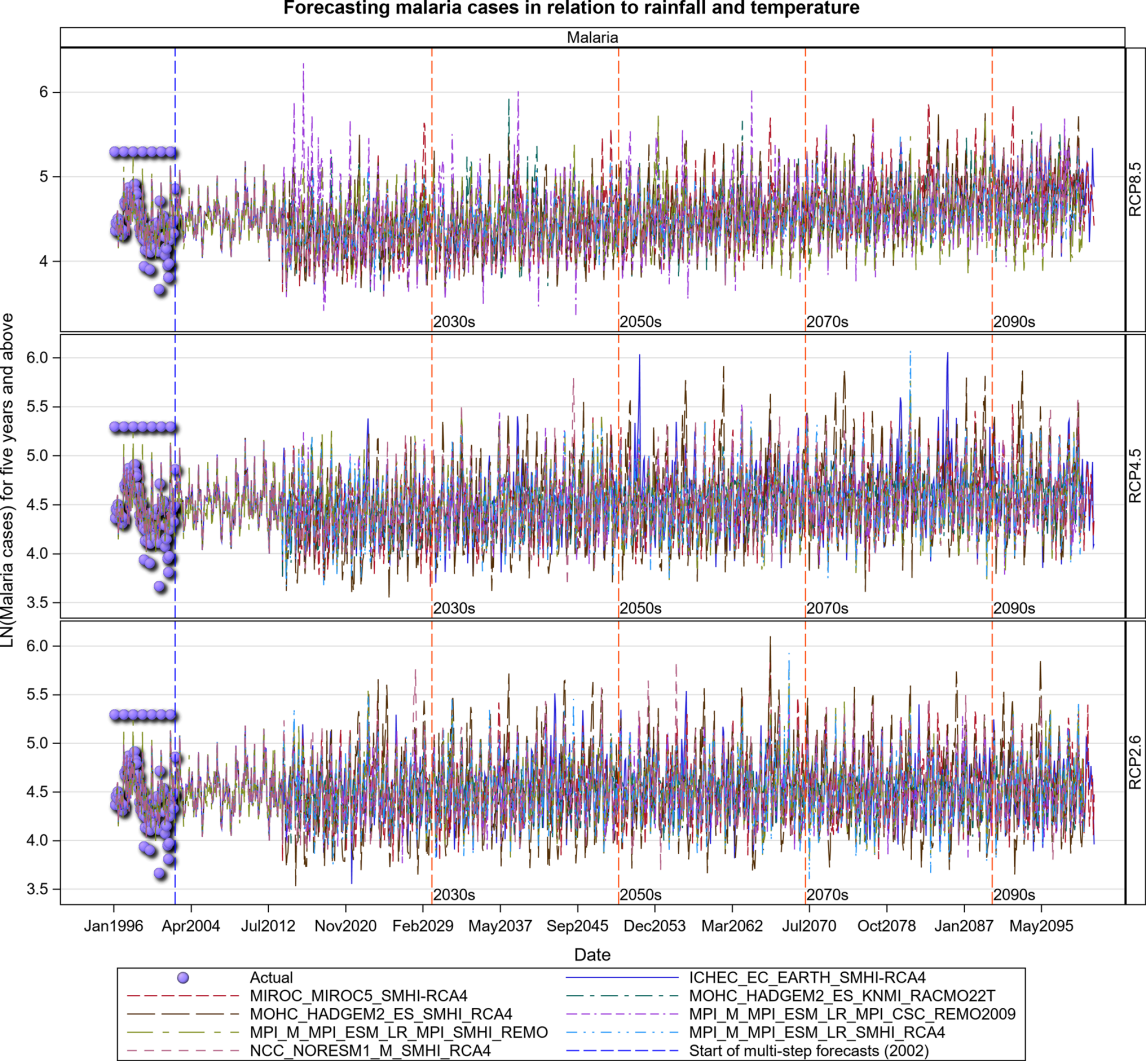
Malaria cases among 5-year old’s and above projected for Muleba hospital, Tanzania, for the period 2006–2100 under the RCP2.6, RCP4.5 and RCP8.5 scenarios, using an ensemble of 8 General Circulation Models

**Fig. 17:**
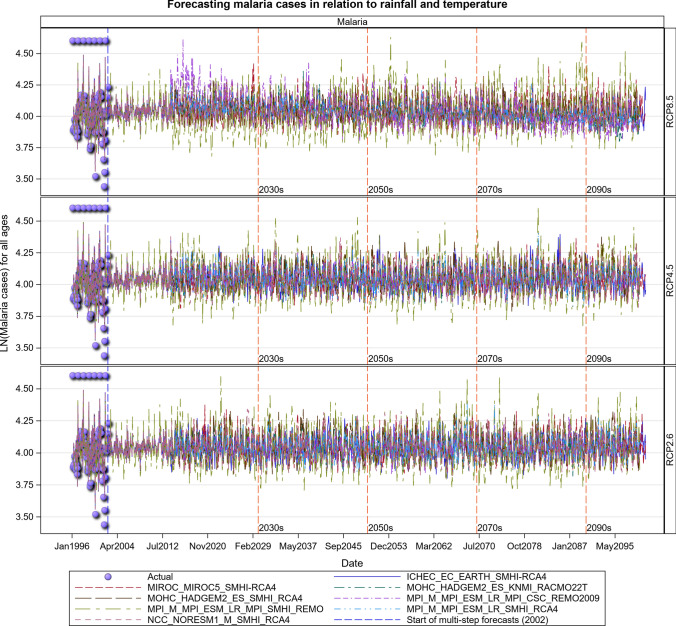
Malaria cases among people of all ages projected for Muleba hospital, Tanzania, for the period 2006–2100 under the RCP2.6, RCP4.5 and RCP8.5 scenarios, using an ensemble of 8 general circulation models

**Fig. 18:**
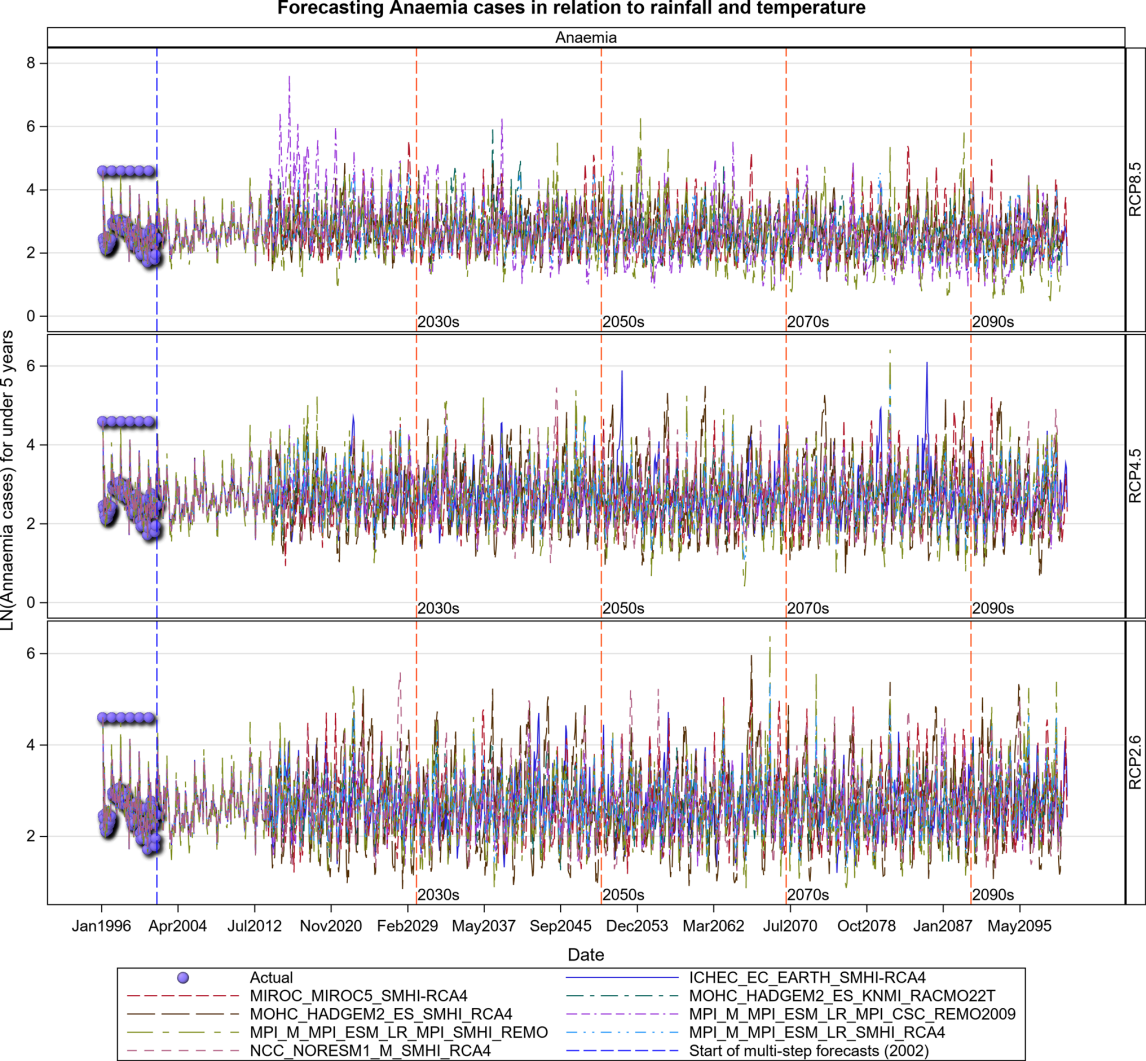
Anaemia cases among under 5-year old’s projected for Muleba hospital in Tanzania, for the period 2006–2100 under the RCP2.6, RCP4.5 and RCP8.5 scenarios, using an ensemble of 8 general circulation models

The Under-five age group responded to increases in maximum temperatures and increased over time most especially under the RCP 8.5 scenario. The malaria cases in this age group will likely increase steeply in the future. The 5 years and above age group also shows an increase in malaria cases in both the RCP 4.5 and RCP 8.5 scenarios, with the steepest increase projected for the RCP 8.5 scenario. More rainfall in the future will likely result in increased malaria and anaemia cases in this and all the other age groups (Figs. 14, 15, 16, 17, 18, SI 19–21). Similar projections of future increases in malaria cases for the 5 years and above age group under the RCP 8.5 scenario are apparent for Muleba (Figs. 14a, 16).

### Malaria Projections for the Ugandan Section of the LVB over 2006–2100

**Fig. 19:**
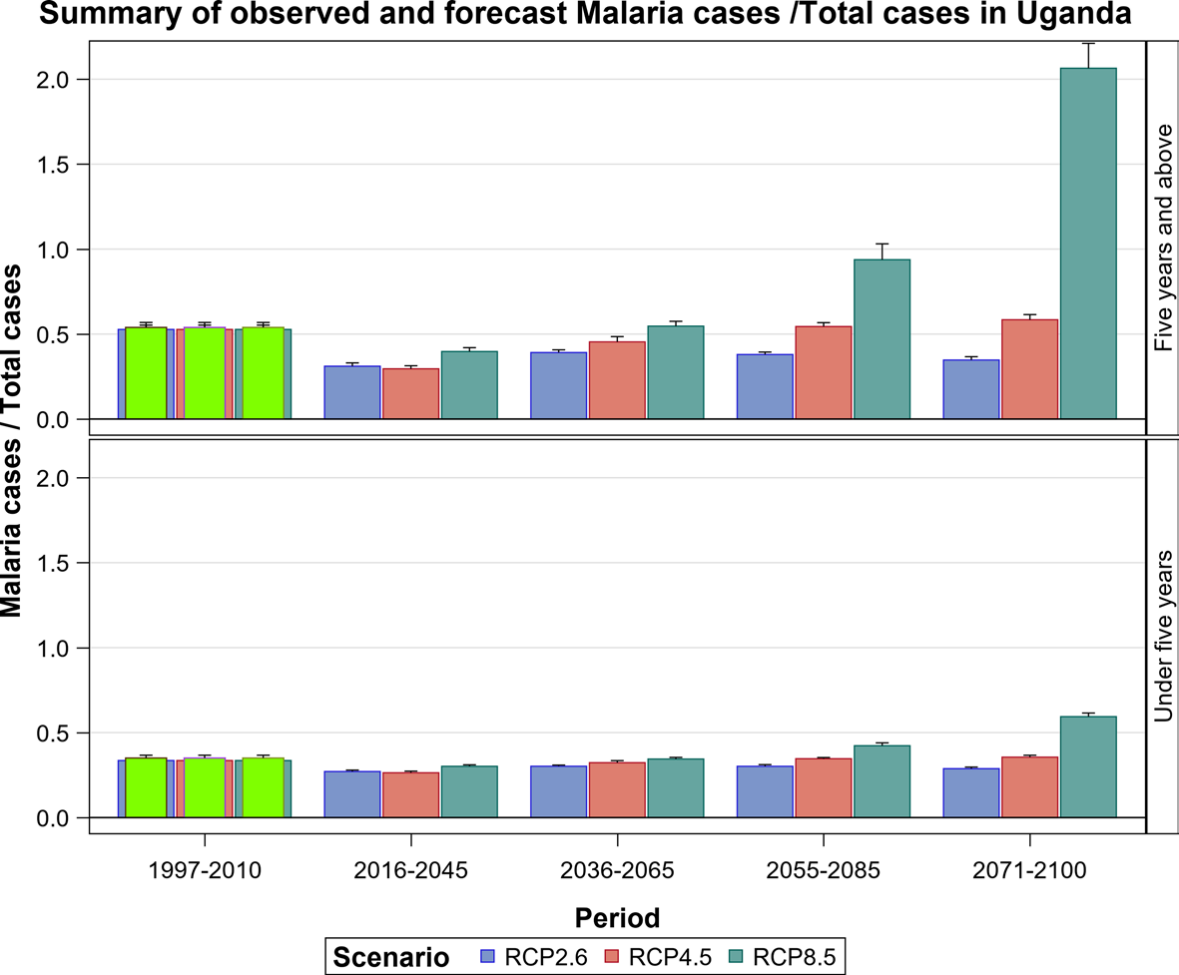
Summary of observed and forecast malaria cases/total cases in Uganda for the period 1995–2100 under RCP2.6, RCP4.5 and RCP8.5 scenarios

**Fig. 20:**
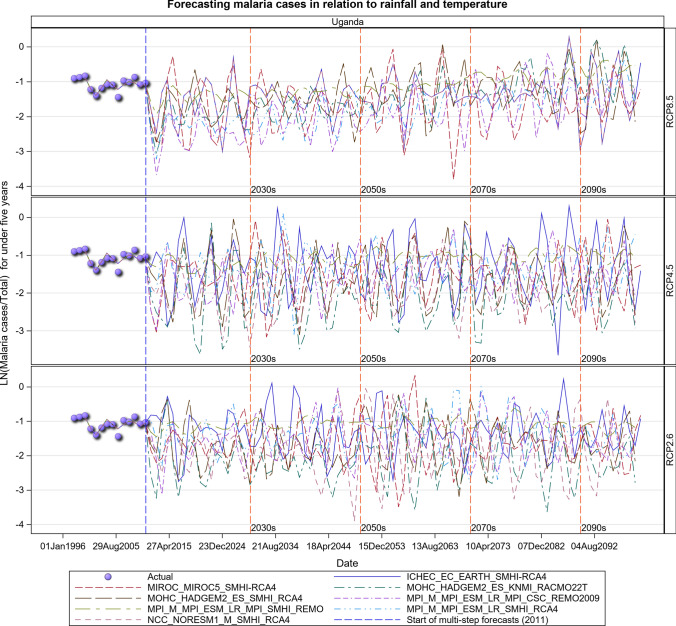
Malaria cases among under 5-year old’s projected for all the hospitals in Uganda, for the period 2011–2100 under the RCP2.6, RCP4.5 and RCP8.5 scenarios, using an ensemble of 8 general circulation models

**Fig. 21:**
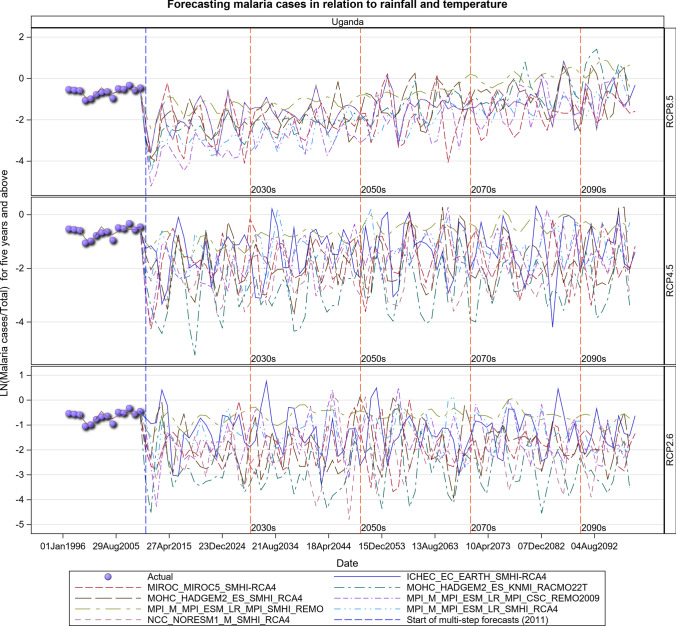
Malaria cases among 5-year old’s and above projected for all the hospitals in Uganda, for the period 2011–2100 under the RCP2.6, RCP4.5 and RCP8.5 scenarios, using an ensemble of 8 general circulation models

**Fig. 22:**
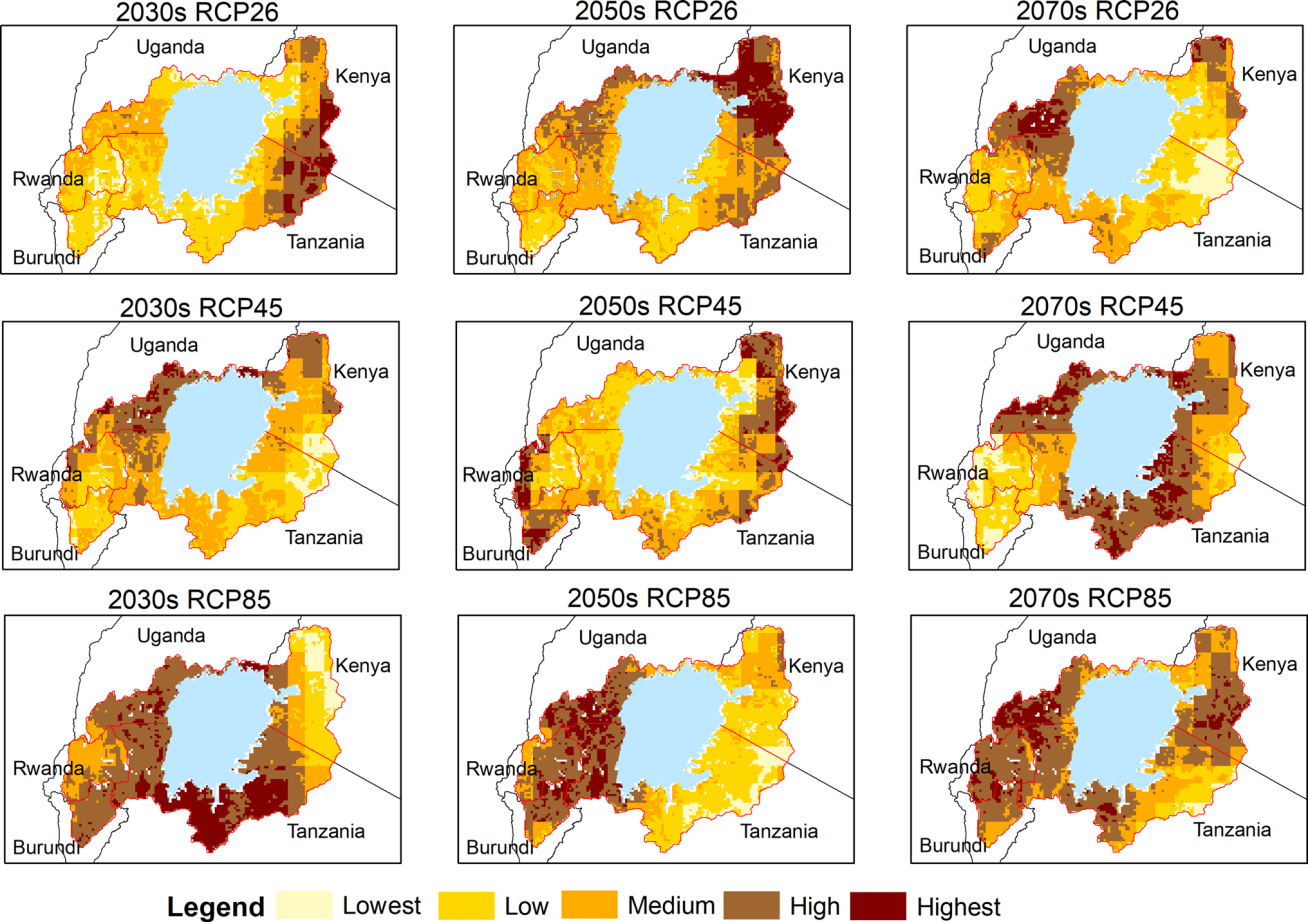
Projected malaria vulnerability maps for the 2030s, 2050s and 2070s under the three scenarios RCP2.6, RCP4.5 and RCP8.5 with projected population in the LVB

The observed malaria cases decreased during 2009–2010, but the average level of transmission in the future is expected to remain stable under the RCP 2.6 scenario. However, considerable inter-annual fluctuations in the level of transmission can be expected as can an increase in cases depending on rainfall and temperature patterns under the RCP 2.6 scenario (Figs. 19, 20, 21, SI 22). The RCP 4.5 and RCP 8.5 scenarios show an upward trend in malaria cases in the future. This is much more pronounced for the RCP 8.5 than the RCP 4.5 scenario (Figs. 19, 20, 21, 22, SI 23).

## Discussion

Climate change is expected to affect malaria transmission, and the extent to which the transmission will likely change should be established so that this can be appropriately addressed by the public health sector and governments. To understand these changes, we examined future malaria scenarios for the Lake Victoria Basin in East Africa in response to climate variability based on three climate scenarios for 2006–2100 defined by the representative concentration pathways (RCPs) RCP 2.6, RCP 4.5, and RCP 8.5 and projected by an ensemble of eight GCMs.

It is crucial to minimize uncertainty in future projections to reliably assess anticipated influences of simulated climatic variability and change. This is often done by considering a range of plausible projection scenarios and using an ensemble of GCMs. However, there are typically too many potential sources of uncertainty to exhaustively consider in any single study in practice. Thus, even though we consider multiple countries, malaria transmission hotpots, rainfall, minimum and maximum temperature components, periods and seasons, we perform extensive model selection using the historical data and consider an ensemble of eight different future projections for each of the three scenarios to reduce uncertainty. We account for uncertainty in the projected future scenarios by considering several sources of uncertainty associated with the simulated scenarios, rainfall and temperature component uncertainty, model selection uncertainty relating to the functional form of the relationship between malaria or anaemia cases and rainfall and temperature, parameter uncertainty in the selected models and model projection uncertainty. To account for uncertainty in the projected future scenarios, we consider three RCP scenarios, including the best case (RCP 2.6), intermediate (RCP 4.5) and worst case (RCP 8.5) scenarios. Using these three scenarios allows us to capture future uncertainties inherent in contemplated future outcomes contingent upon contrasting contemporary policies. Although one may consider many other scenarios, we expect the projections a priori to fall between the two boundary scenarios (RCPs 2.6 and 8.5). For each scenario, we consider an ensemble of eight GCMs to account for projection model uncertainty.

We extensively consider model selection uncertainty in the regression relationships between the historical malaria and anaemia cases and historical rainfall and temperature components and use information theoretics, residual and influence diagnostics and statistical tests to choose between specific rainfall and temperature components (lags or cumulative moving averages) and functional forms of contending models relating malaria and anaemia cases to the selected rainfall and temperature components. First, we use the corrected Akaike Information Criterion plus residual and influence diagnostics and multiple other criteria to choose the models and rainfall and temperature components. Second, we quantify parameter uncertainty using standard errors of the model parameter estimates. Third, for each scenario and GCM combination, we use 95% pointwise confidence bands to quantify projection uncertainty. Lastly, we benchmark the performance of the projection models against the historical malaria (or anaemia) outbreak time series. In all the cases, we find reasonable agreements between the model projections and the historic time series but remarkable variation in the projected series across the ensemble of eight GCMs, reaffirming the importance of using ensemble projections for each scenario.

Our results suggest a likely greater increase in the minimum than the maximum temperatures during 2006–2100. Specifically, minimum temperatures will likely increase by 1–2.5 °C by 2030, and 4–5 °C by 2100 compared to the base period. The projected future warming trend is a regional manifestation of global warming and reinforces IPCC’s [10] projection that by 2100, the average global temperatures will likely have risen by 1–3.5 °C. We project a likely increase in the maximum and minimum temperature components for the three RCP scenarios compared to the base period in the Lake Victoria Basin. Rwanda and Burundi are projected to have some of the highest increases in both the minimum and maximum temperature components. However, the available clinical malaria data for Rwanda and Burundi were insufficient to project malaria cases under the three RCPs scenarios.

Rainfall is projected to increase in the short rainy season (OND) compared to the long rainy season (MAM) for RCP 4.5 and RCP 8.5 for the periods 2050s and 2070s and this will likely make a large contribution to the increase in the annual rainfall in the LVB, especially on its eastern section. The dry season will likely be much drier than in the base period. An increase in precipitation in the short rainy season will likely prolong the malaria transmission season in the Lake Victoria Basin due to the increase in the rain-fed vector breeding habitats [27, 28].

For the intermediate scenario, RCP 4.5, projections show that increased rainfall and warming of the highlands will likely increase the mean number of malaria cases. The most affected time periods will be 2008–2030. El Niño events were reported during 1994–1995, 1997–1998, 2002–2003, 2004–2005, 2006–2007, 2009 and 2015–2016 [29–31] and the projections clearly show an increase in malaria cases in the Lake Victoria Basin in the years when an El Niño event occurred. Epidemics were also reported during these years [32–34]. The under-fives still remains the most vulnerable group to malaria infections under the RCP 4.5 scenario.

These climatic changes have multiple implications for malaria transmission. Increased temperatures expected in the Lake Victoria Basin will likely elevate malaria transmission in the highlands by altering malaria vector ecology and biology [35, 36]. The anticipated temperature rise will likely reduce vector development time and also increase the rate of development of the malaria parasite inside the vector [37]. This will likely cause an increase in malaria transmission in areas where malaria already exists and also in areas that are already malaria transmission hotspots [38, 39]. The increase in minimum temperatures will likely increase the geographic spread of malaria parasites and malaria cases to new areas outside the present malaria distributional range. Thus, highland regions with average temperatures below 18 °C will likely experience new cases due to the projected increase in both the minimum and maximum temperatures [40]. The frequency and intensity of epidemics may thus be expected to increase in these regions. However, high temperatures expected in the lowlands may have a negative effect on holoendemic malaria transmission [37].

An increase in rainfall in the short rainy season (OND) will likely prolong the malaria transmission season and shift seasonal malaria transmission. More malaria cases will likely be reported during this season in the Lake Victoria Basin in future than in the base period. This will likely strain the health systems of the LVB countries as they will be forced to deal with more cases than are usually presented in hospitals during the short rainy season [41].

There will also likely be an increase in anaemia cases which are directly related to malaria infections and are a major cause of morbidity and mortality in the under-5 years [42, 43]. For every 130 cases of malaria reported in 1996, 20 anaemia cases were reported in the under-five age group. This trend is projected to increase in the future and to peak in 2051–2070. An increase in malaria cases in future will thus almost certainly lead to increased anaemia cases. Historical malaria cases in the region were strongly influenced by climate variability from the upsurge in the number of reported clinical cases after El Niño events in 1995–1996, 1997–1998 and 2002–2003. Malaria epidemics were reported during these years [44]. This shows that climate variability influences malaria transmission. Future climatic extremes will also likely cause similar upsurges in clinical malaria cases as evidenced by episodic spikes in the trajectory of the projected malaria cases suggesting epidemic outbreaks.

Despite the changes in climate and its variability, malaria interventions can prevent or reduce the impacts of climate change and reduce the incidence and prevalence of malaria in the Lake Victoria Basin [7, 45]. In 2006, distribution of bed nets to vulnerable groups, such as pregnant women and mothers with children under 5 years, reduced malaria prevalence significantly [46]. In 2011, the roll back malaria campaign for universal bed net distribution also reduced malaria prevalence significantly [47]. Targeted distribution of long-lasting insecticide treated nets in malaria hotspots has prevented outbreaks of epidemics in these regions [48]. Other interventions such as the use of Indoor residual spraying have a major impact in reducing malaria incidence and prevalence in the Lake Victoria Basin [49]. However, there is a risk of intervention failure mainly because of insecticide resistance, shift in biting time, less anthropophily, increased zoophilly and exophily and also a shift in species abundance [50, 51]. There is also a looming threat of drug resistance, but new vaccines, if effective, will hopefully be an added tool to the existing malaria control toolbox.

## Conclusions

Early detection of malaria epidemics and accurate future projections are major strategies in malaria control as they increase preparedness and reduce morbidity and mortality. Climatic changes are expected to greatly impact the highland regions of the Lake Victoria Basin as temperature rise will likely increase the survival rates of the malaria vectors and parasite transmission. Some highland regions will likely experience new cases of malaria transmission for the first time. Larger populations will likely be at risk and novel malaria hotspots will likely emerge. This calls for universal coverage of control interventions to minimize transmissions. Interventions should be restructured to address emerging risks of malaria transmission caused by increasing human vulnerability and suitability of new regions for malaria transmission. Future malaria research should prioritize understanding of the role of vector ecology on malaria, vector abundance and species change in response to climate change and widening variability. Widespread adoption of land uses that reduce vector habitats and lower temperatures should be promoted as adaptation and mitigation strategies for controlling malaria prevalence and spread in the anticipated warmer and wetter future climates.

## Supplementary Information

Below is the link to the electronic supplementary material.

Supplementary file1 (PDF 94026 KB)

Supplementary file2 (XLSX 94316 KB)

Supplementary file3 (XLSX 119246 KB)

Supplementary file4 (XLSX 2625 KB)

## Data Availability

The datasets used and/or analysed during the current study are provided in the supporting information in S1-S3 data.
