# Supplementary material for: Forecasting the Potential Effects of Climate Change on Malaria in the Lake Victoria Basin Using Regionalized Climate Projections
Source: Acta Parasitol. 2022 Aug 12;67(4):1535–63. doi: 10.1007/s11686-022-00588-4 (PMC9705490; doi:10.1007/s11686-022-00588-4)
Supplement: Supplementary file 1 — Supplementary file1 (PDF 94026 KB) [file 11686_2022_588_MOESM1_ESM.pdf]

Supplementary Information (SI)

SI 1: Statistical methods used for analysis and forecasting.

SI 2: Averages of historic rainfall and temperature data for locations of hospital in Kenya and Tanzania.

SI 3: Table on the historical period covered by the data for each hospital, projection periods, response variables and their transformations and predictor variables included in the VARMAX(p , q , s).

SI 4: Roots of the autoregressive (AR) and moving average (MA) characteristic polynomials. The modulus of the roots of its AR polynomial should be less than 1 for a time series to be stationary.

SI 5: Univariate Model White Noise Diagnostics for malaria and anaemia. The results test whether the residuals are correlated and heteroscedastic. The Durbin-Watson test statistics test the null hypothesis that the residuals are uncorrelated. The Jarque-Bera normality test tests the null hypothesis that the residuals are normally distributed. The F statistics and their p-values for ARCH(1) disturbances test the null hypothesis that the residuals have equal covariances.

SI 6: Univariate AR Model Diagnostics for malaria. The F statistics and their p-values for AR(1), AR(1,2), AR(1,2,3) and AR(1,2,3,4) models of residuals test the null hypothesis that the residuals are uncorrelated.

SI 7: Portmanteau Test for Cross Correlations of Residuals. The results show tests for white noise residuals based on the cross correlations of the residuals. Insignificant test results show that we cannot reject the null hypothesis that the residuals are uncorrelated.

SI 8: Univariate model ANOVA diagnostics for malaria and anaemia showing that each model is significant.

SI 9: Parameter estimates for the univariate VARMAX (p,q,s) models for malaria and anaemia. Model selection was based on information theory so insignificant coefficients are retained in the selected models. If a few of the insignificant coefficients are restricted to be zero, then many of the apparently insignificant coefficients become significant.

SI 10: SAS codes (Version 9.4) for fitting the VARMAX (Version 15.1) models to the Malaria and anaemia data and for producing graphs of the historical and projected series.

SI 11: Reported malaria cases in three Kenya hospitals from 1995 to 2007.

SI 12: Percentage of admissions with malaria in Muleba Hospital, Tanzania.

SI 13: Percentage of admissions with anaemia in Muleba Hospital, Tanzania.

SI 14: Temporal trends in reported malaria cases for five years and above and under five years in Uganda expressed as proportions.

SI 15: Historical and Projected malaria cases in Central Unilever Tea Hospital in relation to rainfall and temperature for the period 1995-2100 showing RCP2.6, RCP4.5 and RCP8.5 scenarios in 2030, 2050 and 2070 under 8 General Circulation Models.

SI 16: Historical and Projected malaria cases in Litein Missionary Hospital in relation to rainfall and temperature for the period 1995-2100 showing RCP2.6, RCP4.5 and RCP8.5 scenarios in 2030, 2050 and 2070 under 8 General Circulation Models.

SI 17: Historical and Projected malaria cases in Mukumu Hospital in relation to rainfall and temperature for the period 1995-2100 showing RCP2.6, RCP4.5 and RCP8.5 scenarios in 2030, 2050 and 2070 under 8 General Circulation Models.

SI 18: Historical and Projected anaemia cases for under five years in Muleba Hospital in relation to rainfall and temperature for the period 1996-2100 showing RCP2.6, RCP4.5 and RCP8.5 scenarios in 2030, 2050 and 2070 under 8 General Circulation Models.

SI 19: Historical and Projected malaria cases for under five years in Muleba Hospital in relation to rainfall and temperature for the period 1996-2100 showing RCP2.6, RCP4.5 and RCP8.5 scenarios in 2030, 2050 and 2070 under 8 General Circulation Models.

SI 20: Historical and Projected malaria cases for five years and above in Muleba Hospital in relation to rainfall and temperature for the period 1996-2100 showing RCP2.6, RCP4.5 and RCP8.5 scenarios in 2030, 2050 and 2070 under 8 General Circulation Models.

SI 21: Historical and Projected malaria cases for all ages in Muleba Hospital in relation to rainfall and temperature for the period 1995-2100 showing RCP2.6, RCP4.5 and RCP8.5 scenarios in 2030, 2050 and 2070 under 8 General Circulation Models.

SI 22: Historical and Projected malaria cases in Uganda for under five years in relation to rainfall and temperature for the period 1997-2100 showing RCP2.6, RCP4.5 and RCP8.5 scenarios in 2030, 2050 and 2070 under 8 General Circulation Models.

SI 23: Historical and Projected malaria cases for 5 years and above in Uganda in relation to rainfall and temperature for the period 1997-2100 showing RCP2.6, RCP4.5 and RCP8.5 scenarios in 2030, 2050 and 2070 under 8 General Circulation Models.

S1 Data. The General Circulation Models (GCMs), hospitals, scenarios (RCP2.6, RCP4.5, RCP8.5), period covered by the historical and projected malaria cases, rainfall and temperature components and the projections for each GCM and scenario combination and their associated 95% lower and 95% upper confidence bands for hospitals in Kenya**.** All the rainfall and temperature components for the historical period and for each GCM and Scenario combination have been divided by their corresponding means. The data transformations are described in table **SI 3**.

S2 Data. The General Circulation Models (GCMs), hospitals, scenarios (RCP2.6, RCP4.5, RCP8.5), period covered by the historical and projected malaria or anaemia cases, rainfall and temperature components and the projections for each GCM and scenario combination and their associated 95% lower and 95% upper confidence bands for Muleba hospital in Tanzania**.** All the rainfall and temperature components for the historical period and for each GCM and Scenario combination have been divided by their corresponding means. The data transformations are described in table **SI 3**.

S3 Data. The General Circulation Models (GCMs), hospitals, scenarios (RCP2.6, RCP4.5, RCP8.5), period covered by the historical and projected malaria cases, rainfall and temperature components and the projections for each GCM and scenario combination and their associated 95% lower and 95% upper confidence bands for all the hospitals in Uganda. All the rainfall and temperature components for the historical period and for each GCM and Scenario combination have been divided by their corresponding means. The data transformations are described in table **SI 3**.

SI 1

Statistical analysis and forecasting

Various regression approaches were used to accommodate possibly curvilinear relationships and non-Gaussian frequency distributions for response variables, such as proportion of admissions with malaria. This resulted in the use of standard normal-theory regressions and generalized linear models, including logistic and negative binomial regressions.

Malaria cases for Central Unilever Tea, Litein Missionary and Mukumu Hospitals in Kenya were related to lagged and moving averages of rainfall and minimum and maximum temperature using a negative binomial regression with a log link function in which the mean is a quadratic function of the mean. For Muleba Hospital in Tanzania, the number of anemia and malaria cases for January was reported as 100 for all years. The cases for February to December were reported as proportions of the number for January (P). Thus, the total number of cases for February to December are estimated as Cases=100P. The proportion of admissions with malaria for Muleba was related to lagged or cumulative moving averages of rainfall, minimum and maximum temperatures, separately for each age class, using logistic regression with a binomial error distribution and a logit link function. For the Ugandan hospitals, the total number of malaria cases per year was related to lagged or cumulative moving averages of rainfall, minimum and maximum temperatures, separately for each age class, using negative binomial regression, with the logarithm of the total number of admissions per year as an offset. The negative binomial and logistic regression models allowed for potential overdispersion to account for possible departures of the observed mean-variance relationships from the theoretical expectations for the binomial or negative binomial distribution models.

Each rainfall, minimum and maximum temperature component was divided by its respective long-term mean (S1 Table) for use in all statistical models for all the three hospitals in Kenya and Muleba hospital in Tanzania. Thus, we use only deviations from the long-term mean rainfall, minimum or maximum temperature and not their absolute values. For Uganda, the predictor variables were the untransformed moving averages values of rainfall, minimum and maximum temperatures. However, for projection all the predictors were divided by their means.

Forecasting of the likely future responses of each of the malaria or anemia cases to the projected rainfall and temperatures for each of the three scenarios was carried out using the vector (multivariate) autoregressive moving average processes, VARMAX (*p,q,s*) (Engle and Granger 1987, Hosking 1980, Johansen 1995, Engle 2002). This model can be used to fit and forecast a univariate time series when only a single response series (dependent, response, or endogenous variable) is under consideration or vector processes (i.e., multiple time series) by assuming that at each time point the observations consist of a vector of responses. Each of the responses can depend on its own lagged values and lags of the other vector entries. The VARMAX (*p,q,s*) models we used allow for explanatory series or variables (also commonly referred to as independent, input, predictor, regressor, or exogenous variables), which, in our case are either rainfall, minimum or maximum temperatures, their lagged values or moving averages. The VARMAX (*p,q,s*) also allows for cointegration (Engle and Granger 1987; Johansen 1995) among the multiple response series or variables. Informally, cointegration means that each response series (i.e., each of the multiple response series) might be a nonstationary process. Such a process displays no tendency to return to a mean or deterministic trend function in the long

term if perturbed by an impulse or a shock (e.g., a severe drought). However, one or more linear combinations of the response series (multiple response series) are stationary and thus remain near some constant. A linear combination is simply the sum of the different component response series each multiplied by some appropriately chosen constant.

The VARMARX (*p,q,s*) model we used for forecasting can accommodate the following features. 1) Modelling of several time series simultaneously (vector). 2) Accounting for relationships among the individual component series with current and past values of the other series (X). 3) Feedback from the response series and cross-correlated explanatory series. 4) Cointegration of component series to achieve stationarity. 5) Autoregressive errors (of order *p*). 6) Moving average errors (of order *q*). 7) Distributed lags in the explanatory variables (of order *s*). 8) Seasonality, 9) Mixed autoregressive and moving average errors. 10) Lagged values of the explanatory series. 11) Unequal or heteroscedastic covariances for the residuals using various generalized autoregressive conditional heteroschedasticity (GARCH) models (Engle 2002). 12) The modelling framework we used also allows for testing the dependence of one response series on another (testing for weak exogeneity). 13) Moreover, under certain special conditions, the model allows for testing for Granger causality between two specified groups of variables. For example, the Granger causality test may be used by defining one group consisting of the response series and the other of the climatic predictor series. 14) Lastly, the modelling framework allows for putting various restrictions on the estimated parameter coefficients of the model or their linear combinations and testing hypotheses on linear combinations of the parameter coefficients.

We included distributed lags in rainfall, minimum and maximum temperatures (predictor variables) in the VARMAX models and then used univariate autoregressive moving average models to forecast malaria and anemia cases. Separate forecasts were produced for each hospital, disease and age class of patients. Various lags in rainfall, minimum and maximum temperatures were tested before inclusion in the final autoregressive and moving-average multiple regression models with distributed lags used for forecasting. The significance of seasonal deterministic terms for monthly time series data were similarly tested. Dead-start models that do not allow for present (current) values of the predictor variables were tested for heteroscedasticity in residuals by allowing for GARCH-type (generalized autoregressive conditional heteroscedasticity) conditional heteroscedasticity of residuals. The Akaike information criterion (AIC), corrected AIC (AICC), Hannan-Quinn (HQ) criterion, Schwarz Bayesian criterion (SBC) or Bayesian information criterion (BIC) and the final prediction error (FPE) were all used to determine the AR (autoregressive) and MA (moving average) orders of the forecasting models. Partial cross-correlations for the response variables, Yule-Walker estimates, partial autoregressive coefficients and partial canonical correlations were used to further aid AR order identification. S2 Table summarizes details of the full VARMAX (p, q,s) models used for forecasting.

Parameters of the final models were estimated using the maximum likelihood (ML) method. Roots of the characteristic functions for the AR and MA components (eigenvalues) were

evaluated to quantify their proximity to the unit circle to assess evidence for stationarity of the AR process and inevitability of the MA process in the response series (S3 Table). The following diagnostic tools were used to assess the adequacy of the selected models. 1) Durbin-Watson (DW) test for first-order autocorrelation in the residuals (S4 Table). 2) Jarque-Bera normality test for establishing if the model residuals represent a white noise process by testing the null hypothesis that the residuals are normally distributed (S4 Table). 3) F tests for autoregressive conditional heteroscedastic (ARCH) disturbances in the residuals, which test the null hypothesis that the residuals have equal covariances (S4 Table). 4) F tests for AR disturbance computed from the residuals of the univariate AR(1), AR(1,2), AR(1,2,3) and AR(1,2,3,4) models testing the null hypothesis that the residuals are uncorrelated (S5 Table). 5) Portmanteau test for cross correlations of residuals at specified lags (S6 Table). Univariate model ANOVA diagnostics were used to assess if the selected model for malaria or anemia cases is significant and estimate the coefficient of determination r^2 (S7 Table). Parameter estimates for the final malaria and anemia models used for forecasting are provided in S8 Table. Final forecasts and the associated 95% confidence limits were then generated for the time series of malaria or anemia cases for lead times ending in 2100 (S1-S3 Datas).

The selected order of autoregression $p = 1$ or 2 and moving average errors $q = 1$ or 2 for the VARMAX (p, q,s) are provided in Table S2. The number of lags s varied from 2 to 7 depending on the rainfall, minimum or maximum temperature components (Table S2, 5th column). The VARMAX (p,q,s) model used to forecast the future dynamics of malaria and anemia cases is given by:

$$C_t = \sum_{j=1}^{p} \Phi_j C_{t-j} + \sum_{j=0}^{s} \Omega_j^* \boldsymbol{x}_{t-j} + \boldsymbol{\epsilon}_t - \sum_{j=1}^{q} \Omega_j \boldsymbol{\epsilon}_{t-j} \qquad (1)$$

where C_t is the response variable (Table S2, 4th column) at time t, $\boldsymbol{x}_t$ is a vector of rainfall, minimum and maximum temperatures, each lagged appropriately. $\boldsymbol{\epsilon}_t$ is the vector of the white noise process in C_t. $E(\boldsymbol{\epsilon}_t) = 0$, $E(\boldsymbol{\epsilon}_t \boldsymbol{\epsilon}_t^T) = \boldsymbol{\Sigma}$ and $E(\boldsymbol{\epsilon}_t \boldsymbol{\epsilon}_k^T) = 0$ for $t \neq k$. Model (1) was used to project the likely future trajectories of malaria and anemia cases using the projected rainfall, minimum and maximum temperatures under each of the three climate change scenarios (RCP 2.6, 4.5 and 8.5).

The historical and forecasted malaria and anemia cases, the associated residuals, standard errors, pointwise lower and upper 95% confidence limits under the RCPs 2.6, 4.5 and 8.5 emission scenarios are provided in S1-S3 Datas. Finally, we used graphical plots to visualize all the estimated regression relationships and forecasts.

References

Burnham KP, Anderson D., 2002. Model selection and multi-model inference: a practical information-theoretic approach. New York, New York, USA: Springer, Inc.

Engle, R.F., Granger, C.W.J., 1987. Co-integration and error correction: representation, estimation, and testing. Econometrica 55, 251–276.

Engle, R.F., 2002. Dynamic conditional correlation: A simple class of multivariate generalized autoregressive conditional heteroskedasticity models. Journal of Business and Economic Statistics 20.

Hosking, J.R.M., 1980. The multivariate portmanteau statistic. Journal of the American Statistical Association 75, 602–608.

Johansen, S., 1995. Likelihood-based inference in cointegrated vector autoregressive models. Oxford University Press, New York.

SI **2**

Hospital	Total monthly rainfall	Minimum temperature	Maximum temperature
Central Unilever Tea Hospital, Kericho, Kenya	122.166	11.4933	25.4844
Litein Missionary Hospital, Kericho, Kenya	126.214	12.2522	26.7389
Mukumu Hospital, Kakamega, Kenya	148.931	12.8076	27.4828
Muleba Hospital, Tanzania	109.856	17.0708	27.806

SI 3

Table S2: The historical period covered by the data for each hospital, projection periods, response variables and their transformations and predictor variables included in the model VARMAX(p , q , s).

Hospital, Town, Country	Historical Period	Projection periods	Response and transformation	Predictors included in model	Interval	p	q
Central Unilever Tea Hospital, Kericho, Kenya	01APR1995-28FEB2006	2008-2030, 2031-2050, 2051-2070, 2071-2100	Malaria cases for all ages LN(Cases+1/Mean=106.11450382)	Rain5_6 max2 min2	Month	2	1
Litein Missionary, Hospital Kericho, Kenya	01JAN1999-31DEC2005	2008-2030, 2031-2050, 2051-2070, 2071-2100	Malaria cases for all ages LN(Cases+1/Mean=590.73809524)	Rain4_6 mavmax3 mavmin2	Month	2	2
Mukumu Hospital, Kakamega, Kenya	01JAN1995-01JAN2007	2008-2030, 2031-2050, 2051-2070, 2071-2100	Malaria cases for all ages LN(Cases+1/Mean=487.46206897)	Rain4_6 mavmax3 mavmin2	Month	2	2
Muleba Hospital, Muleba, Tanzania	1996-2002	2003-2030, 2031-2050, 2051-2070, 2071-2100	Anaemia cases for under 5 years LN(Cases)	mavrain7 max max3 min2	Month	1	1
Muleba Hospital, Muleba, Tanzania	1996-2002	2003-2030, 2031-2050, 2051-2070, 2071-2100	Malaria cases for under 5 years LN(Cases)	mavrain7 mavmax3 mavmin2	Month	2	2
Muleba Hospital, Muleba, Tanzania	1996-2002	2003-2030, 2031-2050, 2051-2070, 2071-2100	Malaria cases for 5 years and above LN(Cases)	mavrain7 mavmax3 mavmin2	Month	2	2
Muleba Hospital, Tanzania	1996-2002	2003-2030, 2031-2050, 2051-2070, 2071-2100	Malaria cases, All ages LN(Cases)	mavrain7 mavmax3 mavmin2	Month	2	2
All Hospitals, Uganda	1997-2010	2011-2030, 2031-2050, 2051-2070, 2071-2100	Malaria cases for under 5 years LN(Cases/Total admissions)	mavannualrain2 mavmax3 mavmin2	Annual	2	2
All Hospitals, Uganda	1997-2010	2011-2030, 2031-2050, 2051-2070, 2071-2100	Malaria cases for 5 years and above LN(Cases/Total admissions)	mavannualrain2 mavmax3 mavmin2	Annual	2	2

SI **4**

Country	Hospital	Disease	Age	Root	Index	Real Part	Imaginary P	Modulus	Arctangent	Degree
Kenya	Central Unilever Tea Hospital, Kericho	Malaria	All ages	AR	1	0.72514	0	0.7251	0	0
Kenya	Central Unilever Tea Hospital, Kericho	Malaria	All ages	AR	2	-0.85715	0	0.8571	3.1416	180
Kenya	Litein Missionary Hospital, Kericho	Malaria	All ages	AR	1	0.93863	0	0.9386	0	0
Kenya	Litein Missionary Hospital, Kericho	Malaria	All ages	AR	2	-0.59096	0	0.591	3.1416	180
Kenya	Mukumu Hospital, Kakamega	Malaria	All ages	AR	1	0.76626	0	0.7663	0	0
Kenya	Mukumu Hospital, Kakamega	Malaria	All ages	AR	2	-0.37932	0	0.3793	3.1416	180
Tanzania	Muleba Hospital	Anaemia	Under 5 years	AR	1	-0.3111	0	0.3111	3.1416	180
Tanzania	Muleba Hospital	Malaria	All ages	AR	1	-0.24236	0	0.2424	3.1416	180
Tanzania	Muleba Hospital	Malaria	All ages	AR	2	-0.77579	0	0.7758	3.1416	180
Tanzania	Muleba Hospital	Malaria	Five years and above	AR	1	0.06916	0	0.0692	0	0
Tanzania	Muleba Hospital	Malaria	Five years and above	AR	2	-0.978	0	0.978	3.1416	180
Tanzania	Muleba Hospital	Malaria	Under 5 years	AR	1	-0.21978	0	0.2198	3.1416	180
Tanzania	Muleba Hospital	Malaria	Under 5 years	AR	2	-0.64363	0	0.6436	3.1416	180
Uganda	Uganda, entire country	Malaria	Five years and above	AR	1	0.27421	0.69474	0.7469	1.1949	68.4609
Uganda	Uganda, entire country	Malaria	Five years and above	AR	2	0.27421	-0.69474	0.7469	-1.1949	-68.4609
Uganda	Uganda, entire country	Malaria	Under five years	AR	1	0.22486	0.82438	0.8545	1.3045	74.743
Uganda	Uganda, entire country	Malaria	Under five years	AR	2	0.22486	-0.82438	0.8545	-1.3045	-74.743
Kenya	Central Unilever Tea Hospital, Kericho	Malaria	All ages	MA	1	-1	0	1	3.1416	180
Kenya	Litein Missionary Hospital, Kericho	Malaria	All ages	MA	1	1	0	1	0	0
Kenya	Litein Missionary Hospital, Kericho	Malaria	All ages	MA	2	-0.90415	0	0.9042	3.1416	180
Kenya	Mukumu Hospital, Kakamega	Malaria	All ages	MA	1	-0.23599	0.41055	0.4735	2.0925	119.8905
Kenya	Mukumu Hospital, Kakamega	Malaria	All ages	MA	2	-0.23599	-0.41055	0.4735	-2.0925	-119.8905
Tanzania	Muleba Hospital	Anaemia	Under 5 years	MA	1	-0.82341	0	0.8234	3.1416	180
Tanzania	Muleba Hospital	Malaria	All ages	MA	1	-0.89383	0.19823	0.9156	2.9234	167.4958
Tanzania	Muleba Hospital	Malaria	All ages	MA	2	-0.89383	-0.19823	0.9156	-2.9234	-167.4958
Tanzania	Muleba Hospital	Malaria	Five years and above	MA	1	-0.59167	0	0.5917	3.1416	180
Tanzania	Muleba Hospital	Malaria	Five years and above	MA	2	-1	0	1	3.1416	180
Tanzania	Muleba Hospital	Malaria	Under 5 years	MA	1	-0.45443	0	0.4544	3.1416	180
Tanzania	Muleba Hospital	Malaria	Under 5 years	MA	2	-1	0	1	3.1416	180
Uganda	Uganda, entire country	Malaria	Five years and above	MA	1	0.08784	0.99613	1	1.4828	84.9607
Uganda	Uganda, entire country	Malaria	Five years and above	MA	2	0.08784	-0.99613	1	-1.4828	-84.9607
Uganda	Uganda, entire country	Malaria	Under five years	MA	1	0.15554	0.98783	1	1.4146	81.0519
Uganda	Uganda, entire country	Malaria	Under five years	MA	2	0.15554	-0.98783	1	-1.4146	-81.0519

SI **5**

Country	Hospital	Disease	Age	Response	Durbin-Watson Statistics	Jarque-Bera normality test		ARCH(1) test	
						Chi-Square	Pr > ChiSq	F Value	Pr > F
Kenya	Central Unilever Tea Hospital, Kericho	Malaria	All ages	LN(Cases+1/Mean)	1.97458	21.04	<.0001	5.73	0.0182
Kenya	Litein Missionary Hospital, Kericho	Malaria	All ages	LN(Cases+1/Mean)	1.89234	13.42	0.0012	28.37	<0.0001
Kenya	Mukumu Hospital, Kakamega	Malaria	All ages	LN(Cases+1/Mean)	1.98313	511.15	<.0001	0.08	0.7845
Tanzania	MULEBA HOSPITAL	Anaemia	Under 5 years	LN(Cases)	1.88417	60.6	<.0001	0.11	0.7413
Tanzania	MULEBA HOSPITAL	Malaria	All ages	LN(Cases)	2.04576	3.86	0.1451	6.88	0.0106
Tanzania	MULEBA HOSPITAL	Malaria	Five years and above	LN(Cases)	1.81909	1.48	0.4771	0	0.9499
Tanzania	MULEBA HOSPITAL	Malaria	Under 5 years	LN(Cases)	1.94434	3.07	0.2155	9.24	0.0033
Uganda	Uganda	Malaria	Five years and above	LN(Cases/Total admissions)	2.24631	0.24	0.8883	3.58	0.0911
Uganda	Uganda	Malaria	Under five years	LN(Cases/Total admissions)	2.34006	0.31	0.8583	0.15	0.7092

SI **6**

Country	Hospital	Disease	Age	Response	AR(1) F Value	AR(1) Pr > F	AR(1,2) F Value	AR(1,2) Pr > F	AR(1,2,3) F Value	AR(1,2,3) Pr > F	AR(1,2,34) F Value	AR(1,2,34) Pr > F
Kenya	Central Unilever Tea Hospital, Kericho	Malaria	All ages	log_cases2	0.01	0.9028	0.01	0.9929	0.54	0.6537	0.42	0.7967
Kenya	Litein Missionary Hospital, Kericho	Malaria	All ages	log_cases2	0.22	0.6415	0.16	0.8558	1.74	0.1661	1.83	0.1317
Kenya	Mukumu Hospital, Kakamega	Malaria	All ages	log_cases2	0	0.9585	0.02	0.9764	0.08	0.9729	0.05	0.9944
Tanzania	MULEBA HOSPITAL	Anaemia	Under 5 years	logP	0.53	0.4689	0.54	0.5879	1.14	0.3406	1.07	0.3796
Tanzania	MULEBA HOSPITAL	Malaria	All ages	logP	0.09	0.7644	0.56	0.5722	0.38	0.7694	0.41	0.8038
Tanzania	MULEBA HOSPITAL	Malaria	Five years and above	logP	0.39	0.5323	1.27	0.2863	0.86	0.4652	0.73	0.5777
Tanzania	MULEBA HOSPITAL	Malaria	Under 5 years	logP	0.04	0.8512	0.18	0.8391	0.12	0.9453	0.3	0.8799
Uganda	Uganda, entire country	Malaria	Five years and above	logtotal	0.14	0.7129	0.37	0.7055	0.22	0.8781	0.23	0.9032
Uganda	Uganda, entire country	Malaria	Under five years	logtotal	0.3	0.5943	1.16	0.3661	0.79	0.5512	3.17	0.1851

SI **7**

Country	Hospital	Disease	Age	Up To Lag	Degrees of Freedom	Chi-Square	Pr > ChiSq
Kenya	Central Unilever Tea Hospital, Kericho	Malaria	All ages	4	1	1.77	0.1836
Kenya	Central Unilever Tea Hospital, Kericho	Malaria	All ages	5	2	2.29	0.3187
Kenya	Central Unilever Tea Hospital, Kericho	Malaria	All ages	6	3	5.01	0.1707
Kenya	Central Unilever Tea Hospital, Kericho	Malaria	All ages	7	4	7.96	0.0931
Kenya	Central Unilever Tea Hospital, Kericho	Malaria	All ages	8	5	10.23	0.0689
Kenya	Central Unilever Tea Hospital, Kericho	Malaria	All ages	9	6	20.04	0.0027
Kenya	Central Unilever Tea Hospital, Kericho	Malaria	All ages	10	7	21.43	0.0032
Kenya	Central Unilever Tea Hospital, Kericho	Malaria	All ages	11	8	21.43	0.0061
Kenya	Central Unilever Tea Hospital, Kericho	Malaria	All ages	12	9	21.51	0.0106
Kenya	Litein Missionary Hospital, Kericho	Malaria	All ages	5	1	6.53	0.0106
Kenya	Litein Missionary Hospital, Kericho	Malaria	All ages	6	2	10.23	0.006
Kenya	Litein Missionary Hospital, Kericho	Malaria	All ages	7	3	10.36	0.0157
Kenya	Litein Missionary Hospital, Kericho	Malaria	All ages	8	4	10.4	0.0342
Kenya	Litein Missionary Hospital, Kericho	Malaria	All ages	9	5	10.61	0.0597
Kenya	Litein Missionary Hospital, Kericho	Malaria	All ages	10	6	11.03	0.0873
Kenya	Litein Missionary Hospital, Kericho	Malaria	All ages	11	7	11.12	0.1334
Kenya	Litein Missionary Hospital, Kericho	Malaria	All ages	12	8	12.13	0.1457
Kenya	Mukumu Hospital, Kakamega	Malaria	All ages	5	1	0.31	0.5762
Kenya	Mukumu Hospital, Kakamega	Malaria	All ages	6	2	3.13	0.2095
Kenya	Mukumu Hospital, Kakamega	Malaria	All ages	7	3	3.18	0.365
Kenya	Mukumu Hospital, Kakamega	Malaria	All ages	8	4	7.81	0.0988
Kenya	Mukumu Hospital, Kakamega	Malaria	All ages	9	5	8.36	0.1375
Kenya	Mukumu Hospital, Kakamega	Malaria	All ages	10	6	8.46	0.2062
Kenya	Mukumu Hospital, Kakamega	Malaria	All ages	11	7	11.96	0.1018
Kenya	Mukumu Hospital, Kakamega	Malaria	All ages	12	8	13.97	0.0826
Tanzania	Muleba Hospital	Anaemia	Under 5 years	3	1	2.96	0.0856
Tanzania	Muleba Hospital	Anaemia	Under 5 years	4	2	3.39	0.1835
Tanzania	Muleba Hospital	Anaemia	Under 5 years	5	3	4.23	0.2375
Tanzania	Muleba Hospital	Anaemia	Under 5 years	6	4	13.86	0.0077
Tanzania	Muleba Hospital	Anaemia	Under 5 years	7	5	13.96	0.0159

Tanzania	Muleba Hospital	Anaemia	Under 5 years	8	6	14.63	0.0233
Tanzania	Muleba Hospital	Anaemia	Under 5 years	9	7	14.94	0.0368
Tanzania	Muleba Hospital	Anaemia	Under 5 years	10	8	16.54	0.0353
Tanzania	Muleba Hospital	Anaemia	Under 5 years	11	9	16.6	0.0553
Tanzania	Muleba Hospital	Anaemia	Under 5 years	12	10	26.62	0.003
Tanzania	Muleba Hospital	Malaria	All ages	5	1	2.63	0.105
Tanzania	Muleba Hospital	Malaria	All ages	6	2	2.93	0.2314
Tanzania	Muleba Hospital	Malaria	All ages	7	3	3.96	0.2662
Tanzania	Muleba Hospital	Malaria	All ages	8	4	7.94	0.0937
Tanzania	Muleba Hospital	Malaria	All ages	9	5	10.21	0.0694
Tanzania	Muleba Hospital	Malaria	All ages	10	6	10.98	0.089
Tanzania	Muleba Hospital	Malaria	All ages	11	7	13.16	0.0684
Tanzania	Muleba Hospital	Malaria	All ages	12	8	19.85	0.0109
Tanzania	Muleba Hospital	Malaria	Five years and above	5	1	8.92	0.0028
Tanzania	Muleba Hospital	Malaria	Five years and above	6	2	12.97	0.0015
Tanzania	Muleba Hospital	Malaria	Five years and above	7	3	22.43	<0.0001
Tanzania	Muleba Hospital	Malaria	Five years and above	8	4	22.75	0.0001
Tanzania	Muleba Hospital	Malaria	Five years and above	9	5	23.14	0.0003
Tanzania	Muleba Hospital	Malaria	Five years and above	10	6	24.24	0.0005
Tanzania	Muleba Hospital	Malaria	Five years and above	11	7	26.08	0.0005
Tanzania	Muleba Hospital	Malaria	Five years and above	12	8	30.95	0.0001
Tanzania	Muleba Hospital	Malaria	Under 5 years	5	1	1.34	0.2474
Tanzania	Muleba Hospital	Malaria	Under 5 years	6	2	1.86	0.3943
Tanzania	Muleba Hospital	Malaria	Under 5 years	7	3	1.95	0.5833
Tanzania	Muleba Hospital	Malaria	Under 5 years	8	4	2.97	0.5637
Tanzania	Muleba Hospital	Malaria	Under 5 years	9	5	2.97	0.7051
Tanzania	Muleba Hospital	Malaria	Under 5 years	10	6	3.1	0.796
Tanzania	Muleba Hospital	Malaria	Under 5 years	11	7	5.16	0.641
Tanzania	Muleba Hospital	Malaria	Under 5 years	12	8	10.53	0.2299
Uganda	Uganda, entire country	Malaria	Five years and above	5	1	1.83	0.1761
Uganda	Uganda, entire country	Malaria	Five years and above	6	2	1.98	0.3718
Uganda	Uganda, entire country	Malaria	Five years and above	7	3	1.99	0.5739
Uganda	Uganda, entire country	Malaria	Five years and above	8	4	3.57	0.4673

Uganda	Uganda, entire country	Malaria	Five years and above	9	5	3.63	0.6038
Uganda	Uganda, entire country	Malaria	Five years and above	10	6	3.64	0.7257
Uganda	Uganda, entire country	Malaria	Five years and above	11	7	3.64	0.8205
Uganda	Uganda, entire country	Malaria	Under five years	5	1	6.75	0.0094
Uganda	Uganda, entire country	Malaria	Under five years	6	2	7.44	0.0242
Uganda	Uganda, entire country	Malaria	Under five years	7	3	7.47	0.0583
Uganda	Uganda, entire country	Malaria	Under five years	8	4	7.5	0.1118
Uganda	Uganda, entire country	Malaria	Under five years	9	5	7.59	0.1803
Uganda	Uganda, entire country	Malaria	Under five years	10	6	7.62	0.2674
Uganda	Uganda, entire country	Malaria	Under five years	11	7	7.62	0.3669

SI **8**

Country	Hospital	Disease	Age	Response	R-Square	Standard Deviation	F Value	Pr > F
Kenya	Central Unilever Tea Hospital, Kericho	Malaria	All ages	log_cases2	0.6104	0.24853	31.86	<0.0001
Kenya	Litein Missionary Hospital, Kericho	Malaria	All ages	log_cases2	0.5151	0.1044	11.23	<0.0001
Kenya	Mukumu Hospital, Kakamega	Malaria	All ages	log_cases2	0.7606	0.1521	61.28	<0.0001
Tanzania	MULEBA HOSPITAL	Anaemia	Under 5 years	logP	0.4319	0.48429	8.11	<0.0001
Tanzania	MULEBA HOSPITAL	Malaria	All ages	logP	0.5462	0.15822	11.86	<0.0001
Tanzania	MULEBA HOSPITAL	Malaria	Five years and above	logP	0.5602	0.24356	12.56	<0.0001
Tanzania	MULEBA HOSPITAL	Malaria	Under 5 years	logP	0.4281	0.14121	7.38	<0.0001
Uganda	Uganda, entire country	Malaria	Five years and above	logtotal	0.6724	0.11509	1.17	0.4649
Uganda	Uganda, entire country	Malaria	Under five years	logtotal	0.7428	0.09302	1.65	0.3288

SI **9**

Country	Hospital	Disease	All Ages	Response	Predictor Variable	Parameter	Estimate	Standard Error	t Value	Pr > |t|
Kenya	Central Unilever Tea Hospital, Kericho	Malaria	All Ages	log_cases2	1	CONST1	-0.02145	0.14481	-0.15	0.8825
Kenya	Central Unilever Tea Hospital, Kericho	Malaria	All Ages	log_cases2	rain5_6(t)	XL0_1_1	0.31768	0.09735	3.26	0.0014
Kenya	Central Unilever Tea Hospital, Kericho	Malaria	All Ages	log_cases2	max2(t)	XL0_1_2	0.45515	0.21789	2.09	0.0387
Kenya	Central Unilever Tea Hospital, Kericho	Malaria	All Ages	log_cases2	min2(t)	XL0_1_3	0.00196	0.09921	0.02	0.9843
Kenya	Central Unilever Tea Hospital, Kericho	Malaria	All Ages	log_cases2	log_cases2(t-1)	AR1_1_1	-0.13201	0.06933	-1.9	0.0591
Kenya	Central Unilever Tea Hospital, Kericho	Malaria	All Ages	log_cases2	log_cases2(t-2)	AR2_1_1	0.62155	0.06865	9.05	0.0001
Kenya	Central Unilever Tea Hospital, Kericho	Malaria	All Ages	log_cases2	e1(t-1)	MA1_1_1	-1	0.02791	-35.83	0.0001
Kenya	Litein Missionary Hospital, Kericho	Malaria	All Ages	log_cases2	1	CONST1	0.26651	0.10814	2.46	0.0158
Kenya	Litein Missionary Hospital, Kericho	Malaria	All Ages	log_cases2	rain4_6(t)	XL0_1_1	0.1079	0.0315	3.42	0.001
Kenya	Litein Missionary Hospital, Kericho	Malaria	All Ages	log_cases2	Mavmax3(t)	XL0_1_2	0.58994	0.75788	0.78	0.4386
Kenya	Litein Missionary Hospital, Kericho	Malaria	All Ages	log_cases2	Mavmin2(t)	XL0_1_3	-0.33025	0.10798	-3.06	0.003
Kenya	Litein Missionary Hospital, Kericho	Malaria	All Ages	log_cases2	log_cases2(t-1)	AR1_1_1	0.34767	0.10371	3.35	0.0012
Kenya	Litein Missionary Hospital, Kericho	Malaria	All Ages	log_cases2	log_cases2(t-2)	AR2_1_1	0.55469	0.11569	4.79	0.0001
Kenya	Litein Missionary Hospital, Kericho	Malaria	All Ages	log_cases2	e1(t-1)	MA1_1_1	0.09585	0.09983	0.96	0.3398
Kenya	Litein Missionary Hospital, Kericho	Malaria	All Ages	log_cases2	e1(t-2)	MA2_1_1	0.90415	0.09927	9.11	0.0001
Kenya	Mukumu Hospital, Kakamega	Malaria	All Ages	log_cases2	1	CONST1	-0.31719	0.32753	-0.97	0.3345
Kenya	Mukumu Hospital, Kakamega	Malaria	All Ages	log_cases2	rain4_6(t)	XL0_1_1	0.25572	0.06724	3.8	0.0002
Kenya	Mukumu Hospital, Kakamega	Malaria	All Ages	log_cases2	Mavmax3(t)	XL0_1_2	-3.28699	1.64777	-1.99	0.048
Kenya	Mukumu Hospital, Kakamega	Malaria	All Ages	log_cases2	Mavmin2(t)	XL0_1_3	0.38364	0.30633	1.25	0.2125
Kenya	Mukumu Hospital, Kakamega	Malaria	All Ages	log_cases2	log_cases2(t-1)	AR1_1_1	0.38694	0.20118	1.92	0.0564
Kenya	Mukumu Hospital, Kakamega	Malaria	All Ages	log_cases2	log_cases2(t-2)	AR2_1_1	0.29066	0.18805	1.55	0.1244
Kenya	Mukumu Hospital, Kakamega	Malaria	All Ages	log_cases2	e1(t-1)	MA1_1_1	-0.47197	0.20627	-2.29	0.0236
Kenya	Mukumu Hospital, Kakamega	Malaria	All Ages	log_cases2	e1(t-2)	MA2_1_1	-0.22424	0.1078	-2.08	0.0393
Tanzania	Muleba Hospital	Anaemia	Under 5 years	logP	1	CONST1	2.35352	1.03276	2.28	0.0257
Tanzania	Muleba Hospital	Anaemia	Under 5 years	logP	Mavrain7(t)	XL0_1_1	2.80014	0.50611	5.53	0.0001
Tanzania	Muleba Hospital	Anaemia	Under 5 years	logP	max(t)	XL0_1_2	7.16067	2.78101	2.57	0.0121
Tanzania	Muleba Hospital	Anaemia	Under 5 years	logP	max3(t)	XL0_1_3	4.62806	1.63937	2.82	0.0062
Tanzania	Muleba Hospital	Anaemia	Under 5 years	logP	min2(t)	XL0_1_4	-2.08371	1.0383	-2.01	0.0486
Tanzania	Muleba Hospital	Anaemia	Under 5 years	logP	logP(t-1)	AR1_1_1	-0.3111	0.11298	-2.75	0.0075
Tanzania	Muleba Hospital	Anaemia	Under 5 years	logP	e1(t-1)	MA1_1_1	-0.82341	0.10022	-8.22	0.0001
Tanzania	Muleba Hospital	Malaria	All ages	logP	1	CONST1	7.09902	1.49103	4.76	0.0001

Tanzania	Muleba Hospital	Malaria	All ages	logP	Mavrain7(t)	XL0_1_1	0.60312	0.10075	5.99	0.0001
Tanzania	Muleba Hospital	Malaria	All ages	logP	Mavmax3(t)	XL0_1_2	-4.14528	1.25292	-3.31	0.0014
Tanzania	Muleba Hospital	Malaria	All ages	logP	Mavmin2(t)	XL0_1_3	1.38371	0.82015	1.69	0.0956
Tanzania	Muleba Hospital	Malaria	All ages	logP	logP(t-1)	AR1_1_1	-1.01815	0.09279	-10.97	0.0001
Tanzania	Muleba Hospital	Malaria	All ages	logP	logP(t-2)	AR2_1_1	-0.18802	0.09641	-1.95	0.0548
Tanzania	Muleba Hospital	Malaria	All ages	logP	e1(t-1)	MA1_1_1	-1.78767	0.12594	-14.19	0.0001
Tanzania	Muleba Hospital	Malaria	All ages	logP	e1(t-2)	MA2_1_1	-0.83823	0.12752	-6.57	0.0001
Tanzania	Muleba Hospital	Malaria	Five years and above	logP	1	CONST1	3.92789	1.85663	2.12	0.0376
Tanzania	Muleba Hospital	Malaria	Five years and above	logP	Mavrain7(t)	XL0_1_1	1.00917	0.19529	5.17	0.0001
Tanzania	Muleba Hospital	Malaria	Five years and above	logP	Mavmax3(t)	XL0_1_2	-10.8609	2.07415	-5.24	0.0001
Tanzania	Muleba Hospital	Malaria	Five years and above	logP	Mavmin2(t)	XL0_1_3	3.73474	1.1891	3.14	0.0024
Tanzania	Muleba Hospital	Malaria	Five years and above	logP	logP(t-1)	AR1_1_1	-0.90884	0.09907	-9.17	0.0001
Tanzania	Muleba Hospital	Malaria	Five years and above	logP	logP(t-2)	AR2_1_1	0.06764	0.0846	0.8	0.4264
Tanzania	Muleba Hospital	Malaria	Five years and above	logP	e1(t-1)	MA1_1_1	-1.59167	0.14302	-11.13	0.0001
Tanzania	Muleba Hospital	Malaria	Five years and above	logP	e1(t-2)	MA2_1_1	-0.59167	0.14208	-4.16	0.0001
Tanzania	Muleba Hospital	Malaria	Under 5 years	logP	1	CONST1	8.09306	1.485	5.45	0.0001
Tanzania	Muleba Hospital	Malaria	Under 5 years	logP	Mavrain7(t)	XL0_1_1	0.39676	0.10708	3.71	0.0004
Tanzania	Muleba Hospital	Malaria	Under 5 years	logP	Mavmax3(t)	XL0_1_2	-1.83791	1.41079	-1.3	0.1965
Tanzania	Muleba Hospital	Malaria	Under 5 years	logP	Mavmin2(t)	XL0_1_3	1.39137	0.69122	2.01	0.0476
Tanzania	Muleba Hospital	Malaria	Under 5 years	logP	logP(t-1)	AR1_1_1	-0.86342	0.13529	-6.38	0.0001
Tanzania	Muleba Hospital	Malaria	Under 5 years	logP	logP(t-2)	AR2_1_1	-0.14146	0.09393	-1.51	0.1361
Tanzania	Muleba Hospital	Malaria	Under 5 years	logP	e1(t-1)	MA1_1_1	-1.45443	0.1696	-8.58	0.0001
Tanzania	Muleba Hospital	Malaria	Under 5 years	logP	e1(t-2)	MA2_1_1	-0.45443	0.16392	-2.77	0.007
Uganda	Uganda, entire country	Malaria	Five years and above	logtotal	1	CONST1	-19.9415	8.12213	-2.46	0.0303
Uganda	Uganda, entire country	Malaria	Five years and above	logtotal	mavannualrain2(t)	XL0_1_1	0.00055	0	.	.
Uganda	Uganda, entire country	Malaria	Five years and above	logtotal	mavmax3(t)	XL0_1_2	0.76308	0.38727	1.97	0.0723
Uganda	Uganda, entire country	Malaria	Five years and above	logtotal	mavmin2(t)	XL0_1_3	-0.21166	0.23778	-0.89	0.3909
Uganda	Uganda, entire country	Malaria	Five years and above	logtotal	logtotal(t-1)	AR1_1_1	0.54843	0.21304	2.57	0.0244
Uganda	Uganda, entire country	Malaria	Five years and above	logtotal	logtotal(t-2)	AR2_1_1	-0.55786	0.23798	-2.34	0.0371
Uganda	Uganda, entire country	Malaria	Five years and above	logtotal	e1(t-1)	MA1_1_1	0.17568	0.31565	0.56	0.5881
Uganda	Uganda, entire country	Malaria	Five years and above	logtotal	e1(t-2)	MA2_1_1	-1	0.29609	-3.38	0.0055
Uganda	Uganda, entire country	Malaria	Under five years	logtotal	1	CONST1	-13.6823	7.36143	-1.86	0.0878
Uganda	Uganda, entire country	Malaria	Under five years	logtotal	mavannualrain2(t)	XL0_1_1	0.00056	0	.	.

Uganda	Uganda, entire country	Malaria	Under five years	logtotal	mavmax3(t)	XL0_1_2	0.52289	0.29987	1.74	0.1067
Uganda	Uganda, entire country	Malaria	Under five years	logtotal	mavmin2(t)	XL0_1_3	-0.21436	0.14831	-1.45	0.174
Uganda	Uganda, entire country	Malaria	Under five years	logtotal	logtotal(t-1)	AR1_1_1	0.44972	0.2176	2.07	0.061
Uganda	Uganda, entire country	Malaria	Under five years	logtotal	logtotal(t-2)	AR2_1_1	-0.73017	0.16115	-4.53	0.0007
Uganda	Uganda, entire country	Malaria	Under five years	logtotal	e1(t-1)	MA1_1_1	0.31108	0.28743	1.08	0.3004
Uganda	Uganda, entire country	Malaria	Under five years	logtotal	e1(t-2)	MA2_1_1	-1	0.21264	-4.7	0.0005

SI 10

```
/*-----SAS codes (Version 9.4) for fitting the VARMAX (Version 15.1) models to Malaria data for Kenya--*/

ods graphics on;
proc varmax data=Malaria7 plots=all;
by  GCM Hospital Scenario;
where Hospital ="Central Unilever Tea Hospital, Kericho, Kenya";
id month3 interval=month align=end;
nloptions tech=newrap maxit=1000 pall;
ods output DiagnostAR=DiagnostAR1 DiagnostWN=DiagnostWN1 ANOVA=ANOVA1 MARoots=MARoots1 ARRoots=ARRoots1
PortmanteauTest=PortmanteauTest1;

  model log_cases2

= Rain5_6  max2 min2/p=2 q=1   /*xlag=(4,5,6)*/  /*nocurrentx*/ /*nseason=12*/
                  printform=univariate
                  print=(corry corrx pcorr pcancorr parcoef Estimates roots diagnose) method=ml minic=(type=aic p=4);
*GARCH q=1  form=BEKK subform=garch OUTHT=Malaria_Garch;
causal group1=(Rain5_6  max2 min2) group2=(log_cases2);
ods output corrxgraph=corrxgraph corrygraph=corrygraph CorrXLags=CorrXLags
CorrXbyVar=CorrXbyVar CorrYLags=CorrYLags CorrYbyVar=CorrYbyVar
PartialAR=PartialAR ParameterEstimates=ParameterEstimates1 ParameterGraph=ParameterGraph
PartialAR=PartialAR PartialARGraph=PartialARGraph PartialCanCorr=PartialCanCorr
PartialCorr=PartialCorr PartialCorrbyVar=PartialCorrbyVar PartialCorrGraph=PartialCorrGraph;
output out=pred_Malaria1 lead=1138 back=0;

run;
ods graphics off;
```

```
ods graphics on;
proc varmax data=Malaria7 plots=all;
by  GCM Hospital Scenario;
where Hospital in ('Litein Missionary Hospital, Kericho, Kenya');
id month3 interval=month align=end;
nloptions tech=newrap maxit=1000 pall;
ods output DiagnostAR=DiagnostAR2 DiagnostWN=DiagnostWN2 ANOVA=ANOVA2 MARoots=MARoots2 ARRoots=ARRoots2
PortmanteauTest=PortmanteauTest2;

  model log_cases2

= Rain4_6  mavmax3 mavmin2/p=2 q=2  /*xlag=(4,5,6)*/  /*nocurrentx*/ /*nseason=12*/
                   printform=univariate
                   print=(corry corrx pcorr pcancorr parcoef Estimates roots diagnose) method=ml minic=(type=aic p=4);
*GARCH q=1  form=BEKK subform=garch OUTHT=Malaria_Garch2;
causal group1=( Rain4_6  mavmax3 mavmin2) group2=(log_cases2);
ods output corrxgraph=corrxgraph corrygraph=corrygraph CorrXLags=CorrXLags
CorrXbyVar=CorrXbyVar CorrYLags=CorrYLags CorrYbyVar=CorrYbyVar
PartialAR=PartialAR ParameterEstimates=ParameterEstimates2 ParameterGraph=ParameterGraph
PartialAR=PartialAR PartialARGraph=PartialARGraph PartialCanCorr=PartialCanCorr
PartialCorr=PartialCorr PartialCorrbyVar=PartialCorrbyVar PartialCorrGraph=PartialCorrGraph;
output out=pred_Malaria2 lead=1138 back=0;
run;
ods graphics off;


ods graphics on;
proc varmax data=Malaria7 plots=all;
by  GCM Hospital Scenario;
where Hospital in ('Mukumu Hospital, Kakamega, Kenya');
id month3 interval=month align=end;
nloptions tech=newrap maxit=1000 pall;
```

```
ods output DiagnostAR=DiagnostAR3 DiagnostWN=DiagnostWN3 ANOVA=ANOVA3 MARoots=MARoots3 ARRoots=ARRoots3
PortmanteauTest=PortmanteauTest3;

  model log_cases2

= Rain4_6 mavmax3 mavmin2/p=2 q=2  /*xlag=(4,5,6)*/  /*nocurrentx*/ /*nseason=12*/
                printform=univariate
                print=(corry corrx pcorr pcancorr parcoef Estimates roots diagnose) method=ml minic=(type=aic p=4);

ods output corrxgraph=corrxgraph corrygraph=corrygraph CorrXLags=CorrXLags
CorrXbyVar=CorrXbyVar CorrYLags=CorrYLags CorrYbyVar=CorrYbyVar
PartialAR=PartialAR ParameterEstimates=ParameterEstimates3 ParameterGraph=ParameterGraph
PartialAR=PartialAR PartialARGraph=PartialARGraph PartialCanCorr=PartialCanCorr
PartialCorr=PartialCorr PartialCorrbyVar=PartialCorrbyVar PartialCorrGraph=PartialCorrGraph;
output out=pred_Malaria3 lead=1127 back=0;
run;
ods graphics off;

Data Pred_malaria_Kenya;
Length Scenario $10;
set pred_Malaria1-pred_Malaria3;
Month=Month(Month3);
Year=Year (Month3);
Cases=exp(log_cases2)-1;
Pred=exp(FOR1)-1;
if year<=2007               then Period="1995-2007";
if 2016<=year<=2045    then Period="2016-2045";
if 2036<=year<=2065    then Period="2036-2065";
if 2055<=year<=2085    then Period="2055-2085";
if 2071<=year<=2100    then Period="2071-2100";
run;
/*------SAS codes for fitting the historic and projected trends for Kenya----*/
```

```
%let outdir=F:\joseph_2009\CAMCO\Results\Health\scenarios_health;
proc template;
define style myfont;
parent=styles.journal;

style
GraphFonts   /
'GraphDataFont'     = ("Helvetica",12pt)
'GraphUnicodeFont'  = ("Helvetica",16pt)
'GraphValueFont'    = ("Helvetica",13pt)
'GraphLabelFont'    = ("Helvetica",16pt,bold)
'GraphFootnoteFont' = ("Helvetica",6pt,bold)
'GraphTitleFont'    = ("Helvetica",16pt,bold)
'GraphAnnoFont'     = ("Helvetica",6pt);
*style GraphBackground /  backgroundcolor=white;
*style body  /background=white;
class graphbackground /   color=white;
*class graphwalls      /   color=white;
*class graphgridlines  /   contrastcolor=black;
end;
run;

options orientation=portrait nodate nonumber;
goptions reset=all ;
ods results off;
ods listing close;
ods html path="&outdir" (url=none) file='sastest.html'
image_dpi=300  style=myfont;


/*---- Central Unilever Tea Hospital, Kericho, Kenya----*/
ods graphics on /reset=index  imagename='Kenyan_Unilever_Malaria_cases_projections_2022'
```

```
     width=28 cm height=22 cm;
           ods listing image_dpi=300 style=myfont;
proc sgpanel data=Pred_malaria_Kenya2 noautolegend;
by GCM;
where Hospital="Central Unilever Tea Hospital, Kericho, Kenya" ;
panelby  Hospital Scenario/novarname layout=lattice columns=1 rows=3 spacing=1 uniscale=column sort=data;
styleattrs
    datacontrastcolors=(blue red chartreuse)
    datalinepatterns=( solid shortdash MediumDashDotDot);

band    x=Month3 lower=LCI1 upper=UCI1 /fill fillattrs=(color=chartreuse) transparency=0 name="S4" legendlabel="95% Confidence limits";
scatter  x=Month3 y=log_cases2  / markerattrs=(color=mediumslateblue  symbol=circlefilled size=12) transparency=0 dataskin=sheen name="S1" legendlabel="Actual";
Series  x=Month3 y=FOR1 / lineattrs=(color=deeppink Pattern=1 thickness=2) name="S3" legendlabel="Predicted";
Pbspline x=Month3 y=FOR1 / lineattrs=(color=blue Pattern=1 thickness=2) nomarkers nknots=200 maxpoints=150 name="S2" legendlabel="Smoothed";
rowaxis label="LN(Malaria cases/Mean=106.1)" grid;
colaxis offsetmin=0.05  offsetmax=0.05 label="Date" values=( '01APR1995'd to '01JAN2101'd by 254);
title 'Forecasting malaria cases in relation to rainfall and temperature';
refline '31JAN2006'd / axis=x lineattrs=(pattern=4 color=blue)  labelpos=min name="S5" legendlabel="Start of multi-step forecasts (2006)";
refline '31JAN2030'd/ axis=x lineattrs=(color=orangered pattern=4 thickness=1)  label=("2030s")  labelpos=min;
refline '31JAN2050'd / axis=x lineattrs=(color=orangered pattern=4 thickness=1) label=("2050s")  labelpos=min;
refline '31JAN2070'd / axis=x lineattrs=(color=orangered pattern=4 thickness=1) label=("2070s")  labelpos=min;
refline '31JAN2090'd / axis=x lineattrs=(color=orangered pattern=4 thickness=1) label=("2090s")  labelpos=min;
Keylegend "S1" "S2"  "S3"  "S4" "S5" / border position=bottom across=5 down=1 exclude=("RCP2.6"  "RCP4.5" "RCP8.5");
run;
quit;

ods graphics on /reset=index  imagename='Kenyan_Unilever_Malaria_Ensemble_2022'
     width=28 cm height=26 cm;
```

```
       ods listing image_dpi=300;
proc sgpanel data=Pred_malaria_Kenya2 noautolegend;
where (Hospital="Central Unilever Tea Hospital, Kericho, Kenya" );
panelby  Hospital Scenario/novarname layout=lattice columns=1 rows=3 spacing=1 uniscale=column sort=(auto descending);
scatter  x=Month3 y=log_cases2  / markerattrs=(color=mediumslateblue  symbol=circlefilled size=12) transparency=0 dataskin=sheen
name="S1" legendlabel="Actual";
Series  x=Month3 y=FOR1 /group=GCM name="S3" legendlabel="Predicted";
rowaxis label="LN(Malaria cases/Mean=106.1)" grid;
colaxis offsetmin=0.05  offsetmax=0.05 label="Date" values=( '01APR1995'd to '01JAN2101'd by 254);
title 'Forecasting malaria cases in relation to rainfall and temperature';
refline '31JAN2006'd / axis=x lineattrs=(pattern=4 color=blue)  labelpos=min name="S5" legendlabel="Start of multi-step forecasts
(2006)";
refline '31JAN2030'd/ axis=x lineattrs=(color=orangered pattern=4 thickness=1)  label=("2030s")  labelpos=min;
refline '31JAN2050'd / axis=x lineattrs=(color=orangered pattern=4 thickness=1) label=("2050s")  labelpos=min;
refline '31JAN2070'd / axis=x lineattrs=(color=orangered pattern=4 thickness=1) label=("2070s")  labelpos=min;
refline '31JAN2090'd / axis=x lineattrs=(color=orangered pattern=4 thickness=1) label=("2090s")  labelpos=min;
Keylegend "S1"  "S3"  "S5" / border position=bottom exclude=("RCP2.6"  "RCP4.5" "RCP8.5")
autoitemsize;
run;
quit;

/*---- Litein Missionary Hospital, Kericho, Kenya ----*/

ods graphics on /reset=index  imagename='Kenyan_Litein_Malaria__cases_projections_2022'
   width=28 cm height=26 cm;
ods listing image_dpi=300 /*style=myfont*/;
proc sgpanel data=Pred_malaria_Kenya2 noautolegend;
by GCM;
where Hospital="Litein Missionary Hospital, Kericho, Kenya";
panelby  Hospital Scenario /novarname layout=lattice columns=1 rows=3 spacing=1 uniscale=column sort=data /*(auto descending)*/;
band    x=Month3 lower=LCI1 upper=UCI1 /fill fillattrs=(color=lime) transparency=0.3 name="S4" legendlabel="95% Confidence
limits";
```

```
scatter  x=Month3 y=log_cases2  /markerattrs=(color=mediumslateblue  symbol=circlefilled size=12)transparency=0.1 dataskin=sheen
name="S1" legendlabel="Actual";
Series  x=Month3 y=FOR1 /lineattrs=(color=Tomato Pattern=1 thickness=2) name="S3" legendlabel="Predicted";
Pbspline x=Month3 y=FOR1 / lineattrs=(color=blue Pattern=1 thickness=2) nomarkers nknots=200 maxpoints=150 name="S2"
legendlabel="Smoothed";
rowaxis label="LN(Malaria cases/Mean=590.7)" grid;
colaxis offsetmin=0.05  label="Date" values=( '01JAN1999'd to '01DEC2100'd by 204);
title 'Forecasting malaria cases in relation to rainfall and temperature';
refline '28FEB2006'd / axis=x lineattrs=(pattern=4 color=blue)  labelpos=min name="S5" legendlabel="Start of multi-step forecasts
(2006)";
refline '31JAN2030'd / axis=x lineattrs=(color=orangered pattern=4 thickness=1) label=("2030s")  labelpos=min;
refline '31JAN2050'd / axis=x lineattrs=(color=orangered pattern=4 thickness=1) label=("2050s")  labelpos=min;
refline '31JAN2070'd / axis=x lineattrs=(color=orangered pattern=4 thickness=1) label=("2070s")  labelpos=min;
refline '31JAN2090'd / axis=x lineattrs=(color=orangered pattern=4 thickness=1) label=("2090s")  labelpos=min;
Keylegend "S1" "S2" "S3" "S4" "S5"/ border position=bottom across=5 down=1;
run;
quit;
ods graphics on /reset=index  imagename='Kenyan_Litein_Malaria_Ensemble_2022'
    width=28 cm height=26 cm;
ods listing image_dpi=300 ;
proc sgpanel data=Pred_malaria_Kenya2 noautolegend;
by GCM;
where Hospital="Litein Missionary Hospital, Kericho, Kenya";
panelby  Hospital Scenario /novarname layout=lattice columns=1 rows=3 spacing=1 uniscale=column sort=(auto descending);
scatter  x=Month3 y=log_cases2  /markerattrs=(color=mediumslateblue  symbol=circlefilled size=12)transparency=0.1 dataskin=sheen
name="S1" legendlabel="Actual";
Series  x=Month3 y=FOR1 /group=GCM   name="S3" legendlabel="Predicted";
rowaxis label="LN(Malaria cases/Mean=590.7)" grid;
colaxis offsetmin=0.05  label="Date" values=( '01JAN1999'd to '01DEC2100'd by 204);
title 'Forecasting malaria cases in relation to rainfall and temperature';
refline '28FEB2006'd / axis=x lineattrs=(pattern=4 color=blue)  labelpos=min name="S5" legendlabel="Start of multi-step forecasts
(2006)";
```

```
refline '31JAN2030'd / axis=x lineattrs=(color=orangered pattern=4 thickness=1) label=("2030s")  labelpos=min;
refline '31JAN2050'd / axis=x lineattrs=(color=orangered pattern=4 thickness=1) label=("2050s")  labelpos=min;
refline '31JAN2070'd / axis=x lineattrs=(color=orangered pattern=4 thickness=1) label=("2070s")  labelpos=min;
refline '31JAN2090'd / axis=x lineattrs=(color=orangered pattern=4 thickness=1) label=("2090s")  labelpos=min;
Keylegend "S1"  "S3"  "S5"/ border position= autoitemsize;
run;
quit;

/*---- Mukumu Hospital, Kakamega, Kenya ----*/

ods graphics on / imagename='Kenyan_Mukumu_Malaria_cases_projections2_2022'
    width=28 cm height=22 cm;
        ods listing image_dpi=300 style=myfont;
proc sgpanel data=Pred_malaria_Kenya2 noautolegend;
by GCM;
where Hospital="Mukumu Hospital, Kakamega, Kenya";
panelby  Hospital Scenario /novarname layout=lattice spacing=1 uniscale=column sort=data;
band    x=Month3 lower=LCI1 upper=UCI1 /fill fillattrs=(color=deepskyblue) transparency=0.3 name="S4" legendlabel="95%
Confidence limits";
scatter  x=Month3 y=log_cases2  /markerattrs=(color=mediumslateblue  symbol=circlefilled size=12)transparency=0.1 dataskin=sheen
name="S1" legendlabel="Actual";
Series  x=Month3 y=FOR1 / lineattrs=(color=deeppink Pattern=1 thickness=2) name="S3" legendlabel="Predicted" ;
Pbspline x=Month3 y=FOR1 / lineattrs=(color=yellow Pattern=1 thickness=2) nomarkers nknots=200 maxpoints=150 name="S2"
legendlabel="Smoothed";
rowaxis label="LN(Malaria cases/Mean=487.4)" grid;
colaxis offsetmin=0.05  label="Date" values=( '01JAN1995'd to '01FEB2101'd by 255);
title 'Forecasting malaria cases in relation to rainfall and temperature';
refline '28FEB2007'd / axis=x lineattrs=(pattern=4 color=blue)  labelpos=min name="S5" legendlabel="Start of multi-step forecasts
(2007)";
refline '31JAN2030'd / axis=x lineattrs=(color=orangered pattern=4 thickness=1)  label=("2030s")  labelpos=min;
refline '31JAN2050'd / axis=x lineattrs=(color=orangered pattern=4 thickness=1)  label=("2050s")  labelpos=min;
refline '31JAN2070'd / axis=x lineattrs=(color=orangered pattern=4 thickness=1)  label=("2070s")  labelpos=min;
```

```
refline '31JAN2090'd / axis=x lineattrs=(color=orangered pattern=4 thickness=1)  label=("2090s")  labelpos=min;
Keylegend "S1" "S2" "S3" "S4" "S5"/ border position=bottom across=5 down=1;
run;
quit;

ods graphics on / imagename='Kenyan_Mukumu_Malaria_Ensemble_2022'
    width=28 cm height=26 cm;
          ods listing image_dpi=300;
proc sgpanel data=Pred_malaria_Kenya2 noautolegend;
by GCM;
where Hospital="Mukumu Hospital, Kakamega, Kenya";
panelby  Hospital Scenario /novarname layout=lattice spacing=1 uniscale=column sort=(auto descending);
scatter  x=Month3 y=log_cases2  /markerattrs=(color=mediumslateblue  symbol=circlefilled size=12)transparency=0.1 dataskin=sheen
name="S1" legendlabel="Actual";
Series  x=Month3 y=FOR1 /group=GCM  name="S3" legendlabel="Predicted" ;
rowaxis label="LN(Malaria cases/Mean=487.4)" grid;
colaxis offsetmin=0.05  label="Date" values=( '01JAN1995'd to '01FEB2101'd by 255);
title 'Forecasting malaria cases in relation to rainfall and temperature';
refline '28FEB2007'd / axis=x lineattrs=(pattern=4 color=blue)  labelpos=min name="S5" legendlabel="Start of multi-step forecasts
(2007)";
refline '31JAN2030'd / axis=x lineattrs=(color=orangered pattern=4 thickness=1)  label=("2030s")  labelpos=min;
refline '31JAN2050'd / axis=x lineattrs=(color=orangered pattern=4 thickness=1)  label=("2050s")  labelpos=min;
refline '31JAN2070'd / axis=x lineattrs=(color=orangered pattern=4 thickness=1)  label=("2070s")  labelpos=min;
refline '31JAN2090'd / axis=x lineattrs=(color=orangered pattern=4 thickness=1)  label=("2090s")  labelpos=min;
Keylegend "S1" "S3" "S5"/ border position=bottom autoitemsize;
run;
quit;

/*-----SAS codes (Version 9.4) for fitting the VARMAX (Version 15.1) models to Malaria and Anaemia data for Muleba Hospital, Tanzania
-----*/
```

```
ods graphics on;
proc varmax data=Muleba6 plots=all;
by  GCM Scenario disease age;
where  disease="Anaemia" and age="Under 5 years"  and GCM="MPI_M_MPI_ESM_LR_MPI_SMHI_REMO";
id month3 interval=month align=end;
nloptions tech=newrap maxit=2000 pall;
ods output DiagnostAR=DiagnostAR4 DiagnostWN=DiagnostWN4 ANOVA=ANOVA4 MARoots=MARoots4 ARRoots=ARRoots4
PortmanteauTest=PortmanteauTest4;

  model logp

= mavrain7  max max3 min2/p=1 q=1  /*xlag=(4,5,6)*/  /*nocurrentx*/ /*nseason=12*/
                    printform=univariate
                    print=(corry corrx pcorr pcancorr parcoef Estimates roots diagnose) method=ml minic=(type=aic p=4);

ods output corrxgraph=corrxgraph corrygraph=corrygraph CorrXLags=CorrXLags
CorrXbyVar=CorrXbyVar CorrYLags=CorrYLags CorrYbyVar=CorrYbyVar
PartialAR=PartialAR ParameterEstimates=ParameterEstimates4 ParameterGraph=ParameterGraph
PartialAR=PartialAR PartialARGraph=PartialARGraph PartialCanCorr=PartialCanCorr
PartialCorr=PartialCorr PartialCorrbyVar=PartialCorrbyVar PartialCorrGraph=PartialCorrGraph;
output out=pred_Muleba1 lead=1182 back=0;
run;
ods graphics off;

ods graphics on;
proc varmax data=Muleba6 plots=all;
by  GCM Scenario disease age;
where  disease="Malaria" and age="All ages";
id month3 interval=month align=end;
nloptions tech=newrap maxit=2000 pall;
ods output DiagnostAR=DiagnostAR5 DiagnostWN=DiagnostWN5 ANOVA=ANOVA5 MARoots=MARoots5 ARRoots=ARRoots5
PortmanteauTest=PortmanteauTest5;
```

```
   model logp

=  mavrain7  mavmax3 mavmin2/p=2 q=2  /*xlag=(4,5,6)*/  /*nocurrentx*/ /*nseason=12*/
                    printform=univariate
                    print=(corry corrx pcorr pcancorr parcoef Estimates roots diagnose) method=ml minic=(type=aic p=4);

ods output corrxgraph=corrxgraph corrygraph=corrygraph CorrXLags=CorrXLags
CorrXbyVar=CorrXbyVar CorrYLags=CorrYLags CorrYbyVar=CorrYbyVar
PartialAR=PartialAR ParameterEstimates=ParameterEstimates5 ParameterGraph=ParameterGraph
PartialAR=PartialAR PartialARGraph=PartialARGraph PartialCanCorr=PartialCanCorr
PartialCorr=PartialCorr PartialCorrbyVar=PartialCorrbyVar PartialCorrGraph=PartialCorrGraph;
output out=pred_Muleba2 lead=1181 back=0;
run;
ods graphics off;


ods graphics on;
proc varmax data=Muleba6 plots=all;
by  GCM Scenario disease age;
where  disease="Malaria" and age="Five years and above";
id month3 interval=month align=end;
nloptions tech=newrap maxit=2000 pall;
ods output DiagnostAR=DiagnostAR6 DiagnostWN=DiagnostWN6 ANOVA=ANOVA6 MARoots=MARoots6 ARRoots=ARRoots6
PortmanteauTest=PortmanteauTest6;

   model logp

= mavrain7  mavmax3 mavmin2/p=2 q=2  /*xlag=(4,5,6)*/  /*nocurrentx*/ /*nseason=12*/
                    printform=univariate
                    print=(corry corrx pcorr pcancorr parcoef Estimates roots diagnose) method=ml minic=(type=aic p=4);
```

```
ods output corrxgraph=corrxgraph corrygraph=corrygraph CorrXLags=CorrXLags
CorrXbyVar=CorrXbyVar CorrYLags=CorrYLags CorrYbyVar=CorrYbyVar
PartialAR=PartialAR ParameterEstimates=ParameterEstimates6 ParameterGraph=ParameterGraph
PartialAR=PartialAR PartialARGraph=PartialARGraph PartialCanCorr=PartialCanCorr
PartialCorr=PartialCorr PartialCorrbyVar=PartialCorrbyVar PartialCorrGraph=PartialCorrGraph;
output out=pred_Muleba3 lead=1181 back=0;
run;
ods graphics off;


ods graphics on;
proc varmax data=Muleba6 plots=all;
by GCM  Scenario disease age;
where  disease="Malaria" and age="Under 5 years";
id month3 interval=month align=end;
nloptions tech=newrap maxit=2000 pall;
ods output DiagnostAR=DiagnostAR7 DiagnostWN=DiagnostWN7 ANOVA=ANOVA7 MARoots=MARoots7 ARRoots=ARRoots7
PortmanteauTest=PortmanteauTest7;

  model logp

= mavrain7  mavmax3 mavmin2/p=2 q=2  /*xlag=(4,5,6)*/  /*nocurrentx*/ /*nseason=12*/
                 printform=univariate
                 print=(corry corrx pcorr pcancorr parcoef Estimates roots diagnose) method=ml minic=(type=aic p=4);

ods output corrxgraph=corrxgraph corrygraph=corrygraph CorrXLags=CorrXLags
CorrXbyVar=CorrXbyVar CorrYLags=CorrYLags CorrYbyVar=CorrYbyVar
PartialAR=PartialAR ParameterEstimates=ParameterEstimates7 ParameterGraph=ParameterGraph
PartialAR=PartialAR PartialARGraph=PartialARGraph PartialCanCorr=PartialCanCorr
PartialCorr=PartialCorr PartialCorrbyVar=PartialCorrbyVar PartialCorrGraph=PartialCorrGraph;
output out=pred_Muleba4 lead=1181 back=0;
run;
```

```
ods graphics off;


data Muleba_pred;
set pred_Muleba1-pred_Muleba4;
Month=Month(Month3);
Year=Year (Month3);
Cases=exp(logP);
Pred=exp(FOR1);
if year<=2003            then Period="1996-2002";
if 2016<=year<=2045     then Period="2016-2045";
if 2036<=year<=2065     then Period="2036-2065";
if 2055<=year<=2085     then Period="2055-2085";
if 2071<=year<=2100     then Period="2071-2100";

run;

ods graphics on / imagename='Muleba_Anaemia_cases_projections_2016_under 5 years_join'
     width=28 cm height=22 cm ANTIALIASMAX=150000;
          ods listing image_dpi=300;
proc sgpanel data=Muleba_pred noautolegend;
by GCM;
where Disease="Anaemia" and Age="Under 5 years";
panelby Disease  Scenario  /novarname layout=lattice  spacing=1 uniscale=column sort=(auto descending);
band     x=Month3 lower=LCI1 upper=UCI1 /fill fillattrs=(color=chartreuse) transparency=0.3 name="S4" legendlabel="95% Confidence
limits";
scatter  x=Month3 y=logp  /markerattrs=(color=slateblue  symbol=circlefilled size=12)transparency=0.1 dataskin=sheen name="S1"
legendlabel="Actual";
Series  x=Month3 y=FOR1 / lineattrs=(color=deeppink Pattern=1 thickness=2) name="S3" legendlabel="Predicted";
Pbspline x=Month3 y=FOR1 / lineattrs=(color=blue Pattern=1 thickness=2) nomarkers nknots=200 maxpoints=150 name="S2"
legendlabel="Smoothed";;
rowaxis label="LN(Annaemia cases) for under 5 years" grid;
```

```
colaxis offsetmin=0.05  offsetmax=0.05 label="Date" values=( '01JAN1996'd to '01DEC2100'd by 252);
title 'Forecasting Anaemia cases in relation to rainfall and temperature';
refline '01JAN2002'd / axis=x lineattrs=(pattern=4 color=blue)  labelpos=min name="S5" legendlabel="Start of multi-step forecasts
(2002)";
refline '31JAN2030'd / axis=x lineattrs=(color=orangered pattern=4 thickness=1)  label=("2030s")  labelpos=min;
refline '31JAN2050'd / axis=x lineattrs=(color=orangered pattern=4 thickness=1)  label=("2050s")  labelpos=min;
refline '31JAN2070'd / axis=x lineattrs=(color=orangered pattern=4 thickness=1)  label=("2070s")  labelpos=min;
refline '31JAN2090'd / axis=x lineattrs=(color=orangered pattern=4 thickness=1)  label=("2090s")  labelpos=min;
Keylegend "S1" "S2" "S3" "S4" "S5"/ border position=bottom across=5 down=1;
run;
quit;

ods graphics on / imagename='Muleba_Anaemia_Ensemble_under 5 years'
    width=28 cm height=26 cm ANTIALIASMAX=150000;
        ods listing image_dpi=300;
proc sgpanel data=Muleba_pred noautolegend;
by GCM;
where Disease="Anaemia" and Age="Under 5 years";
panelby Disease  Scenario  /novarname layout=lattice spacing=1 uniscale=column sort=(auto descending);
scatter  x=Month3 y=logp  /markerattrs=(color=slateblue  symbol=circlefilled size=12)transparency=0.1 dataskin=sheen name="S1"
legendlabel="Actual";
Series  x=Month3 y=FOR1 /group=GCM  name="S3" legendlabel="Predicted";
rowaxis label="LN(Annaemia cases) for under 5 years" grid;
colaxis offsetmin=0.05  offsetmax=0.05 label="Date" values=( '01JAN1996'd to '01DEC2100'd by 252);
title 'Forecasting Anaemia cases in relation to rainfall and temperature';
refline '01JAN2002'd / axis=x lineattrs=(pattern=4 color=blue)  labelpos=min name="S5" legendlabel="Start of multi-step forecasts
(2002)";
refline '31JAN2030'd / axis=x lineattrs=(color=orangered pattern=4 thickness=1)  label=("2030s")  labelpos=min;
refline '31JAN2050'd / axis=x lineattrs=(color=orangered pattern=4 thickness=1)  label=("2050s")  labelpos=min;
refline '31JAN2070'd / axis=x lineattrs=(color=orangered pattern=4 thickness=1)  label=("2070s")  labelpos=min;
refline '31JAN2090'd / axis=x lineattrs=(color=orangered pattern=4 thickness=1)  label=("2090s")  labelpos=min;
Keylegend "S1" "S3"  "S5"/ border position=bottom autoitemsize;
```

```
run;
quit;

ods graphics on / imagename='Muleba_Malaria_cases_projections_2016_All ages_join'
    width=28 cm height=20 cm ANTIALIASMAX=134700;
        ods listing image_dpi=300;
proc sgpanel data=pred_Muleba2 noautolegend;
by GCM;
where Disease="Malaria" and Age="All ages";
panelby  Disease Scenario /novarname layout=lattice spacing=1 uniscale=column sort=(auto descending);
band    x=Month3 lower=LCI1 upper=UCI1 /fill fillattrs=(color=lime) transparency=0.3 name="S4" legendlabel="95% Confidence
limits";
scatter  x=Month3 y=logP  /markerattrs=(color=mediumslateblue  symbol=circlefilled size=12)transparency=0.1 dataskin=sheen
name="S1" legendlabel="Actual";
Series  x=Month3 y=FOR1 / lineattrs=(color=Tomato Pattern=1 thickness=2) name="S3" legendlabel="Predicted";
Pbspline x=Month3 y=FOR1 / lineattrs=(color=blue Pattern=1 thickness=2) nomarkers nknots=200 maxpoints=150 name="S2"
legendlabel="Smoothed";
rowaxis label="LN(Malaria cases) for all ages" grid;
colaxis offsetmin=0.05  label="Date" values=( '01JAN1996'd to '01DEC2100'd by 252);
title 'Forecasting malaria cases in relation to rainfall and temperature';
refline '01AUG2002'd / axis=x lineattrs=(pattern=4 color=blue)  labelpos=min name="S5" legendlabel="Start of multi-step forecasts
(2002)";
refline '31JAN2030'd / axis=x lineattrs=(color=orangered pattern=4 thickness=1)  label=("2030s")  labelpos=min;
refline '31JAN2050'd / axis=x lineattrs=(color=orangered pattern=4 thickness=1)  label=("2050s")  labelpos=min;
refline '31JAN2070'd / axis=x lineattrs=(color=orangered pattern=4 thickness=1)  label=("2070s")  labelpos=min;
refline '31JAN2090'd / axis=x lineattrs=(color=orangered pattern=4 thickness=1)  label=("2090s")  labelpos=min;
Keylegend "S1" "S2" "S3" "S4" "S5"/ border position=bottom across=5 down=1;
run;
quit;


ods graphics on / imagename='Muleba_Malaria_Ensemble_All ages'
```

```
     width=28 cm height=26 cm ANTIALIASMAX=134700;
         ods listing image_dpi=300;
proc sgpanel data=pred_Muleba2 noautolegend;
by GCM;
where Disease="Malaria" and Age="All ages";
panelby  Disease Scenario /novarname layout=lattice  spacing=1 uniscale=column sort=(auto descending);
scatter  x=Month3 y=logP  /markerattrs=(color=mediumslateblue  symbol=circlefilled size=12)transparency=0.1 dataskin=sheen
name="S1" legendlabel="Actual";
Series  x=Month3 y=FOR1 /group= name="S3" legendlabel="Predicted";
rowaxis label="LN(Malaria cases) for all ages" grid;
colaxis offsetmin=0.05  label="Date" values=( '01JAN1996'd to '01DEC2100'd by 252);
title 'Forecasting malaria cases in relation to rainfall and temperature';
refline '01AUG2002'd / axis=x lineattrs=(pattern=4 color=blue)  labelpos=min name="S5" legendlabel="Start of multi-step forecasts
(2002)";
refline '31JAN2030'd / axis=x lineattrs=(color=orangered pattern=4 thickness=1)  label=("2030s")  labelpos=min;
refline '31JAN2050'd / axis=x lineattrs=(color=orangered pattern=4 thickness=1)  label=("2050s")  labelpos=min;
refline '31JAN2070'd / axis=x lineattrs=(color=orangered pattern=4 thickness=1)  label=("2070s")  labelpos=min;
refline '31JAN2090'd / axis=x lineattrs=(color=orangered pattern=4 thickness=1)  label=("2090s")  labelpos=min;
Keylegend "S1" "S3"  "S5"/ border position=bottom autoitemsize;
run;
quit;


ods graphics on / imagename='Muleba_Malaria_cases_projections_2016_Five years and above_join'
     width=28 cm height=22 cm ANTIALIASMAX=134700;
         ods listing image_dpi=300;
proc sgpanel data=pred_Muleba3 noautolegend;
by GCM;
where Disease="Malaria" and Age="Five years and above";
panelby  Disease Scenario /novarname layout=lattice spacing=1 uniscale=column sort=(auto descending);
band     x=Month3 lower=LCI1 upper=UCI1 /fill fillattrs=(color=lime) transparency=0.3 name="S4" legendlabel="95% Confidence
limits";
```

```
scatter  x=Month3 y=logP  /markerattrs=(color=mediumslateblue  symbol=circlefilled size=12)transparency=0.1 dataskin=sheen
name="S1" legendlabel="Actual";
Series  x=Month3 y=FOR1 / lineattrs=(color=Tomato Pattern=1 thickness=2) name="S3" legendlabel="Predicted";
Pbspline x=Month3 y=FOR1 / lineattrs=(color=blue Pattern=1 thickness=2) nomarkers nknots=200 maxpoints=150 name="S2"
legendlabel="Smoothed";
rowaxis label="LN(Malaria cases) for five years and above" grid;
colaxis offsetmin=0.05  label="Date" values=( '01JAN1996'd to '01DEC2100'd by 252);
title 'Forecasting malaria cases in relation to rainfall and temperature';
refline '01AUG2002'd / axis=x lineattrs=(pattern=4 color=blue)  labelpos=min name="S5" legendlabel="Start of multi-step forecasts
(2002)";
refline '31JAN2030'd / axis=x lineattrs=(color=orangered pattern=4 thickness=1)  label=("2030s")  labelpos=min;
refline '31JAN2050'd / axis=x lineattrs=(color=orangered pattern=4 thickness=1)  label=("2050s")  labelpos=min;
refline '31JAN2070'd / axis=x lineattrs=(color=orangered pattern=4 thickness=1)  label=("2070s")  labelpos=min;
refline '31JAN2090'd / axis=x lineattrs=(color=orangered pattern=4 thickness=1)  label=("2090s")  labelpos=min;
Keylegend "S1" "S2" "S3" "S4" "S5"/ border position=bottom across=5 down=1;
run;
quit;


ods graphics on / imagename='Muleba_Malaria_Ensemble_Five years and above'
     width=28 cm height=26 cm ANTIALIASMAX=134700;
          ods listing image_dpi=300;
proc sgpanel data=pred_Muleba3 noautolegend;
by GCM;
where Disease="Malaria" and Age="Five years and above";
panelby  Disease Scenario /novarname layout=lattice  spacing=1 uniscale=column sort=(auto descending);
scatter  x=Month3 y=logP  /markerattrs=(color=mediumslateblue  symbol=circlefilled size=12)transparency=0.1 dataskin=sheen
name="S1" legendlabel="Actual";
Series  x=Month3 y=FOR1 /group= name="S3" legendlabel="Predicted";
rowaxis label="LN(Malaria cases) for five years and above" grid;
colaxis offsetmin=0.05  label="Date" values=( '01JAN1996'd to '01DEC2100'd by 252);
```

```
title 'Forecasting malaria cases in relation to rainfall and temperature';
refline '01AUG2002'd / axis=x lineattrs=(pattern=4 color=blue)  labelpos=min name="S5" legendlabel="Start of multi-step forecasts
(2002)";
refline '31JAN2030'd / axis=x lineattrs=(color=orangered pattern=4 thickness=1)  label=("2030s")  labelpos=min;
refline '31JAN2050'd / axis=x lineattrs=(color=orangered pattern=4 thickness=1)  label=("2050s")  labelpos=min;
refline '31JAN2070'd / axis=x lineattrs=(color=orangered pattern=4 thickness=1)  label=("2070s")  labelpos=min;
refline '31JAN2090'd / axis=x lineattrs=(color=orangered pattern=4 thickness=1)  label=("2090s")  labelpos=min;
Keylegend "S1" "S2" "S3" "S4" "S5"/ border position= autoitemsize;
run;
quit;


ods graphics on / imagename='Muleba_Malaria_cases_projections_2016_Under 5 years_join'
    width=28 cm height=22 cm ANTIALIASMAX=134700;
        ods listing image_dpi=300;
proc sgpanel data=pred_Muleba4 noautolegend;
by GCM;
where Disease="Malaria" and Age="Under 5 years";
panelby  Disease Scenario /novarname layout=lattice /*columns=1 rows=1*/ spacing=1 uniscale=column sort=(auto descending);
band    x=Month3 lower=LCI1 upper=UCI1 /fill fillattrs=(color=lime) transparency=0.3 name="S4" legendlabel="95% Confidence
limits";
scatter  x=Month3 y=logP  /markerattrs=(color=mediumslateblue  symbol=circlefilled size=12)transparency=0.1 dataskin=sheen
name="S1" legendlabel="Actual";
Series  x=Month3 y=FOR1 / lineattrs=(color=Tomato Pattern=1 thickness=2) name="S3" legendlabel="Predicted";
Pbspline x=Month3 y=FOR1 / lineattrs=(color=blue Pattern=1 thickness=2) nomarkers nknots=200 maxpoints=150 name="S2"
legendlabel="Smoothed";
rowaxis label="LN(Malaria cases)  for under five years" grid;
colaxis offsetmin=0.05  label="Date" values=( '01JAN1996'd to '01DEC2100'd by 252);
title 'Forecasting malaria cases in relation to rainfall and temperature';
refline '01AUG2002'd / axis=x lineattrs=(pattern=4 color=blue)  labelpos=min name="S5" legendlabel="Start of multi-step forecasts
(2002)";
refline '31JAN2030'd / axis=x lineattrs=(color=orangered pattern=4 thickness=1)  label=("2030s")  labelpos=min;
```

```
refline '31JAN2050'd / axis=x lineattrs=(color=orangered pattern=4 thickness=1)  label=("2050s")  labelpos=min;
refline '31JAN2070'd / axis=x lineattrs=(color=orangered pattern=4 thickness=1)  label=("2070s")  labelpos=min;
refline '31JAN2090'd / axis=x lineattrs=(color=orangered pattern=4 thickness=1)  label=("2090s")  labelpos=min;
Keylegend "S1" "S2" "S3" "S4" "S5"/ border position=bottom across=5 down=1;
run;
quit;


ods graphics on / imagename='Muleba_Malaria_Ensemble_Under 5 years'
    width=28 cm height=26 cm ANTIALIASMAX=134700;
         ods listing image_dpi=300;
proc sgpanel data=pred_Muleba4 noautolegend;
by GCM;
where Disease="Malaria" and Age="Under 5 years";
panelby  Disease Scenario /novarname layout=lattice  spacing=1 uniscale=column sort=(auto descending);
scatter  x=Month3 y=logP  /markerattrs=(color=mediumslateblue  symbol=circlefilled size=12)transparency=0.1 dataskin=sheen
name="S1" legendlabel="Actual";
Series  x=Month3 y=FOR1 / group=GCM name="S3" legendlabel="Predicted";
rowaxis label="LN(Malaria cases)  for under five years" grid;
colaxis offsetmin=0.05  label="Date" values=( '01JAN1996'd to '01DEC2100'd by 252);
title 'Forecasting malaria cases in relation to rainfall and temperature';
refline '01AUG2002'd / axis=x lineattrs=(pattern=4 color=blue)  labelpos=min name="S5" legendlabel="Start of multi-step forecasts
(2002)";
refline '31JAN2030'd / axis=x lineattrs=(color=orangered pattern=4 thickness=1)  label=("2030s")  labelpos=min;
refline '31JAN2050'd / axis=x lineattrs=(color=orangered pattern=4 thickness=1)  label=("2050s")  labelpos=min;
refline '31JAN2070'd / axis=x lineattrs=(color=orangered pattern=4 thickness=1)  label=("2070s")  labelpos=min;
refline '31JAN2090'd / axis=x lineattrs=(color=orangered pattern=4 thickness=1)  label=("2090s")  labelpos=min;
Keylegend "S1" "S3" "S5"/ border position=bottom autoitemsize;
run;
quit;
```

```
/*-----SAS codes for fitting models to the Malaria data for Uganda--*/;
/*-----SAS codes (Version 9.4) for fitting the VARMAX (Version 15.1) models to Malaria data for Uganda -----*/


ods graphics on;
proc varmax data=Malaria_Uganda5 plots=all;
by  GCM Scenario age;
where  age="Five years and above";
id month3 interval=year align=begin;
nloptions tech=newrap maxit=2000 pall;
ods output DiagnostAR=DiagnostAR8 DiagnostWN=DiagnostWN8 ANOVA=ANOVA8 MARoots=MARoots8 ARRoots=ARRoots8
PortmanteauTest=PortmanteauTest8;

  model logtotal

= mavannualrain2  mavmax3 mavmin2/p=2 q=2  /*xlag=(4,5,6)*/  /*nocurrentx*/ /*nseason=12*/
                    printform=univariate
                    print=(corry corrx pcorr pcancorr parcoef Estimates roots diagnose) method=ml minic=(type=aic p=3);

ods output corrxgraph=corrxgraph corrygraph=corrygraph CorrXLags=CorrXLags
CorrXbyVar=CorrXbyVar CorrYLags=CorrYLags CorrYbyVar=CorrYbyVar
PartialAR=PartialAR ParameterEstimates=ParameterEstimates8 ParameterGraph=ParameterGraph
PartialAR=PartialAR PartialARGraph=PartialARGraph PartialCanCorr=PartialCanCorr
PartialCorr=PartialCorr PartialCorrbyVar=PartialCorrbyVar PartialCorrGraph=PartialCorrGraph;
output out=pred_uganda1 lead=90 back=0;
run;
ods graphics off;



ods graphics on;
proc varmax data=Malaria_Uganda5 plots=all;
```

```
by  GCM Scenario age;
where  age="Under five years" ;
id month3 interval=year align=end;
nloptions tech=newrap maxit=2000 pall;
ods output DiagnostAR=DiagnostAR9 DiagnostWN=DiagnostWN9 ANOVA=ANOVA9 MARoots=MARoots9 ARRoots=ARRoots9
PortmanteauTest=PortmanteauTest9;

  model logtotal

=  mavannualrain2   mavmax3 mavmin2/p=2 q=2  /*xlag=(4,5,6)*/  /*nocurrentx*/ /*nseason=12*/
                    printform=univariate
                    print=(corry corrx pcorr pcancorr parcoef Estimates roots diagnose) method=ml minic=(type=aic p=3);

ods output corrxgraph=corrxgraph corrygraph=corrygraph CorrXLags=CorrXLags
CorrXbyVar=CorrXbyVar CorrYLags=CorrYLags CorrYbyVar=CorrYbyVar
PartialAR=PartialAR ParameterEstimates=ParameterEstimates9 ParameterGraph=ParameterGraph
PartialAR=PartialAR PartialARGraph=PartialARGraph PartialCanCorr=PartialCanCorr
PartialCorr=PartialCorr PartialCorrbyVar=PartialCorrbyVar PartialCorrGraph=PartialCorrGraph;
output out=pred_uganda2 lead=90 back=0;
run;
ods graphics off;

data pred_uganda12; set pred_uganda1 pred_uganda2;
Year=Year(Month3);
Region="Uganda";
run;

data Uganda_pred;
set pred_uganda12;
Cases_prop=exp(logtotal);
Pred_Prop=exp(FOR1);
if year<=2010      then Period="1997-2010";
```

```
if 2016<=year<=2045    then Period="2016-2045";
if 2036<=year<=2065    then Period="2036-2065";
if 2055<=year<=2085    then Period="2055-2085";
if 2071<=year<=2100    then Period="2071-2100";
run;



ods graphics on / imagename='Uganda_Malaria_cases_projections_2016_Five years and above_original'
    width=28 cm height=22 cm;
          ods listing image_dpi=300 style=myfont;
proc sgpanel data=pred_uganda12  noautolegend;
by GCM;
where age="Five years and above";
panelby  Region Scenario /novarname layout=lattice /*columns=1 rows=1*/ spacing=1 uniscale=column sort=(auto descending);
band    x=Month3 lower=LCI1 upper=UCI1 /fill fillattrs=(color=skyblue) transparency=0.3 name="S4" legendlabel="95% Confidence limits";
scatter  x=Month3  y=logtotal   /markerattrs=(color=mediumslateblue  symbol=circlefilled size=12)transparency=0.1 dataskin=sheen name="S1" legendlabel="Actual";
Series   x=Month3 y=FOR1 / lineattrs=(color=Tomato Pattern=1 thickness=2) name="S3" legendlabel="Predicted";
Pbspline x=Month3  y=FOR1 / lineattrs=(color=blue Pattern=1 thickness=2) nomarkers nknots=200 maxpoints=150 name="S2" legendlabel="Smoothed";
rowaxis label="LN(Malaria cases/Total)  for five years and above" grid;
colaxis offsetmin=0.05  label="Date" values=( '01JAN1996'd to '01DEC2100'd by 252);
title 'Forecasting malaria cases in relation to rainfall and temperature';
refline '31JAN2011'd / axis=x lineattrs=(pattern=4 color=blue)  labelpos=min name="S5" legendlabel="Start of multi-step forecasts (2011)";
refline '31JAN2030'd / axis=x lineattrs=(color=orangered pattern=4 thickness=1)  label=("2030s")  labelpos=min;
refline '31JAN2050'd / axis=x lineattrs=(color=orangered pattern=4 thickness=1)  label=("2050s")  labelpos=min;
refline '31JAN2070'd / axis=x lineattrs=(color=orangered pattern=4 thickness=1)  label=("2070s")  labelpos=min;
refline '31JAN2090'd / axis=x lineattrs=(color=orangered pattern=4 thickness=1)  label=("2090s")  labelpos=min;
Keylegend "S1" "S2" "S3" "S4" "S5"/ border position=bottom across=5 down=1;
```

```
run;
quit;


ods graphics on / imagename='Uganda_Malaria_Ensemble_Five years and above'
     width=28 cm height=26 cm ANTIALIASMAX=150000 outputfmt=png
     imagemap=on;
          ods listing image_dpi=300;
proc sgpanel data=pred_uganda12  noautolegend;
by GCM;
where age="Five years and above";
panelby  Region Scenario /novarname layout=lattice spacing=1 uniscale=column sort=(auto descending);
scatter  x=Month3  y=logtotal   /markerattrs=(color=mediumslateblue  symbol=circlefilled size=12)transparency=0.1 dataskin=sheen
name="S1" legendlabel="Actual";
Series   x=Month3 y=FOR1 / group=GCM  name="S3" legendlabel="Predicted";
rowaxis label="LN(Malaria cases/Total)  for five years and above" grid;
colaxis offsetmin=0.05  label="Date" values=( '01JAN1996'd to '01DEC2100'd by 252);
title 'Forecasting malaria cases in relation to rainfall and temperature';
refline '31JAN2011'd / axis=x lineattrs=(pattern=4 color=blue)  labelpos=min name="S5" legendlabel="Start of multi-step forecasts
(2011)";
refline '31JAN2030'd / axis=x lineattrs=(color=orangered pattern=4 thickness=1)  label=("2030s")  labelpos=min;
refline '31JAN2050'd / axis=x lineattrs=(color=orangered pattern=4 thickness=1)  label=("2050s")  labelpos=min;
refline '31JAN2070'd / axis=x lineattrs=(color=orangered pattern=4 thickness=1)  label=("2070s")  labelpos=min;
refline '31JAN2090'd / axis=x lineattrs=(color=orangered pattern=4 thickness=1)  label=("2090s")  labelpos=min;
Keylegend "S1" "S3" "S5"/ border position=bottom  autoitemsize;
run;
quit;


ods graphics on / imagename='Uganda_Malaria_cases_projections_2016_Under Five years_original'
     width=28 cm height=22 cm;
          ods listing image_dpi=300 style=myfont;
```

```
proc sgpanel data=pred_uganda12  noautolegend;
by GCM;
where age="Under five years";
panelby  Region Scenario /novarname layout=lattice spacing=1 uniscale=column sort=(auto descending);
band    x=Month3 lower=LCI1 upper=UCI1 /fill fillattrs=(color=skyblue) transparency=0.3 name="S4" legendlabel="95% Confidence
limits";
scatter  x=Month3 y=logtotal   /markerattrs=(color=mediumslateblue  symbol=circlefilled size=12)transparency=0.1 dataskin=sheen
name="S1" legendlabel="Actual";
Series   x=Month3 y=FOR1 / lineattrs=(color=Tomato Pattern=1 thickness=2) name="S3" legendlabel="Predicted";
Pbspline x=Month3  y=FOR1 / lineattrs=(color=blue Pattern=1 thickness=2) nomarkers nknots=200 maxpoints=150 name="S2"
legendlabel="Smoothed";
rowaxis label="LN(Malaria cases/Total)  for under five years" grid;
colaxis offsetmin=0.05  label="Date" values=( '01JAN1996'd to '01DEC2100'd by 252) ;
title 'Forecasting malaria cases in relation to rainfall and temperature';
refline '31JAN2011'd / axis=x lineattrs=(pattern=4 color=blue)  labelpos=min name="S5" legendlabel="Start of multi-step forecasts
(2011)";
refline '31JAN2030'd / axis=x lineattrs=(color=orangered pattern=4 thickness=1)  label=("2030s")  labelpos=min;
refline '31JAN2050'd / axis=x lineattrs=(color=orangered pattern=4 thickness=1)  label=("2050s")  labelpos=min;
refline '31JAN2070'd / axis=x lineattrs=(color=orangered pattern=4 thickness=1)  label=("2070s")  labelpos=min;
refline '31JAN2090'd / axis=x lineattrs=(color=orangered pattern=4 thickness=1)  label=("2090s")  labelpos=min;
Keylegend "S1" "S2" "S3" "S4" "S5"/ border position=bottom across=5 down=1;
run;
quit;


ods graphics on / imagename='Uganda_Malaria_Ensemble_Under Five years'
    width=28 cm height=26 cm ANTIALIASMAX=150000;
        ods listing image_dpi=300;
proc sgpanel data=pred_uganda12  noautolegend;
by GCM;
where age="Under five years";
panelby  Region Scenario /novarname layout=lattice  spacing=1 uniscale=column sort=(auto descending);
```

```
scatter  x=Month3 y=logtotal   /markerattrs=(color=mediumslateblue  symbol=circlefilled size=12)transparency=0.1 dataskin=sheen
name="S1" legendlabel="Actual";
Series   x=Month3 y=FOR1 / group=GCM name="S3" legendlabel="Predicted";
rowaxis label="LN(Malaria cases/Total)  for under five years" grid;
colaxis offsetmin=0.05  label="Date" values=( '01JAN1996'd to '01DEC2100'd by 252) ;
title 'Forecasting malaria cases in relation to rainfall and temperature';
refline '31JAN2011'd / axis=x lineattrs=(pattern=4 color=blue)  labelpos=min name="S5" legendlabel="Start of multi-step forecasts
(2011)";
refline '31JAN2030'd / axis=x lineattrs=(color=orangered pattern=4 thickness=1)  label=("2030s")  labelpos=min;
refline '31JAN2050'd / axis=x lineattrs=(color=orangered pattern=4 thickness=1)  label=("2050s")  labelpos=min;
refline '31JAN2070'd / axis=x lineattrs=(color=orangered pattern=4 thickness=1)  label=("2070s")  labelpos=min;
refline '31JAN2090'd / axis=x lineattrs=(color=orangered pattern=4 thickness=1)  label=("2090s")  labelpos=min;
Keylegend "S1" "S3" "S5"/ border position=bottom
 autoitemsize;
run;
quit;
```

SI 11

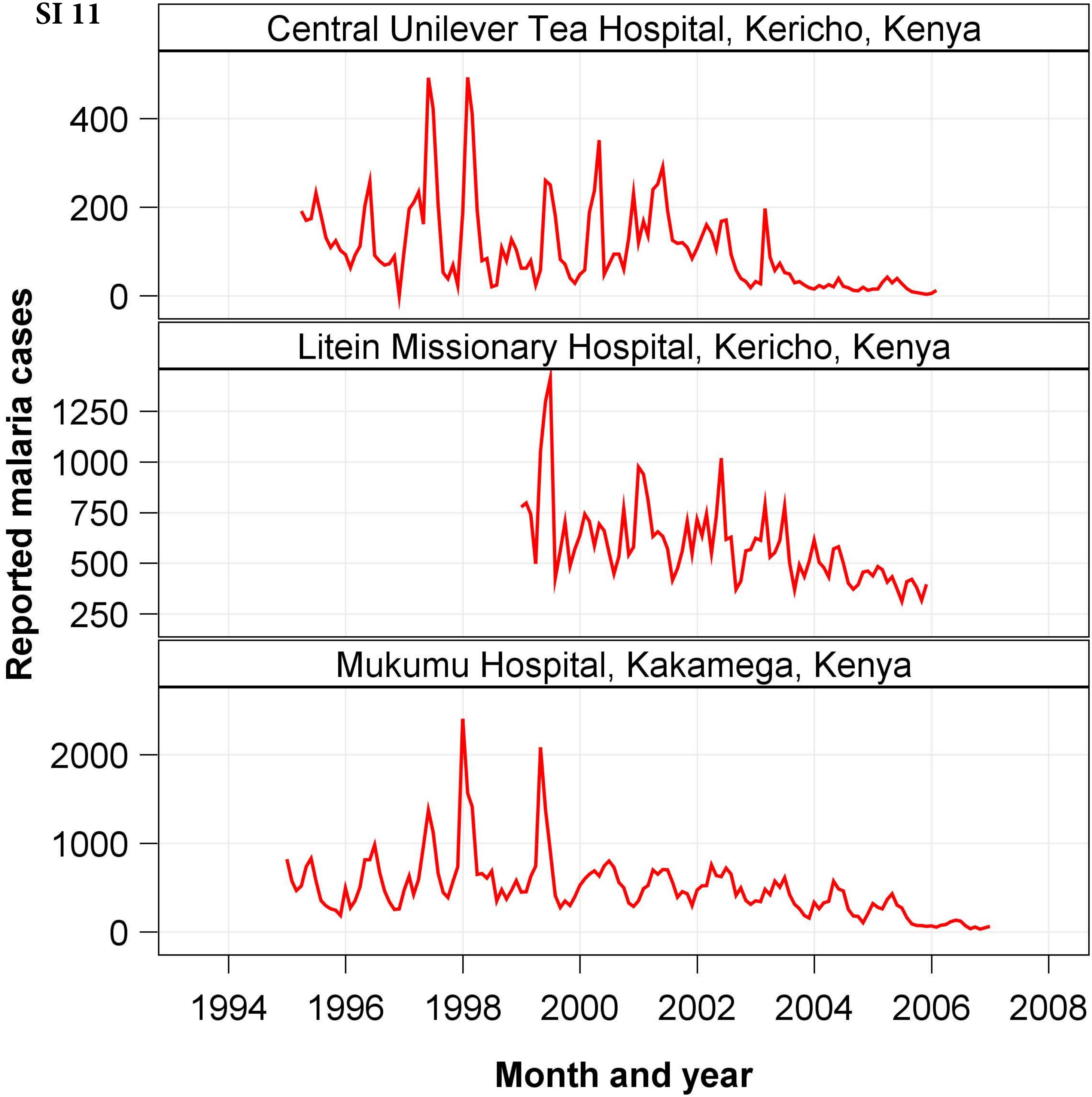
Central Unilever Tea Hospital, Kericho, Kenya
Litein Missionary Hospital, Kericho, Kenya
Mukumu Hospital, Kakamega, Kenya
Reported malaria cases
400
200
0
1250
1000
750
500
250
2000
1000
0
1994
1996
1998
2000
2002
2004
2006
2008
Month and year

SI 12

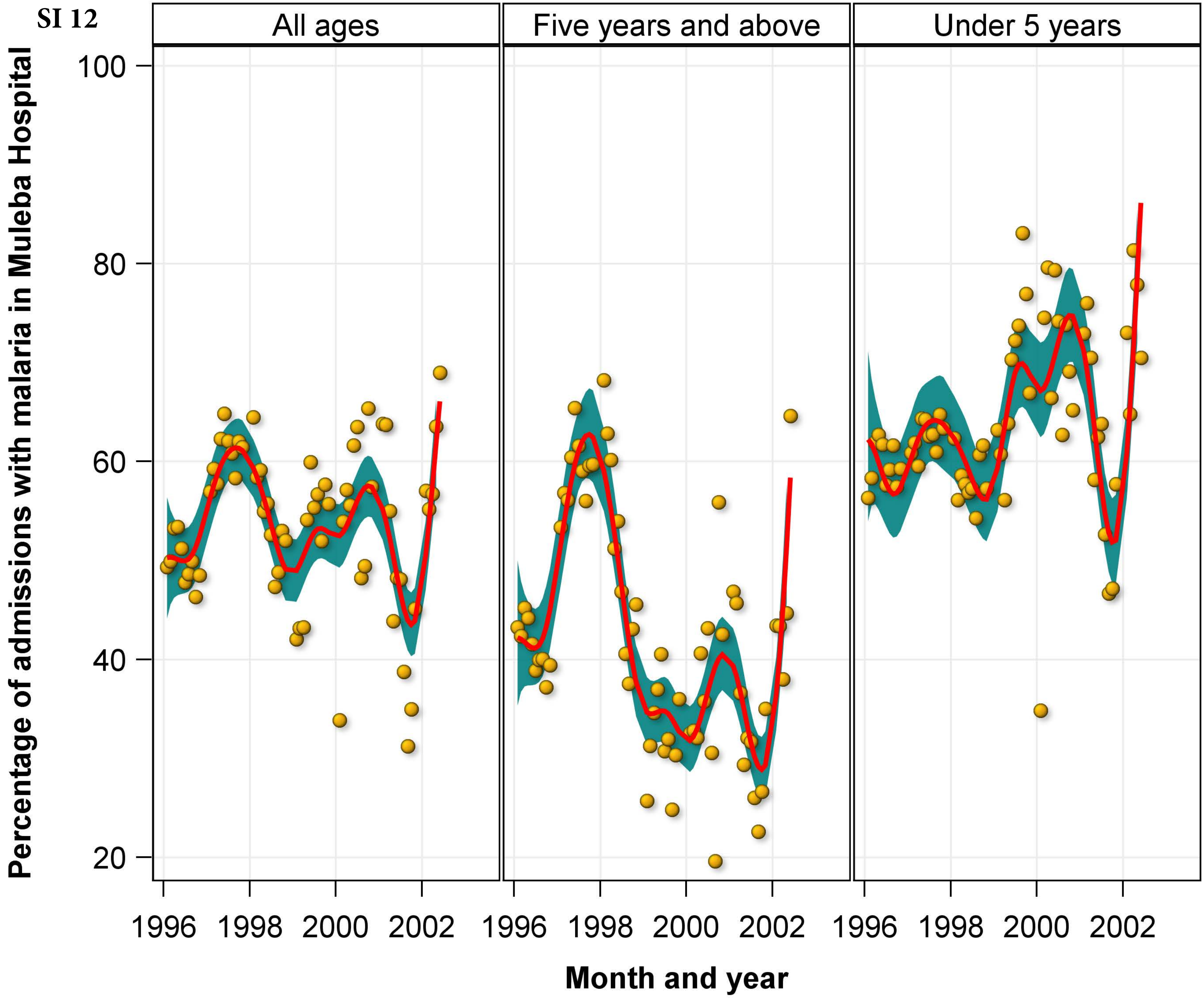

All ages
Five years and above
Under 5 years
Percentage of admissions with malaria in Muleba Hospital
100
80
60
40
20
1996
1998
2000
2002
Month and year

SI 13

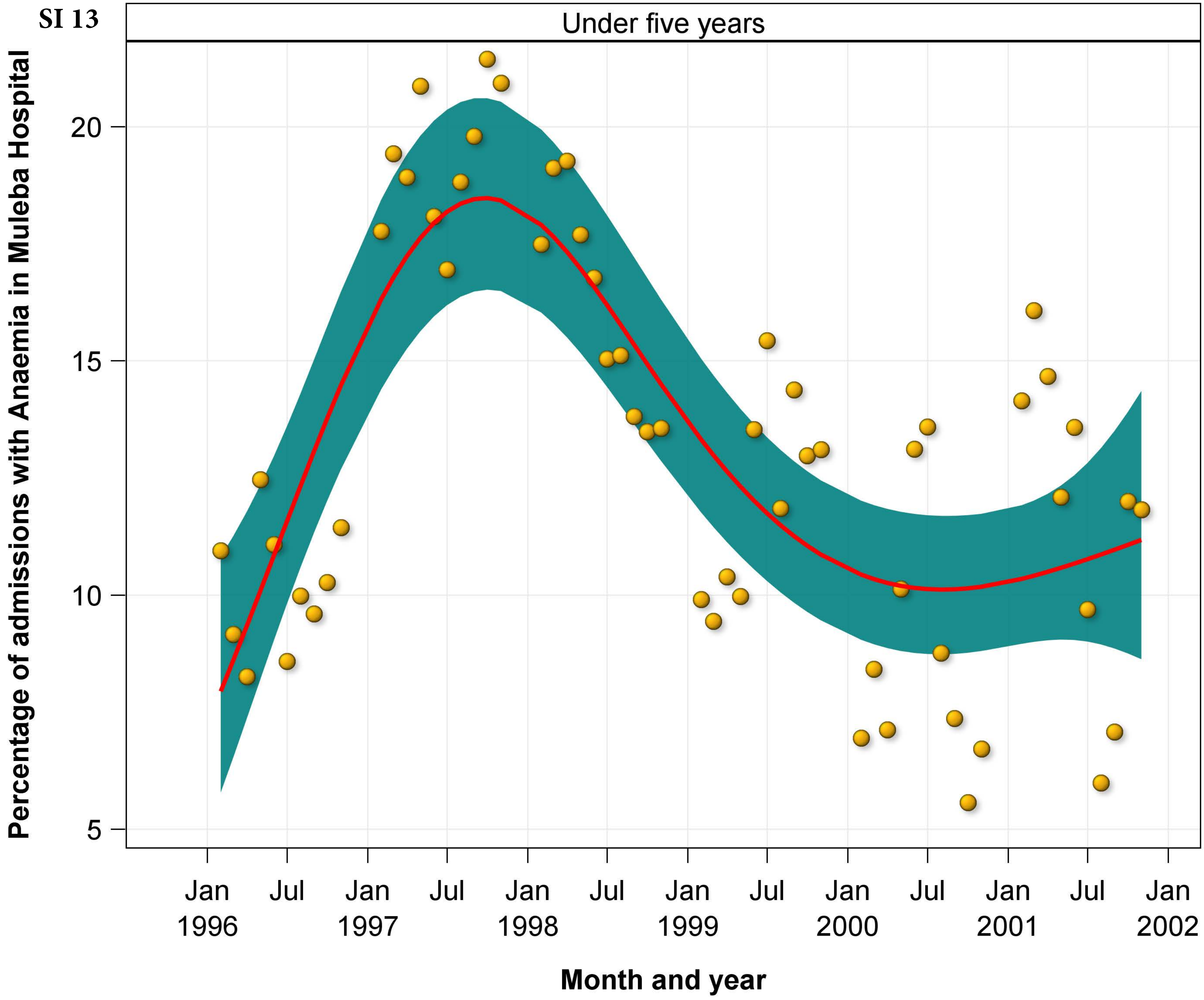
Under five years
Percentage of admissions with Anaemia in Muleba Hospital
20
15
10
5
Jan 1996
Jul
Jan 1997
Jul
Jan 1998
Jul
Jan 1999
Jul
Jan 2000
Jul
Jan 2001
Jul
Jan 2002
Month and year

SI 14

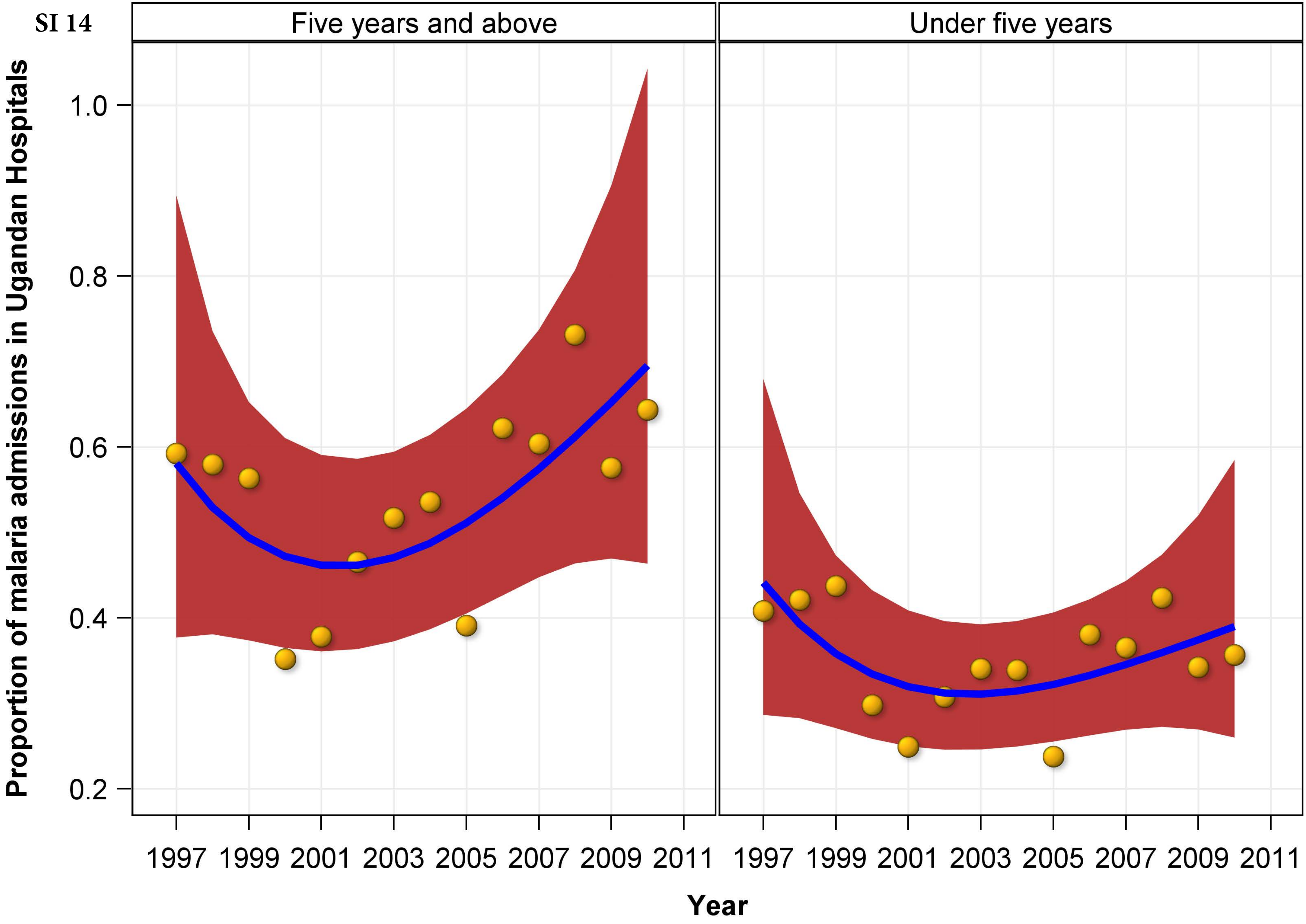

Five years and above
Under five years
Proportion of malaria admissions in Ugandan Hospitals
1.0
0.8
0.6
0.4
0.2
1997
1999
2001
2003
2005
2007
2009
2011
Year

SI15

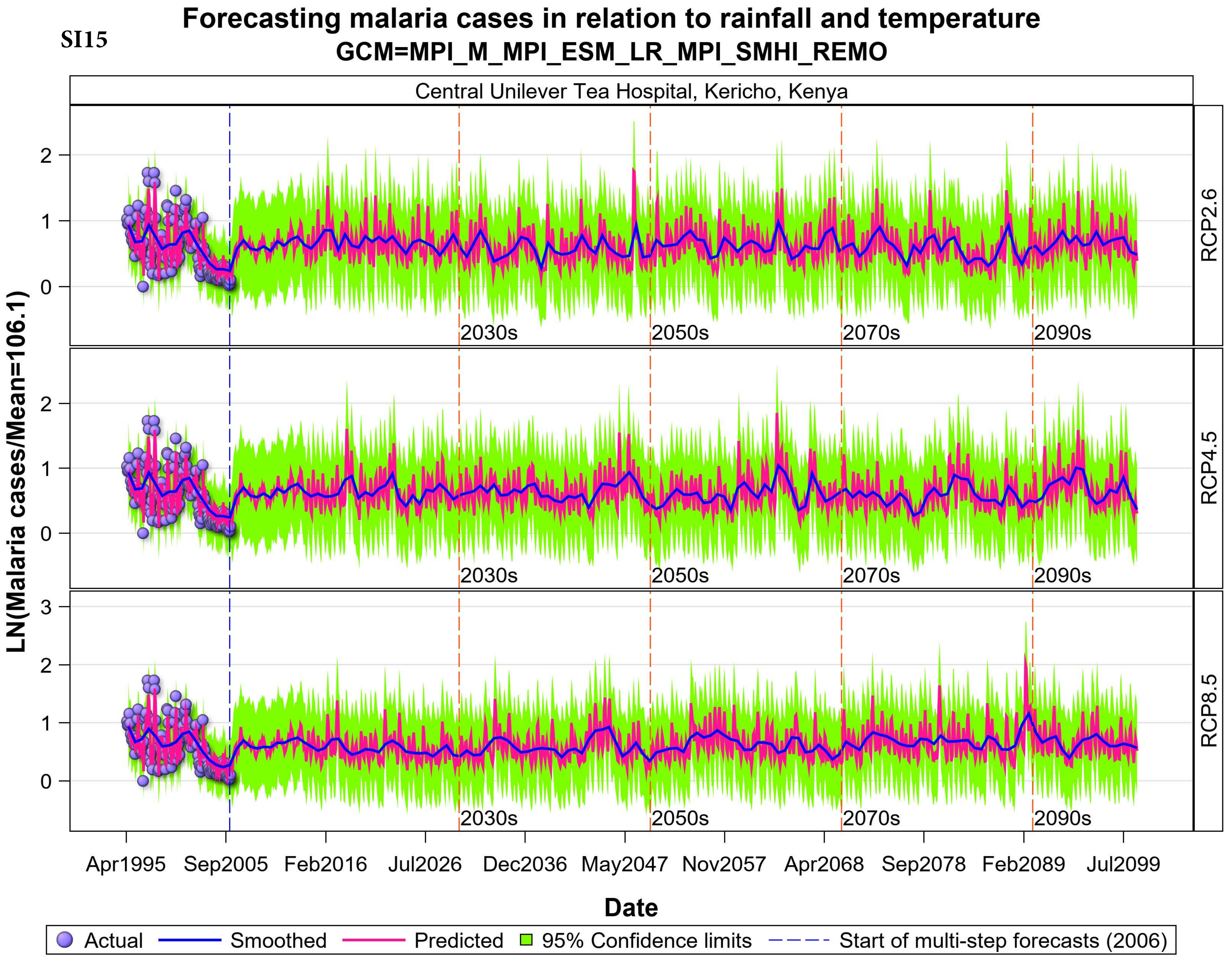

Forecasting malaria cases in relation to rainfall and temperature
GCM=MPI_M_MPI_ESM_LR_MPI_SMHI_REMO
Central Unilever Tea Hospital, Kericho, Kenya
RCP2.6
RCP4.5
RCP8.5
2030s
2050s
2070s
2090s
LN(Malaria cases/Mean=106.1)
Apr1995
Sep2005
Feb2016
Jul2026
Dec2036
May2047
Nov2057
Apr2068
Sep2078
Feb2089
Jul2099
Date
Actual
Smoothed
Predicted
95% Confidence limits
Start of multi-step forecasts (2006)

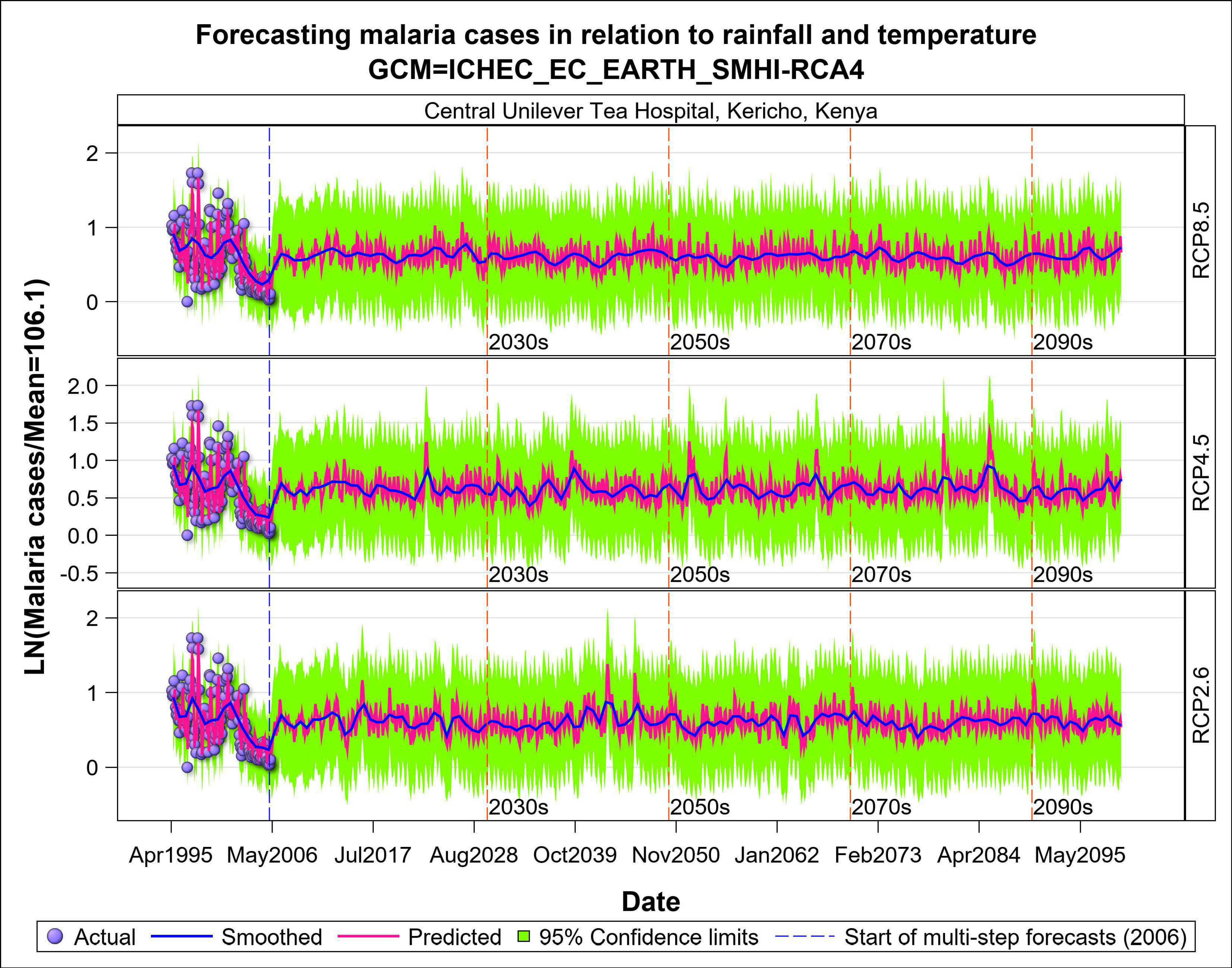
Forecasting malaria cases in relation to rainfall and temperature
GCM=ICHEC_EC_EARTH_SMHI-RCA4
Central Unilever Tea Hospital, Kericho, Kenya
LN(Malaria cases/Mean=106.1)
RCP8.5
RCP4.5
RCP2.6
2030s
2050s
2070s
2090s
Apr1995
May2006
Jul2017
Aug2028
Oct2039
Nov2050
Jan2062
Feb2073
Apr2084
May2095
Date
Actual
Smoothed
Predicted
95% Confidence limits
Start of multi-step forecasts (2006)

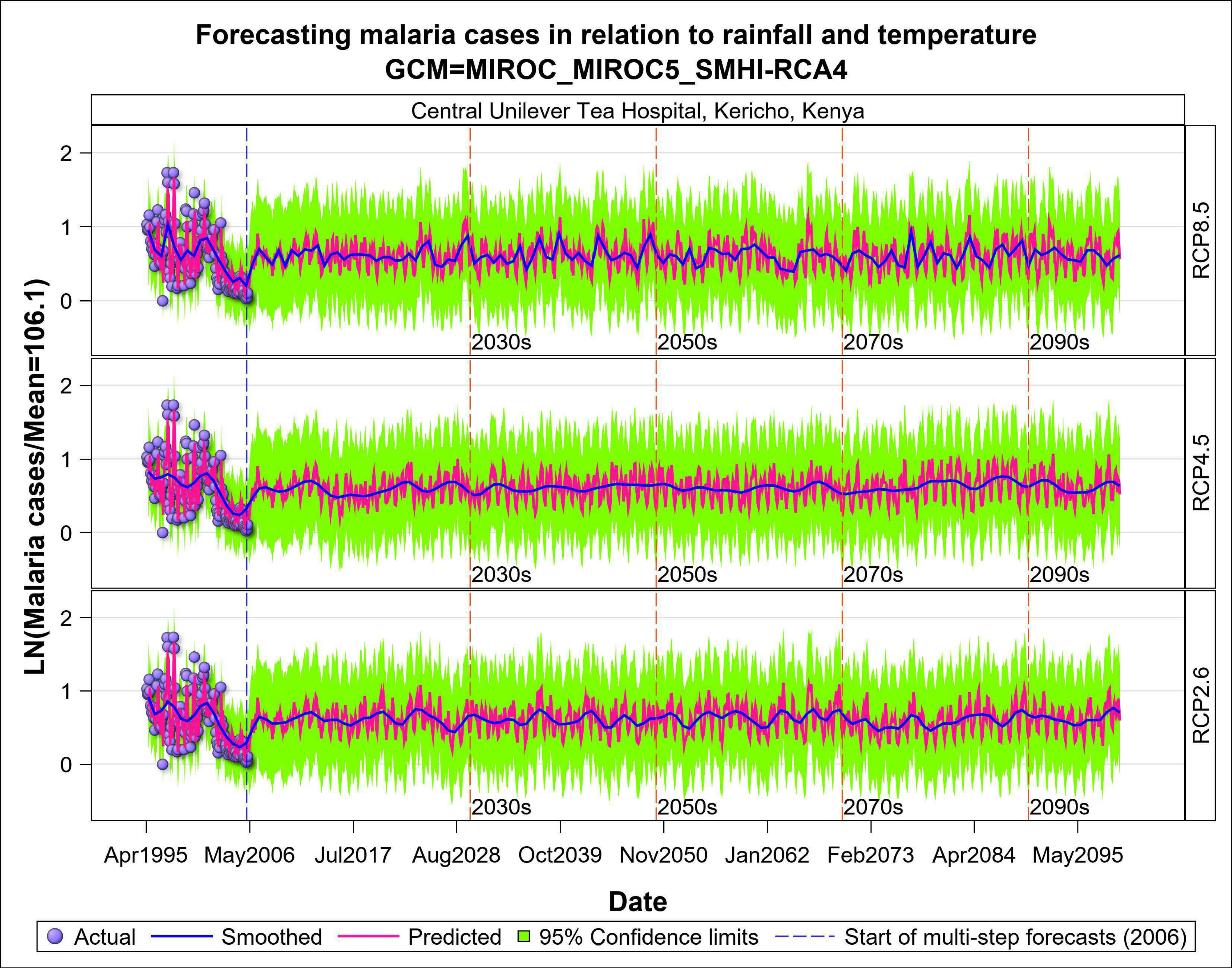
Forecasting malaria cases in relation to rainfall and temperature
GCM=MIROC_MIROC5_SMHI-RCA4
Central Unilever Tea Hospital, Kericho, Kenya
RCP8.5
RCP4.5
RCP2.6
2030s
2050s
2070s
2090s
LN(Malaria cases/Mean=106.1)
Date
Apr1995
May2006
Jul2017
Aug2028
Oct2039
Nov2050
Jan2062
Feb2073
Apr2084
May2095
Actual
Smoothed
Predicted
95% Confidence limits
Start of multi-step forecasts (2006)

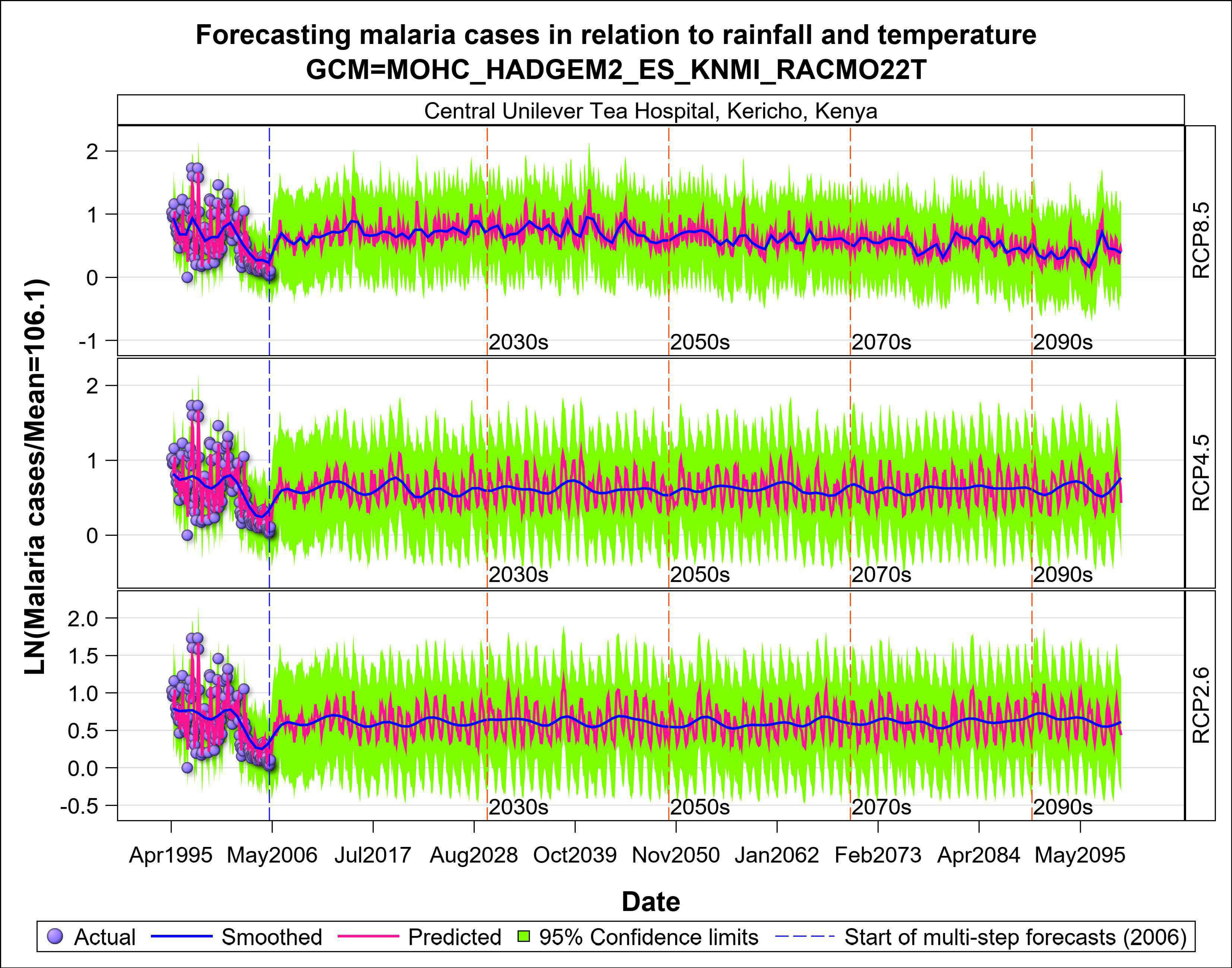
Forecasting malaria cases in relation to rainfall and temperature
GCM=MOHC_HADGEM2_ES_KNMI_RACMO22T
Central Unilever Tea Hospital, Kericho, Kenya
RCP8.5
RCP4.5
RCP2.6
2030s
2050s
2070s
2090s
LN(Malaria cases/Mean=106.1)
Apr1995
May2006
Jul2017
Aug2028
Oct2039
Nov2050
Jan2062
Feb2073
Apr2084
May2095
Date
Actual
Smoothed
Predicted
95% Confidence limits
Start of multi-step forecasts (2006)

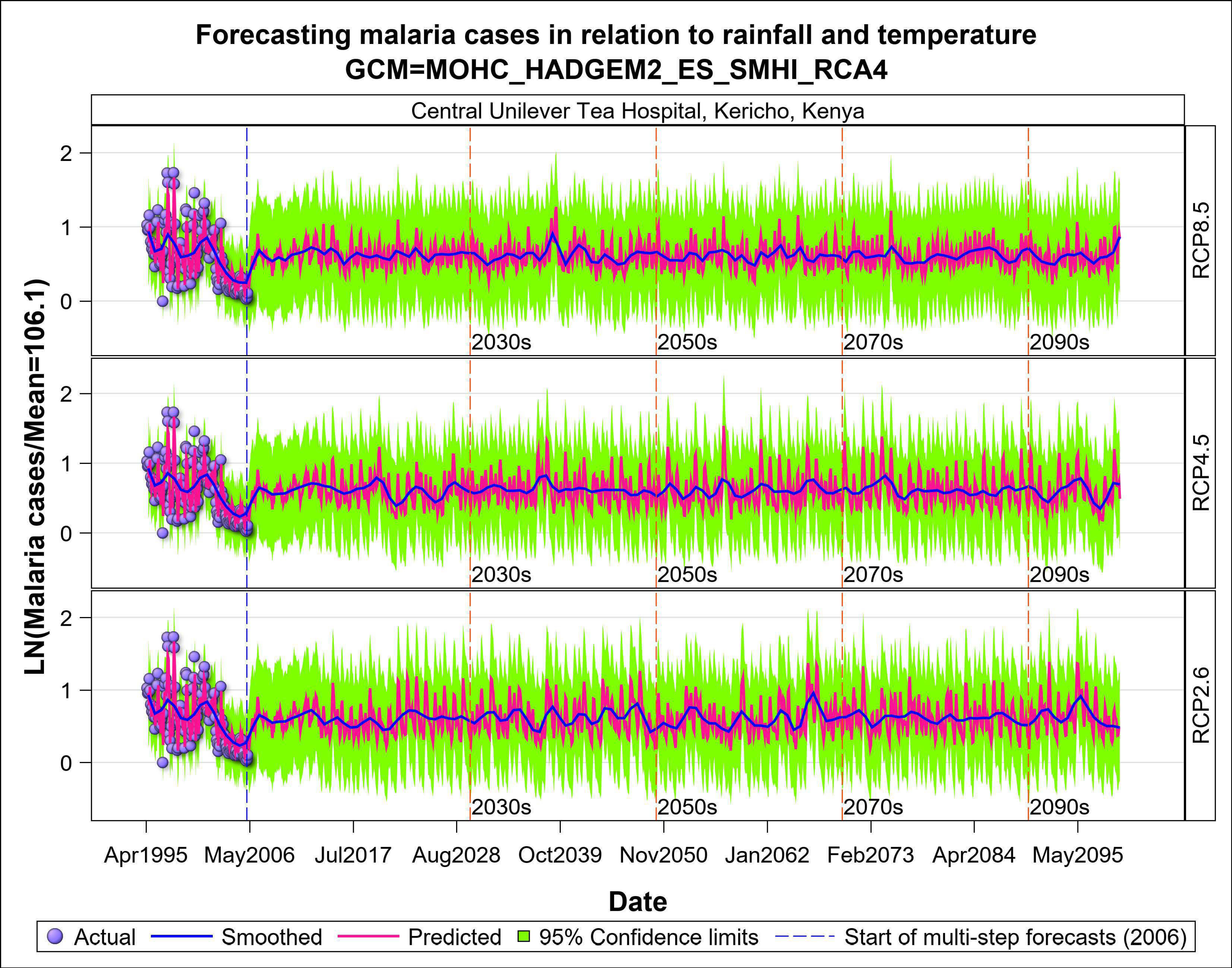

Forecasting malaria cases in relation to rainfall and temperature
GCM=MOHC_HADGEM2_ES_SMHI_RCA4
Central Unilever Tea Hospital, Kericho, Kenya
RCP8.5
RCP4.5
RCP2.6
2030s
2050s
2070s
2090s
LN(Malaria cases/Mean=106.1)
2
1
0
Apr1995
May2006
Jul2017
Aug2028
Oct2039
Nov2050
Jan2062
Feb2073
Apr2084
May2095
Date
Actual
Smoothed
Predicted
95% Confidence limits
Start of multi-step forecasts (2006)

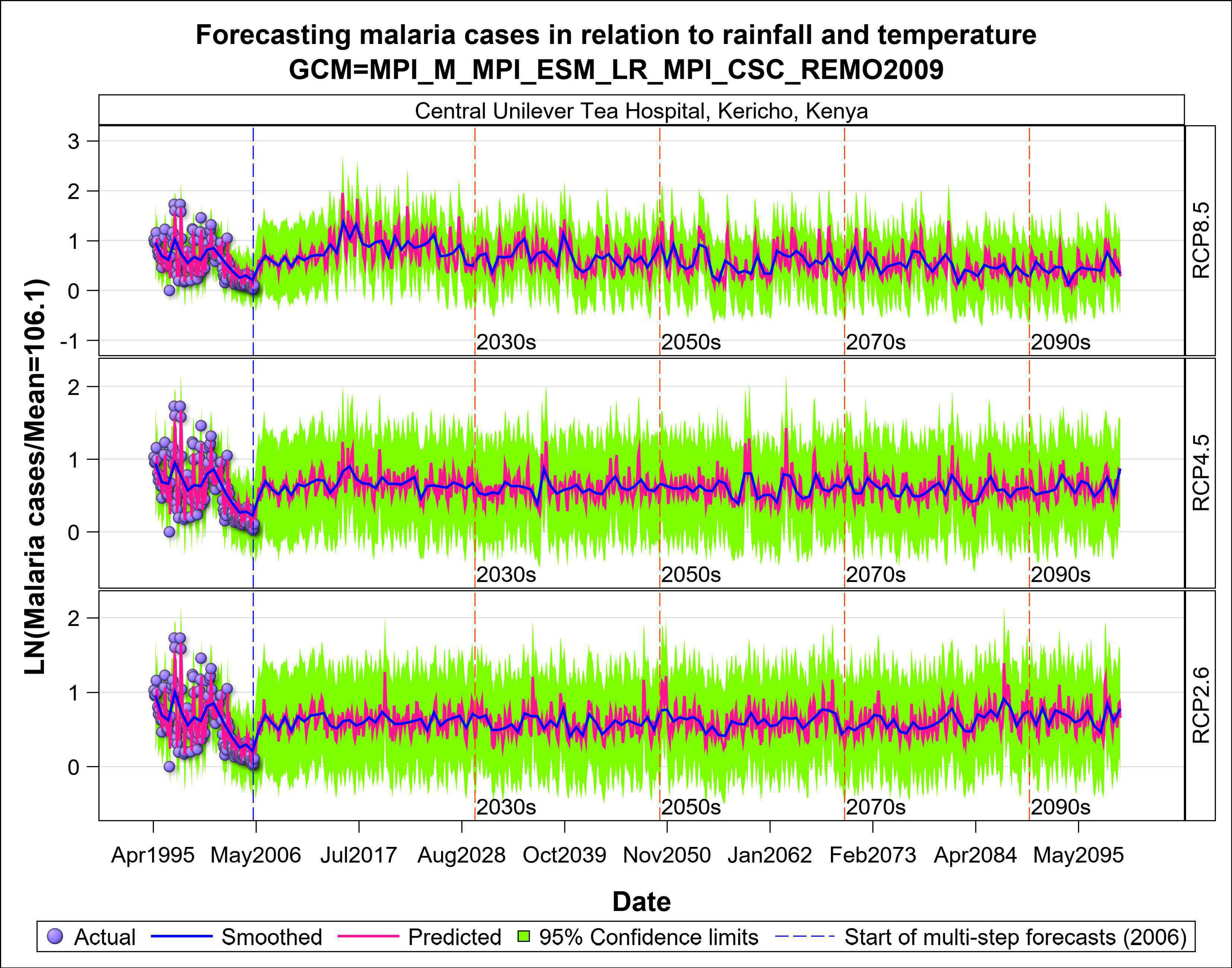

Forecasting malaria cases in relation to rainfall and temperature
GCM=MPI_M_MPI_ESM_LR_MPI_CSC_REMO2009
Central Unilever Tea Hospital, Kericho, Kenya
RCP8.5
RCP4.5
RCP2.6
2030s
2050s
2070s
2090s
LN(Malaria cases/Mean=106.1)
Apr1995
May2006
Jul2017
Aug2028
Oct2039
Nov2050
Jan2062
Feb2073
Apr2084
May2095
Date
Actual
Smoothed
Predicted
95% Confidence limits
Start of multi-step forecasts (2006)

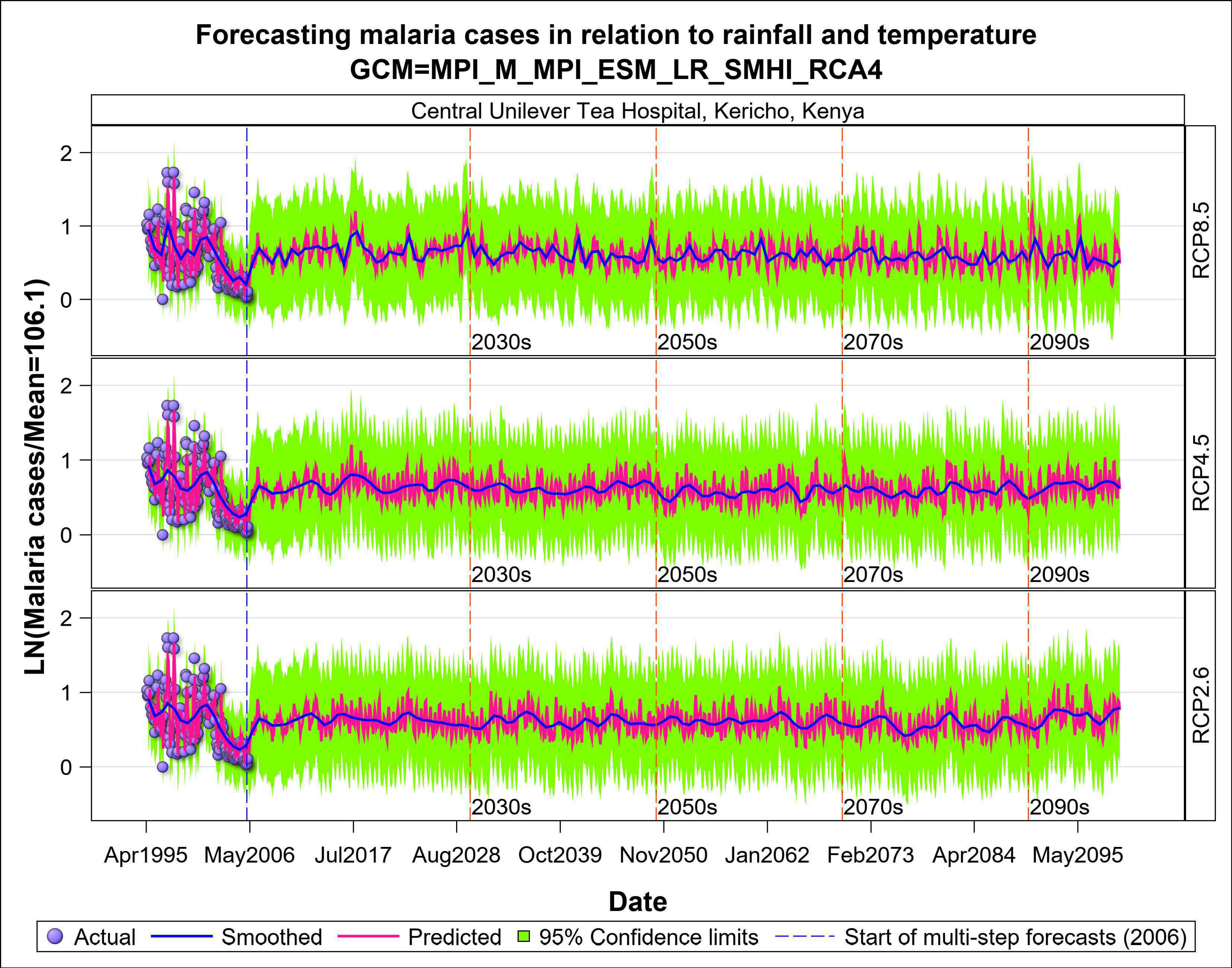
Forecasting malaria cases in relation to rainfall and temperature
GCM=MPI_M_MPI_ESM_LR_SMHI_RCA4
Central Unilever Tea Hospital, Kericho, Kenya
RCP8.5
RCP4.5
RCP2.6
2030s
2050s
2070s
2090s
LN(Malaria cases/Mean=106.1)
0
1
2
Apr1995
May2006
Jul2017
Aug2028
Oct2039
Nov2050
Jan2062
Feb2073
Apr2084
May2095
Date
Actual
Smoothed
Predicted
95% Confidence limits
Start of multi-step forecasts (2006)

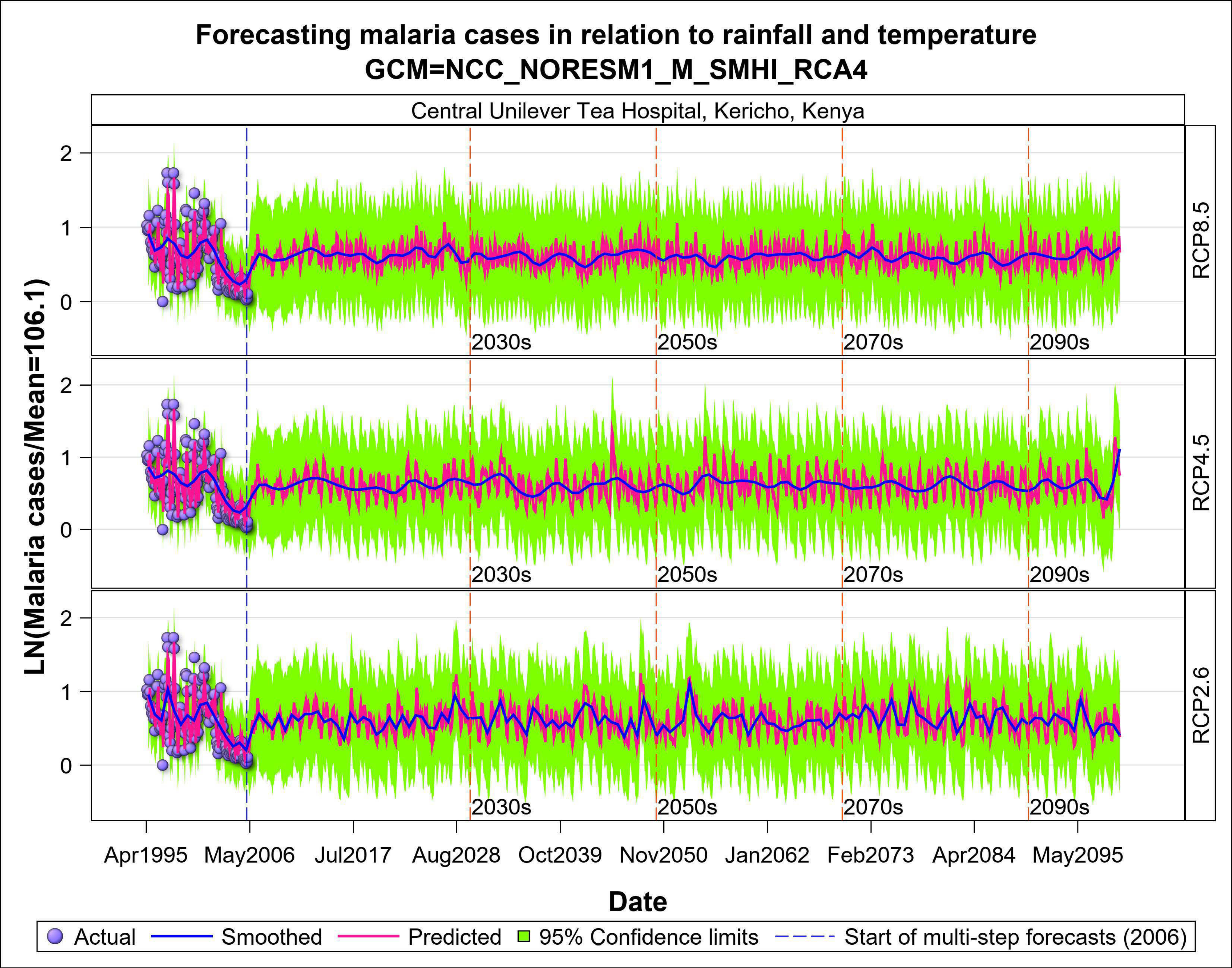
Forecasting malaria cases in relation to rainfall and temperature
GCM=NCC_NORESM1_M_SMHI_RCA4
Central Unilever Tea Hospital, Kericho, Kenya
RCP8.5
RCP4.5
RCP2.6
2030s
2050s
2070s
2090s
LN(Malaria cases/Mean=106.1)
2
1
0
Apr1995
May2006
Jul2017
Aug2028
Oct2039
Nov2050
Jan2062
Feb2073
Apr2084
May2095
Date
Actual
Smoothed
Predicted
95% Confidence limits
Start of multi-step forecasts (2006)

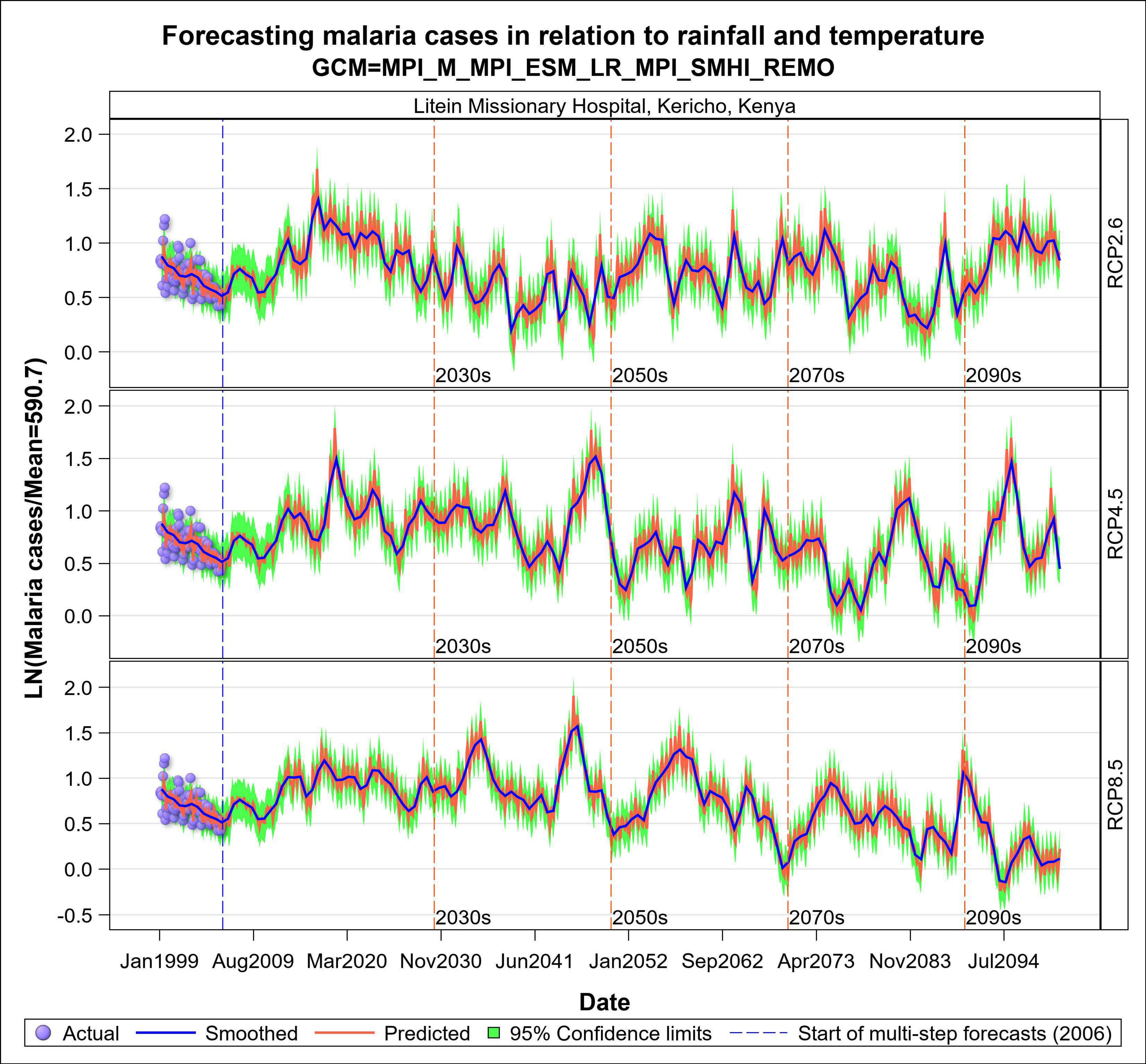

Forecasting malaria cases in relation to rainfall and temperature
GCM=MPI_M_MPI_ESM_LR_MPI_SMHI_REMO
Litein Missionary Hospital, Kericho, Kenya
LN(Malaria cases/Mean=590.7)
RCP2.6
RCP4.5
RCP8.5
2030s
2050s
2070s
2090s
Jan1999
Aug2009
Mar2020
Nov2030
Jun2041
Jan2052
Sep2062
Apr2073
Nov2083
Jul2094
Date
Actual
Smoothed
Predicted
95% Confidence limits
Start of multi-step forecasts (2006)

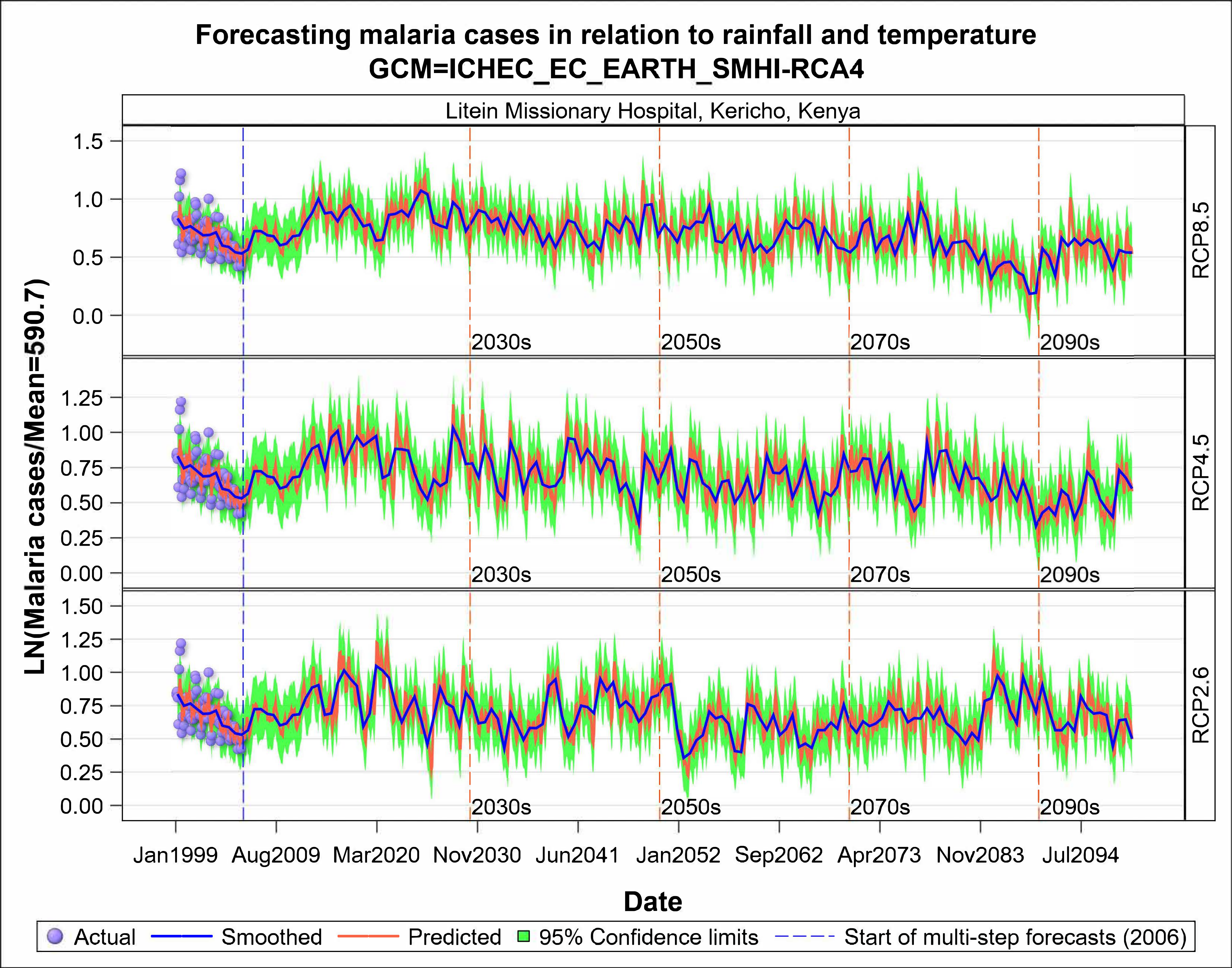

Forecasting malaria cases in relation to rainfall and temperature
GCM=ICHEC_EC_EARTH_SMHI-RCA4
Litein Missionary Hospital, Kericho, Kenya
RCP8.5
RCP4.5
RCP2.6
2030s
2050s
2070s
2090s
LN(Malaria cases/Mean=590.7)
Jan1999
Aug2009
Mar2020
Nov2030
Jun2041
Jan2052
Sep2062
Apr2073
Nov2083
Jul2094
Date
Actual
Smoothed
Predicted
95% Confidence limits
Start of multi-step forecasts (2006)

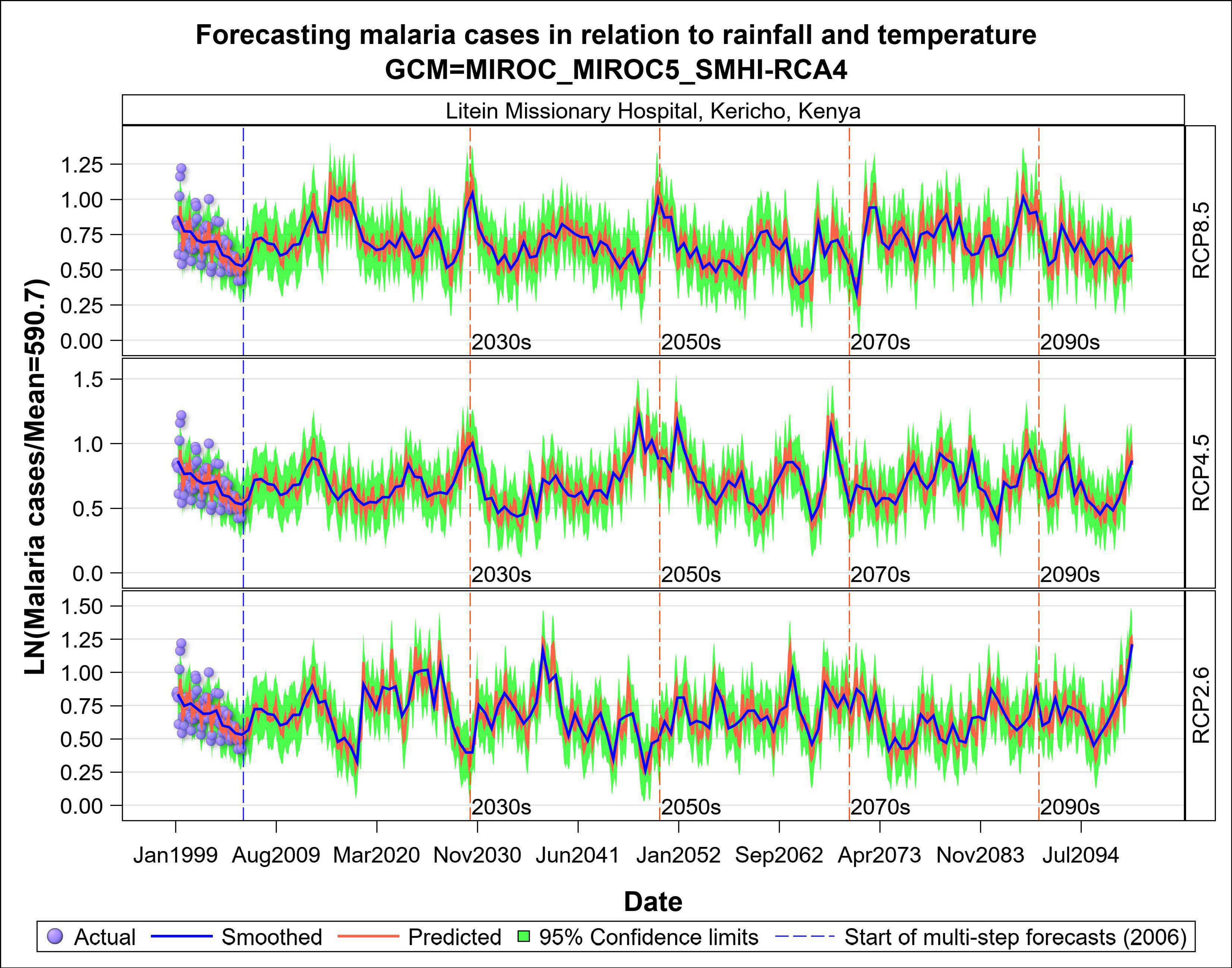
Forecasting malaria cases in relation to rainfall and temperature
GCM=MIROC_MIROC5_SMHI-RCA4
Litein Missionary Hospital, Kericho, Kenya
RCP8.5
RCP4.5
RCP2.6
2030s
2050s
2070s
2090s
LN(Malaria cases/Mean=590.7)
1.25
1.00
0.75
0.50
0.25
0.00
1.5
1.0
0.5
0.0
1.50
Jan1999
Aug2009
Mar2020
Nov2030
Jun2041
Jan2052
Sep2062
Apr2073
Nov2083
Jul2094
Date
Actual
Smoothed
Predicted
95% Confidence limits
Start of multi-step forecasts (2006)

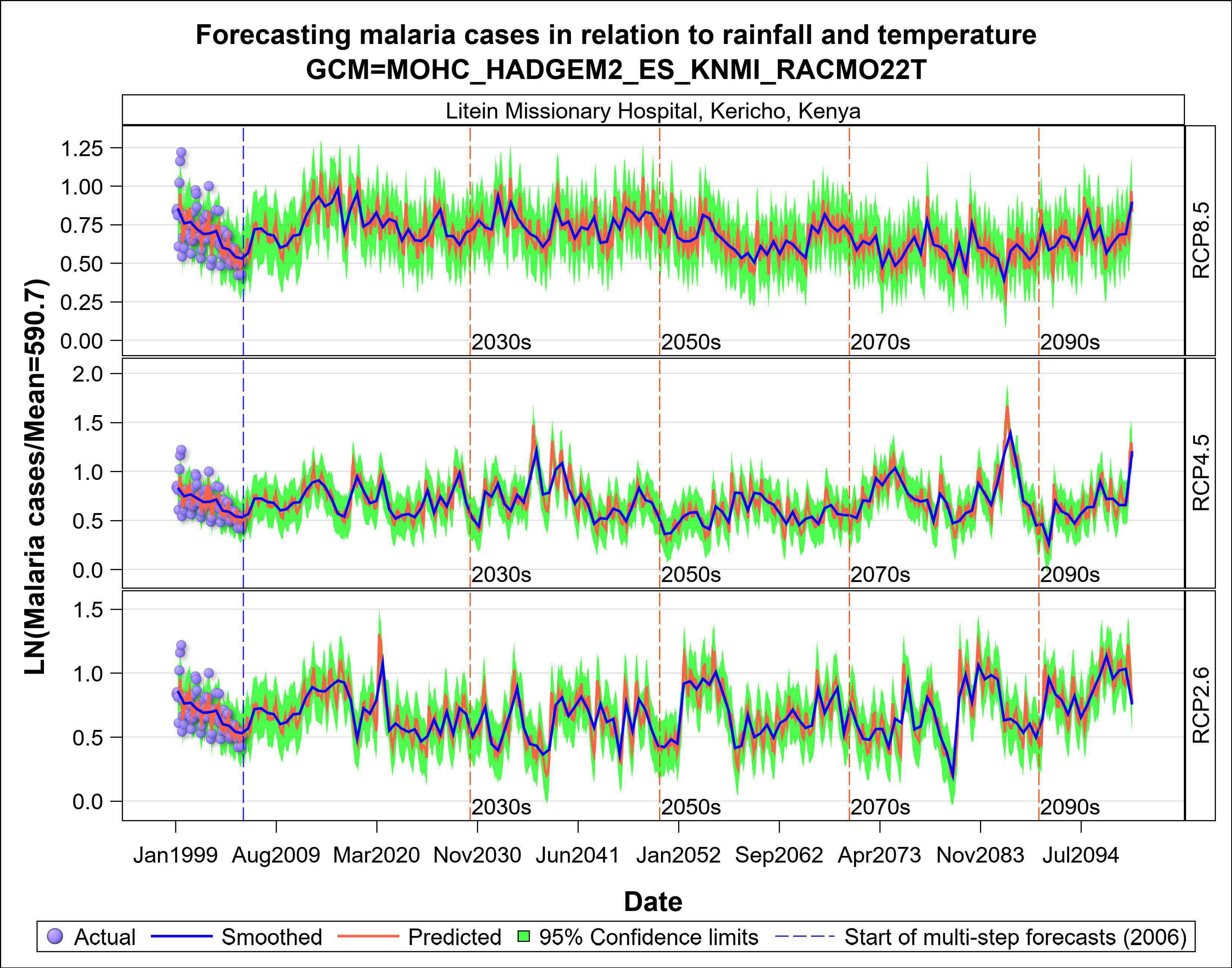

Forecasting malaria cases in relation to rainfall and temperature
GCM=MOHC_HADGEM2_ES_KNMI_RACMO22T
Litein Missionary Hospital, Kericho, Kenya
LN(Malaria cases/Mean=590.7)
RCP8.5
RCP4.5
RCP2.6
2030s
2050s
2070s
2090s
Jan1999
Aug2009
Mar2020
Nov2030
Jun2041
Jan2052
Sep2062
Apr2073
Nov2083
Jul2094
Date
Actual
Smoothed
Predicted
95% Confidence limits
Start of multi-step forecasts (2006)

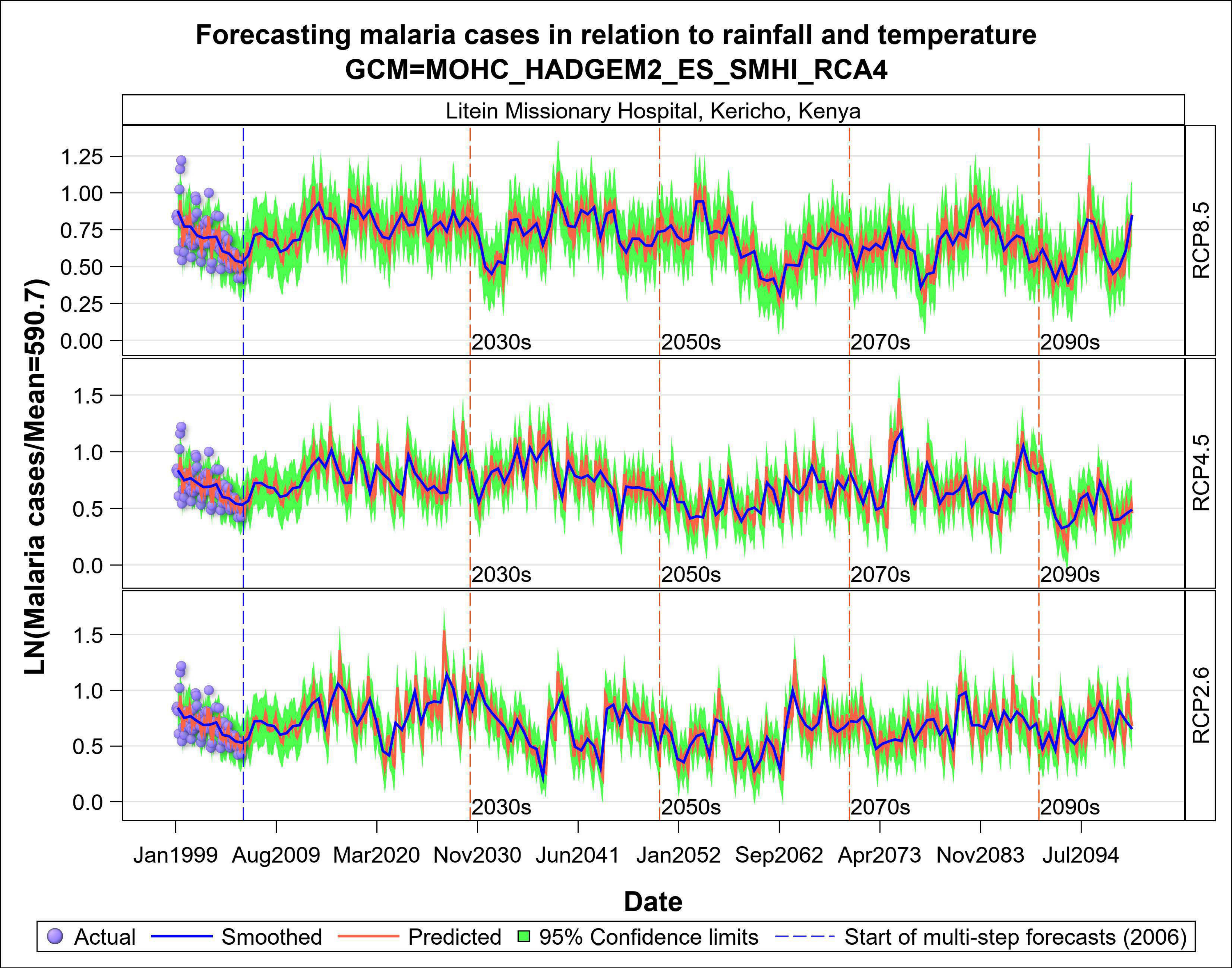

Forecasting malaria cases in relation to rainfall and temperature
GCM=MOHC_HADGEM2_ES_SMHI_RCA4
Litein Missionary Hospital, Kericho, Kenya
RCP8.5
RCP4.5
RCP2.6
LN(Malaria cases/Mean=590.7)
1.25
1.00
0.75
0.50
0.25
0.00
1.5
1.0
0.5
0.0
2030s
2050s
2070s
2090s
Jan1999
Aug2009
Mar2020
Nov2030
Jun2041
Jan2052
Sep2062
Apr2073
Nov2083
Jul2094
Date
Actual
Smoothed
Predicted
95% Confidence limits
Start of multi-step forecasts (2006)

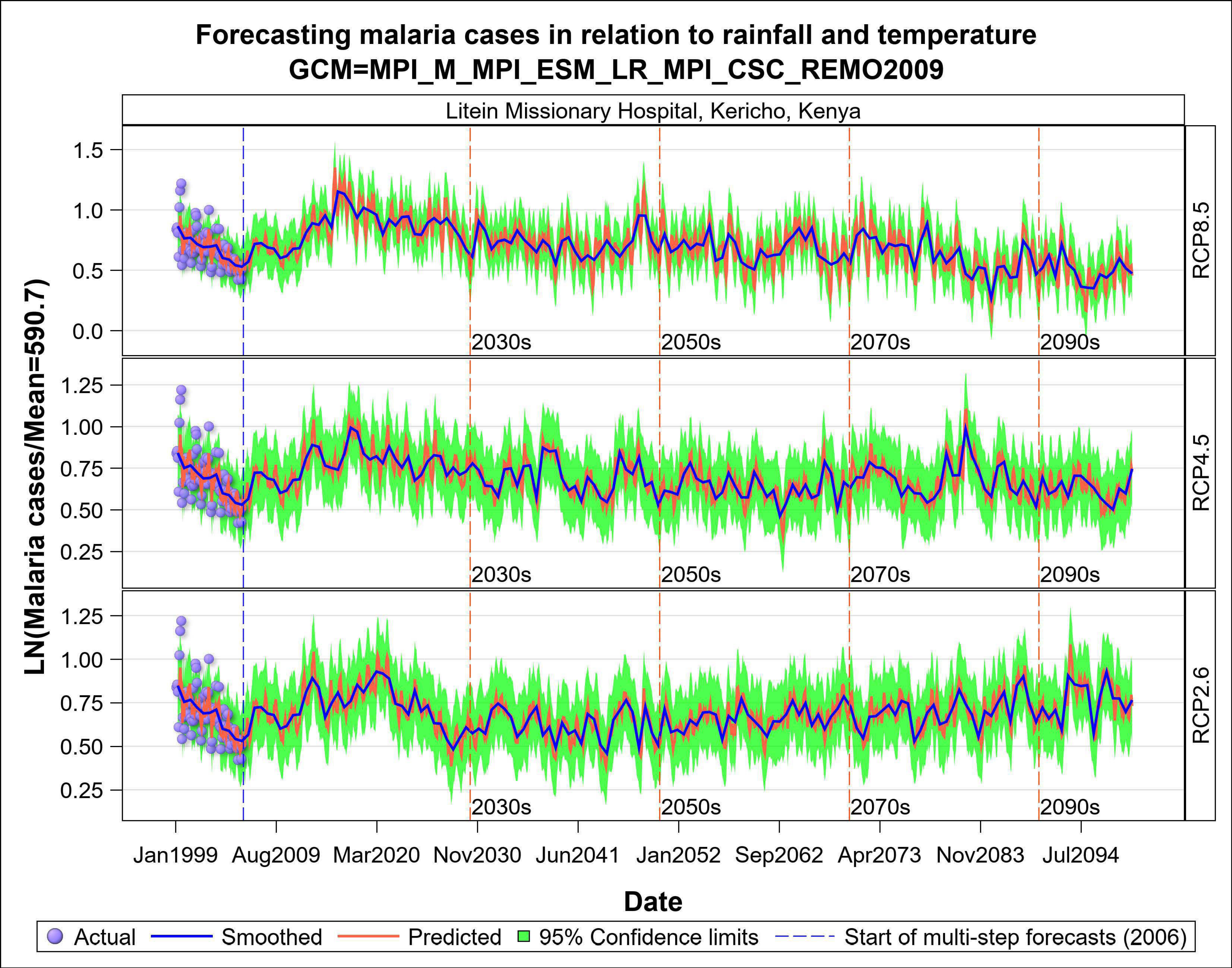

Forecasting malaria cases in relation to rainfall and temperature
GCM=MPI_M_MPI_ESM_LR_MPI_CSC_REMO2009
Litein Missionary Hospital, Kericho, Kenya
RCP8.5
RCP4.5
RCP2.6
2030s
2050s
2070s
2090s
LN(Malaria cases/Mean=590.7)
1.5
1.0
0.5
0.0
1.25
1.00
0.75
0.50
0.25
Jan1999
Aug2009
Mar2020
Nov2030
Jun2041
Jan2052
Sep2062
Apr2073
Nov2083
Jul2094
Date
Actual
Smoothed
Predicted
95% Confidence limits
Start of multi-step forecasts (2006)

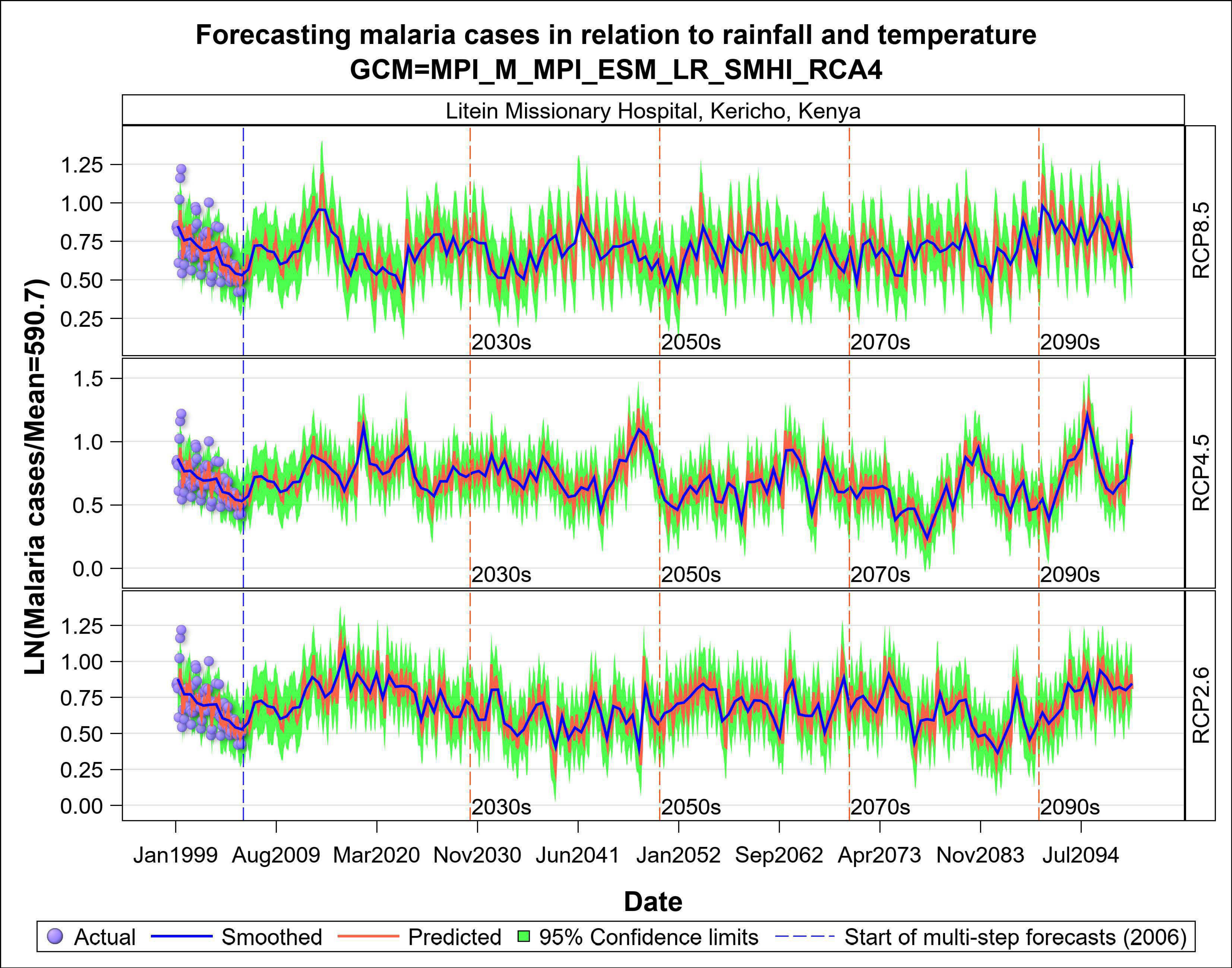
Forecasting malaria cases in relation to rainfall and temperature
GCM=MPI_M_MPI_ESM_LR_SMHI_RCA4
Litein Missionary Hospital, Kericho, Kenya
RCP8.5
RCP4.5
RCP2.6
2030s
2050s
2070s
2090s
LN(Malaria cases/Mean=590.7)
1.25
1.00
0.75
0.50
0.25
1.5
1.0
0.5
0.0
0.00
Jan1999
Aug2009
Mar2020
Nov2030
Jun2041
Jan2052
Sep2062
Apr2073
Nov2083
Jul2094
Date
Actual
Smoothed
Predicted
95% Confidence limits
Start of multi-step forecasts (2006)

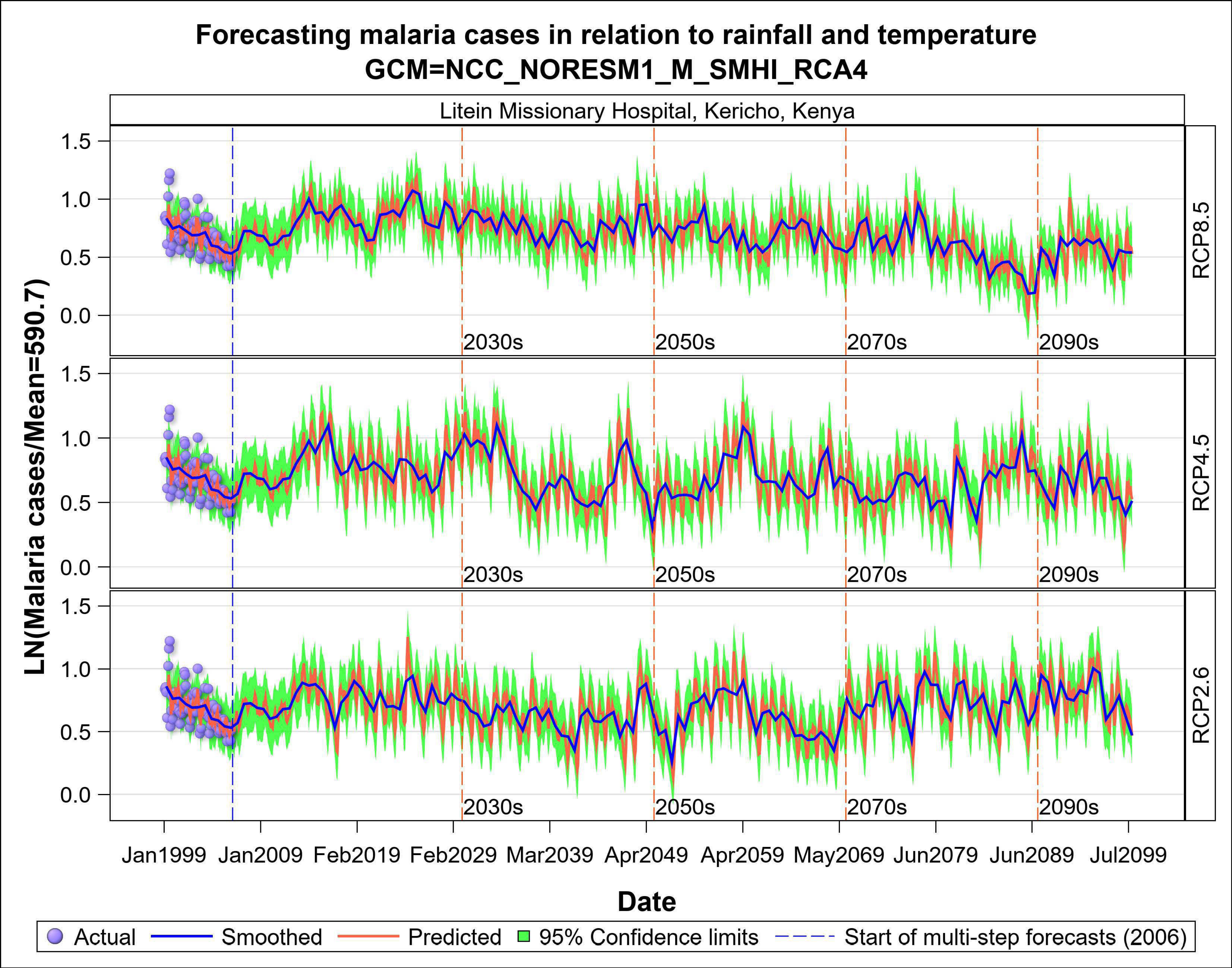

Forecasting malaria cases in relation to rainfall and temperature
GCM=NCC_NORESM1_M_SMHI_RCA4
Litein Missionary Hospital, Kericho, Kenya
LN(Malaria cases/Mean=590.7)
1.5
1.0
0.5
0.0
2030s
2050s
2070s
2090s
RCP8.5
RCP4.5
RCP2.6
Jan1999
Jan2009
Feb2019
Feb2029
Mar2039
Apr2049
Apr2059
May2069
Jun2079
Jun2089
Jul2099
Date
Actual
Smoothed
Predicted
95% Confidence limits
Start of multi-step forecasts (2006)

SI17

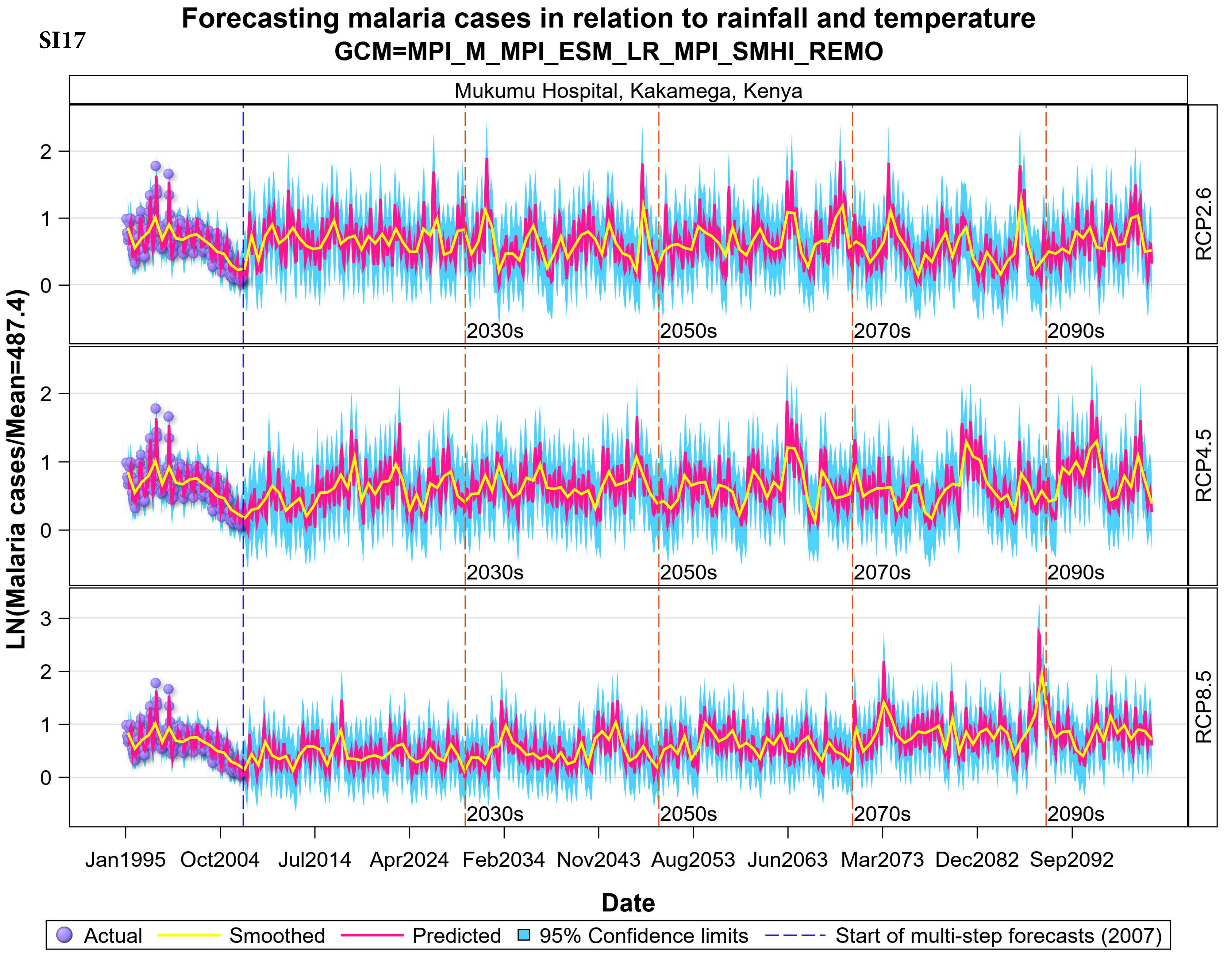
Forecasting malaria cases in relation to rainfall and temperature
GCM=MPI_M_MPI_ESM_LR_MPI_SMHI_REMO
Mukumu Hospital, Kakamega, Kenya
LN(Malaria cases/Mean=487.4)
RCP2.6
RCP4.5
RCP8.5
2030s
2050s
2070s
2090s
Jan1995
Oct2004
Jul2014
Apr2024
Feb2034
Nov2043
Aug2053
Jun2063
Mar2073
Dec2082
Sep2092
Date
Actual
Smoothed
Predicted
95% Confidence limits
Start of multi-step forecasts (2007)

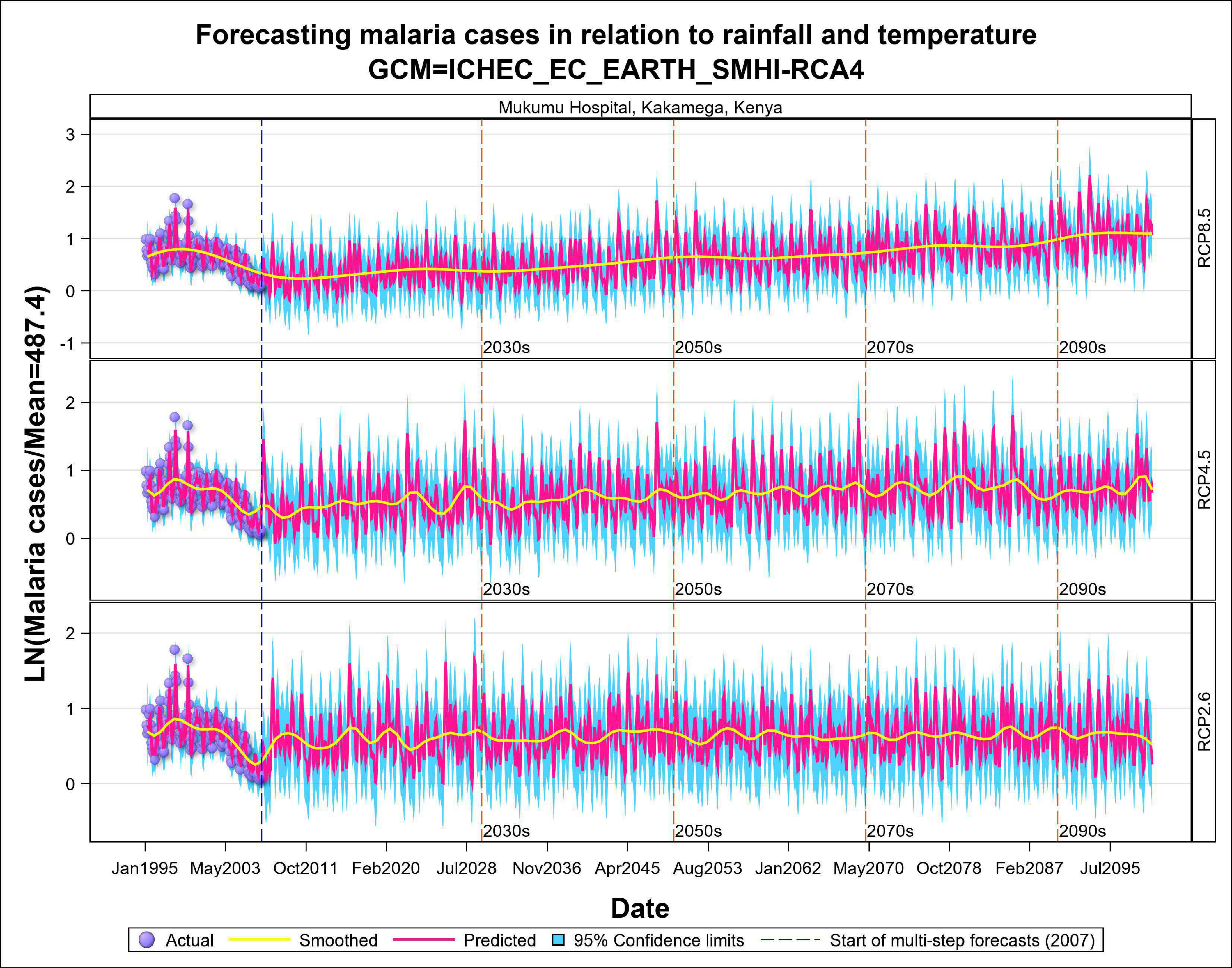

Forecasting malaria cases in relation to rainfall and temperature
GCM=ICHEC_EC_EARTH_SMHI-RCA4
Mukumu Hospital, Kakamega, Kenya
LN(Malaria cases/Mean=487.4)
RCP8.5
RCP4.5
RCP2.6
2030s
2050s
2070s
2090s
Jan1995
May2003
Oct2011
Feb2020
Jul2028
Nov2036
Apr2045
Aug2053
Jan2062
May2070
Oct2078
Feb2087
Jul2095
Date
Actual
Smoothed
Predicted
95% Confidence limits
Start of multi-step forecasts (2007)

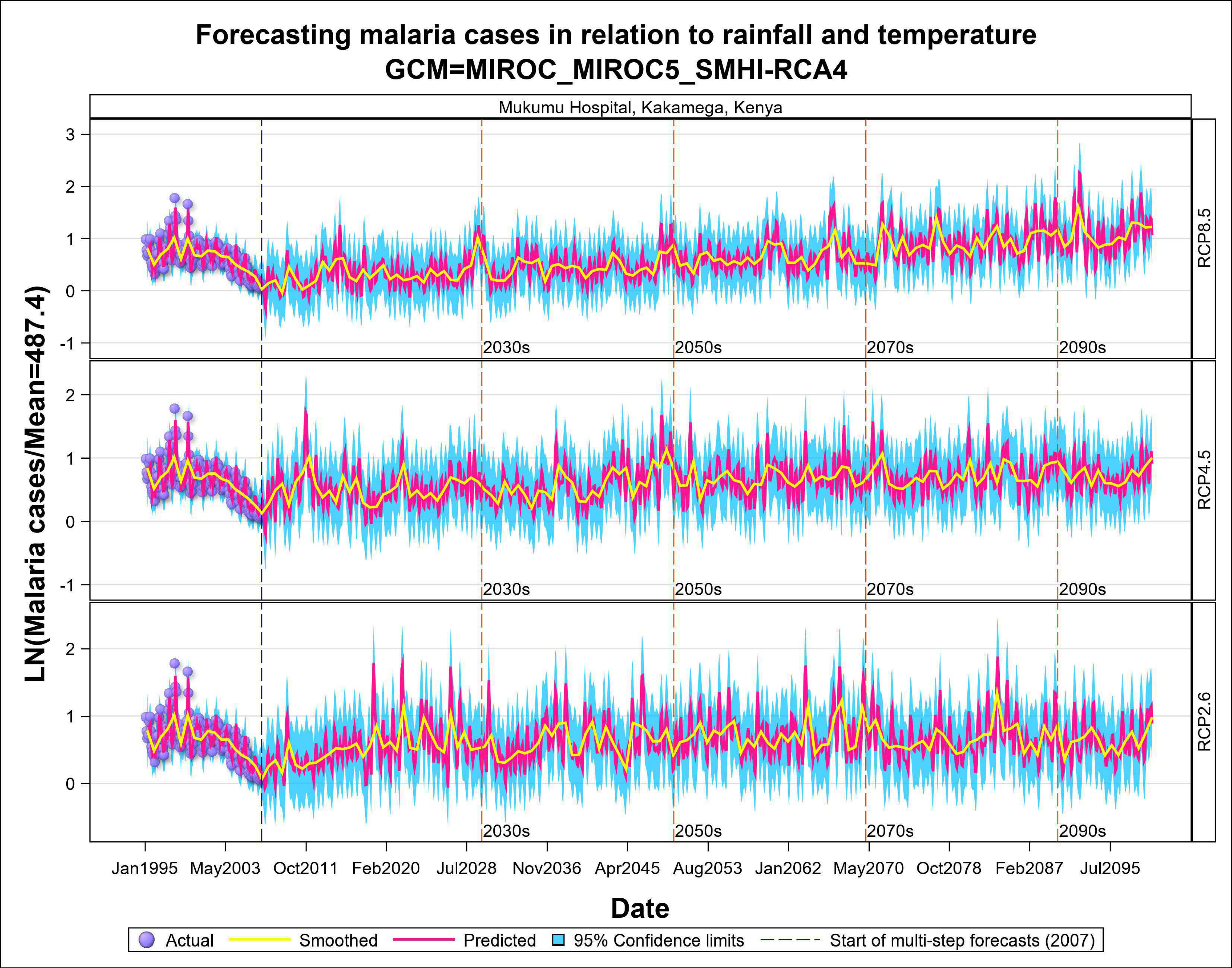

Forecasting malaria cases in relation to rainfall and temperature
GCM=MIROC_MIROC5_SMHI-RCA4
Mukumu Hospital, Kakamega, Kenya
RCP8.5
RCP4.5
RCP2.6
2030s
2050s
2070s
2090s
LN(Malaria cases/Mean=487.4)
Date
Jan1995
May2003
Oct2011
Feb2020
Jul2028
Nov2036
Apr2045
Aug2053
Jan2062
May2070
Oct2078
Feb2087
Jul2095
Actual
Smoothed
Predicted
95% Confidence limits
Start of multi-step forecasts (2007)

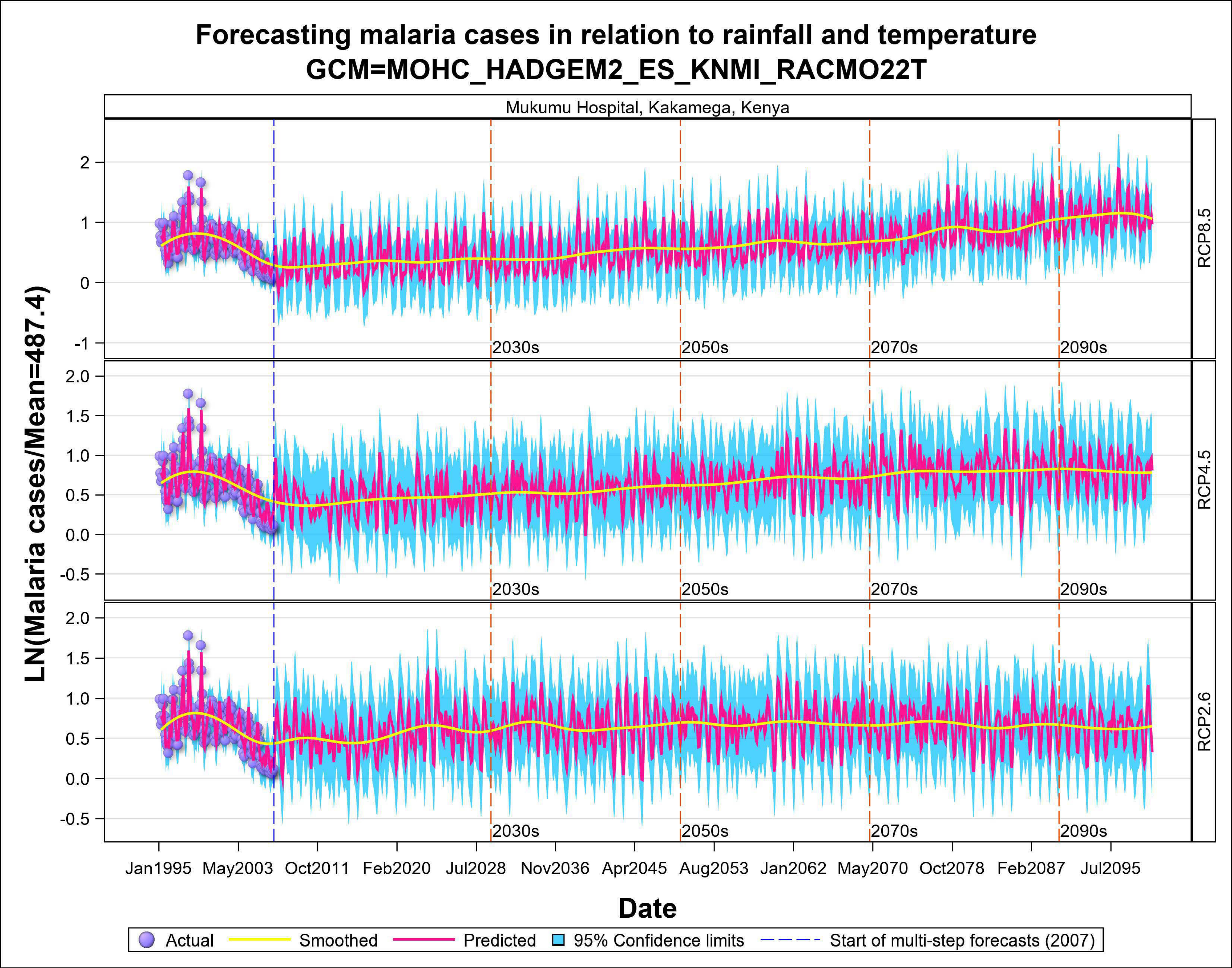

Forecasting malaria cases in relation to rainfall and temperature
GCM=MOHC_HADGEM2_ES_KNMI_RACMO22T
Mukumu Hospital, Kakamega, Kenya
RCP8.5
RCP4.5
RCP2.6
2030s
2050s
2070s
2090s
LN(Malaria cases/Mean=487.4)
Jan1995
May2003
Oct2011
Feb2020
Jul2028
Nov2036
Apr2045
Aug2053
Jan2062
May2070
Oct2078
Feb2087
Jul2095
Date
Actual
Smoothed
Predicted
95% Confidence limits
Start of multi-step forecasts (2007)

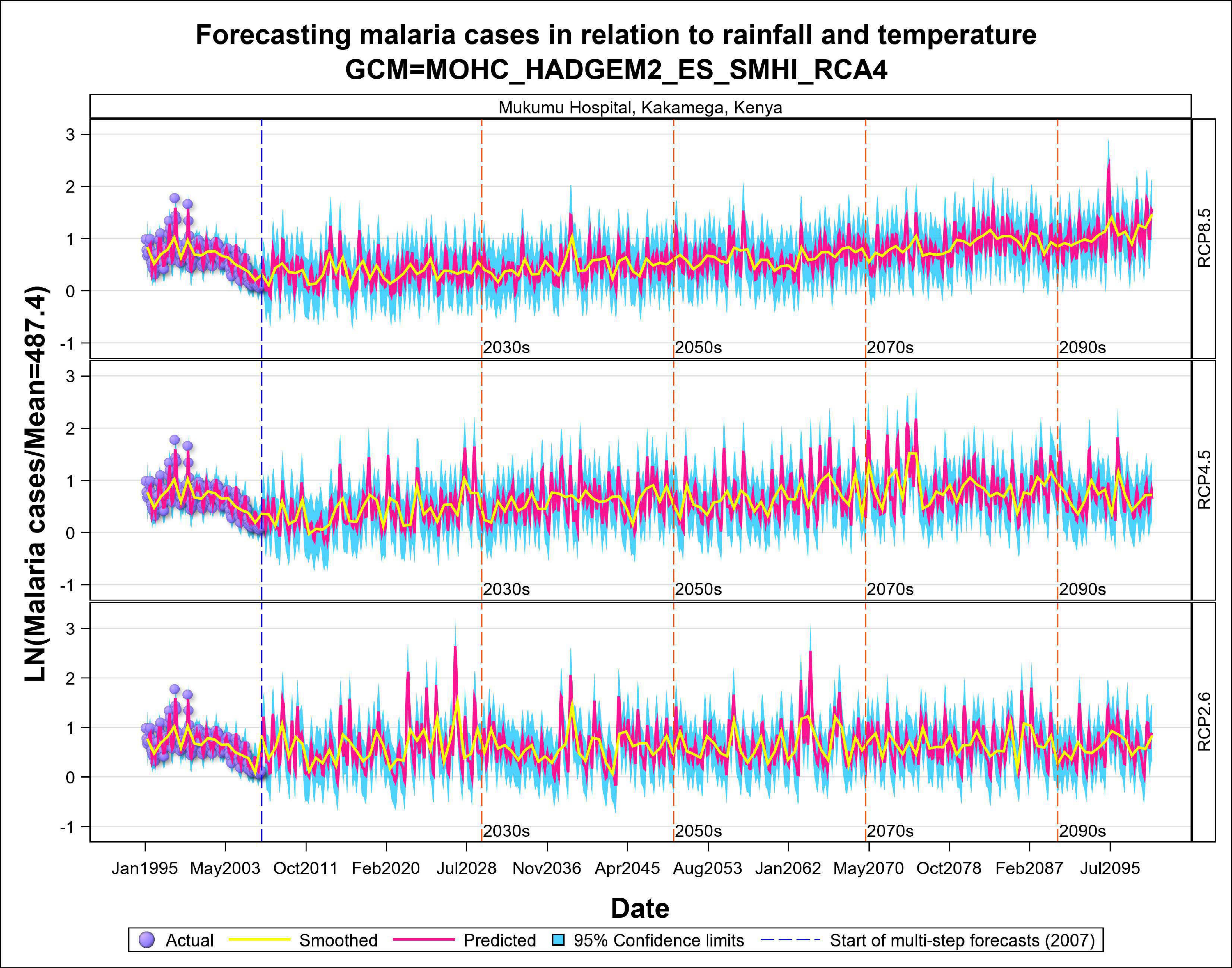
Forecasting malaria cases in relation to rainfall and temperature
GCM=MOHC_HADGEM2_ES_SMHI_RCA4
Mukumu Hospital, Kakamega, Kenya
RCP8.5
RCP4.5
RCP2.6
2030s
2050s
2070s
2090s
LN(Malaria cases/Mean=487.4)
3
2
1
0
-1
Jan1995
May2003
Oct2011
Feb2020
Jul2028
Nov2036
Apr2045
Aug2053
Jan2062
May2070
Oct2078
Feb2087
Jul2095
Date
Actual
Smoothed
Predicted
95% Confidence limits
Start of multi-step forecasts (2007)

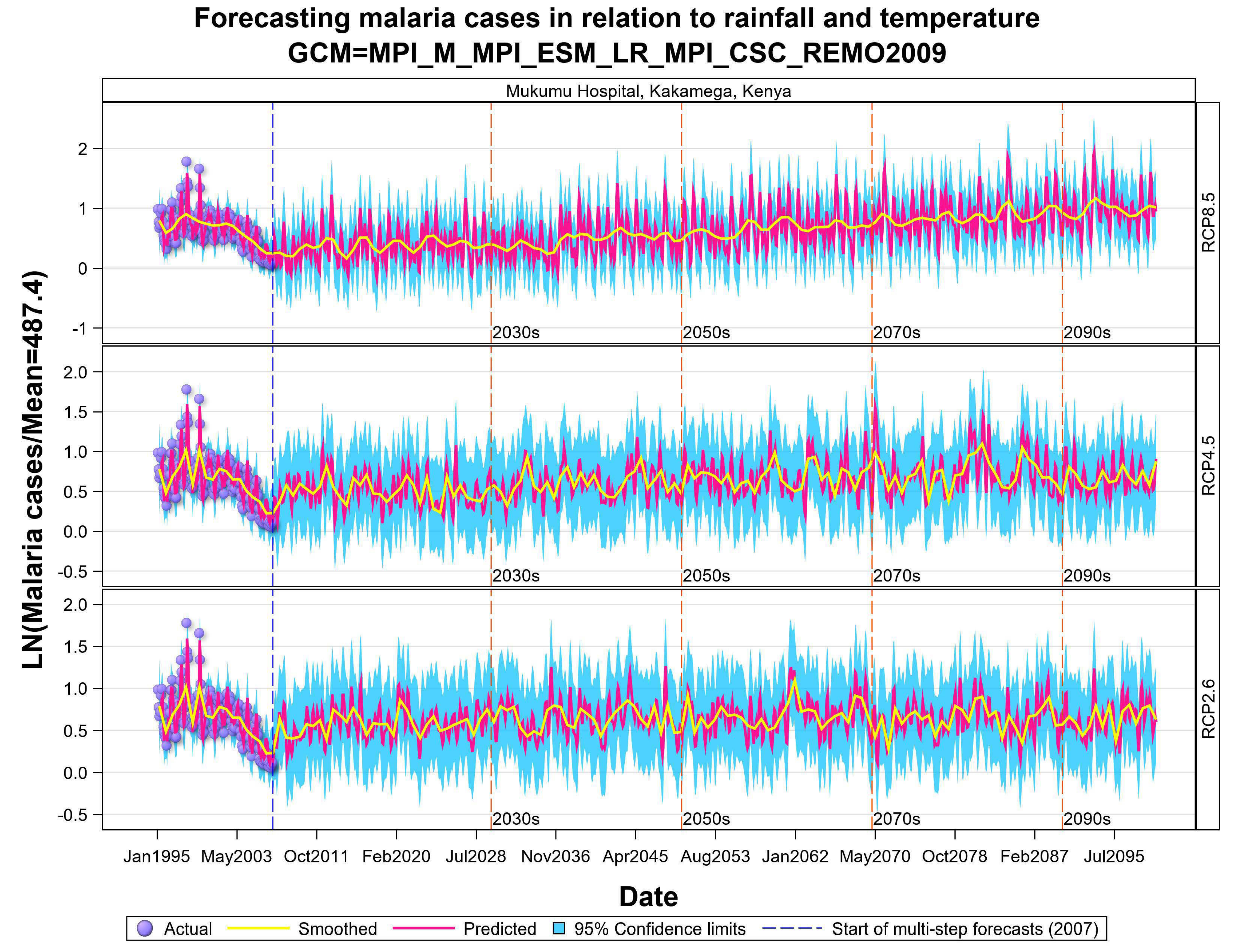

Forecasting malaria cases in relation to rainfall and temperature
GCM=MPI_M_MPI_ESM_LR_MPI_CSC_REMO2009
Mukumu Hospital, Kakamega, Kenya
RCP8.5
RCP4.5
RCP2.6
2030s
2050s
2070s
2090s
LN(Malaria cases/Mean=487.4)
Jan1995
May2003
Oct2011
Feb2020
Jul2028
Nov2036
Apr2045
Aug2053
Jan2062
May2070
Oct2078
Feb2087
Jul2095
Date
Actual
Smoothed
Predicted
95% Confidence limits
Start of multi-step forecasts (2007)

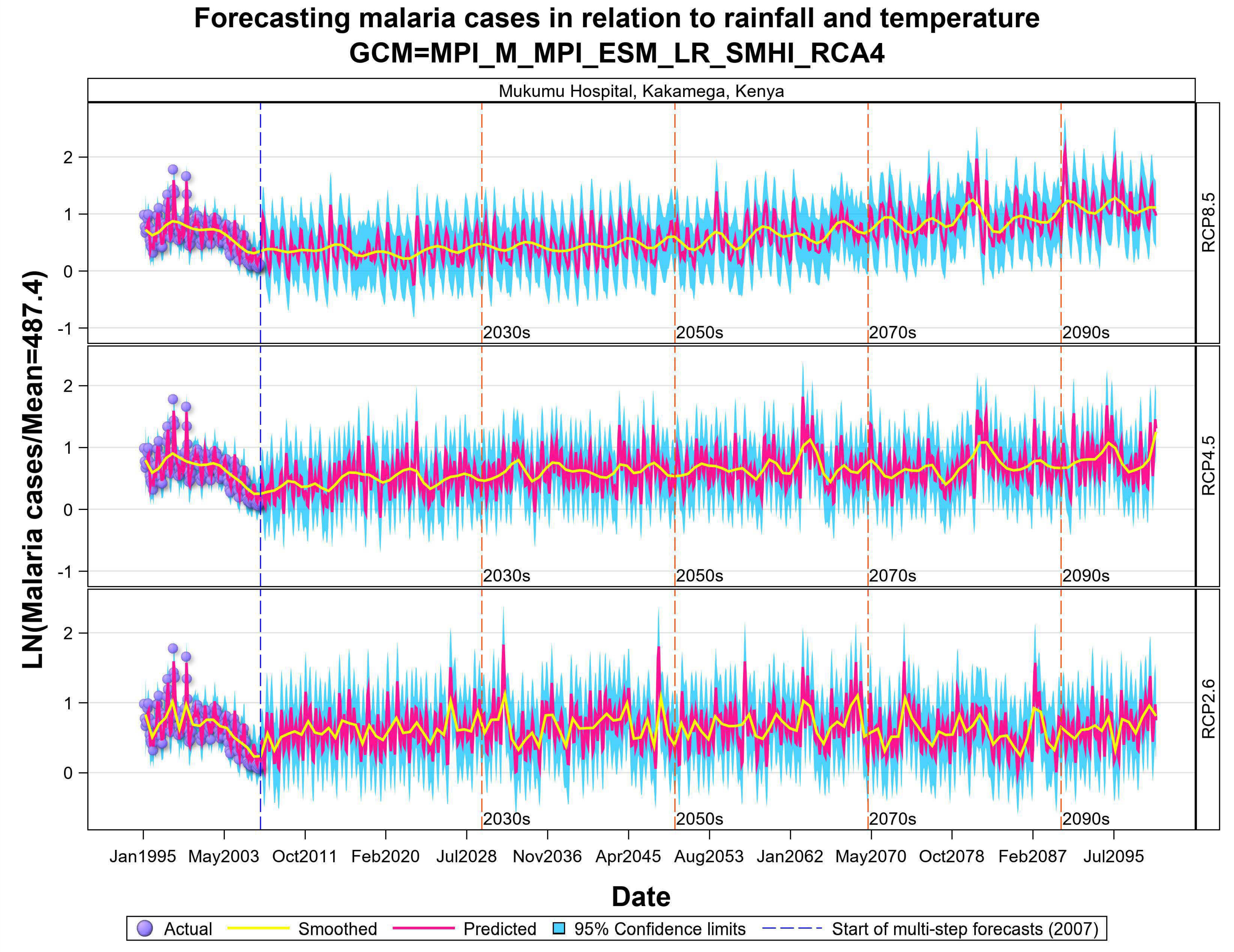

Forecasting malaria cases in relation to rainfall and temperature
GCM=MPI_M_MPI_ESM_LR_SMHI_RCA4
Mukumu Hospital, Kakamega, Kenya
LN(Malaria cases/Mean=487.4)
RCP8.5
RCP4.5
RCP2.6
2030s
2050s
2070s
2090s
Jan1995
May2003
Oct2011
Feb2020
Jul2028
Nov2036
Apr2045
Aug2053
Jan2062
May2070
Oct2078
Feb2087
Jul2095
Date
Actual
Smoothed
Predicted
95% Confidence limits
Start of multi-step forecasts (2007)

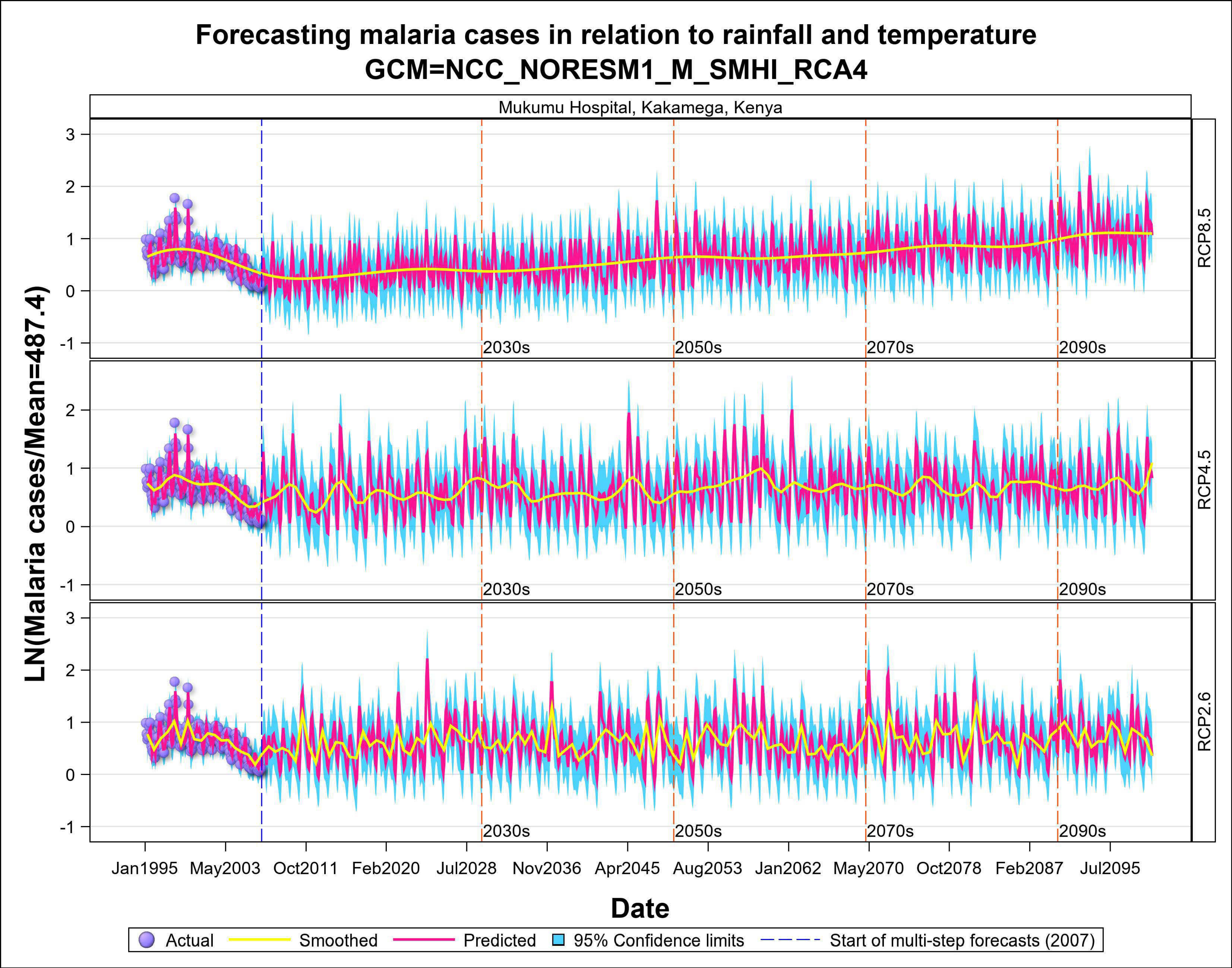
Forecasting malaria cases in relation to rainfall and temperature
GCM=NCC_NORESM1_M_SMHI_RCA4
Mukumu Hospital, Kakamega, Kenya
LN(Malaria cases/Mean=487.4)
RCP8.5
RCP4.5
RCP2.6
2030s
2050s
2070s
2090s
Jan1995
May2003
Oct2011
Feb2020
Jul2028
Nov2036
Apr2045
Aug2053
Jan2062
May2070
Oct2078
Feb2087
Jul2095
Date
Actual
Smoothed
Predicted
95% Confidence limits
Start of multi-step forecasts (2007)

SI18

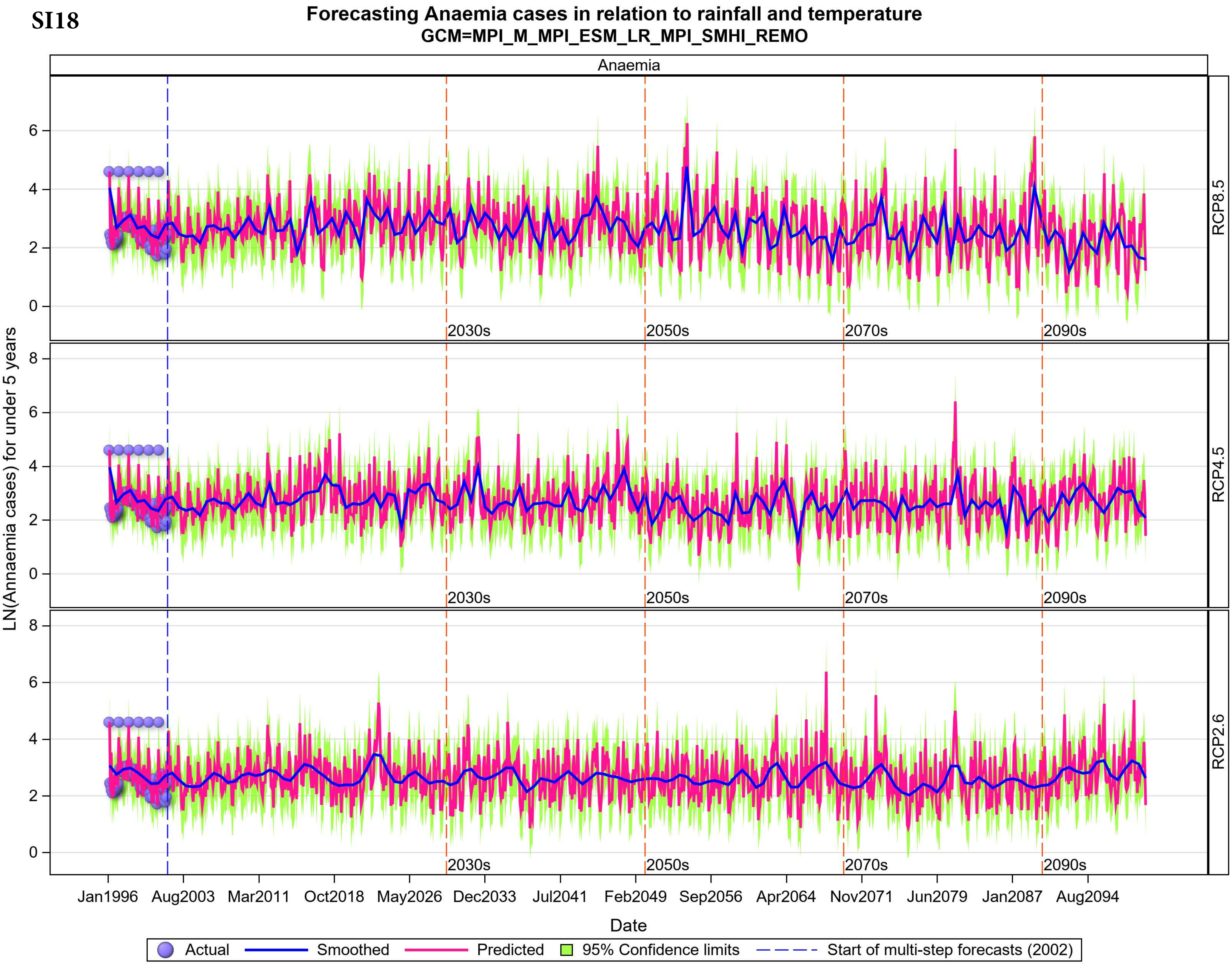

Forecasting Anaemia cases in relation to rainfall and temperature
GCM=MPI_M_MPI_ESM_LR_MPI_SMHI_REMO
Anaemia
RCP8.5
RCP4.5
RCP2.6
2030s
2050s
2070s
2090s
LN(Annaemia cases) for under 5 years
Jan1996
Aug2003
Mar2011
Oct2018
May2026
Dec2033
Jul2041
Feb2049
Sep2056
Apr2064
Nov2071
Jun2079
Jan2087
Aug2094
Date
Actual
Smoothed
Predicted
95% Confidence limits
Start of multi-step forecasts (2002)

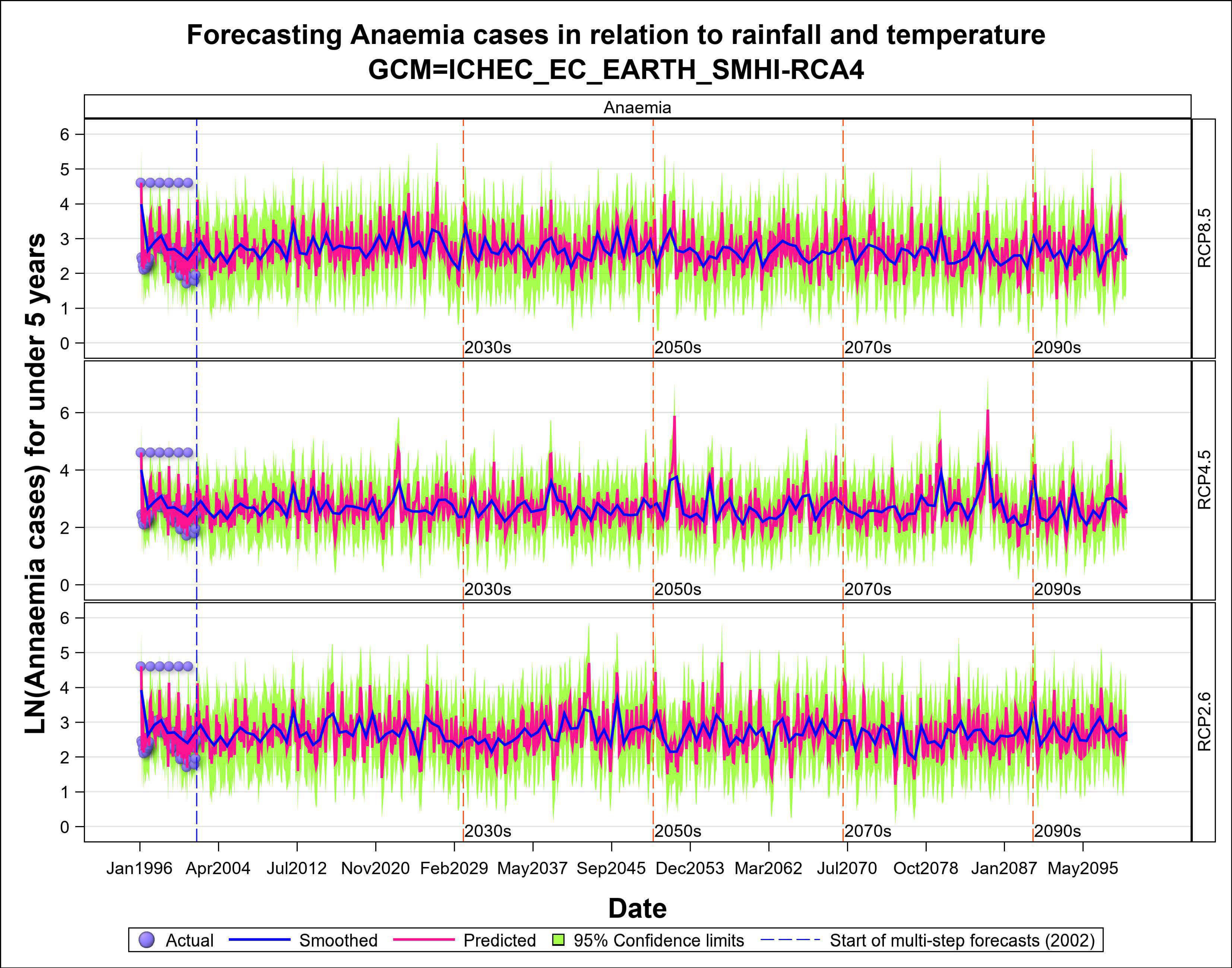

Forecasting Anaemia cases in relation to rainfall and temperature
GCM=ICHEC_EC_EARTH_SMHI-RCA4
Anaemia
LN(Annaemia cases) for under 5 years
RCP8.5
RCP4.5
RCP2.6
2030s
2050s
2070s
2090s
Jan1996
Apr2004
Jul2012
Nov2020
Feb2029
May2037
Sep2045
Dec2053
Mar2062
Jul2070
Oct2078
Jan2087
May2095
Date
Actual
Smoothed
Predicted
95% Confidence limits
Start of multi-step forecasts (2002)

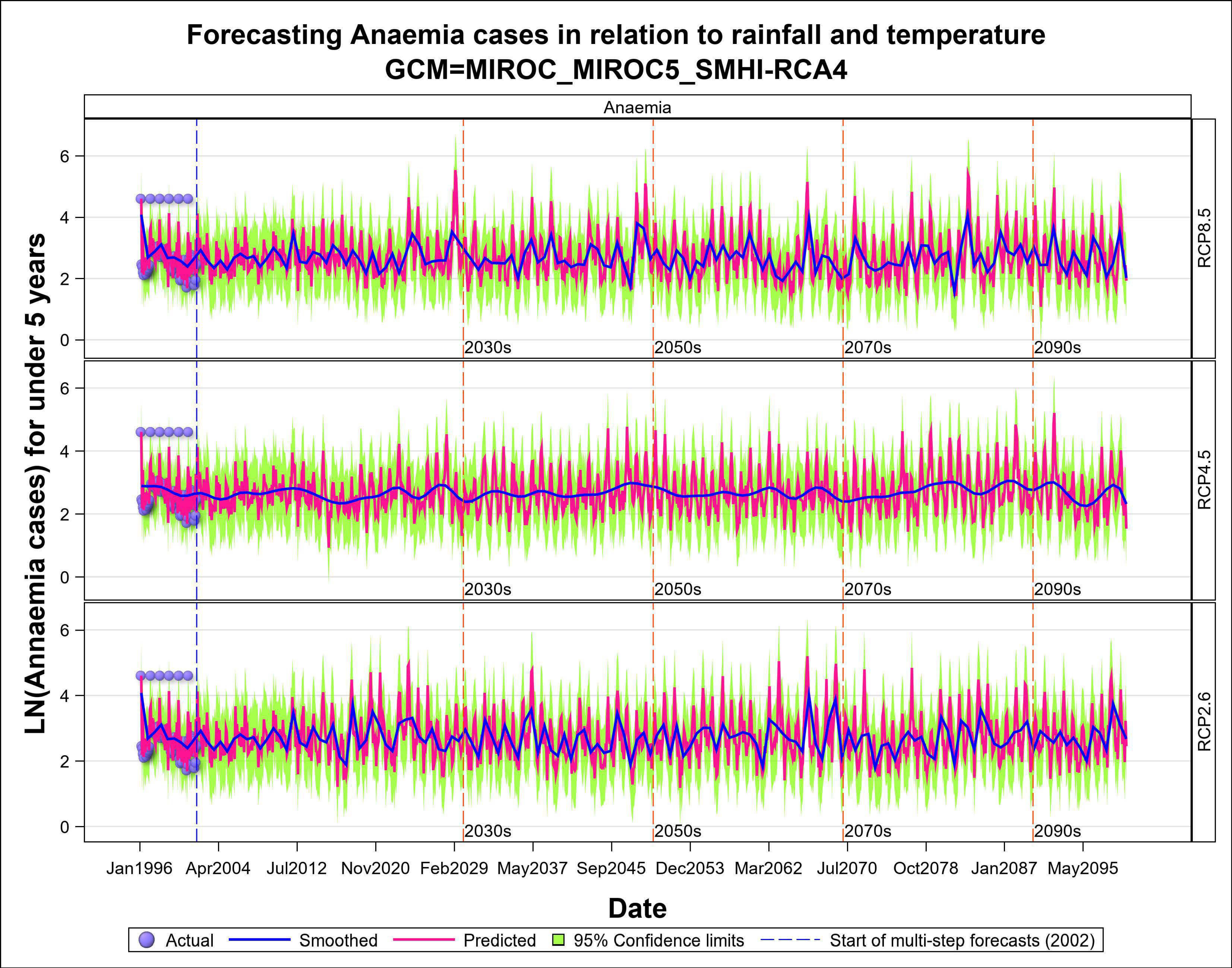

Forecasting Anaemia cases in relation to rainfall and temperature
GCM=MIROC_MIROC5_SMHI-RCA4
Anaemia
RCP8.5
RCP4.5
RCP2.6
2030s
2050s
2070s
2090s
0
2
4
6
LN(Annaemia cases) for under 5 years
Jan1996
Apr2004
Jul2012
Nov2020
Feb2029
May2037
Sep2045
Dec2053
Mar2062
Jul2070
Oct2078
Jan2087
May2095
Date
Actual
Smoothed
Predicted
95% Confidence limits
Start of multi-step forecasts (2002)

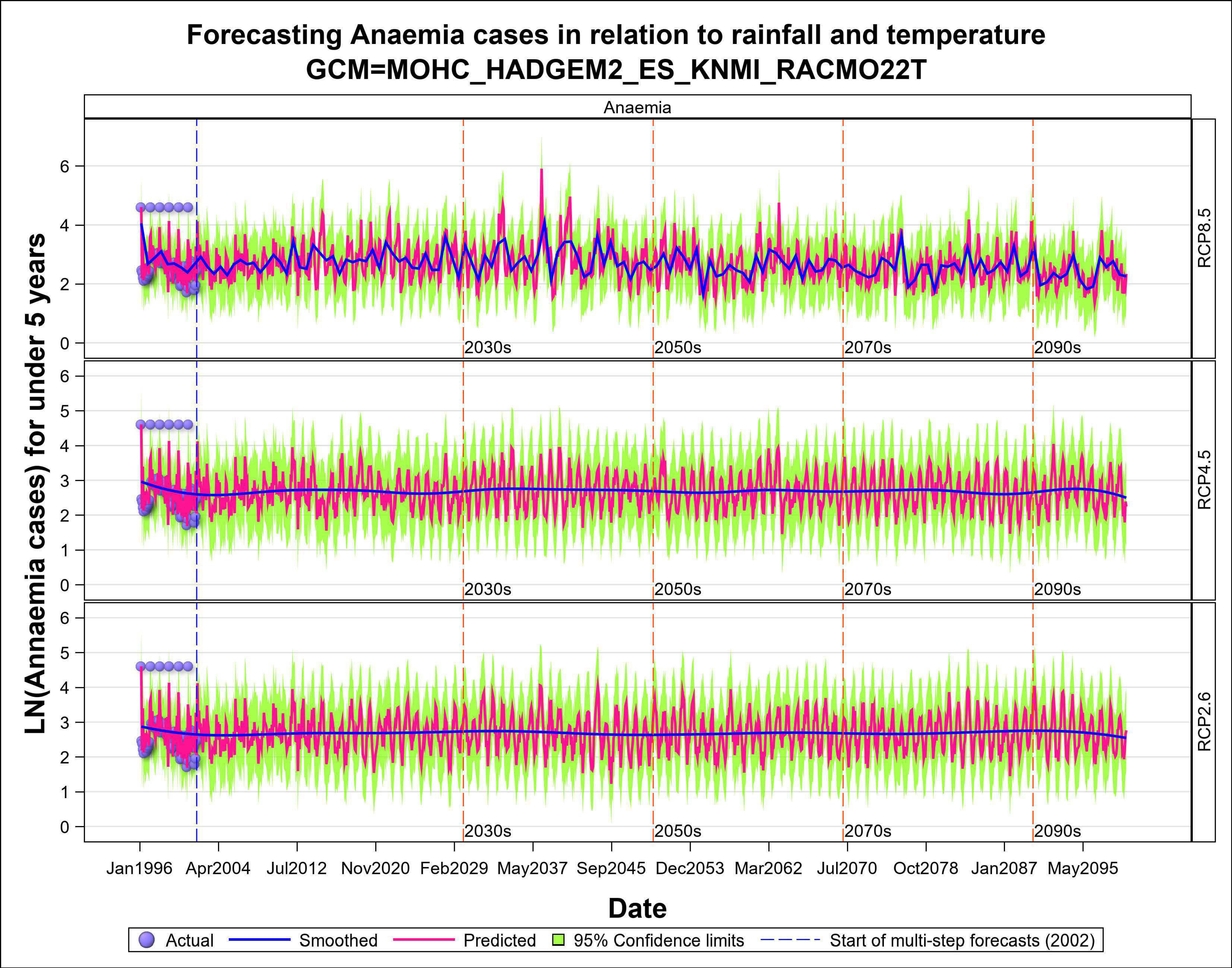

Forecasting Anaemia cases in relation to rainfall and temperature
GCM=MOHC_HADGEM2_ES_KNMI_RACMO22T
Anaemia
RCP8.5
RCP4.5
RCP2.6
2030s
2050s
2070s
2090s
LN(Annaemia cases) for under 5 years
0
1
2
3
4
5
6
Jan1996
Apr2004
Jul2012
Nov2020
Feb2029
May2037
Sep2045
Dec2053
Mar2062
Jul2070
Oct2078
Jan2087
May2095
Date
Actual
Smoothed
Predicted
95% Confidence limits
Start of multi-step forecasts (2002)

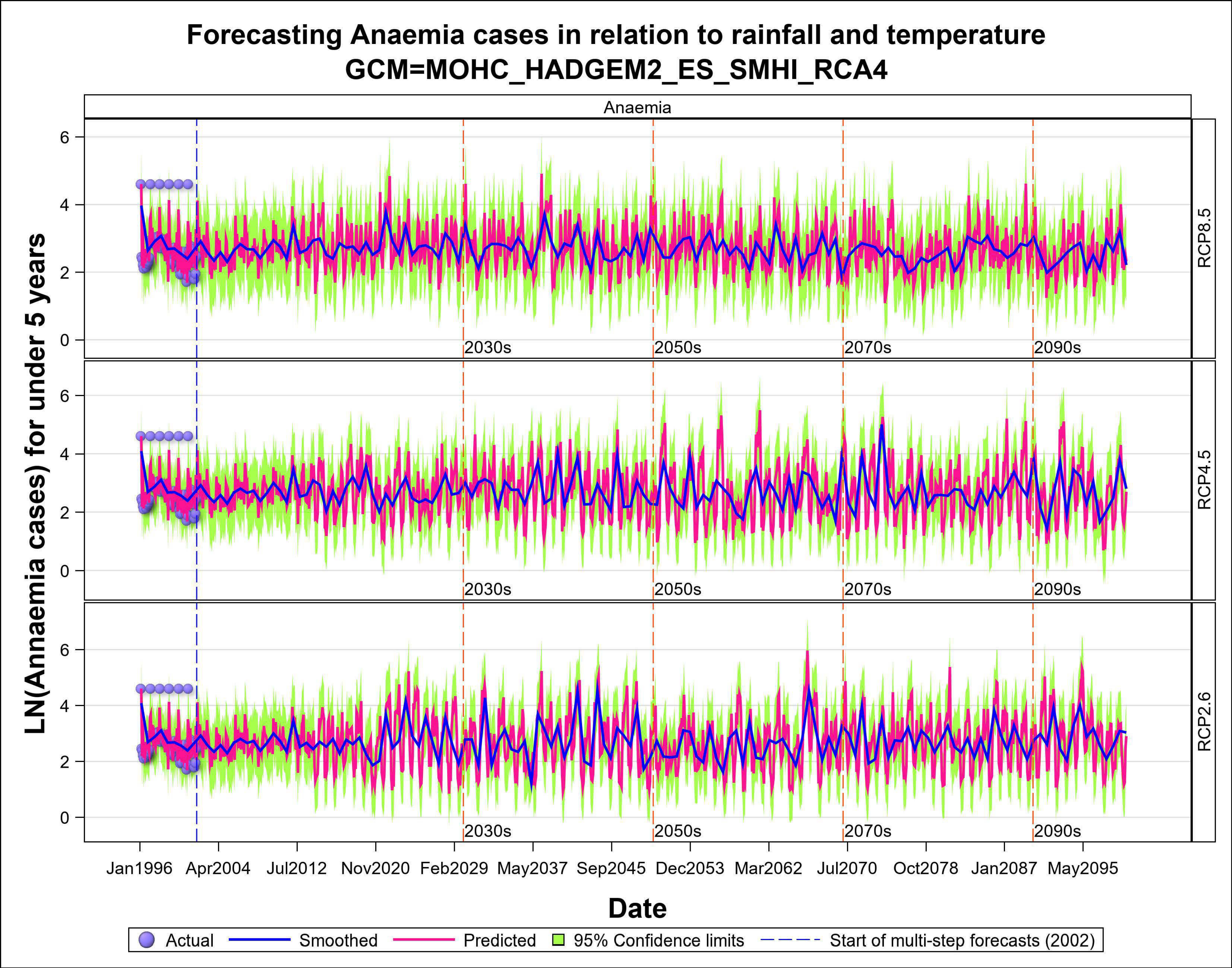

Forecasting Anaemia cases in relation to rainfall and temperature
GCM=MOHC_HADGEM2_ES_SMHI_RCA4
Anaemia
RCP8.5
RCP4.5
RCP2.6
2030s
2050s
2070s
2090s
LN(Annaemia cases) for under 5 years
0
2
4
6
Jan1996
Apr2004
Jul2012
Nov2020
Feb2029
May2037
Sep2045
Dec2053
Mar2062
Jul2070
Oct2078
Jan2087
May2095
Date
Actual
Smoothed
Predicted
95% Confidence limits
Start of multi-step forecasts (2002)

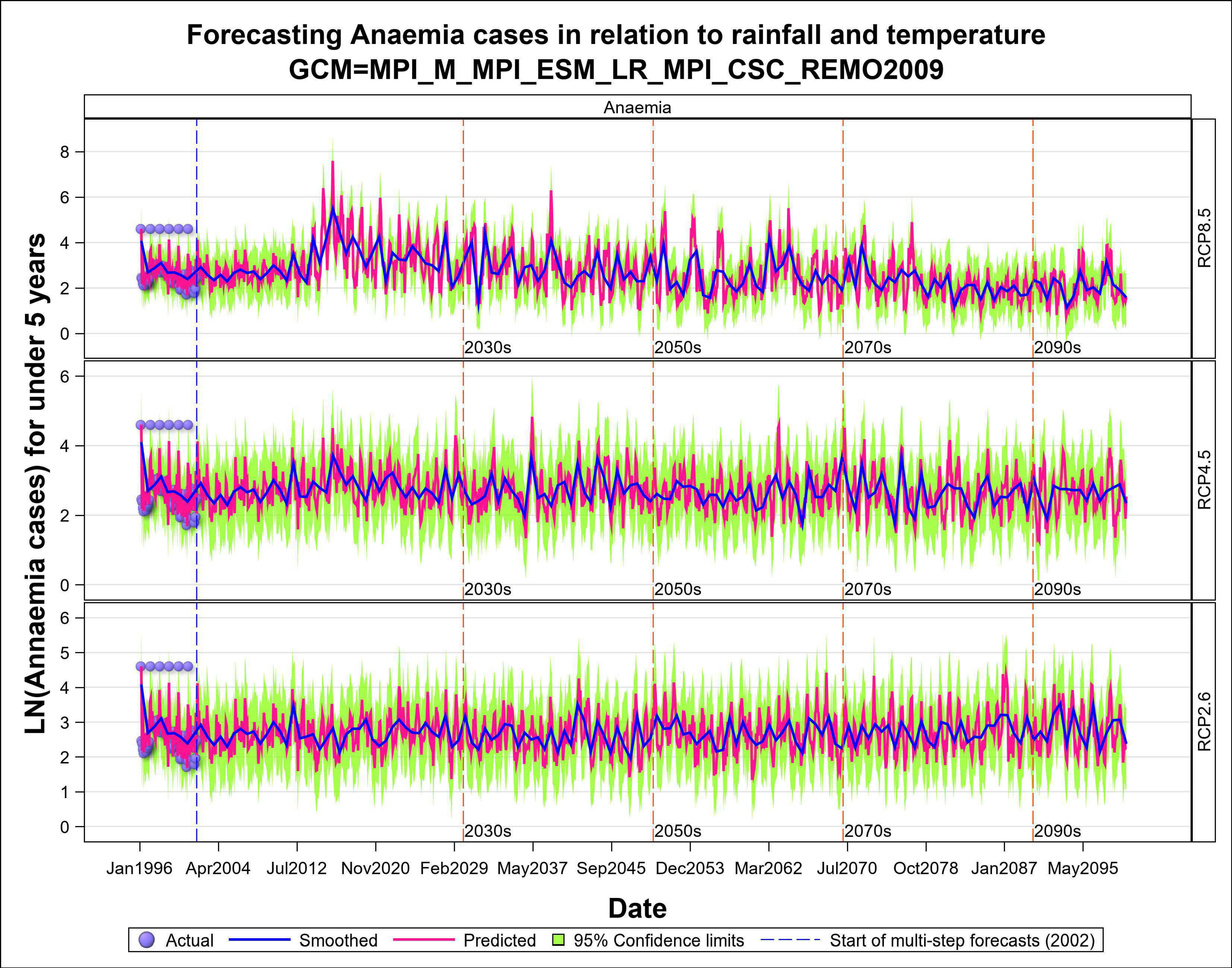

Forecasting Anaemia cases in relation to rainfall and temperature
GCM=MPI_M_MPI_ESM_LR_MPI_CSC_REMO2009
Anaemia
RCP8.5
RCP4.5
RCP2.6
2030s
2050s
2070s
2090s
LN(Annaemia cases) for under 5 years
Jan1996
Apr2004
Jul2012
Nov2020
Feb2029
May2037
Sep2045
Dec2053
Mar2062
Jul2070
Oct2078
Jan2087
May2095
Date
Actual
Smoothed
Predicted
95% Confidence limits
Start of multi-step forecasts (2002)

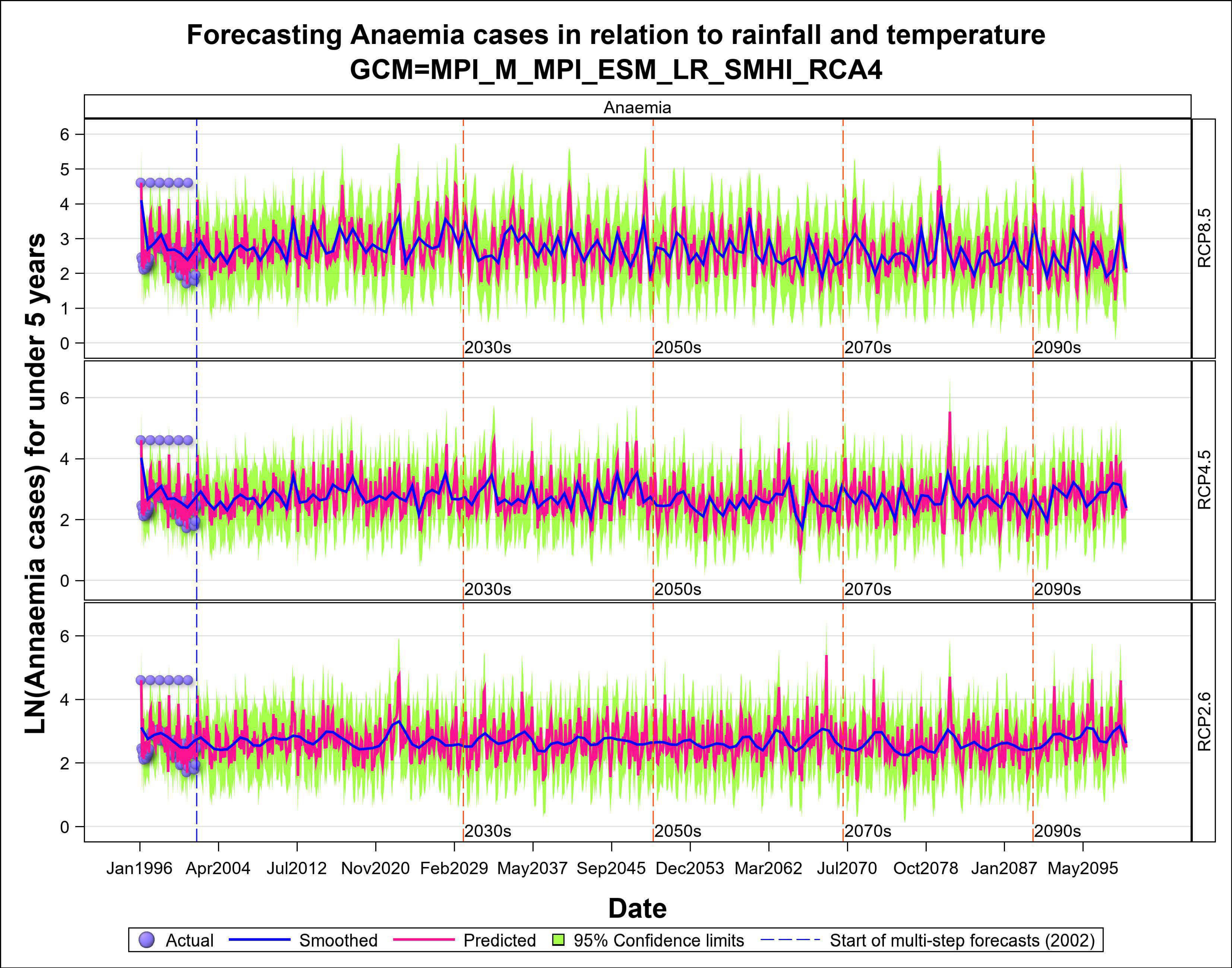

Forecasting Anaemia cases in relation to rainfall and temperature
GCM=MPI_M_MPI_ESM_LR_SMHI_RCA4
Anaemia
RCP8.5
RCP4.5
RCP2.6
2030s
2050s
2070s
2090s
LN(Annaemia cases) for under 5 years
Jan1996
Apr2004
Jul2012
Nov2020
Feb2029
May2037
Sep2045
Dec2053
Mar2062
Jul2070
Oct2078
Jan2087
May2095
Date
Actual
Smoothed
Predicted
95% Confidence limits
Start of multi-step forecasts (2002)

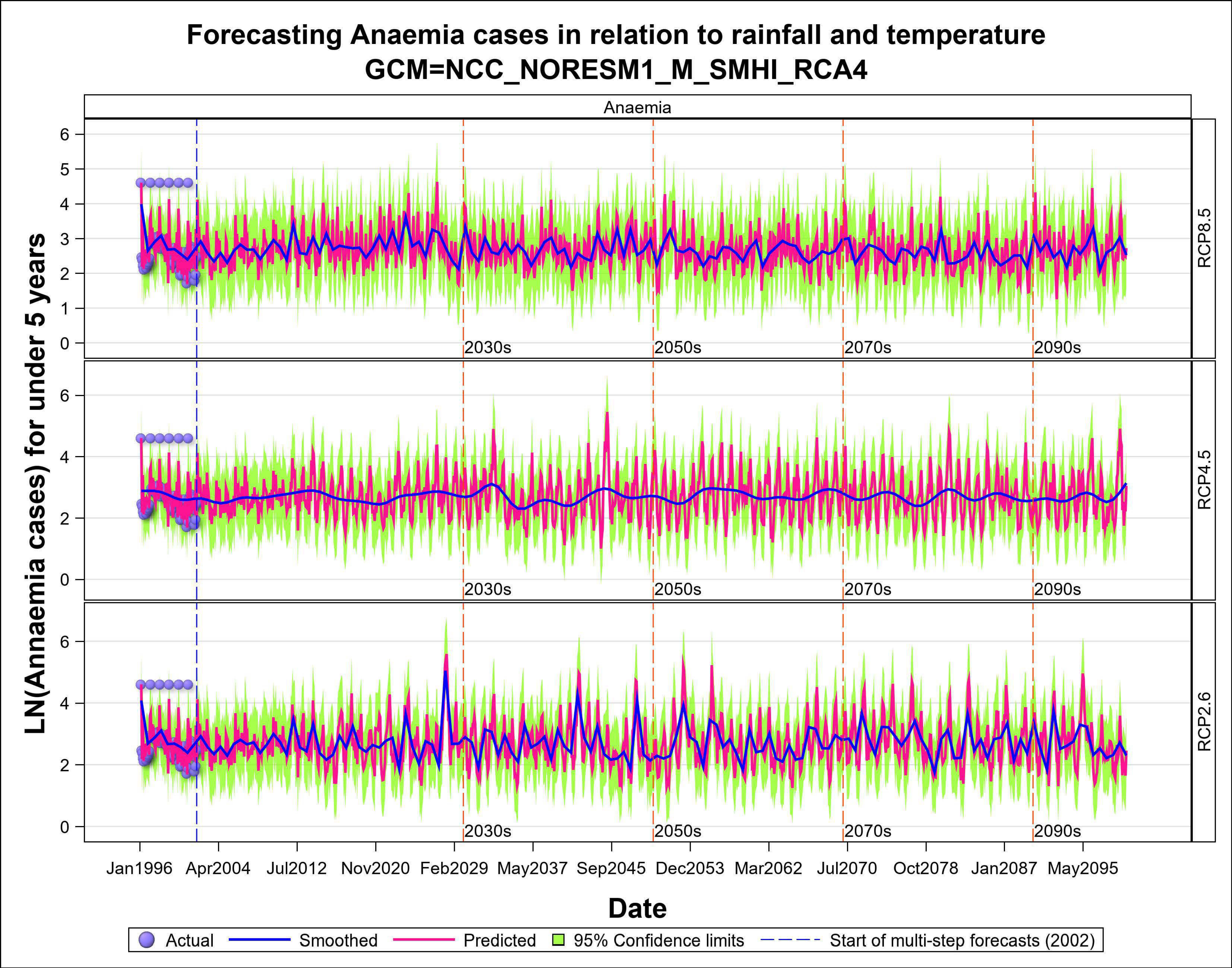

Forecasting Anaemia cases in relation to rainfall and temperature
GCM=NCC_NORESM1_M_SMHI_RCA4
Anaemia
RCP8.5
RCP4.5
RCP2.6
2030s
2050s
2070s
2090s
LN(Annaemia cases) for under 5 years
Jan1996
Apr2004
Jul2012
Nov2020
Feb2029
May2037
Sep2045
Dec2053
Mar2062
Jul2070
Oct2078
Jan2087
May2095
Date
Actual
Smoothed
Predicted
95% Confidence limits
Start of multi-step forecasts (2002)

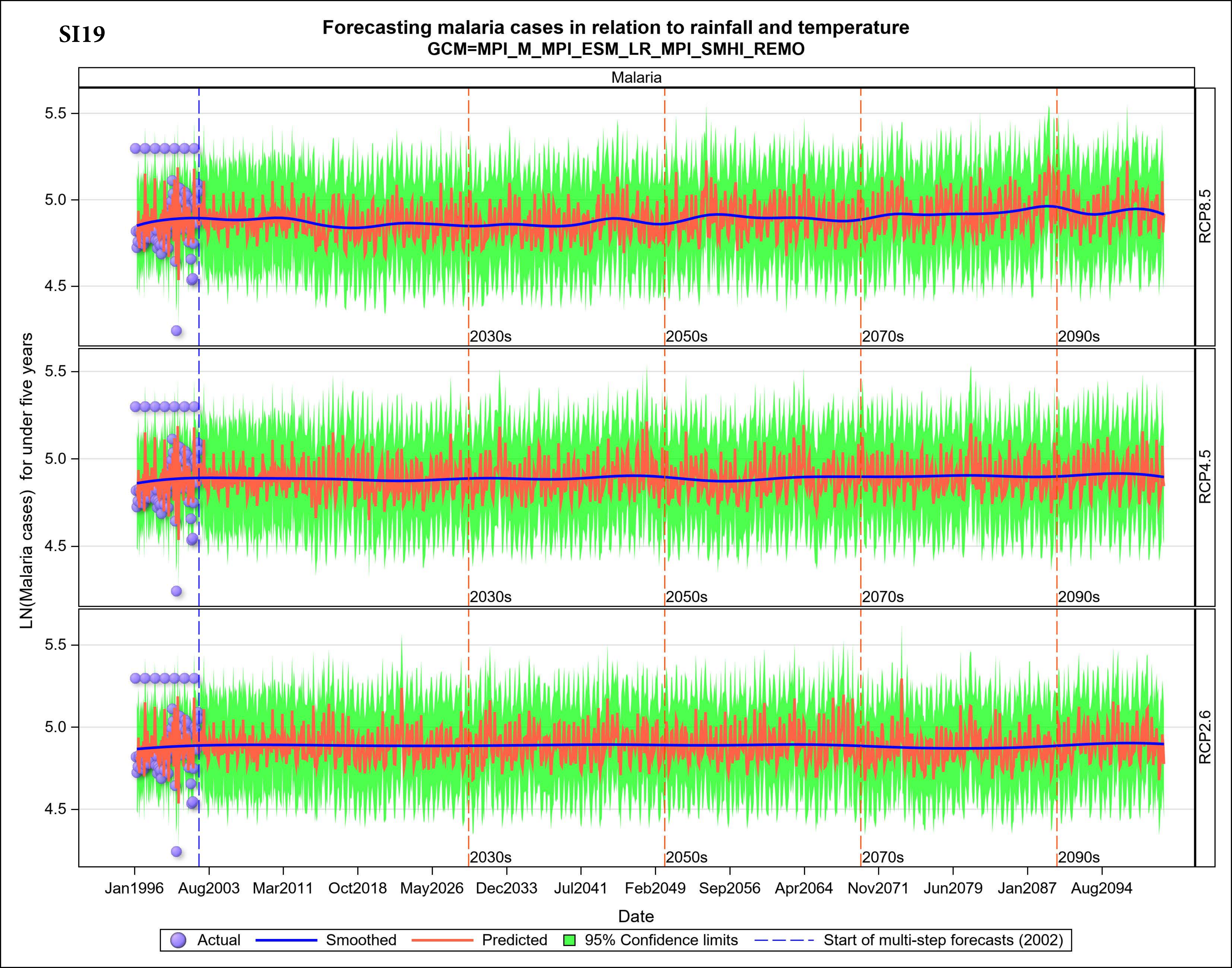
SI19
Forecasting malaria cases in relation to rainfall and temperature
GCM=MPI_M_MPI_ESM_LR_MPI_SMHI_REMO
Malaria
RCP8.5
RCP4.5
RCP2.6
2030s
2050s
2070s
2090s
LN(Malaria cases) for under five years
5.5
5.0
4.5
Jan1996
Aug2003
Mar2011
Oct2018
May2026
Dec2033
Jul2041
Feb2049
Sep2056
Apr2064
Nov2071
Jun2079
Jan2087
Aug2094
Date
Actual
Smoothed
Predicted
95% Confidence limits
Start of multi-step forecasts (2002)

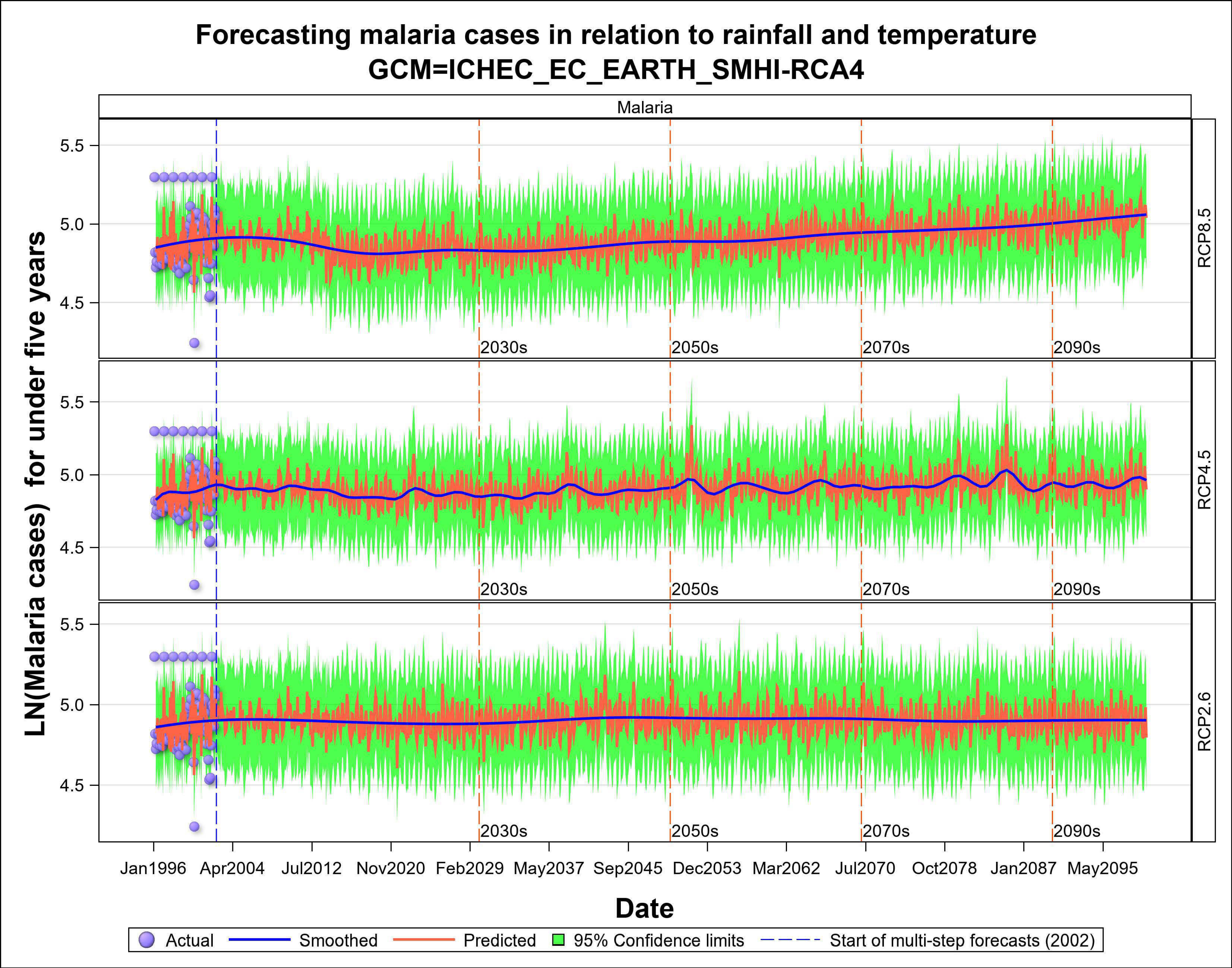

Forecasting malaria cases in relation to rainfall and temperature
GCM=ICHEC_EC_EARTH_SMHI-RCA4
Malaria
RCP8.5
RCP4.5
RCP2.6
2030s
2050s
2070s
2090s
LN(Malaria cases) for under five years
5.5
5.0
4.5
Jan1996
Apr2004
Jul2012
Nov2020
Feb2029
May2037
Sep2045
Dec2053
Mar2062
Jul2070
Oct2078
Jan2087
May2095
Date
Actual
Smoothed
Predicted
95% Confidence limits
Start of multi-step forecasts (2002)

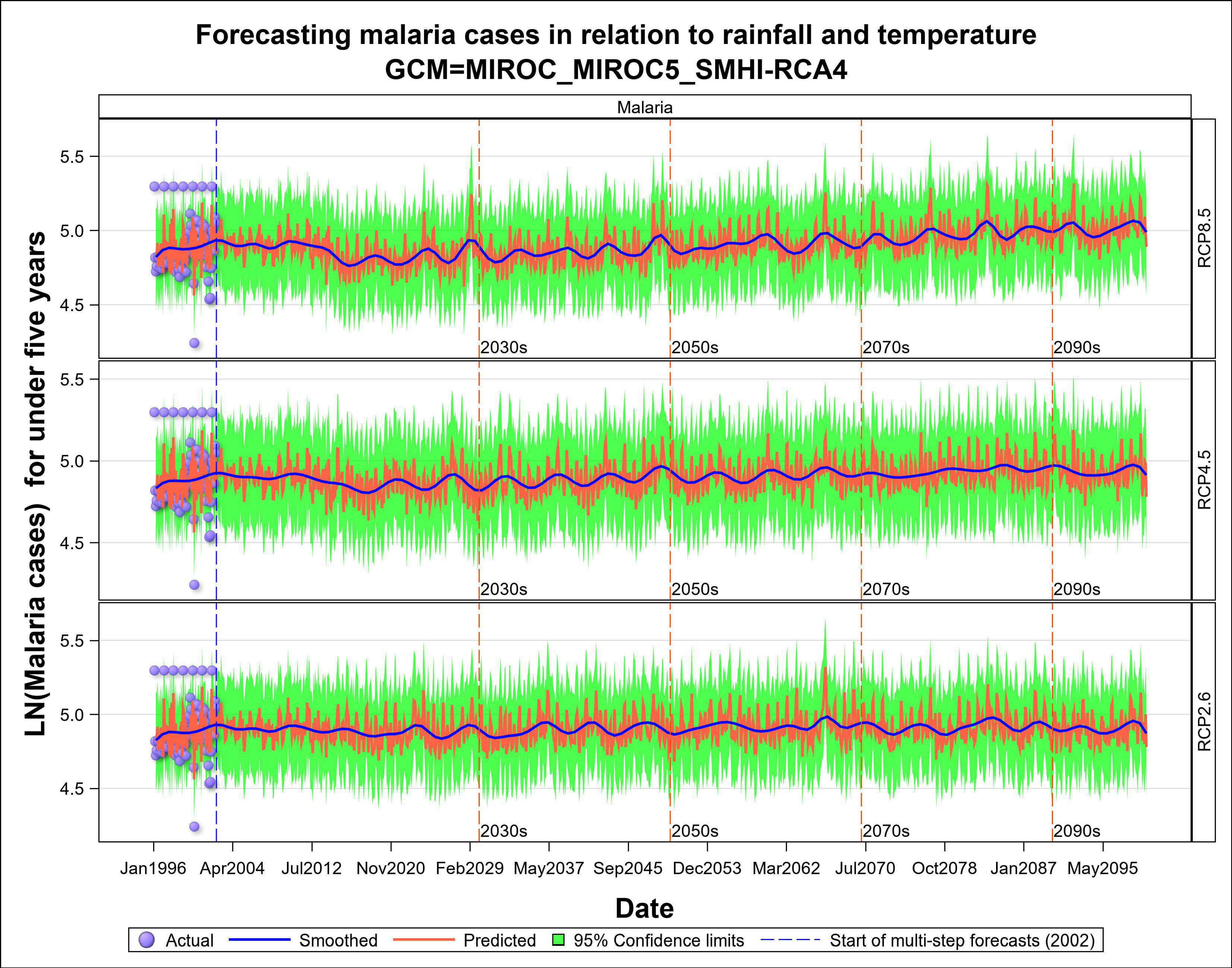

Forecasting malaria cases in relation to rainfall and temperature
GCM=MIROC_MIROC5_SMHI-RCA4
Malaria
RCP8.5
RCP4.5
RCP2.6
2030s
2050s
2070s
2090s
5.5
5.0
4.5
LN(Malaria cases) for under five years
Jan1996
Apr2004
Jul2012
Nov2020
Feb2029
May2037
Sep2045
Dec2053
Mar2062
Jul2070
Oct2078
Jan2087
May2095
Date
Actual
Smoothed
Predicted
95% Confidence limits
Start of multi-step forecasts (2002)

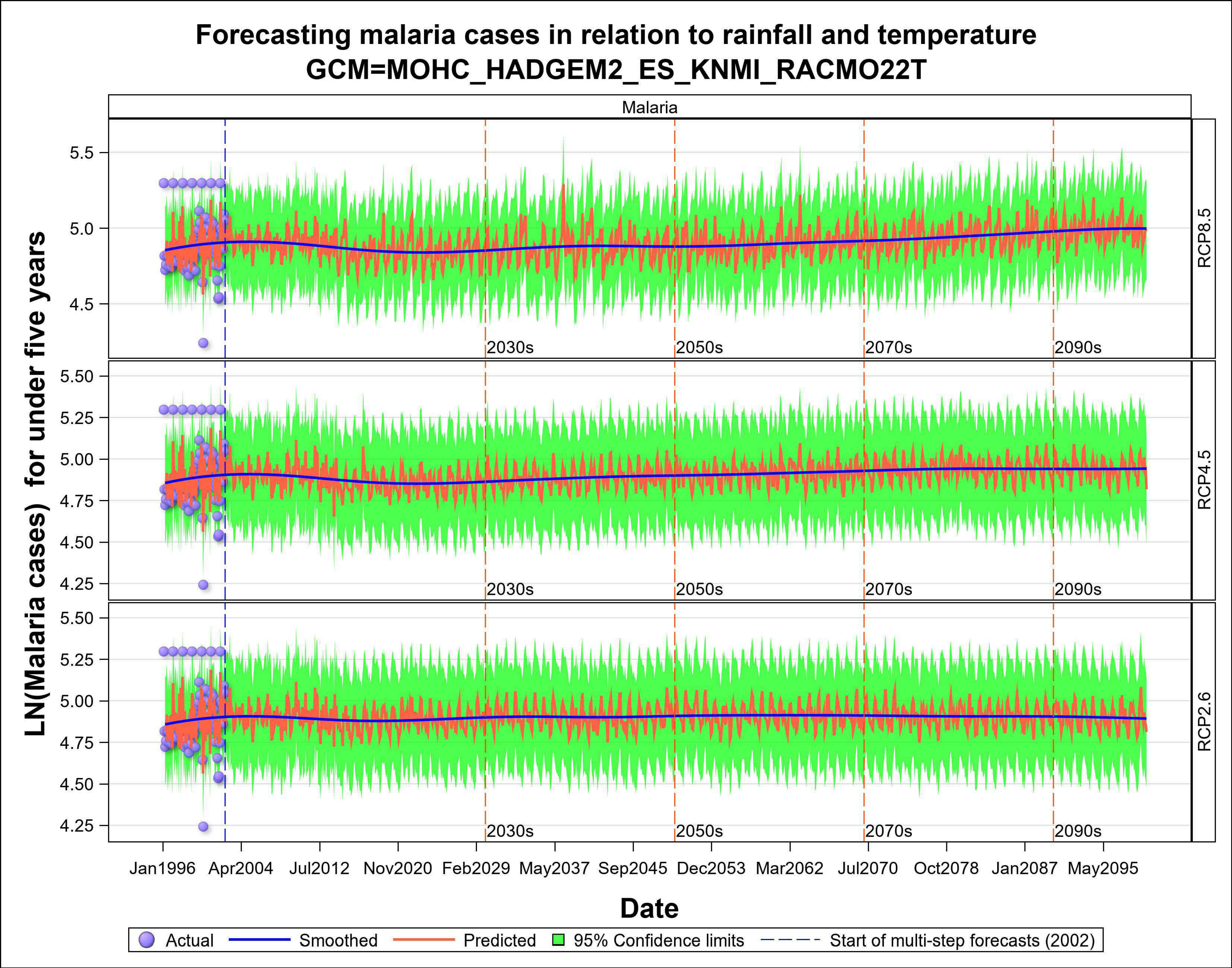

Forecasting malaria cases in relation to rainfall and temperature
GCM=MOHC_HADGEM2_ES_KNMI_RACMO22T
Malaria
RCP8.5
RCP4.5
RCP2.6
LN(Malaria cases) for under five years
5.5
5.0
4.5
5.50
5.25
5.00
4.75
4.50
4.25
2030s
2050s
2070s
2090s
Jan1996
Apr2004
Jul2012
Nov2020
Feb2029
May2037
Sep2045
Dec2053
Mar2062
Jul2070
Oct2078
Jan2087
May2095
Date
Actual
Smoothed
Predicted
95% Confidence limits
Start of multi-step forecasts (2002)

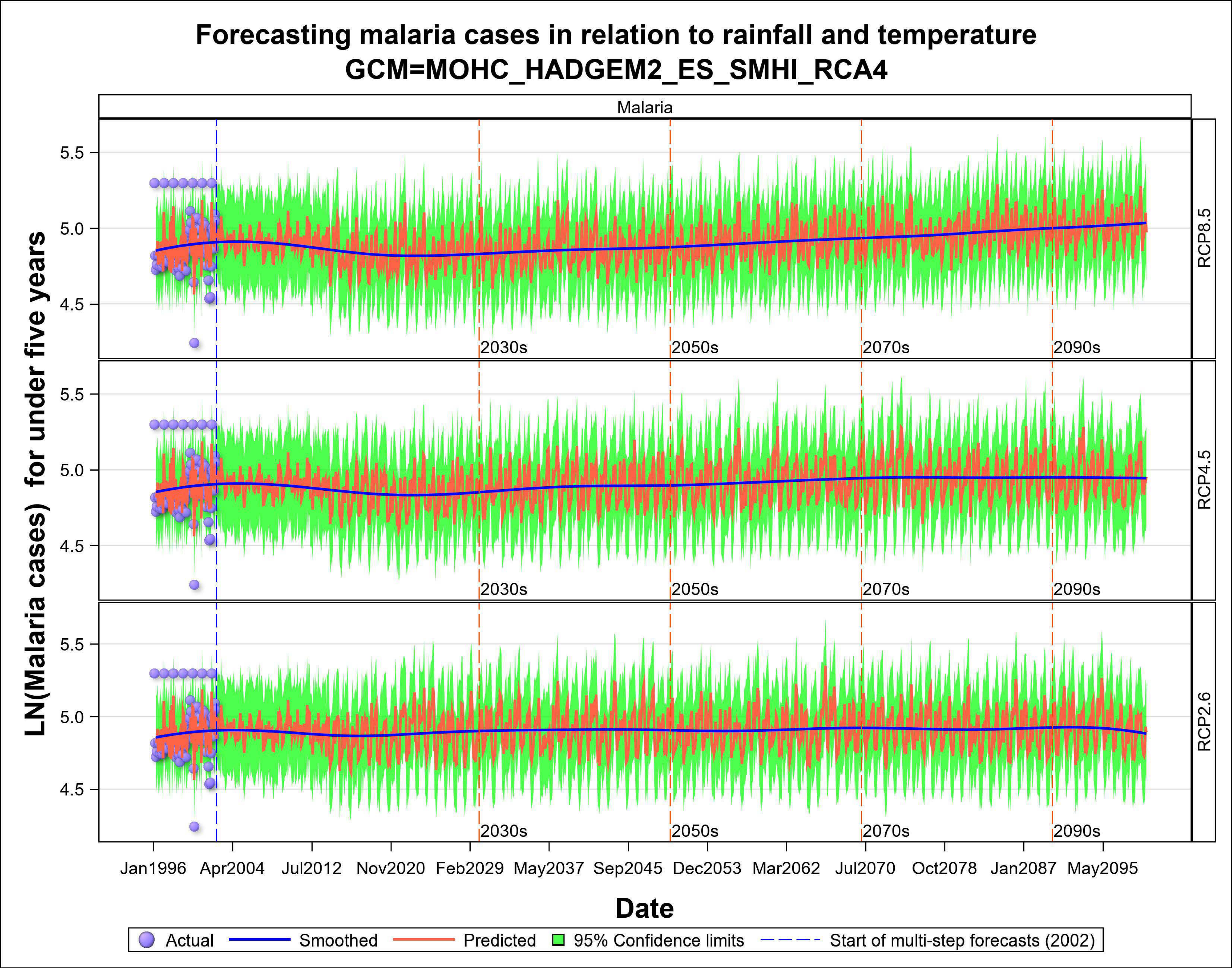

Forecasting malaria cases in relation to rainfall and temperature
GCM=MOHC_HADGEM2_ES_SMHI_RCA4
Malaria
RCP8.5
RCP4.5
RCP2.6
2030s
2050s
2070s
2090s
5.5
5.0
4.5
LN(Malaria cases) for under five years
Jan1996
Apr2004
Jul2012
Nov2020
Feb2029
May2037
Sep2045
Dec2053
Mar2062
Jul2070
Oct2078
Jan2087
May2095
Date
Actual
Smoothed
Predicted
95% Confidence limits
Start of multi-step forecasts (2002)

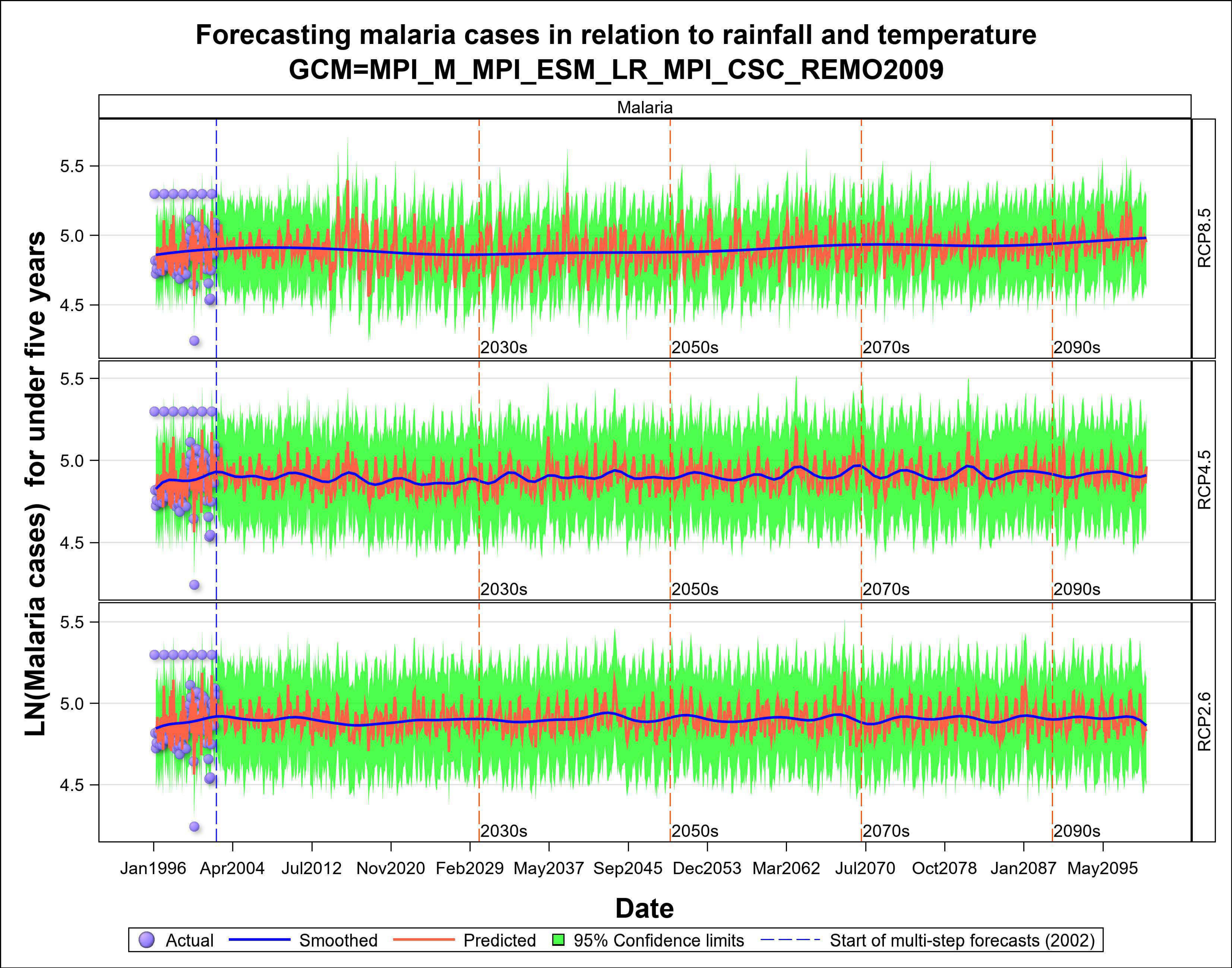
Forecasting malaria cases in relation to rainfall and temperature
GCM=MPI_M_MPI_ESM_LR_MPI_CSC_REMO2009
Malaria
RCP8.5
RCP4.5
RCP2.6
2030s
2050s
2070s
2090s
5.5
5.0
4.5
LN(Malaria cases) for under five years
Jan1996
Apr2004
Jul2012
Nov2020
Feb2029
May2037
Sep2045
Dec2053
Mar2062
Jul2070
Oct2078
Jan2087
May2095
Date
Actual
Smoothed
Predicted
95% Confidence limits
Start of multi-step forecasts (2002)

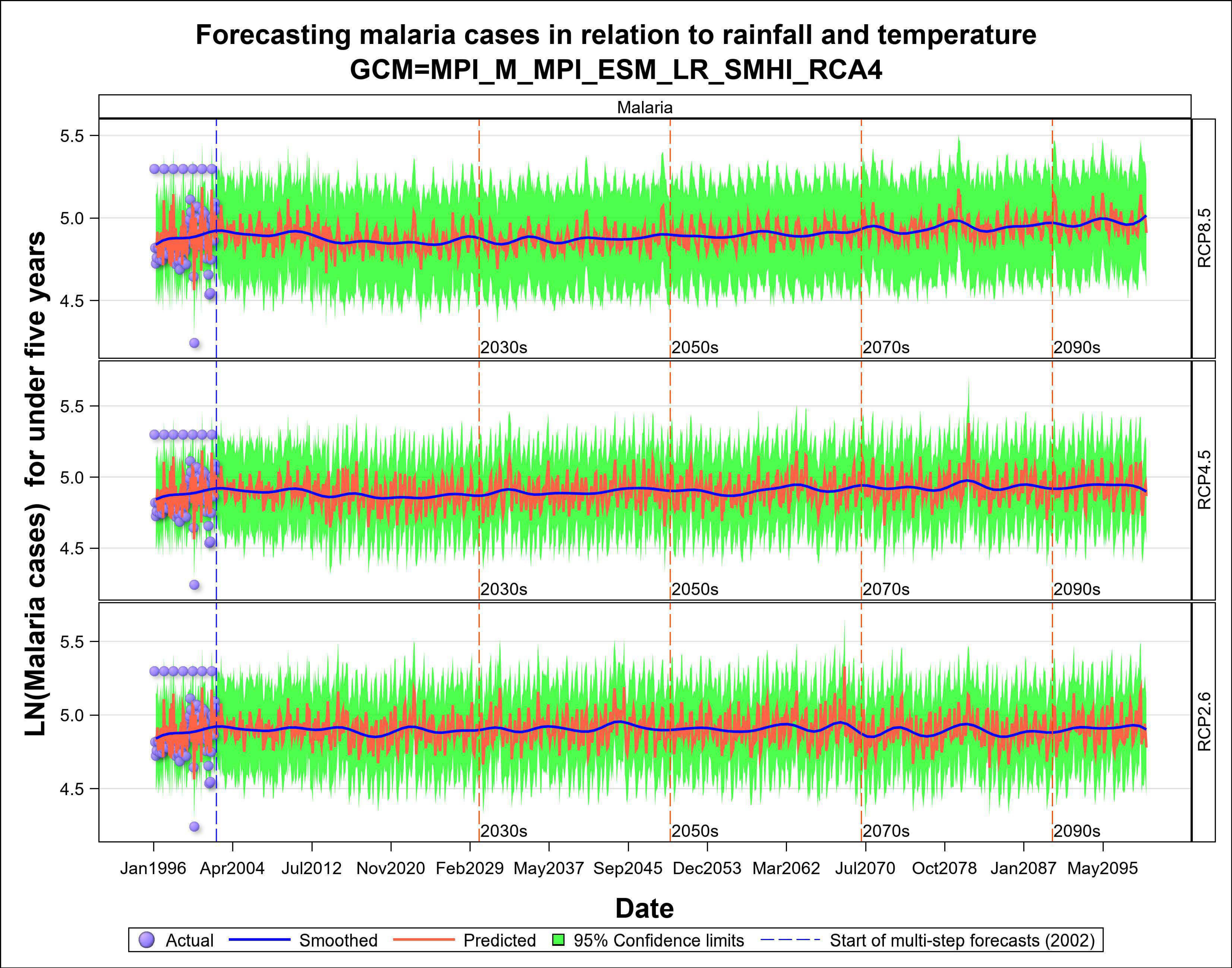

Forecasting malaria cases in relation to rainfall and temperature
GCM=MPI_M_MPI_ESM_LR_SMHI_RCA4
Malaria
RCP8.5
RCP4.5
RCP2.6
2030s
2050s
2070s
2090s
5.5
5.0
4.5
LN(Malaria cases) for under five years
Jan1996
Apr2004
Jul2012
Nov2020
Feb2029
May2037
Sep2045
Dec2053
Mar2062
Jul2070
Oct2078
Jan2087
May2095
Date
Actual
Smoothed
Predicted
95% Confidence limits
Start of multi-step forecasts (2002)

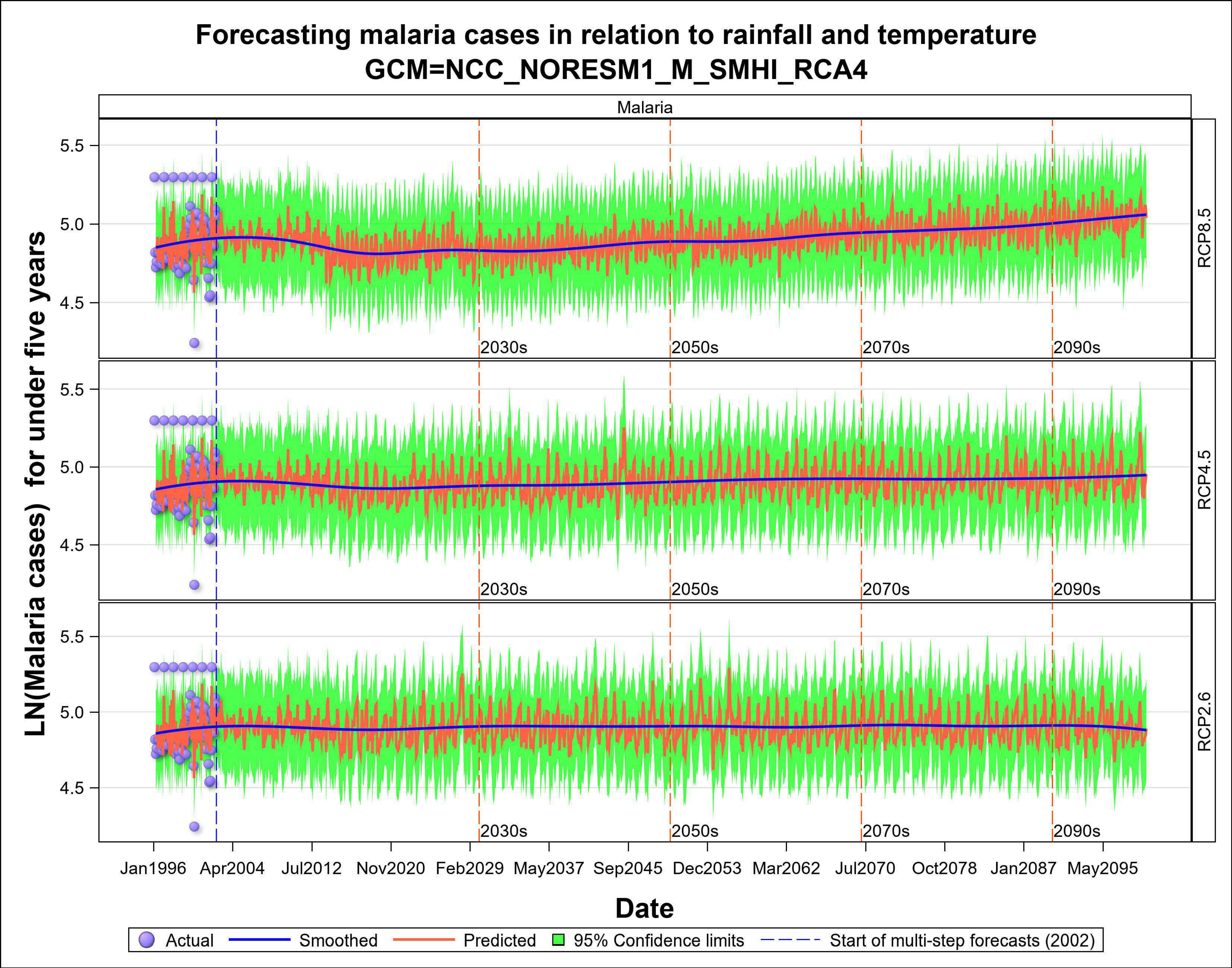

Forecasting malaria cases in relation to rainfall and temperature
GCM=NCC_NORESM1_M_SMHI_RCA4
Malaria
RCP8.5
RCP4.5
RCP2.6
2030s
2050s
2070s
2090s
5.5
5.0
4.5
LN(Malaria cases) for under five years
Jan1996
Apr2004
Jul2012
Nov2020
Feb2029
May2037
Sep2045
Dec2053
Mar2062
Jul2070
Oct2078
Jan2087
May2095
Date
Actual
Smoothed
Predicted
95% Confidence limits
Start of multi-step forecasts (2002)

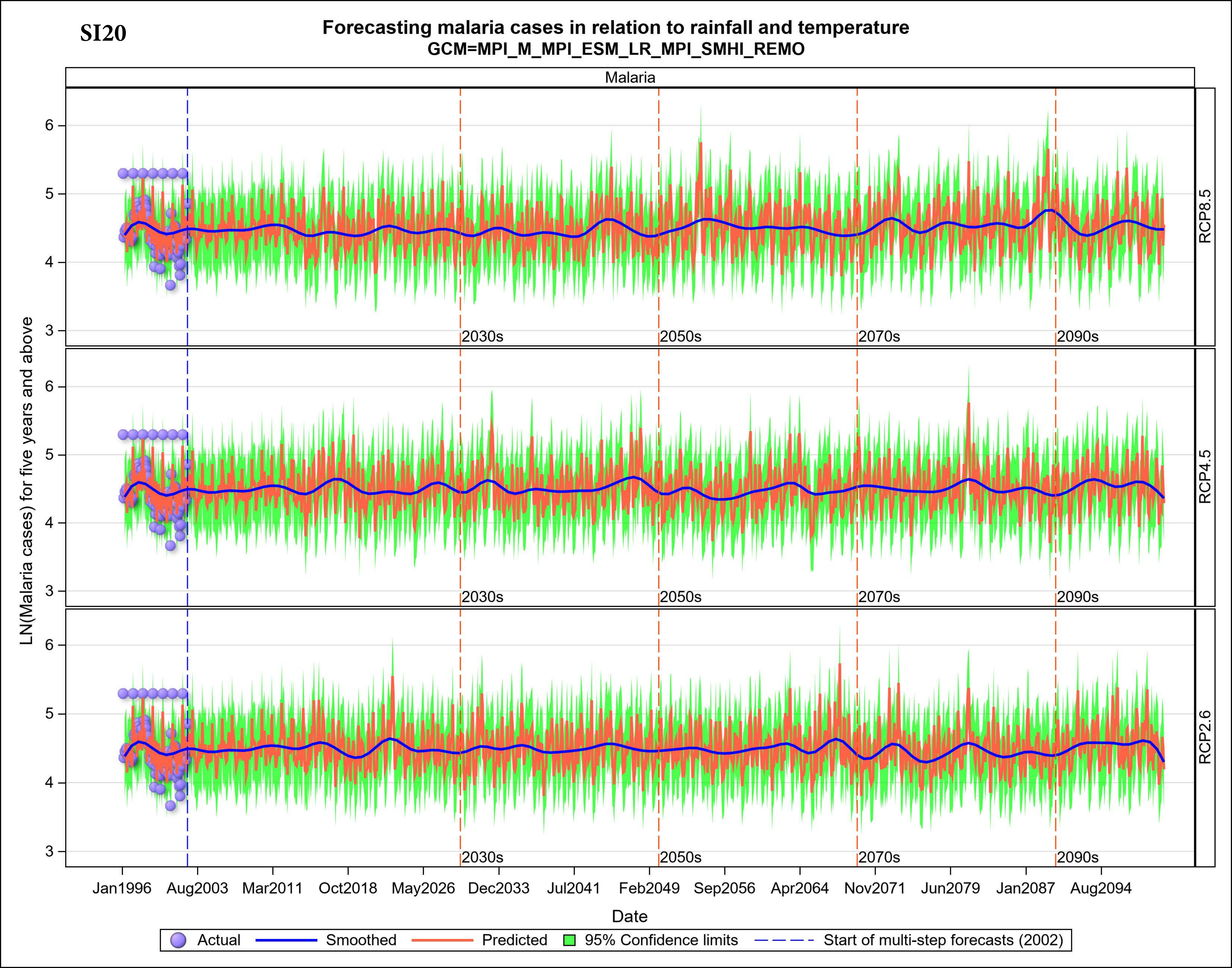

SI20
Forecasting malaria cases in relation to rainfall and temperature
GCM=MPI_M_MPI_ESM_LR_MPI_SMHI_REMO
Malaria
RCP8.5
RCP4.5
RCP2.6
2030s
2050s
2070s
2090s
LN(Malaria cases) for five years and above
Date
Jan1996
Aug2003
Mar2011
Oct2018
May2026
Dec2033
Jul2041
Feb2049
Sep2056
Apr2064
Nov2071
Jun2079
Jan2087
Aug2094
Actual
Smoothed
Predicted
95% Confidence limits
Start of multi-step forecasts (2002)

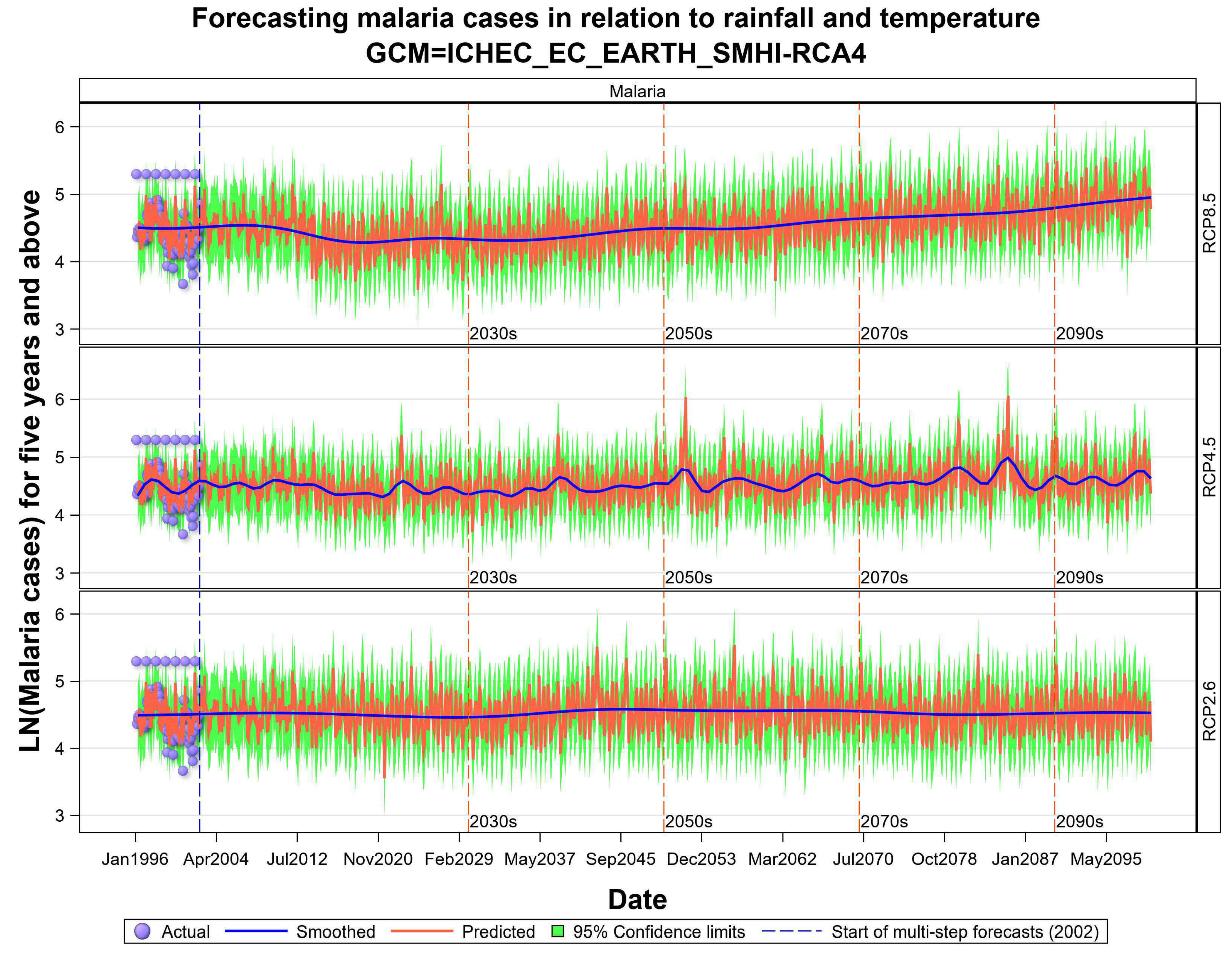

Forecasting malaria cases in relation to rainfall and temperature
GCM=ICHEC_EC_EARTH_SMHI-RCA4
Malaria
RCP8.5
RCP4.5
RCP2.6
2030s
2050s
2070s
2090s
LN(Malaria cases) for five years and above
Jan1996
Apr2004
Jul2012
Nov2020
Feb2029
May2037
Sep2045
Dec2053
Mar2062
Jul2070
Oct2078
Jan2087
May2095
Date
Actual
Smoothed
Predicted
95% Confidence limits
Start of multi-step forecasts (2002)

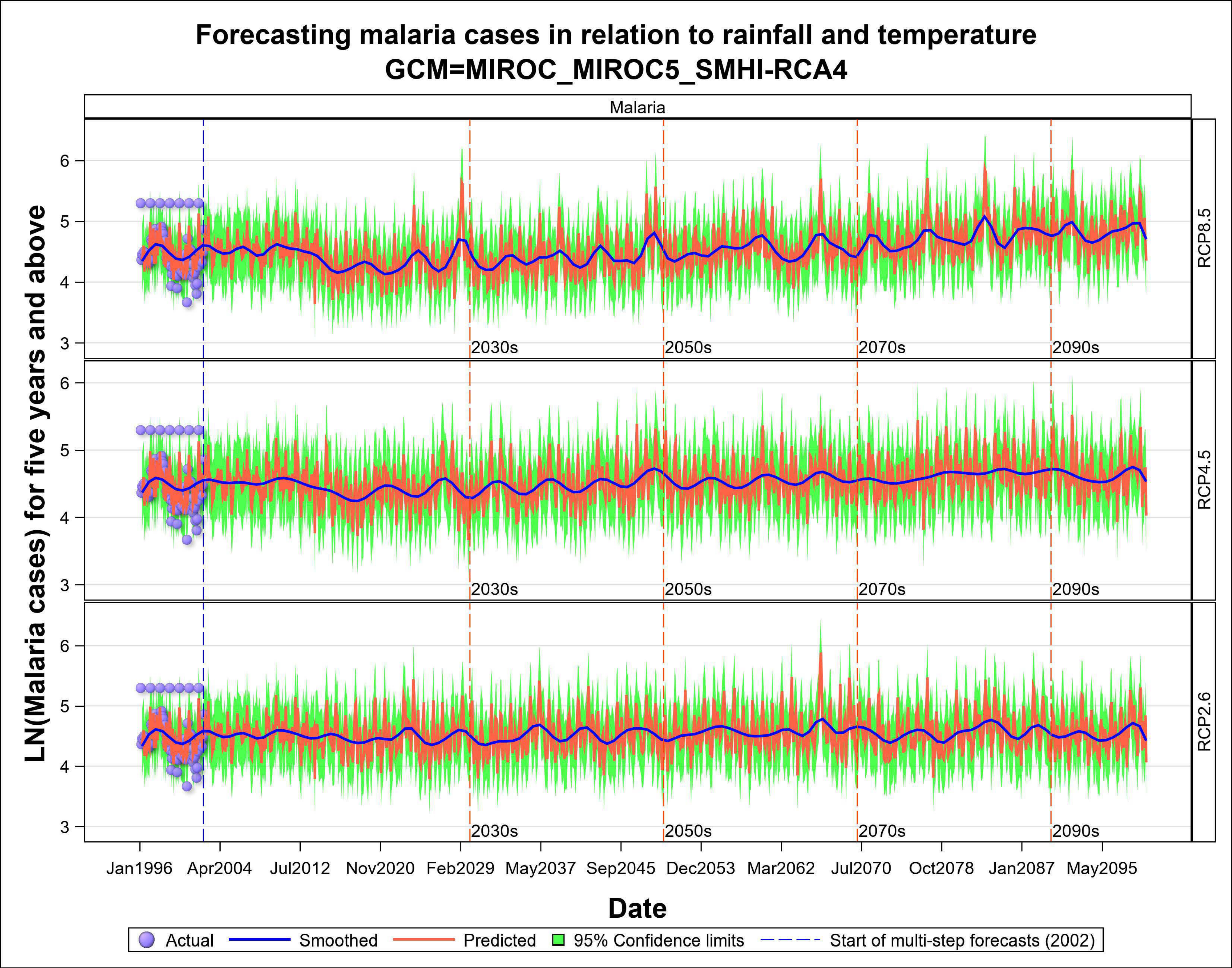

Forecasting malaria cases in relation to rainfall and temperature
GCM=MIROC_MIROC5_SMHI-RCA4
Malaria
RCP8.5
RCP4.5
RCP2.6
2030s
2050s
2070s
2090s
LN(Malaria cases) for five years and above
Jan1996
Apr2004
Jul2012
Nov2020
Feb2029
May2037
Sep2045
Dec2053
Mar2062
Jul2070
Oct2078
Jan2087
May2095
Date
Actual
Smoothed
Predicted
95% Confidence limits
Start of multi-step forecasts (2002)

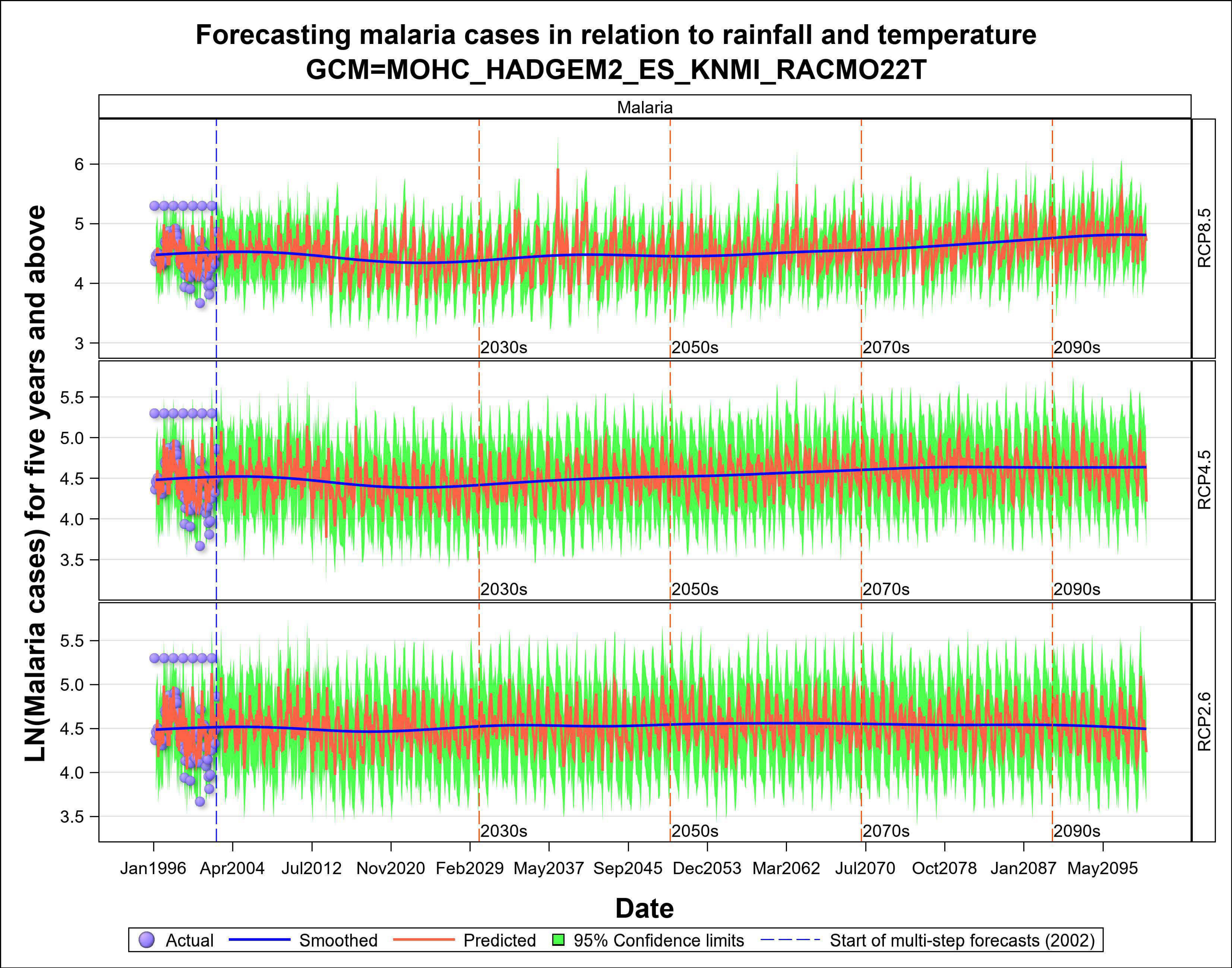

Forecasting malaria cases in relation to rainfall and temperature
GCM=MOHC_HADGEM2_ES_KNMI_RACMO22T
Malaria
RCP8.5
RCP4.5
RCP2.6
2030s
2050s
2070s
2090s
LN(Malaria cases) for five years and above
Jan1996
Apr2004
Jul2012
Nov2020
Feb2029
May2037
Sep2045
Dec2053
Mar2062
Jul2070
Oct2078
Jan2087
May2095
Date
Actual
Smoothed
Predicted
95% Confidence limits
Start of multi-step forecasts (2002)

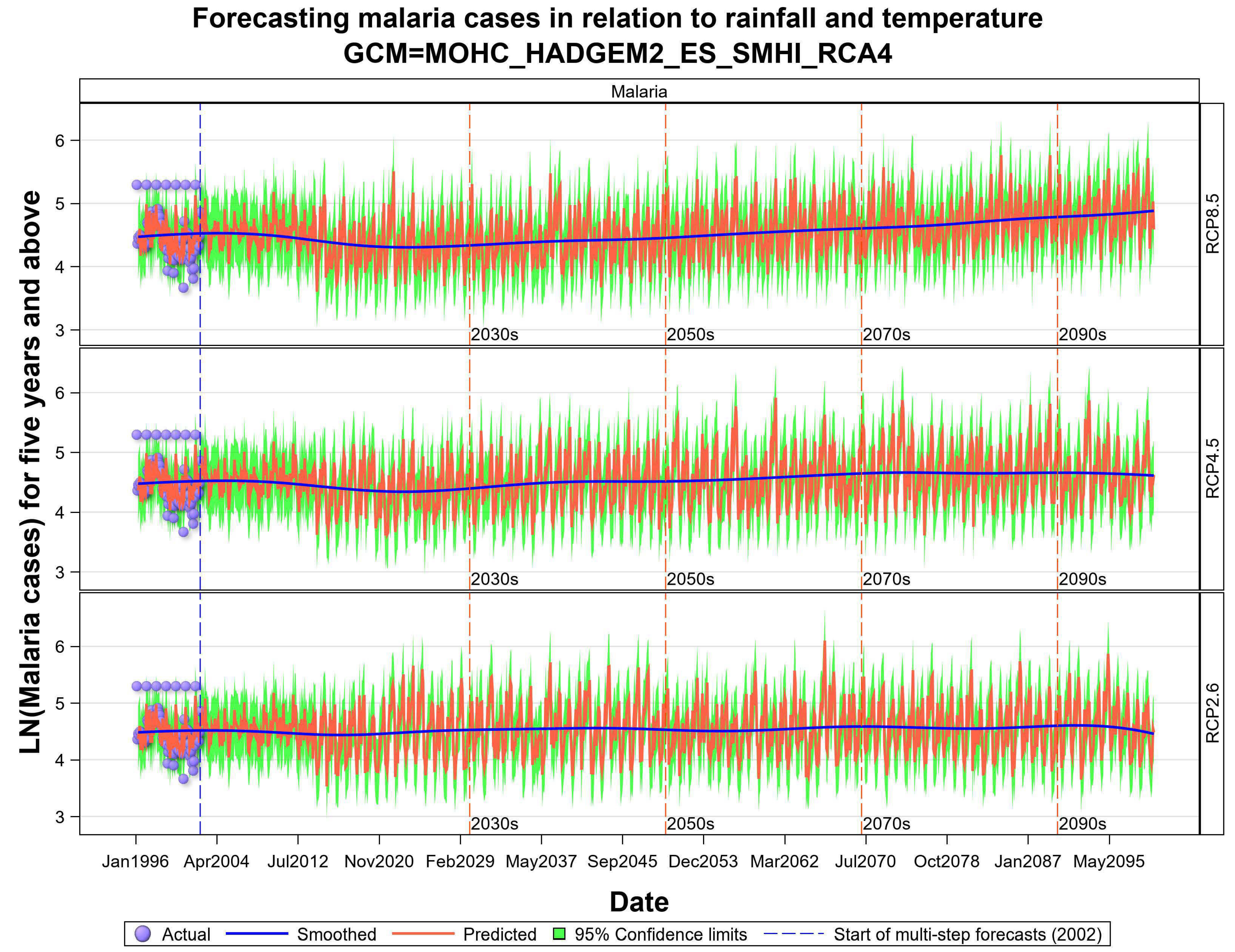

Forecasting malaria cases in relation to rainfall and temperature
GCM=MOHC_HADGEM2_ES_SMHI_RCA4
Malaria
RCP8.5
RCP4.5
RCP2.6
2030s
2050s
2070s
2090s
LN(Malaria cases) for five years and above
Jan1996
Apr2004
Jul2012
Nov2020
Feb2029
May2037
Sep2045
Dec2053
Mar2062
Jul2070
Oct2078
Jan2087
May2095
Date
Actual
Smoothed
Predicted
95% Confidence limits
Start of multi-step forecasts (2002)

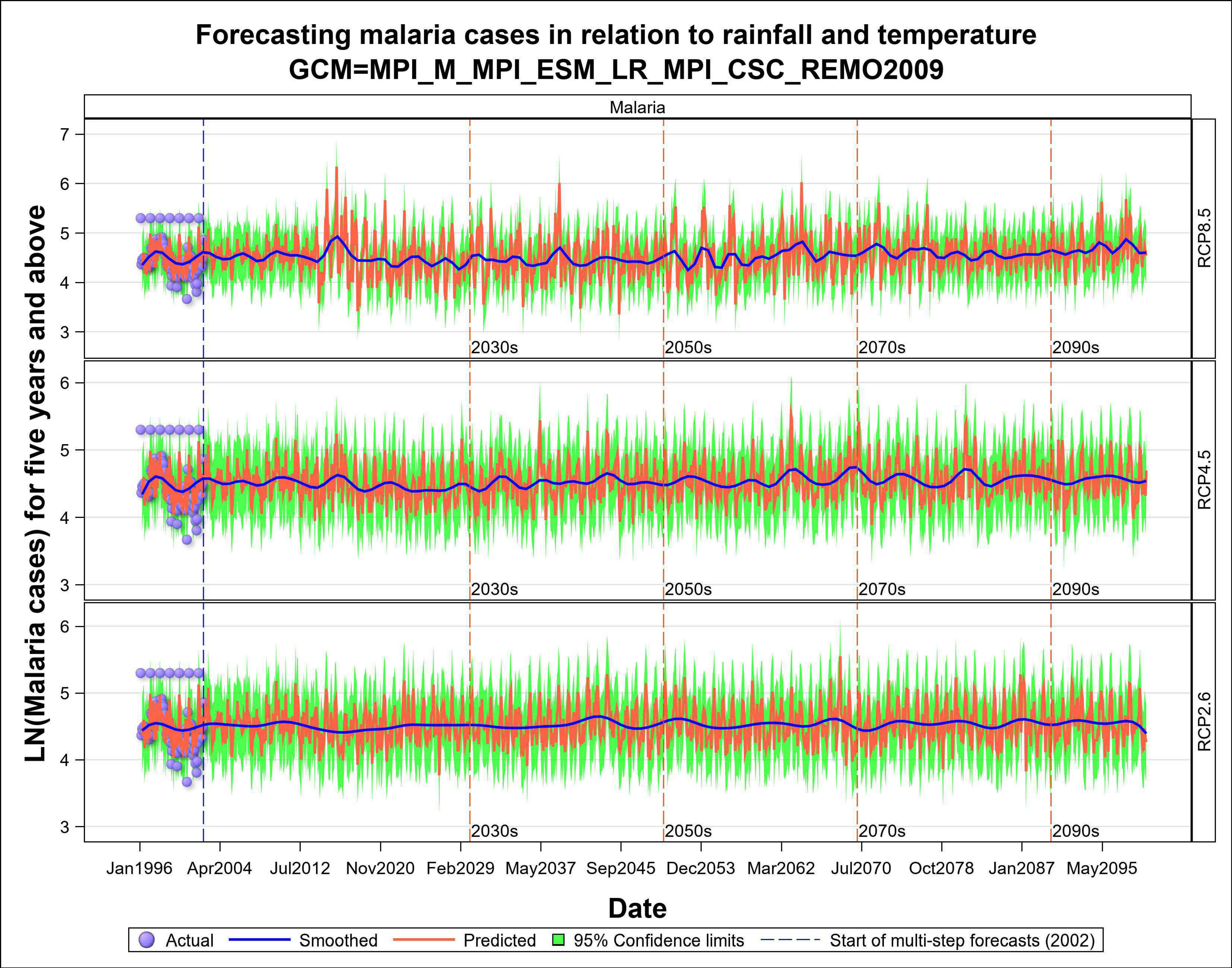
Forecasting malaria cases in relation to rainfall and temperature
GCM=MPI_M_MPI_ESM_LR_MPI_CSC_REMO2009
Malaria
RCP8.5
RCP4.5
RCP2.6
2030s
2050s
2070s
2090s
LN(Malaria cases) for five years and above
Jan1996
Apr2004
Jul2012
Nov2020
Feb2029
May2037
Sep2045
Dec2053
Mar2062
Jul2070
Oct2078
Jan2087
May2095
Date
Actual
Smoothed
Predicted
95% Confidence limits
Start of multi-step forecasts (2002)

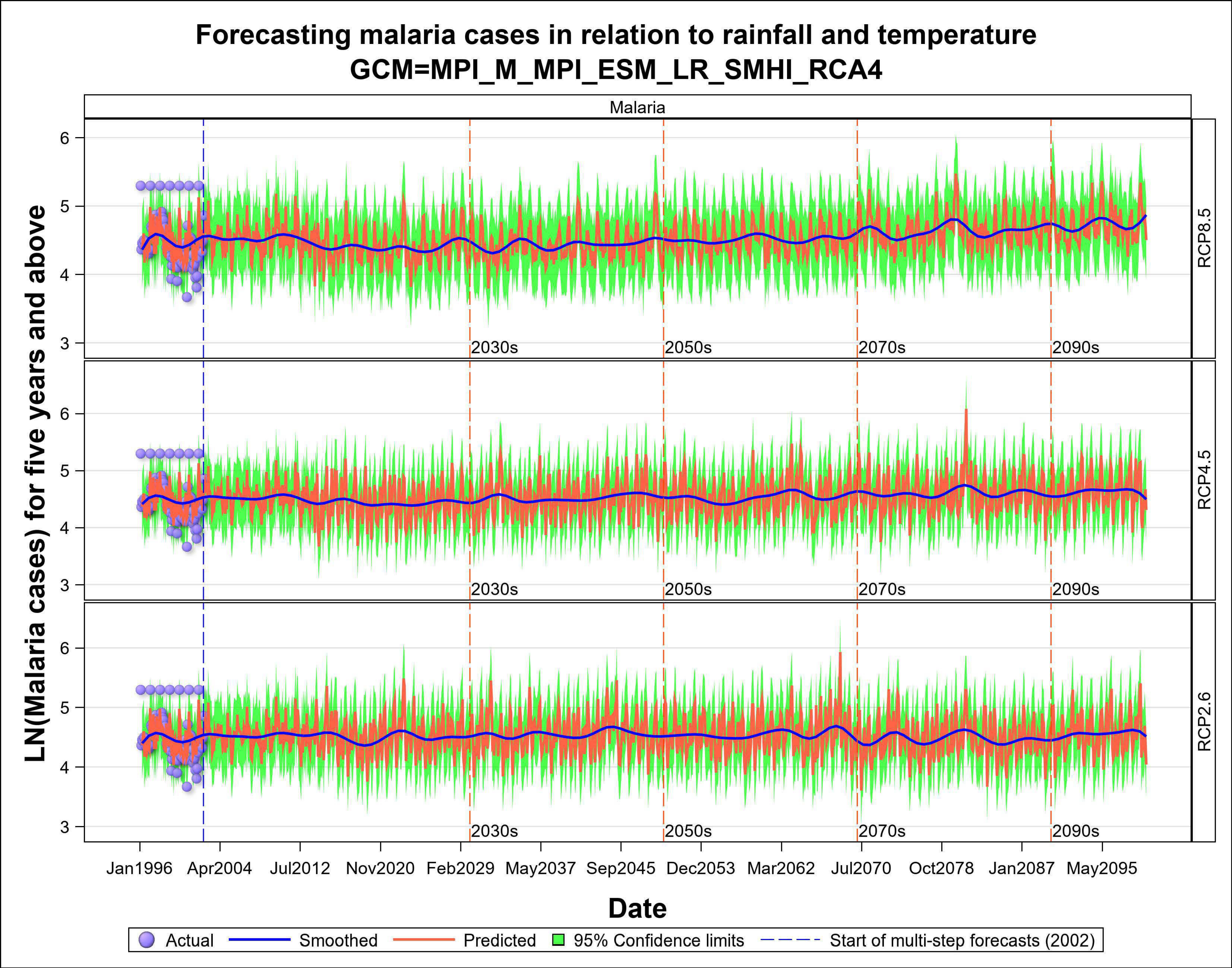

Forecasting malaria cases in relation to rainfall and temperature
GCM=MPI_M_MPI_ESM_LR_SMHI_RCA4
Malaria
RCP8.5
RCP4.5
RCP2.6
2030s
2050s
2070s
2090s
3
4
5
6
LN(Malaria cases) for five years and above
Jan1996
Apr2004
Jul2012
Nov2020
Feb2029
May2037
Sep2045
Dec2053
Mar2062
Jul2070
Oct2078
Jan2087
May2095
Date
Actual
Smoothed
Predicted
95% Confidence limits
Start of multi-step forecasts (2002)

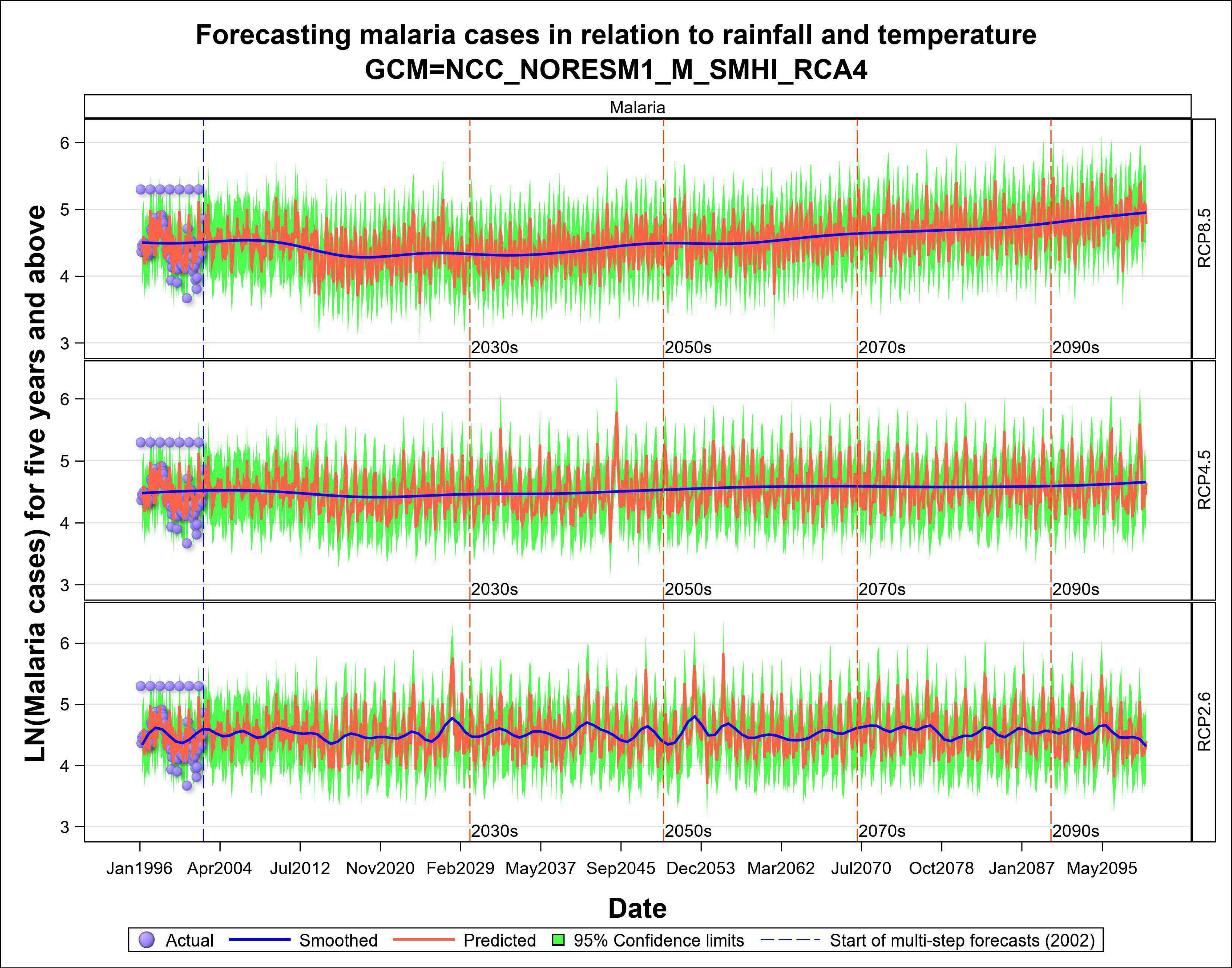

Forecasting malaria cases in relation to rainfall and temperature
GCM=NCC_NORESM1_M_SMHI_RCA4
Malaria
RCP8.5
RCP4.5
RCP2.6
2030s
2050s
2070s
2090s
LN(Malaria cases) for five years and above
6
5
4
3
Jan1996
Apr2004
Jul2012
Nov2020
Feb2029
May2037
Sep2045
Dec2053
Mar2062
Jul2070
Oct2078
Jan2087
May2095
Date
Actual
Smoothed
Predicted
95% Confidence limits
Start of multi-step forecasts (2002)

SI21

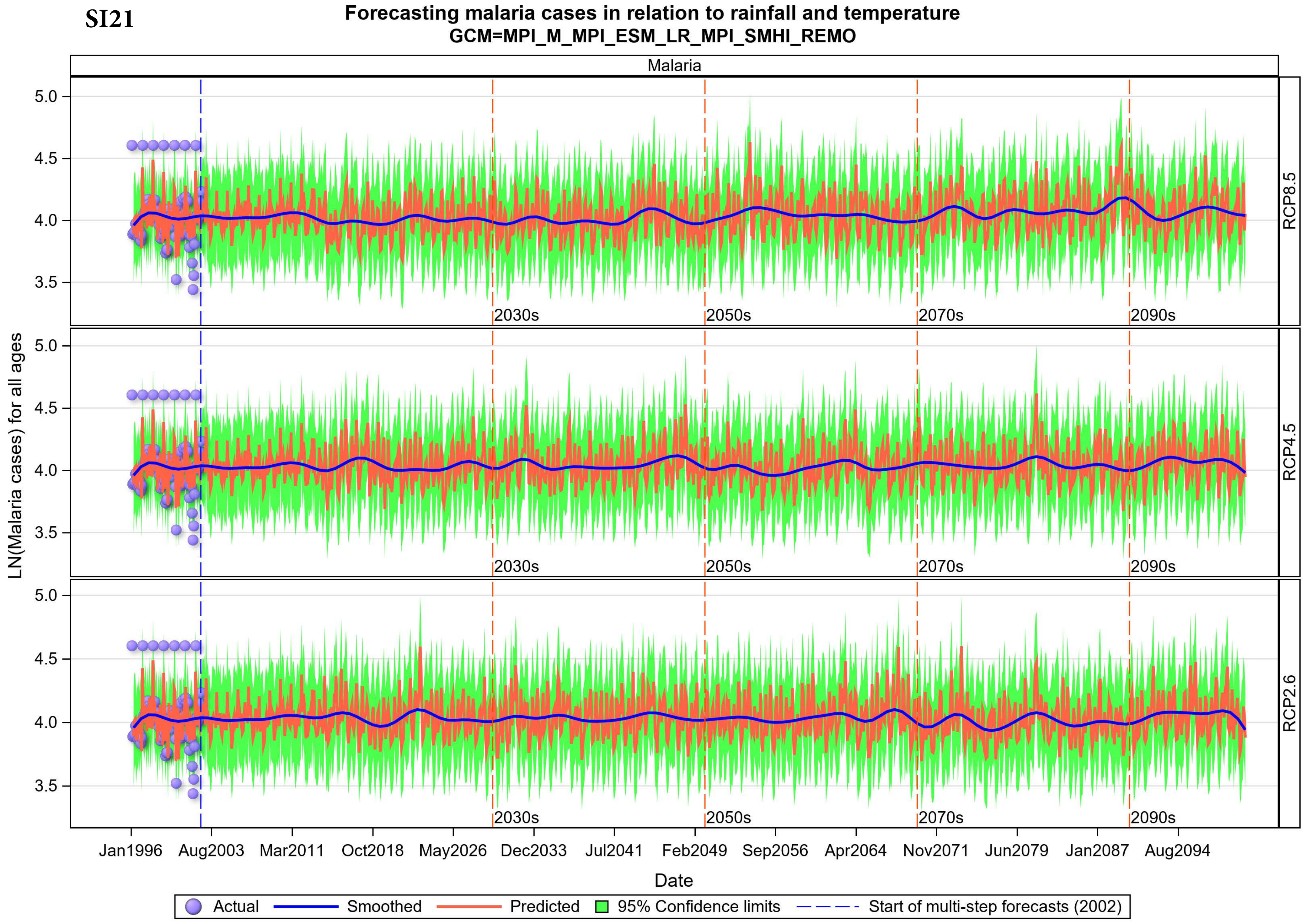
Forecasting malaria cases in relation to rainfall and temperature
GCM=MPI_M_MPI_ESM_LR_MPI_SMHI_REMO
Malaria
LN(Malaria cases) for all ages
RCP8.5
RCP4.5
RCP2.6
2030s
2050s
2070s
2090s
5.0
4.5
4.0
3.5
Jan1996
Aug2003
Mar2011
Oct2018
May2026
Dec2033
Jul2041
Feb2049
Sep2056
Apr2064
Nov2071
Jun2079
Jan2087
Aug2094
Date
Actual
Smoothed
Predicted
95% Confidence limits
Start of multi-step forecasts (2002)

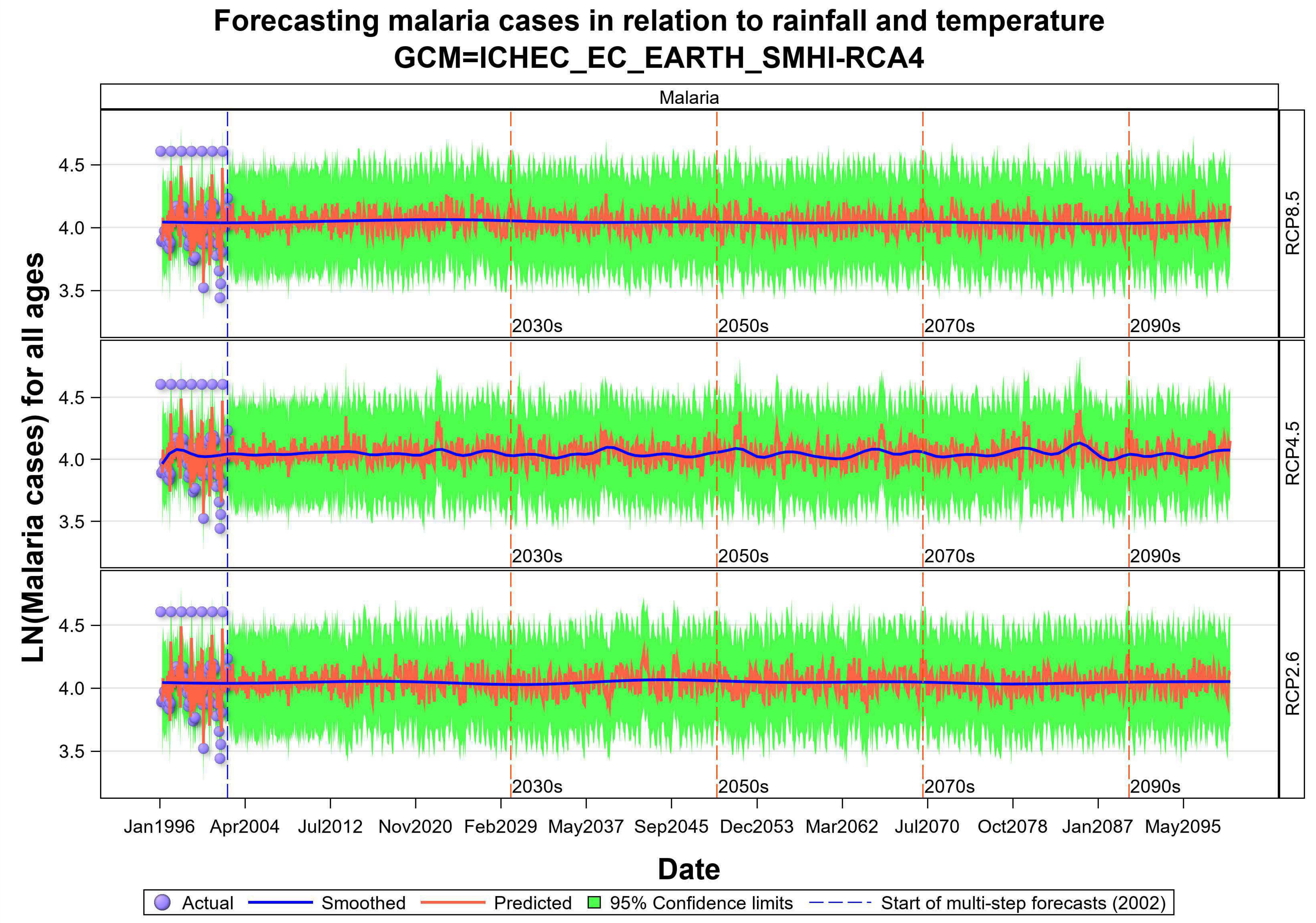

Forecasting malaria cases in relation to rainfall and temperature
GCM=ICHEC_EC_EARTH_SMHI-RCA4
Malaria
RCP8.5
RCP4.5
RCP2.6
2030s
2050s
2070s
2090s
4.5
4.0
3.5
LN(Malaria cases) for all ages
Jan1996
Apr2004
Jul2012
Nov2020
Feb2029
May2037
Sep2045
Dec2053
Mar2062
Jul2070
Oct2078
Jan2087
May2095
Date
Actual
Smoothed
Predicted
95% Confidence limits
Start of multi-step forecasts (2002)

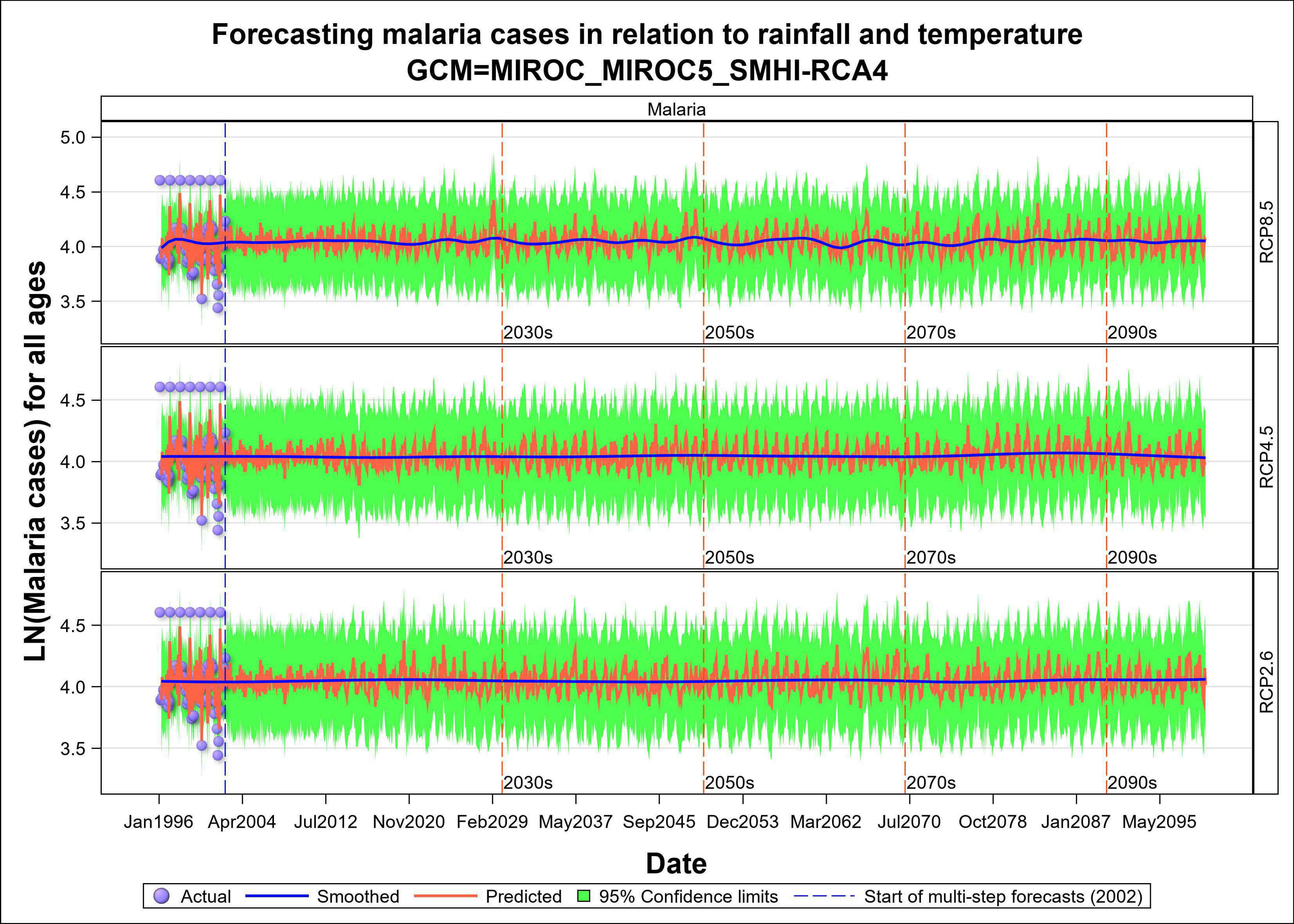
Forecasting malaria cases in relation to rainfall and temperature
GCM=MIROC_MIROC5_SMHI-RCA4
Malaria
RCP8.5
RCP4.5
RCP2.6
2030s
2050s
2070s
2090s
LN(Malaria cases) for all ages
5.0
4.5
4.0
3.5
Jan1996
Apr2004
Jul2012
Nov2020
Feb2029
May2037
Sep2045
Dec2053
Mar2062
Jul2070
Oct2078
Jan2087
May2095
Date
Actual
Smoothed
Predicted
95% Confidence limits
Start of multi-step forecasts (2002)

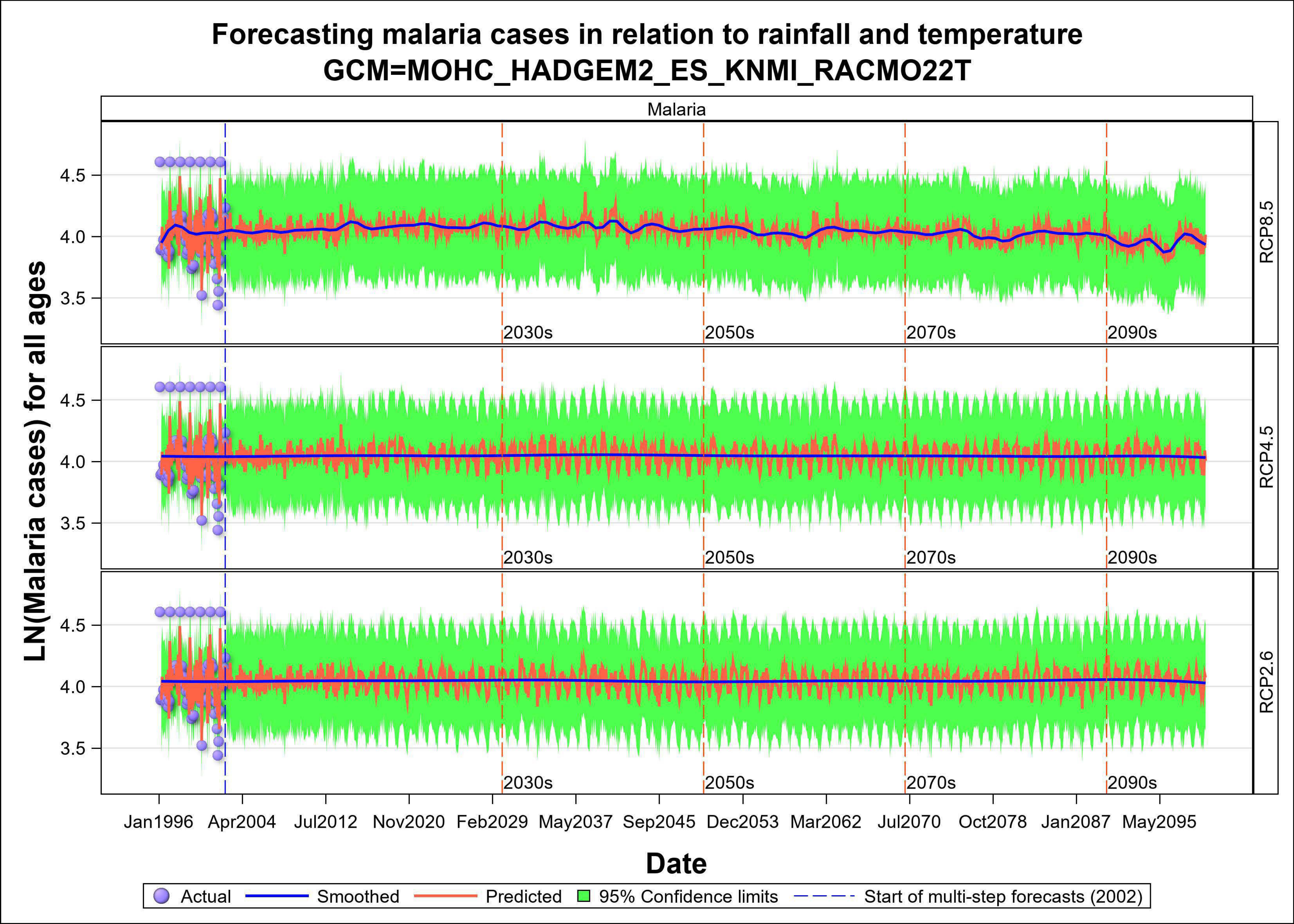

Forecasting malaria cases in relation to rainfall and temperature
GCM=MOHC_HADGEM2_ES_KNMI_RACMO22T
Malaria
RCP8.5
RCP4.5
RCP2.6
LN(Malaria cases) for all ages
4.5
4.0
3.5
2030s
2050s
2070s
2090s
Jan1996
Apr2004
Jul2012
Nov2020
Feb2029
May2037
Sep2045
Dec2053
Mar2062
Jul2070
Oct2078
Jan2087
May2095
Date
Actual
Smoothed
Predicted
95% Confidence limits
Start of multi-step forecasts (2002)

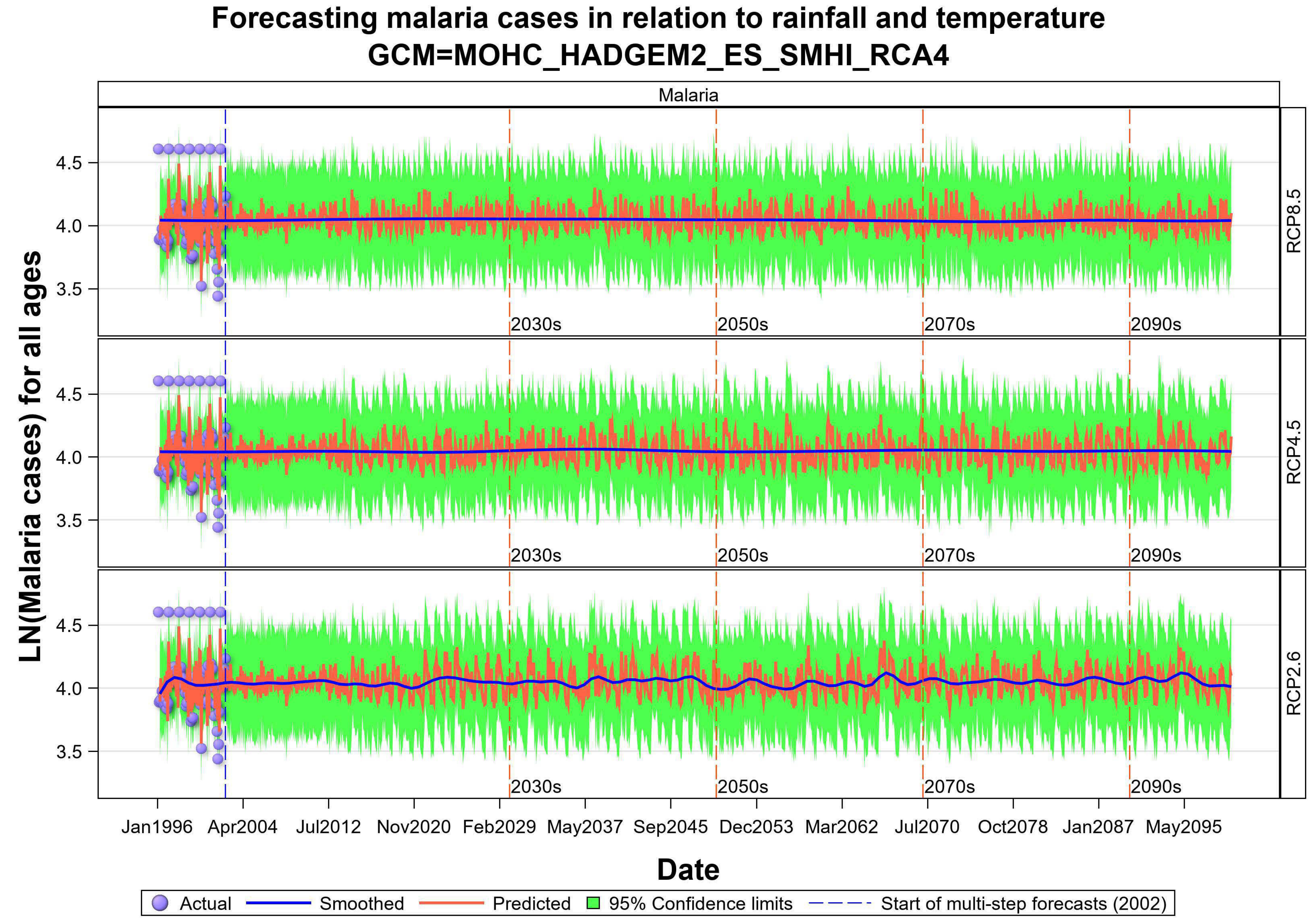
Forecasting malaria cases in relation to rainfall and temperature
GCM=MOHC_HADGEM2_ES_SMHI_RCA4
Malaria
RCP8.5
RCP4.5
RCP2.6
2030s
2050s
2070s
2090s
4.5
4.0
3.5
LN(Malaria cases) for all ages
Jan1996
Apr2004
Jul2012
Nov2020
Feb2029
May2037
Sep2045
Dec2053
Mar2062
Jul2070
Oct2078
Jan2087
May2095
Date
Actual
Smoothed
Predicted
95% Confidence limits
Start of multi-step forecasts (2002)

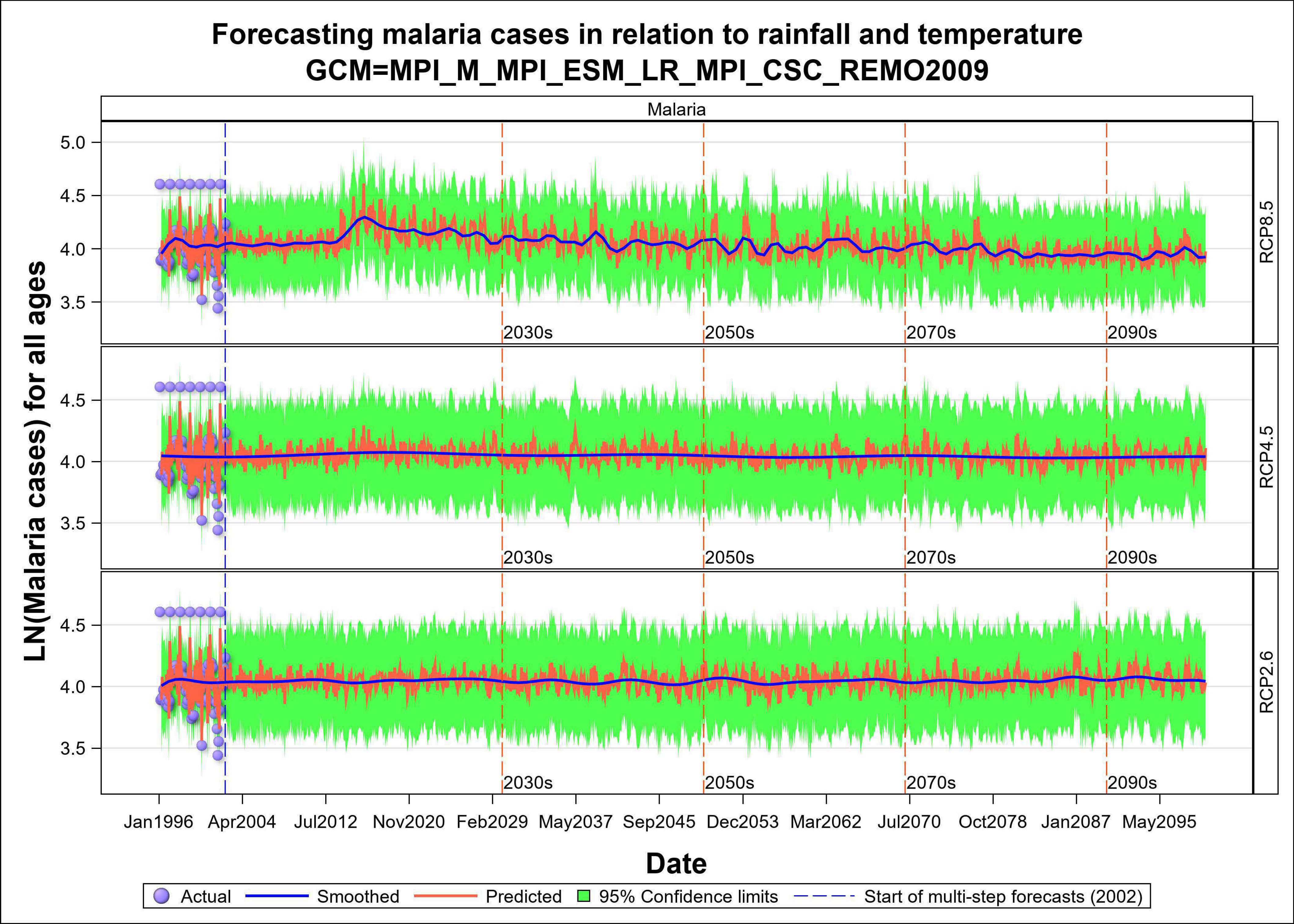
Forecasting malaria cases in relation to rainfall and temperature
GCM=MPI_M_MPI_ESM_LR_MPI_CSC_REMO2009
Malaria
LN(Malaria cases) for all ages
RCP8.5
RCP4.5
RCP2.6
2030s
2050s
2070s
2090s
5.0
4.5
4.0
3.5
Jan1996
Apr2004
Jul2012
Nov2020
Feb2029
May2037
Sep2045
Dec2053
Mar2062
Jul2070
Oct2078
Jan2087
May2095
Date
Actual
Smoothed
Predicted
95% Confidence limits
Start of multi-step forecasts (2002)

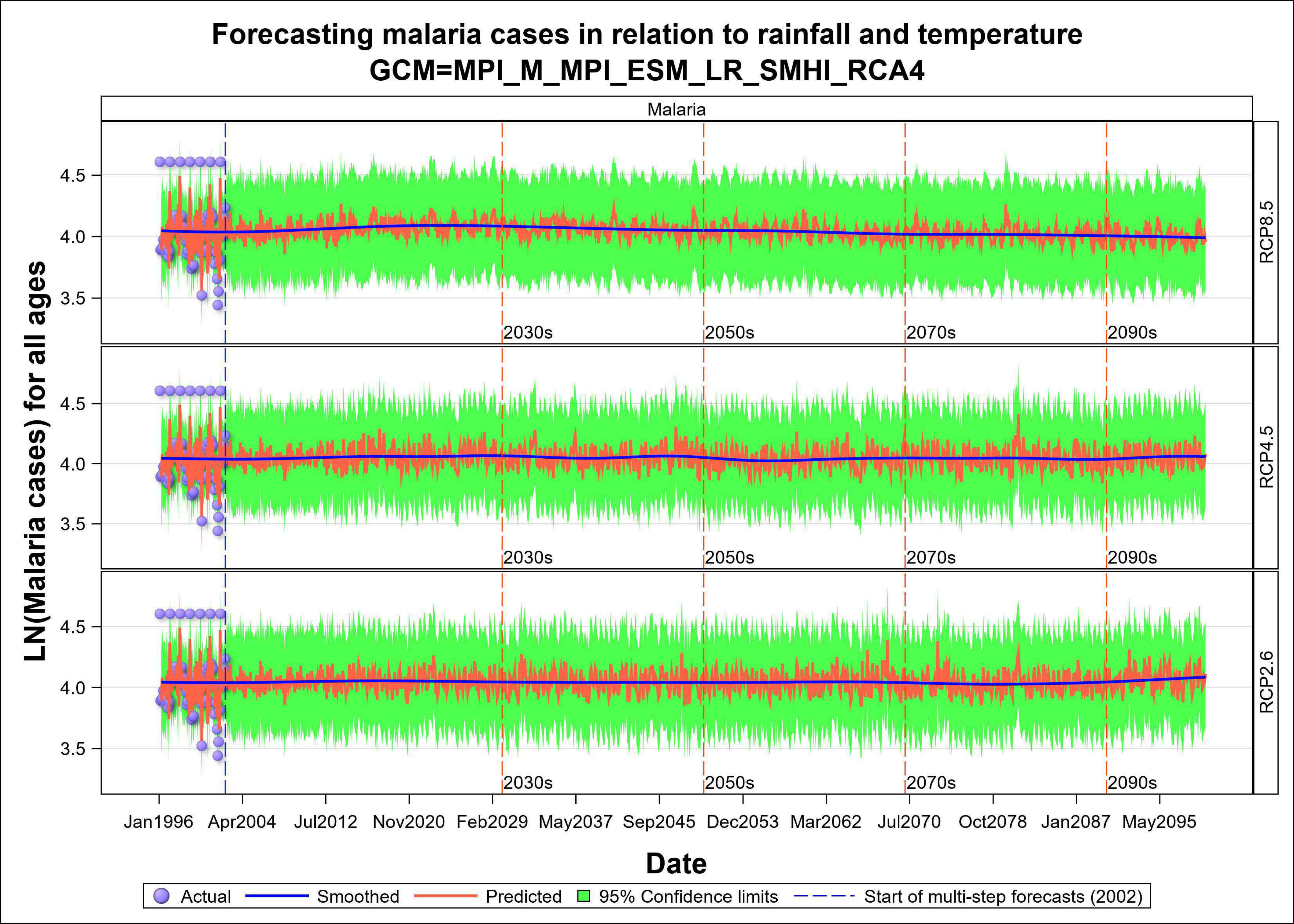

Forecasting malaria cases in relation to rainfall and temperature
GCM=MPI_M_MPI_ESM_LR_SMHI_RCA4
Malaria
RCP8.5
RCP4.5
RCP2.6
2030s
2050s
2070s
2090s
4.5
4.0
3.5
LN(Malaria cases) for all ages
Jan1996
Apr2004
Jul2012
Nov2020
Feb2029
May2037
Sep2045
Dec2053
Mar2062
Jul2070
Oct2078
Jan2087
May2095
Date
Actual
Smoothed
Predicted
95% Confidence limits
Start of multi-step forecasts (2002)

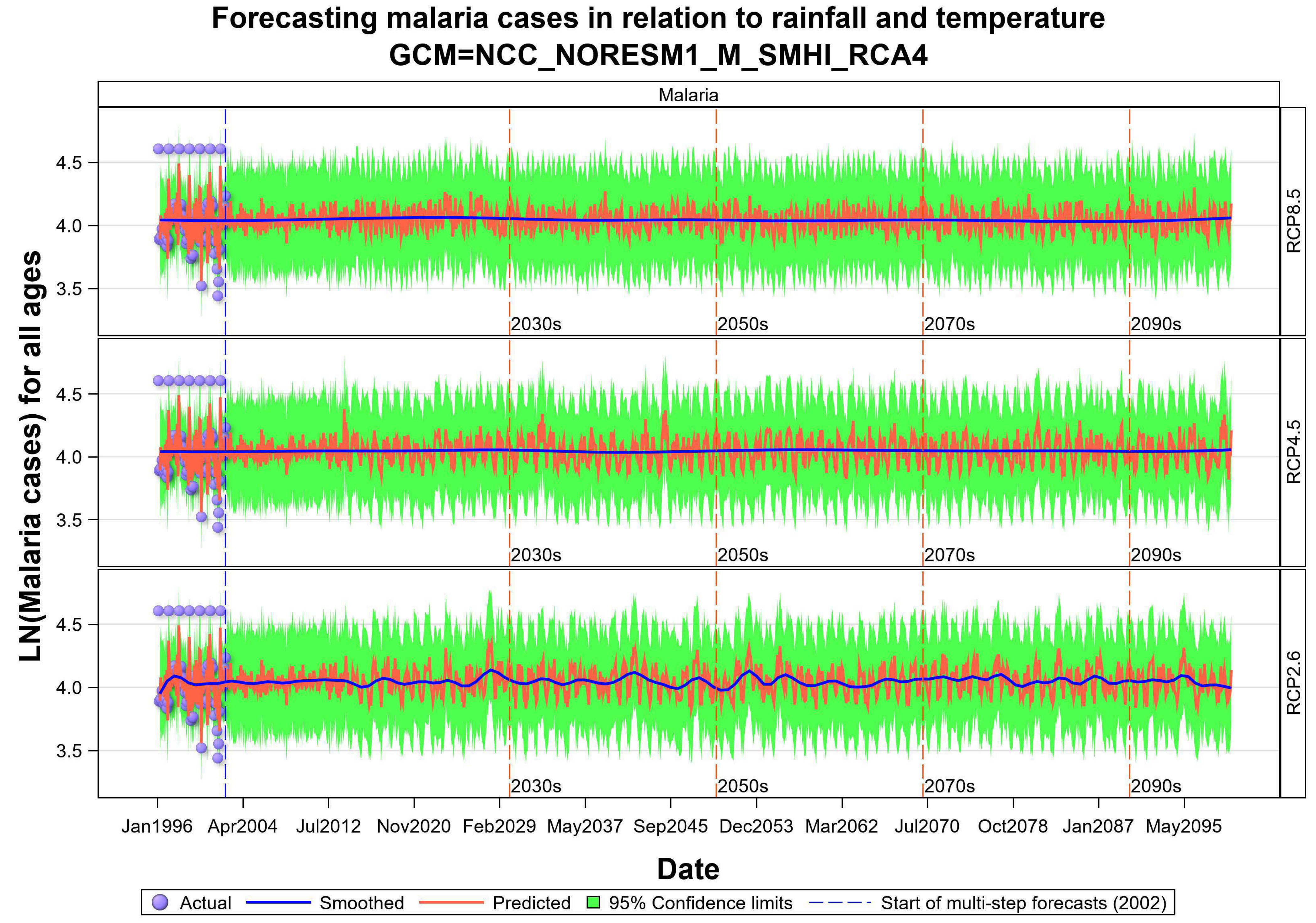

Forecasting malaria cases in relation to rainfall and temperature
GCM=NCC_NORESM1_M_SMHI_RCA4
Malaria
RCP8.5
RCP4.5
RCP2.6
LN(Malaria cases) for all ages
4.5
4.0
3.5
2030s
2050s
2070s
2090s
Jan1996
Apr2004
Jul2012
Nov2020
Feb2029
May2037
Sep2045
Dec2053
Mar2062
Jul2070
Oct2078
Jan2087
May2095
Date
Actual
Smoothed
Predicted
95% Confidence limits
Start of multi-step forecasts (2002)

SI22

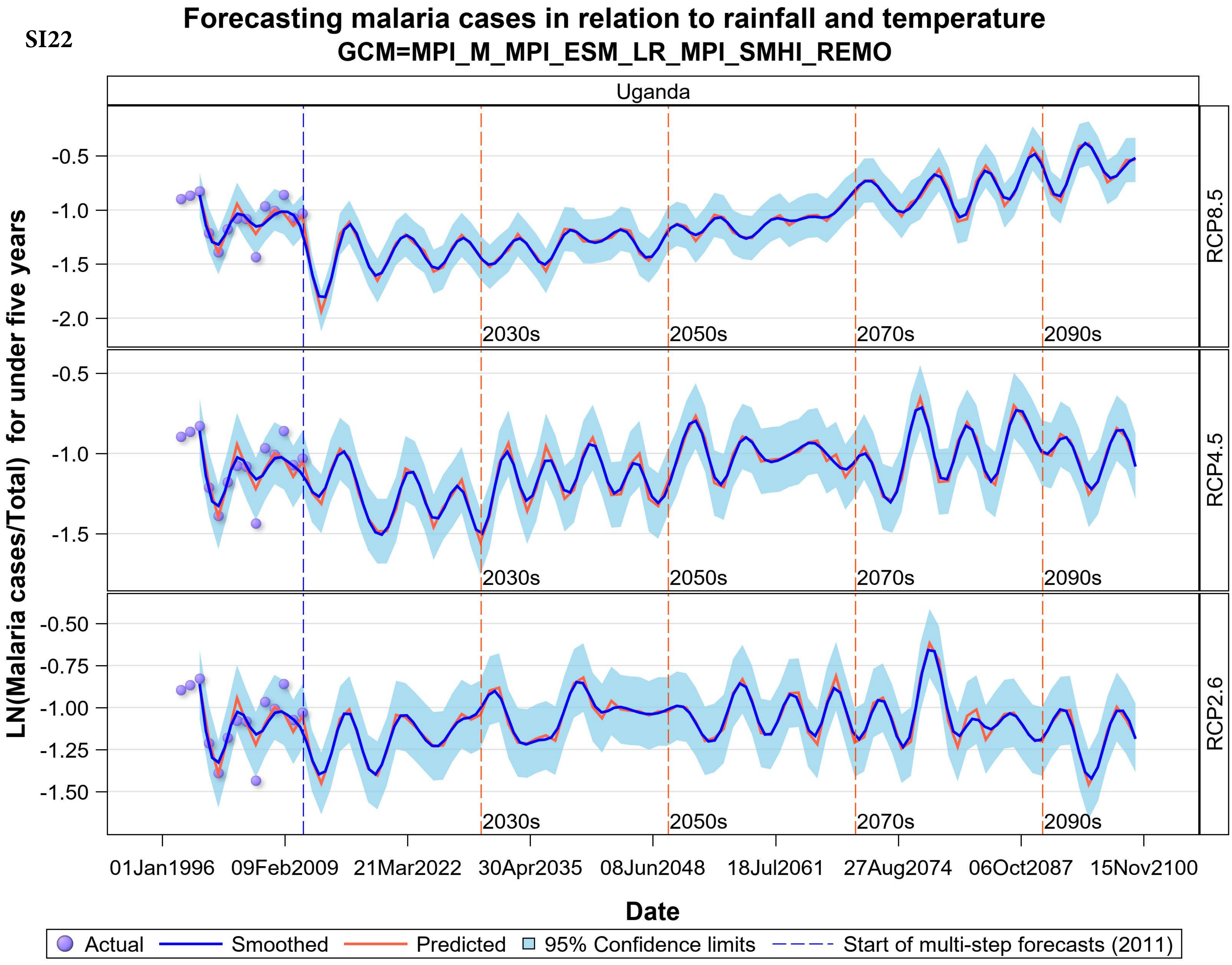

Forecasting malaria cases in relation to rainfall and temperature
GCM=MPI_M_MPI_ESM_LR_MPI_SMHI_REMO
Uganda
RCP8.5
RCP4.5
RCP2.6
2030s
2050s
2070s
2090s
LN(Malaria cases/Total) for under five years
-0.5
-1.0
-1.5
-2.0
-0.50
-0.75
-1.00
-1.25
-1.50
01Jan1996
09Feb2009
21Mar2022
30Apr2035
08Jun2048
18Jul2061
27Aug2074
06Oct2087
15Nov2100
Date
Actual
Smoothed
Predicted
95% Confidence limits
Start of multi-step forecasts (2011)

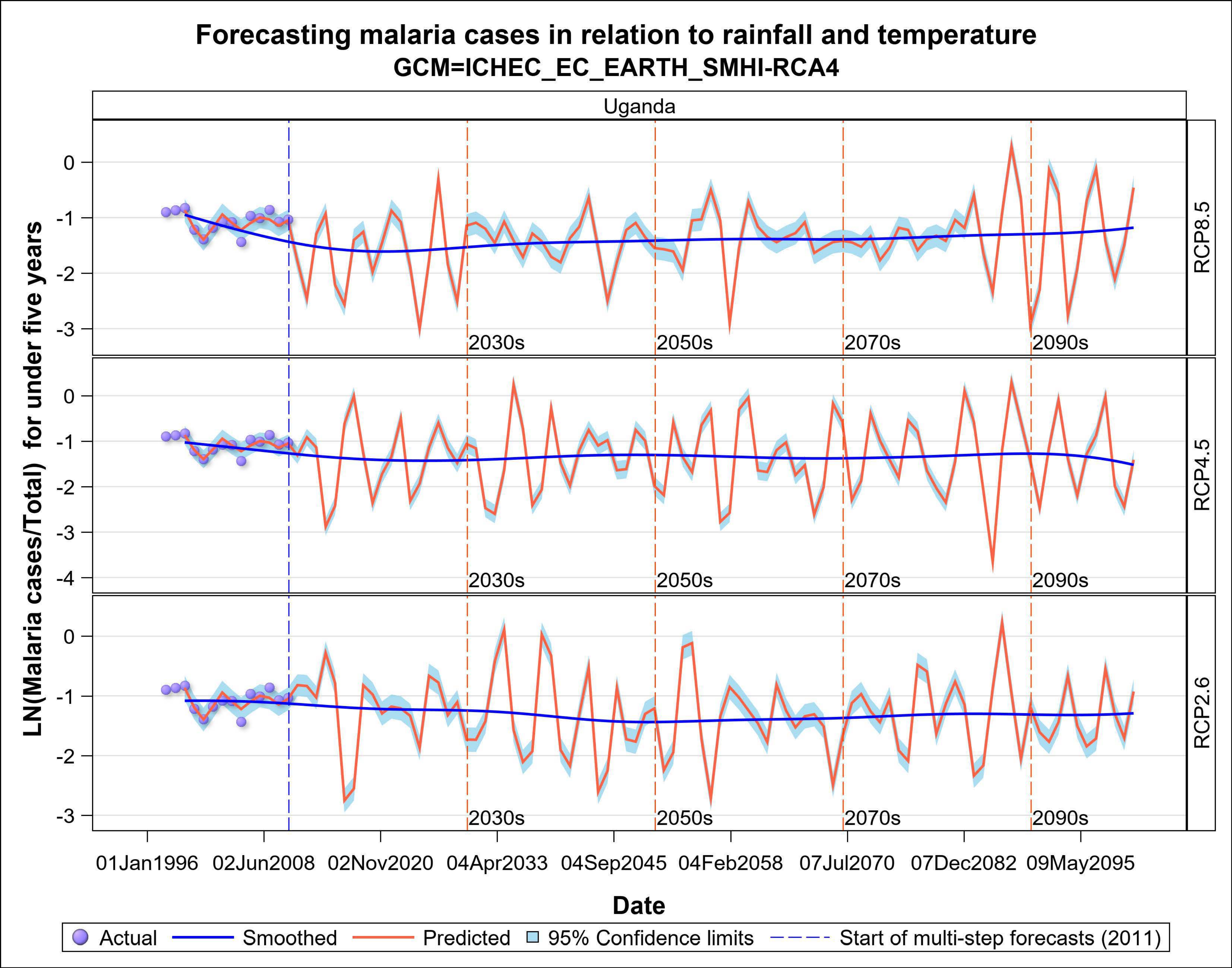
Forecasting malaria cases in relation to rainfall and temperature
GCM=ICHEC_EC_EARTH_SMHI-RCA4
Uganda
RCP8.5
RCP4.5
RCP2.6
2030s
2050s
2070s
2090s
LN(Malaria cases/Total) for under five years
0
-1
-2
-3
-4
01Jan1996
02Jun2008
02Nov2020
04Apr2033
04Sep2045
04Feb2058
07Jul2070
07Dec2082
09May2095
Date
Actual
Smoothed
Predicted
95% Confidence limits
Start of multi-step forecasts (2011)

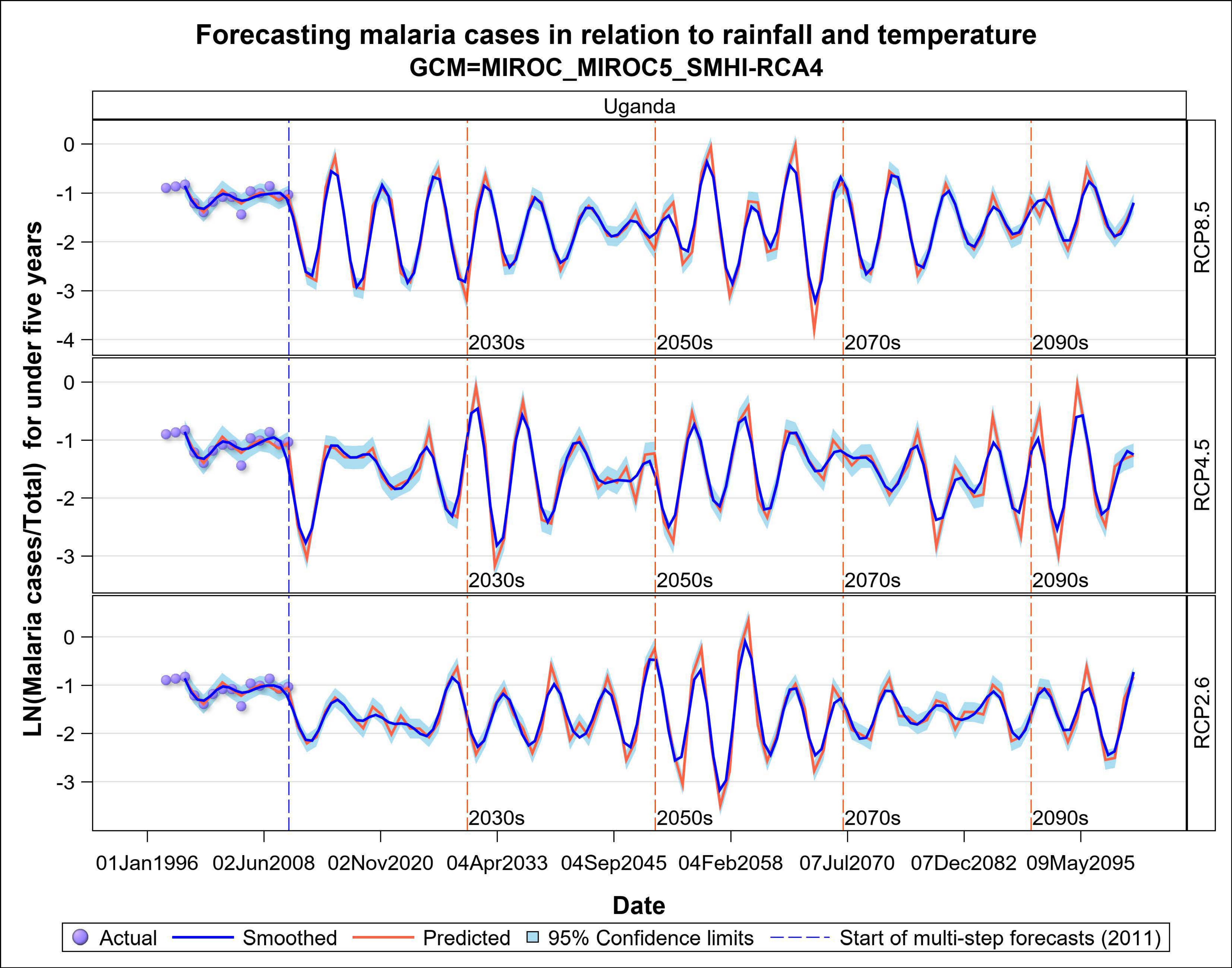

Forecasting malaria cases in relation to rainfall and temperature
GCM=MIROC_MIROC5_SMHI-RCA4
Uganda
RCP8.5
RCP4.5
RCP2.6
LN(Malaria cases/Total) for under five years
0
-1
-2
-3
-4
2030s
2050s
2070s
2090s
01Jan1996
02Jun2008
02Nov2020
04Apr2033
04Sep2045
04Feb2058
07Jul2070
07Dec2082
09May2095
Date
Actual
Smoothed
Predicted
95% Confidence limits
Start of multi-step forecasts (2011)

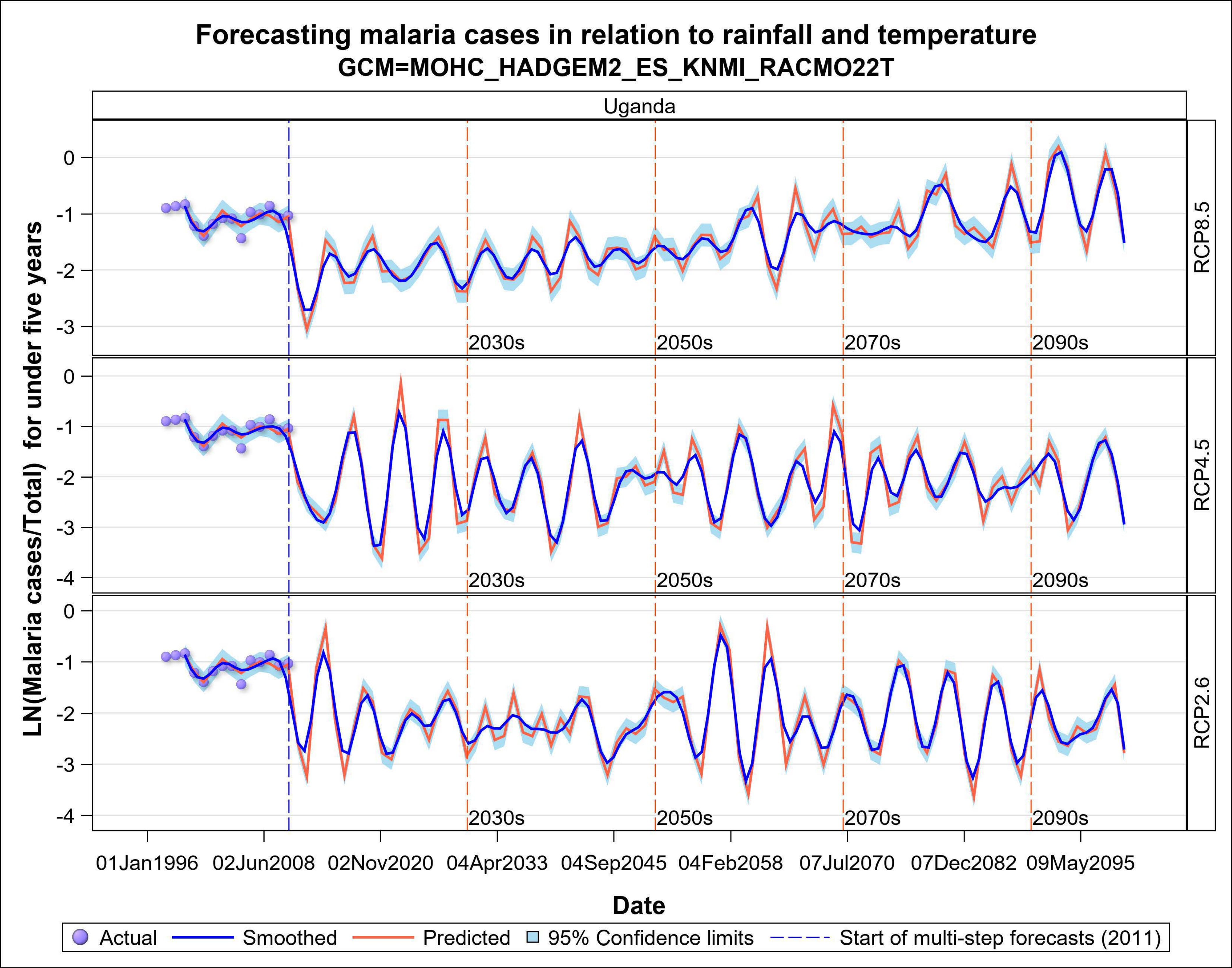

Forecasting malaria cases in relation to rainfall and temperature
GCM=MOHC_HADGEM2_ES_KNMI_RACMO22T
Uganda
RCP8.5
RCP4.5
RCP2.6
2030s
2050s
2070s
2090s
0
-1
-2
-3
-4
LN(Malaria cases/Total) for under five years
01Jan1996
02Jun2008
02Nov2020
04Apr2033
04Sep2045
04Feb2058
07Jul2070
07Dec2082
09May2095
Date
Actual
Smoothed
Predicted
95% Confidence limits
Start of multi-step forecasts (2011)

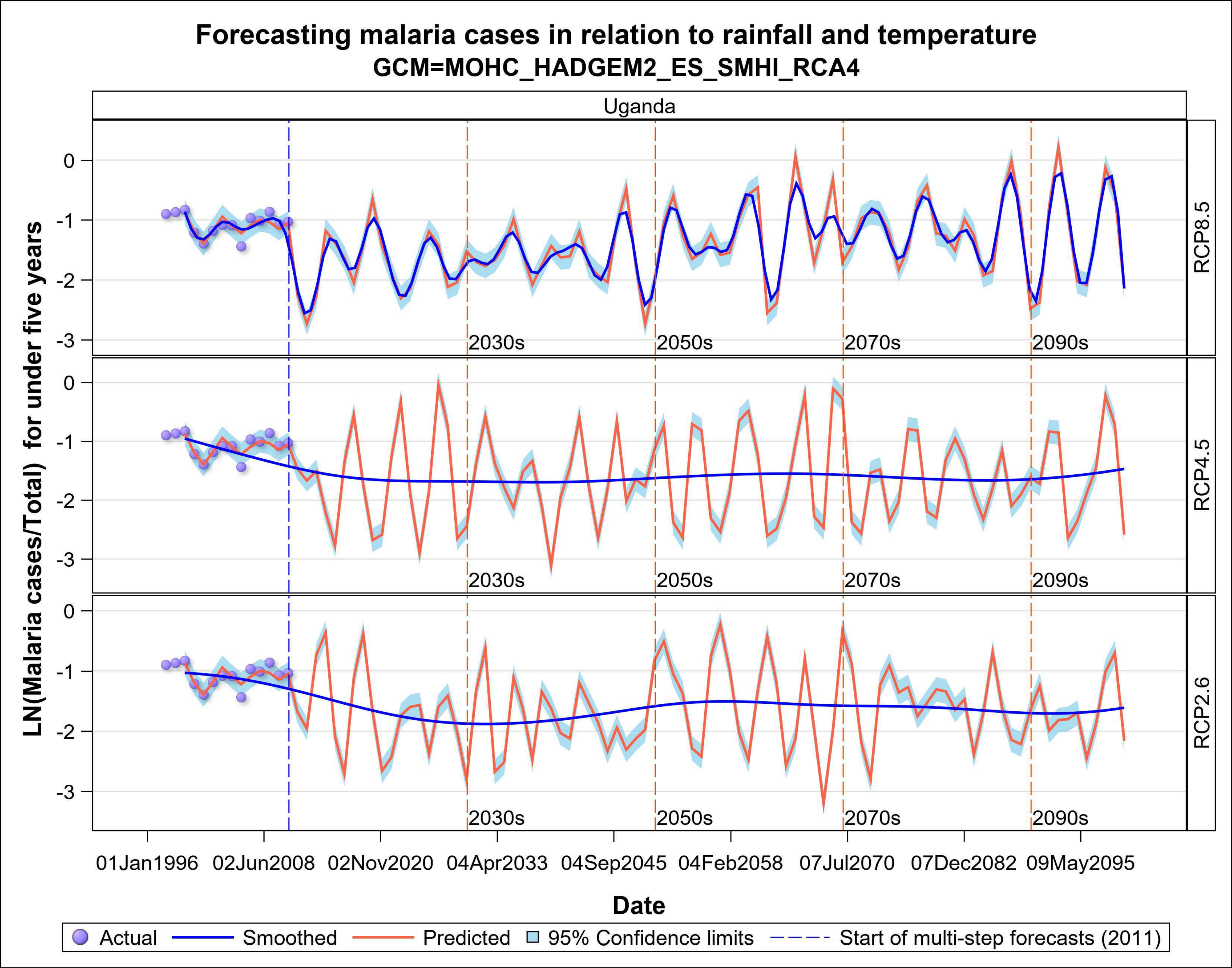

Forecasting malaria cases in relation to rainfall and temperature
GCM=MOHC_HADGEM2_ES_SMHI_RCA4
Uganda
RCP8.5
RCP4.5
RCP2.6
LN(Malaria cases/Total) for under five years
0
-1
-2
-3
2030s
2050s
2070s
2090s
01Jan1996
02Jun2008
02Nov2020
04Apr2033
04Sep2045
04Feb2058
07Jul2070
07Dec2082
09May2095
Date
Actual
Smoothed
Predicted
95% Confidence limits
Start of multi-step forecasts (2011)

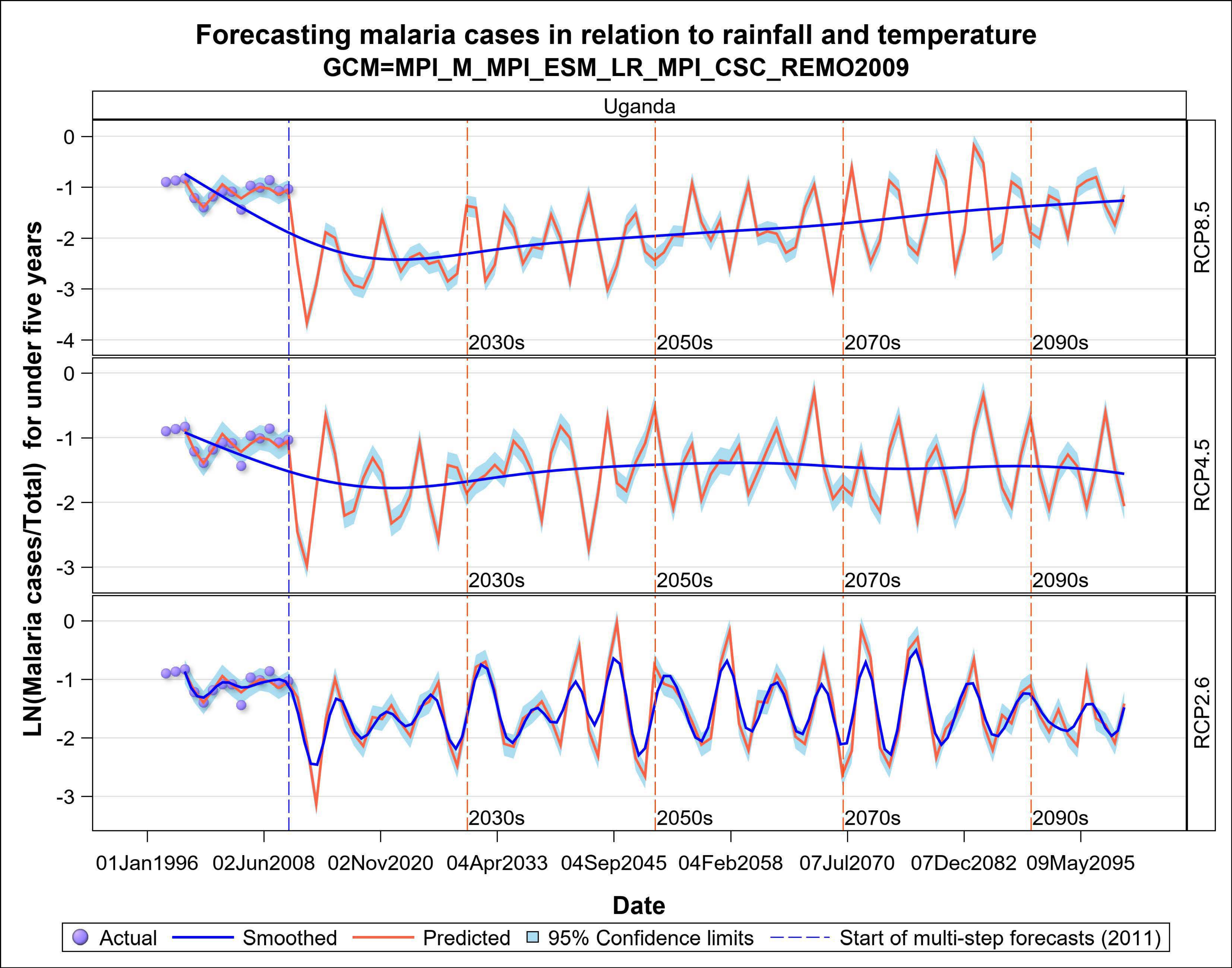

Forecasting malaria cases in relation to rainfall and temperature
GCM=MPI_M_MPI_ESM_LR_MPI_CSC_REMO2009
Uganda
RCP8.5
RCP4.5
RCP2.6
2030s
2050s
2070s
2090s
LN(Malaria cases/Total) for under five years
0
-1
-2
-3
-4
01Jan1996
02Jun2008
02Nov2020
04Apr2033
04Sep2045
04Feb2058
07Jul2070
07Dec2082
09May2095
Date
Actual
Smoothed
Predicted
95% Confidence limits
Start of multi-step forecasts (2011)

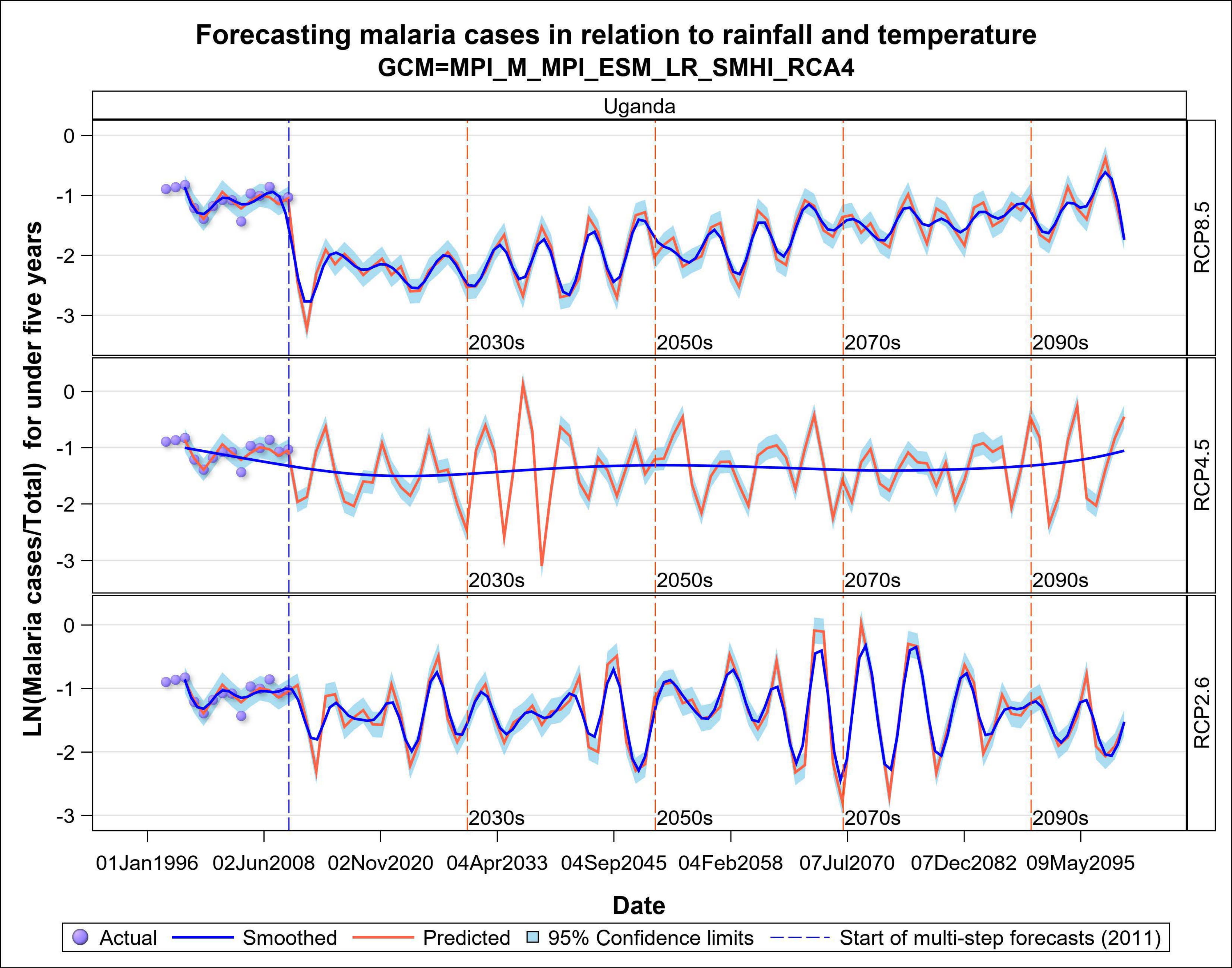

Forecasting malaria cases in relation to rainfall and temperature
GCM=MPI_M_MPI_ESM_LR_SMHI_RCA4
Uganda
RCP8.5
RCP4.5
RCP2.6
0
-1
-2
-3
2030s
2050s
2070s
2090s
LN(Malaria cases/Total) for under five years
01Jan1996
02Jun2008
02Nov2020
04Apr2033
04Sep2045
04Feb2058
07Jul2070
07Dec2082
09May2095
Date
Actual
Smoothed
Predicted
95% Confidence limits
Start of multi-step forecasts (2011)

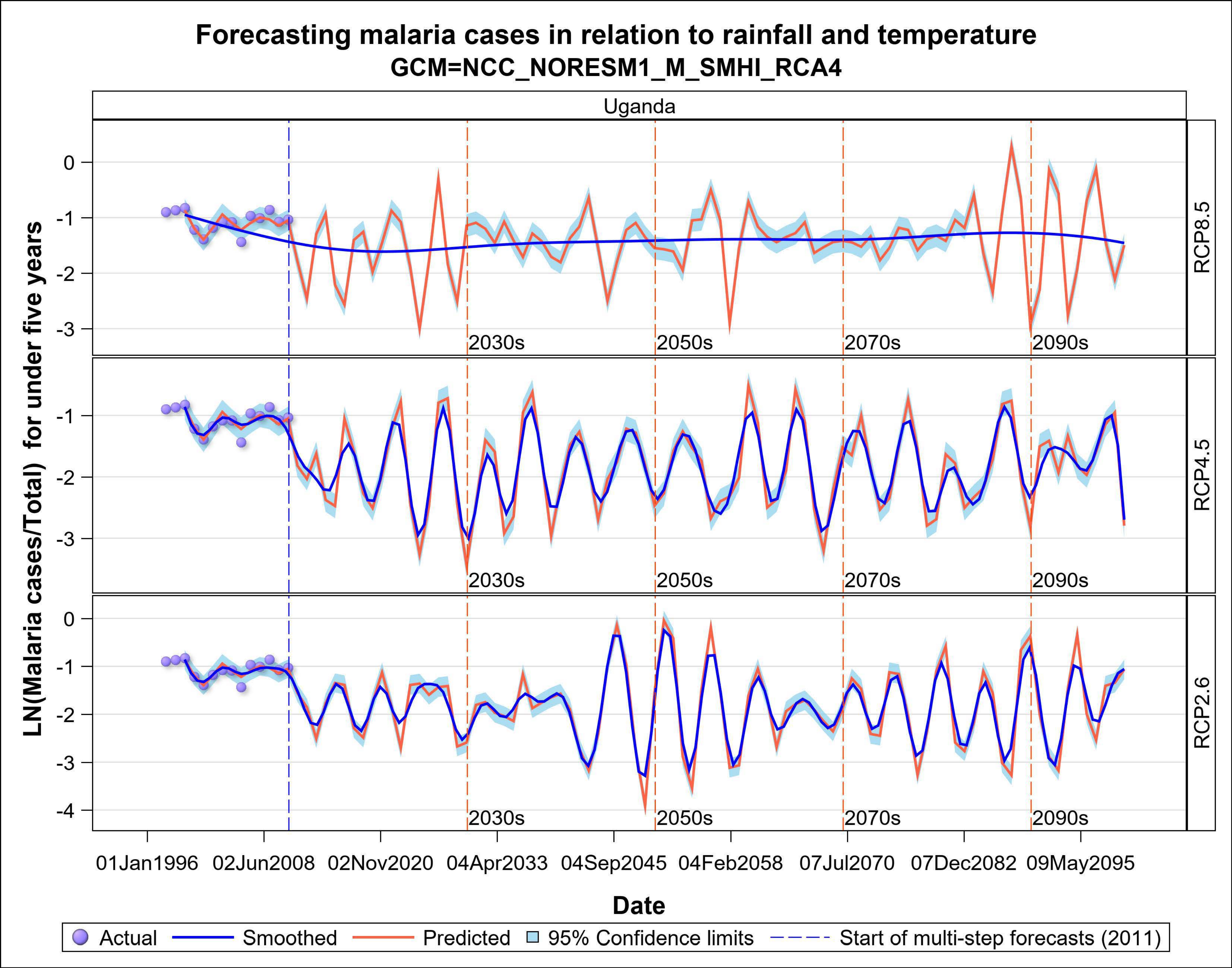

Forecasting malaria cases in relation to rainfall and temperature
GCM=NCC_NORESM1_M_SMHI_RCA4
Uganda
RCP8.5
RCP4.5
RCP2.6
2030s
2050s
2070s
2090s
LN(Malaria cases/Total) for under five years
0
-1
-2
-3
-4
01Jan1996
02Jun2008
02Nov2020
04Apr2033
04Sep2045
04Feb2058
07Jul2070
07Dec2082
09May2095
Date
Actual
Smoothed
Predicted
95% Confidence limits
Start of multi-step forecasts (2011)

SI23

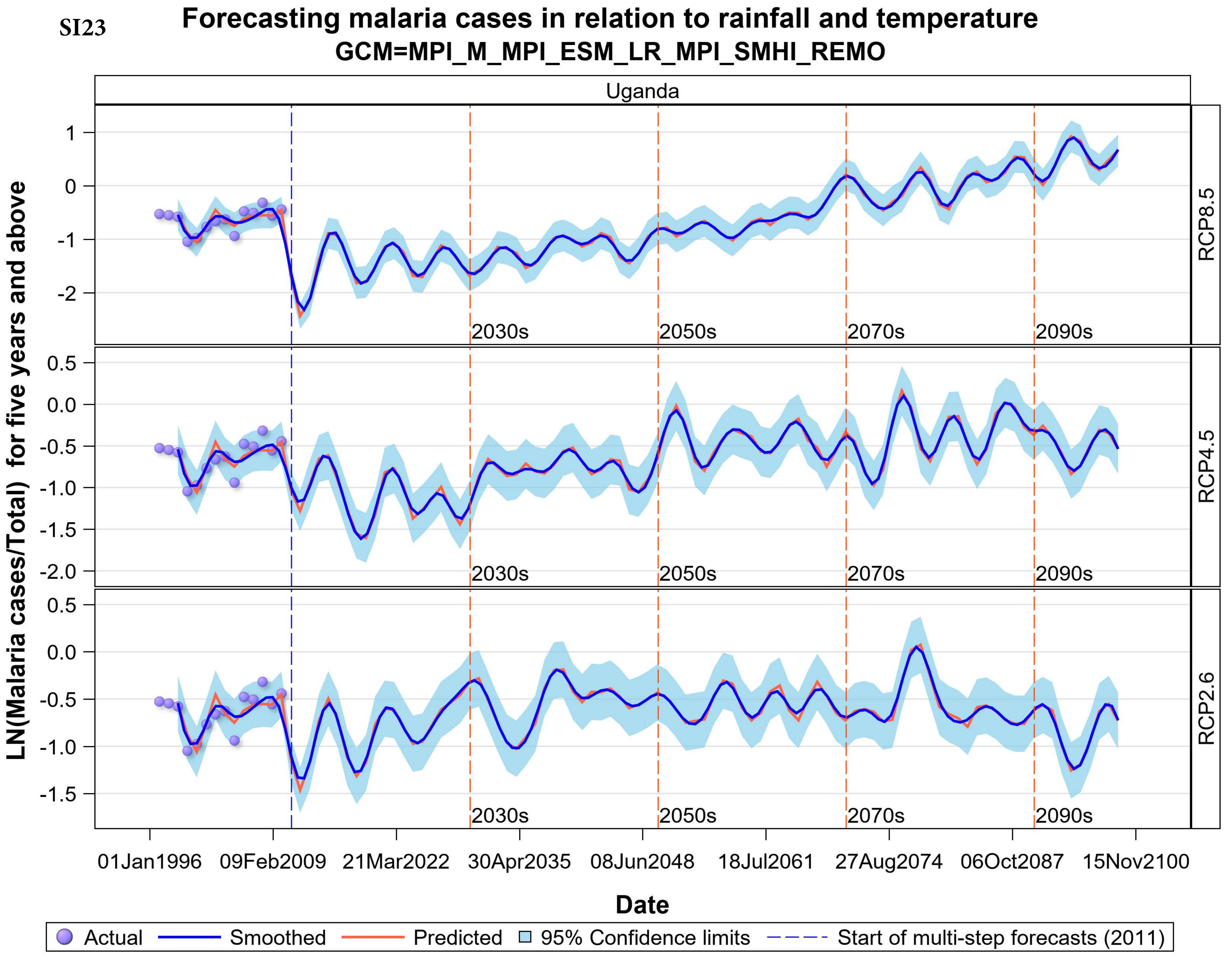

Forecasting malaria cases in relation to rainfall and temperature
GCM=MPI_M_MPI_ESM_LR_MPI_SMHI_REMO
Uganda
RCP8.5
RCP4.5
RCP2.6
2030s
2050s
2070s
2090s
LN(Malaria cases/Total) for five years and above
1
0
-1
-2
0.5
0.0
-0.5
-1.0
-1.5
-2.0
01Jan1996
09Feb2009
21Mar2022
30Apr2035
08Jun2048
18Jul2061
27Aug2074
06Oct2087
15Nov2100
Date
Actual
Smoothed
Predicted
95% Confidence limits
Start of multi-step forecasts (2011)

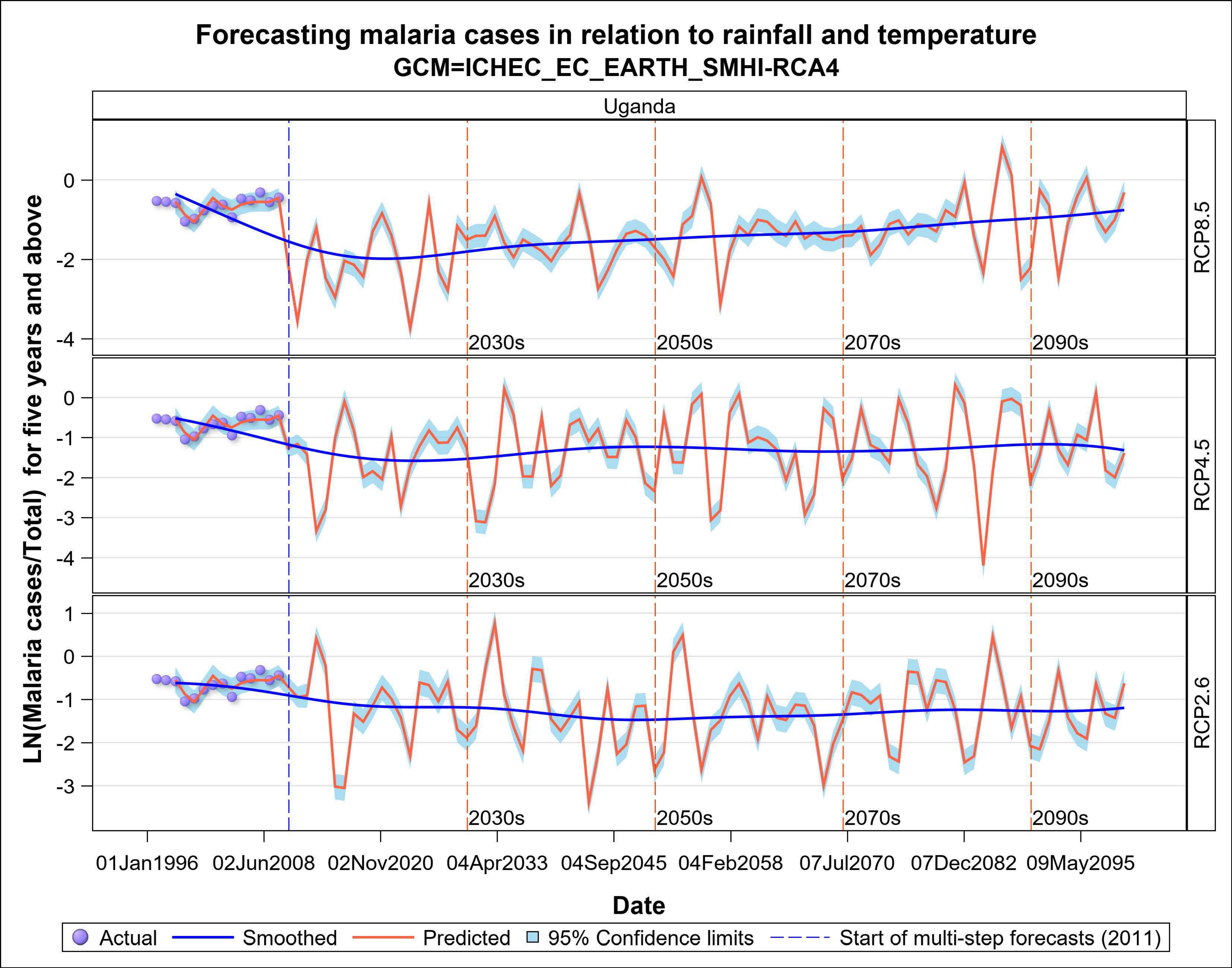

Forecasting malaria cases in relation to rainfall and temperature
GCM=ICHEC_EC_EARTH_SMHI-RCA4
Uganda
RCP8.5
RCP4.5
RCP2.6
LN(Malaria cases/Total) for five years and above
2030s
2050s
2070s
2090s
01Jan1996
02Jun2008
02Nov2020
04Apr2033
04Sep2045
04Feb2058
07Jul2070
07Dec2082
09May2095
Date
Actual
Smoothed
Predicted
95% Confidence limits
Start of multi-step forecasts (2011)

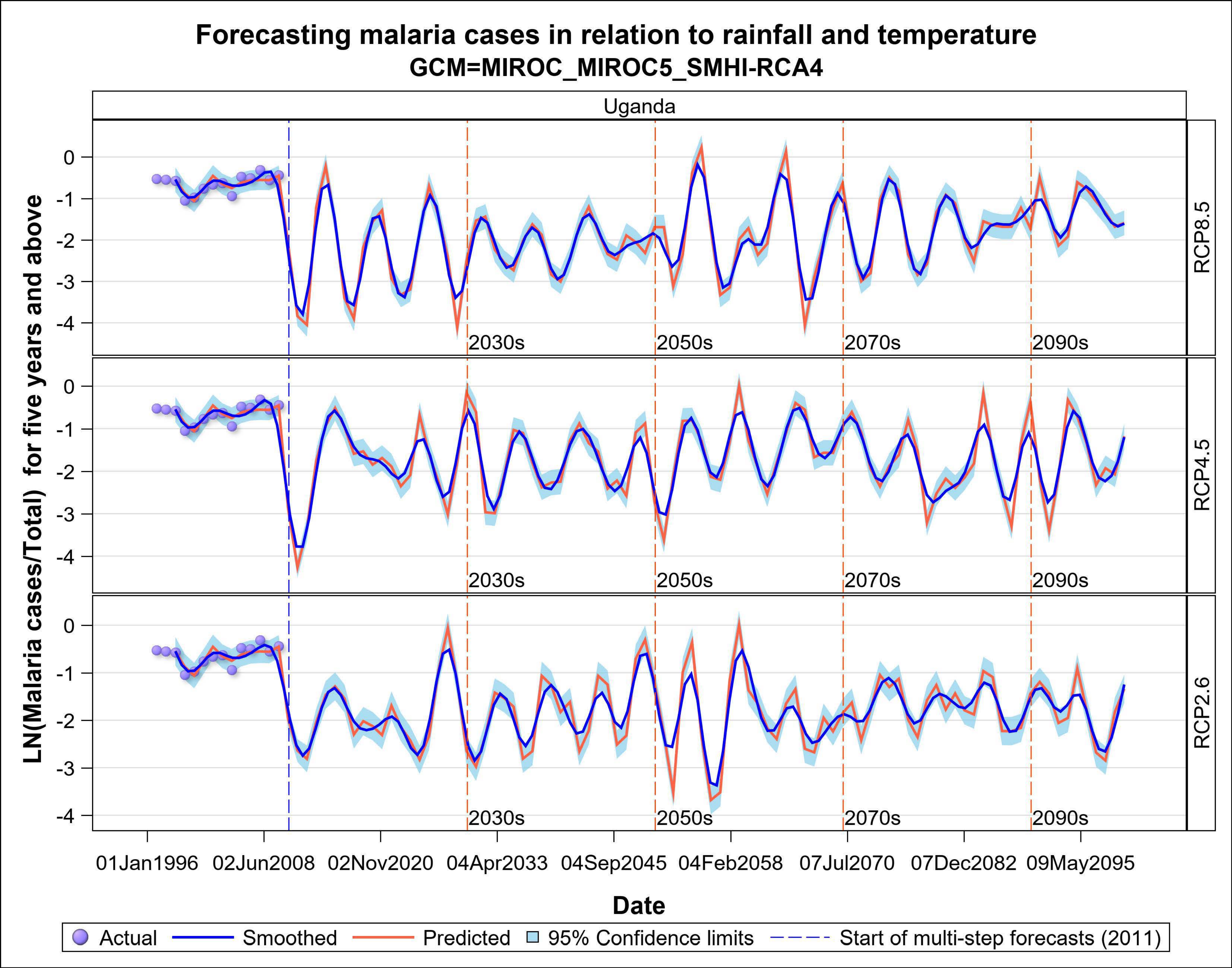

Forecasting malaria cases in relation to rainfall and temperature
GCM=MIROC_MIROC5_SMHI-RCA4
Uganda
RCP8.5
RCP4.5
RCP2.6
2030s
2050s
2070s
2090s
0
-1
-2
-3
-4
LN(Malaria cases/Total) for five years and above
01Jan1996
02Jun2008
02Nov2020
04Apr2033
04Sep2045
04Feb2058
07Jul2070
07Dec2082
09May2095
Date
Actual
Smoothed
Predicted
95% Confidence limits
Start of multi-step forecasts (2011)

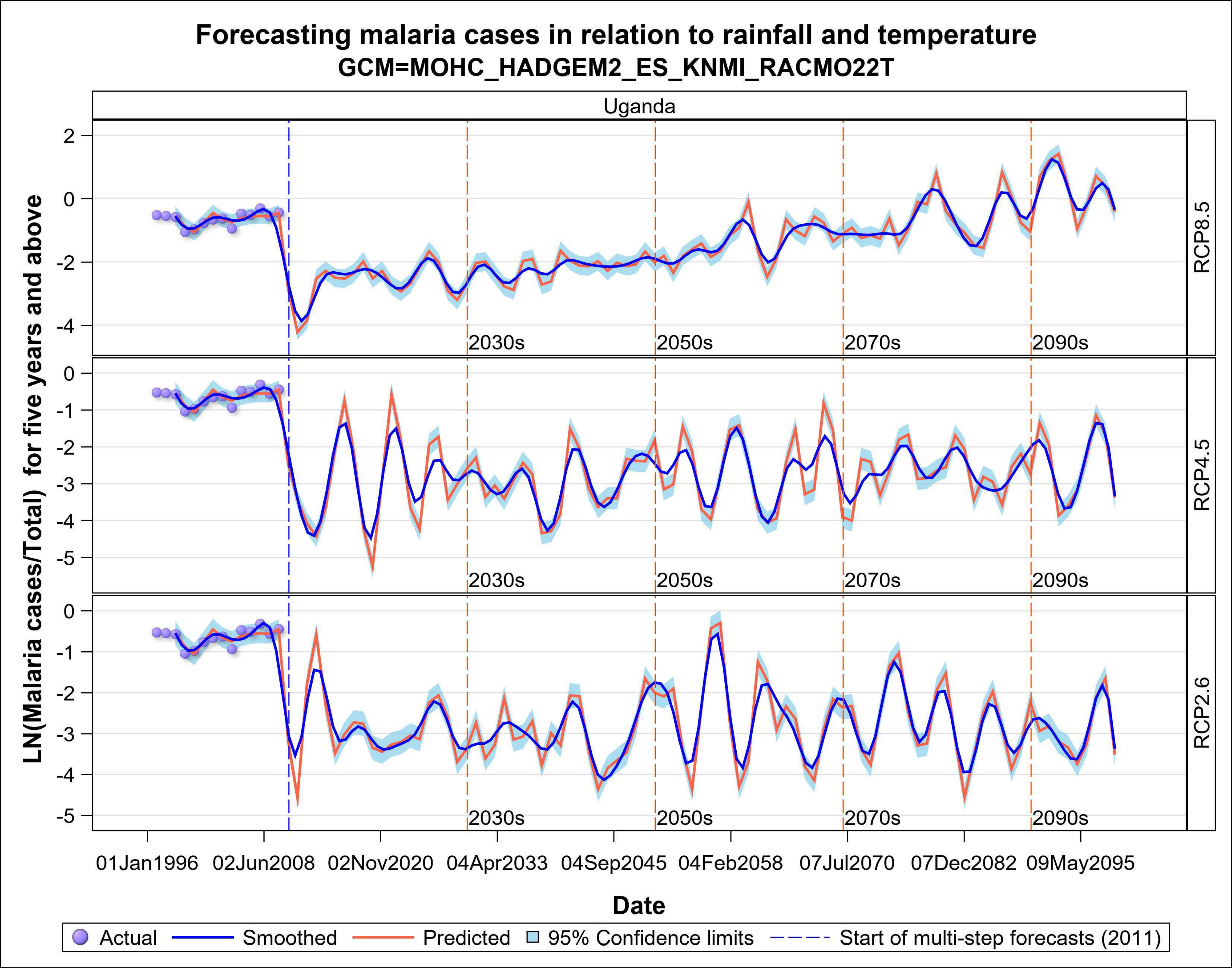
Forecasting malaria cases in relation to rainfall and temperature
GCM=MOHC_HADGEM2_ES_KNMI_RACMO22T
Uganda
RCP8.5
RCP4.5
RCP2.6
2030s
2050s
2070s
2090s
LN(Malaria cases/Total) for five years and above
Date
01Jan1996
02Jun2008
02Nov2020
04Apr2033
04Sep2045
04Feb2058
07Jul2070
07Dec2082
09May2095
Actual
Smoothed
Predicted
95% Confidence limits
Start of multi-step forecasts (2011)

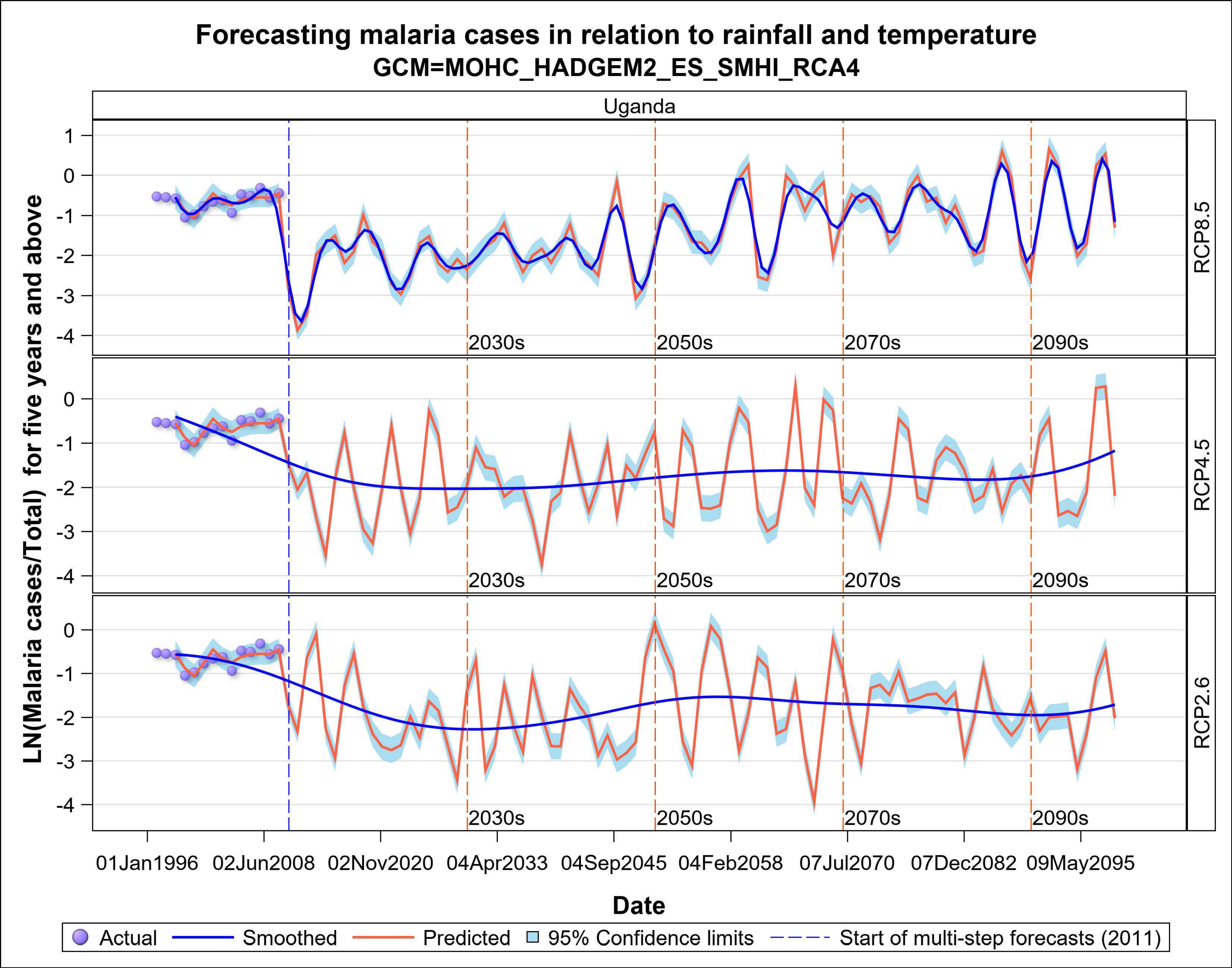

Forecasting malaria cases in relation to rainfall and temperature
GCM=MOHC_HADGEM2_ES_SMHI_RCA4
Uganda
RCP8.5
RCP4.5
RCP2.6
2030s
2050s
2070s
2090s
LN(Malaria cases/Total) for five years and above
1
0
-1
-2
-3
-4
01Jan1996
02Jun2008
02Nov2020
04Apr2033
04Sep2045
04Feb2058
07Jul2070
07Dec2082
09May2095
Date
Actual
Smoothed
Predicted
95% Confidence limits
Start of multi-step forecasts (2011)

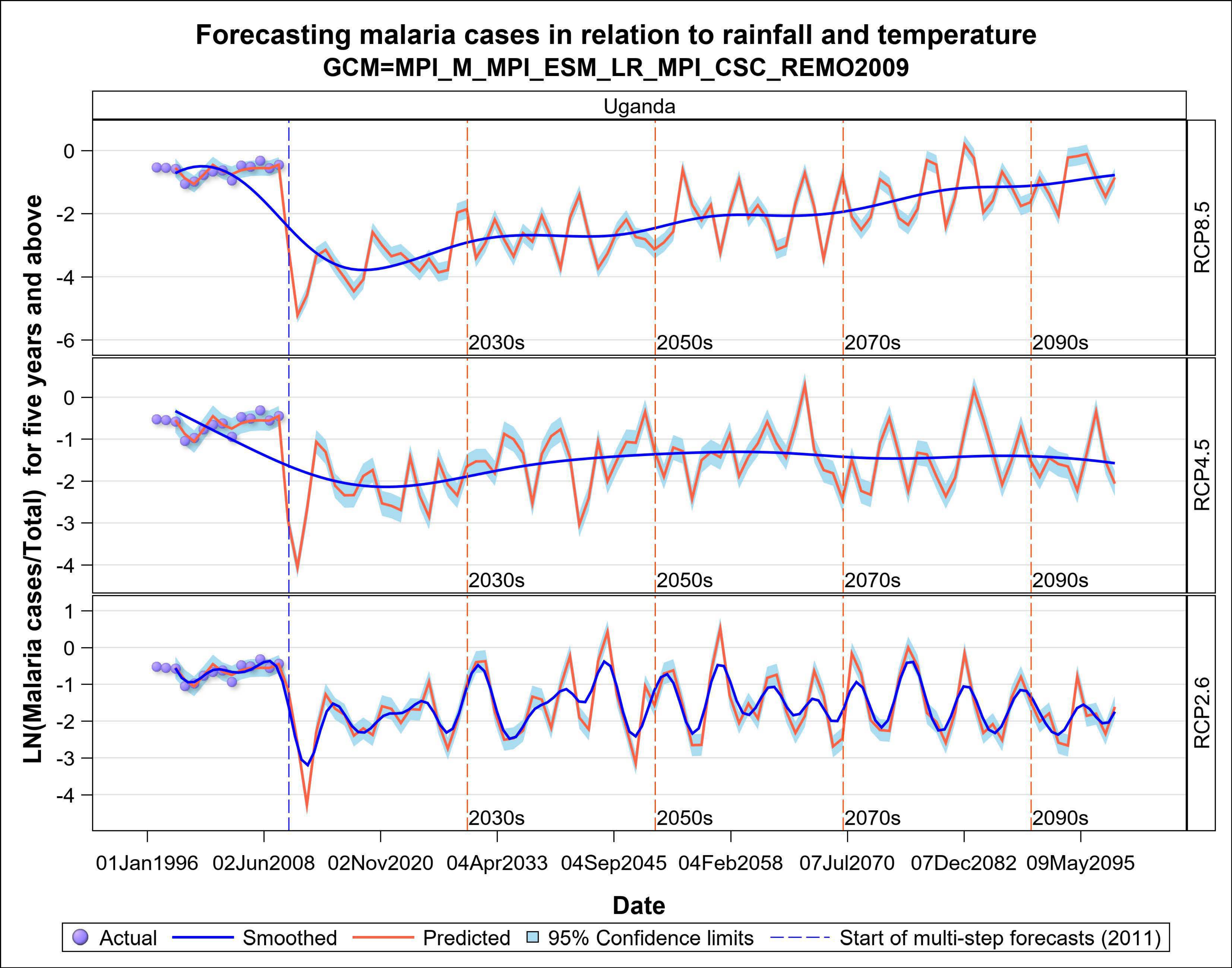

Forecasting malaria cases in relation to rainfall and temperature
GCM=MPI_M_MPI_ESM_LR_MPI_CSC_REMO2009
Uganda
RCP8.5
RCP4.5
RCP2.6
2030s
2050s
2070s
2090s
LN(Malaria cases/Total) for five years and above
01Jan1996
02Jun2008
02Nov2020
04Apr2033
04Sep2045
04Feb2058
07Jul2070
07Dec2082
09May2095
Date
Actual
Smoothed
Predicted
95% Confidence limits
Start of multi-step forecasts (2011)

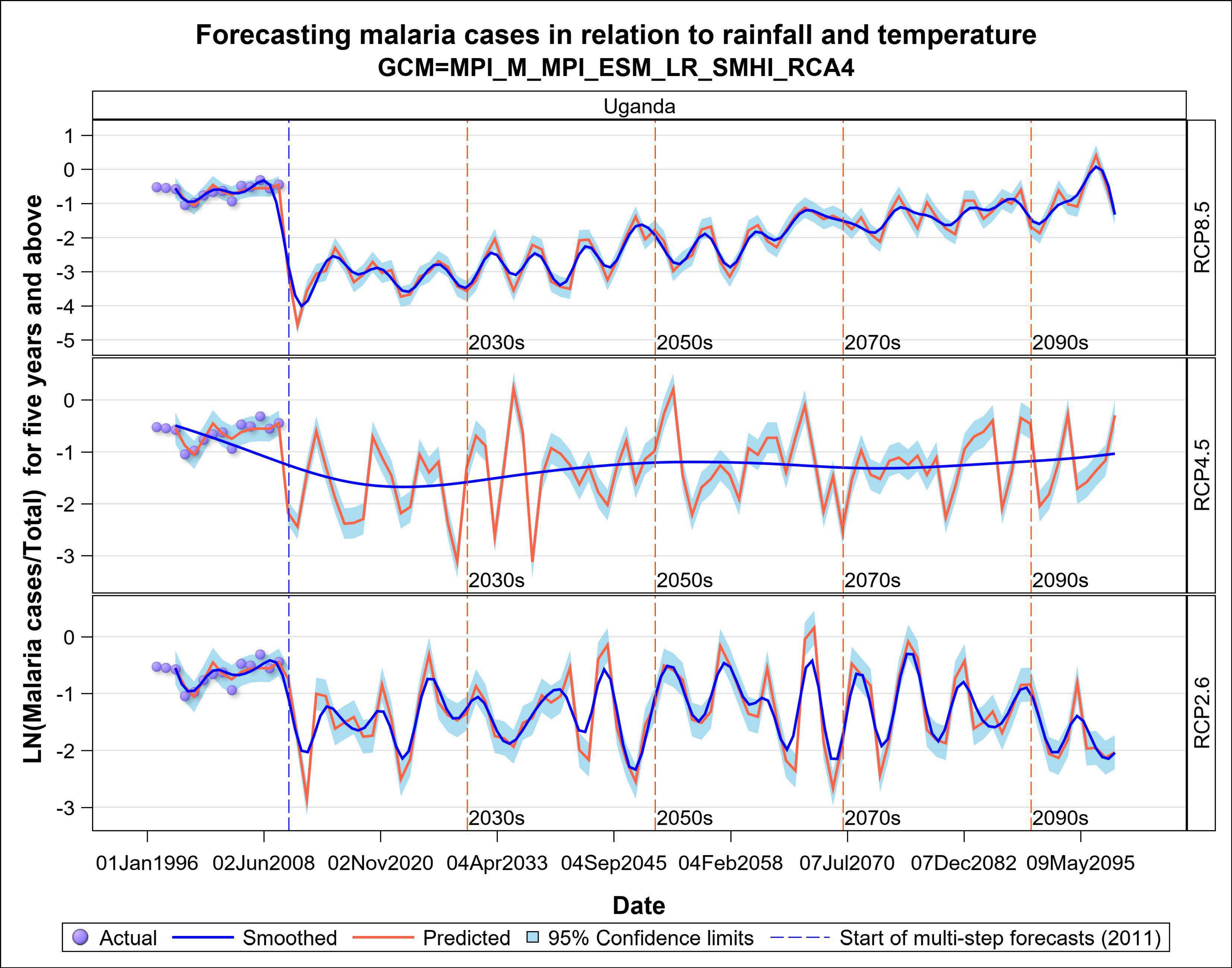
Forecasting malaria cases in relation to rainfall and temperature
GCM=MPI_M_MPI_ESM_LR_SMHI_RCA4
Uganda
RCP8.5
RCP4.5
RCP2.6
2030s
2050s
2070s
2090s
LN(Malaria cases/Total) for five years and above
1
0
-1
-2
-3
-4
-5
01Jan1996
02Jun2008
02Nov2020
04Apr2033
04Sep2045
04Feb2058
07Jul2070
07Dec2082
09May2095
Date
Actual
Smoothed
Predicted
95% Confidence limits
Start of multi-step forecasts (2011)

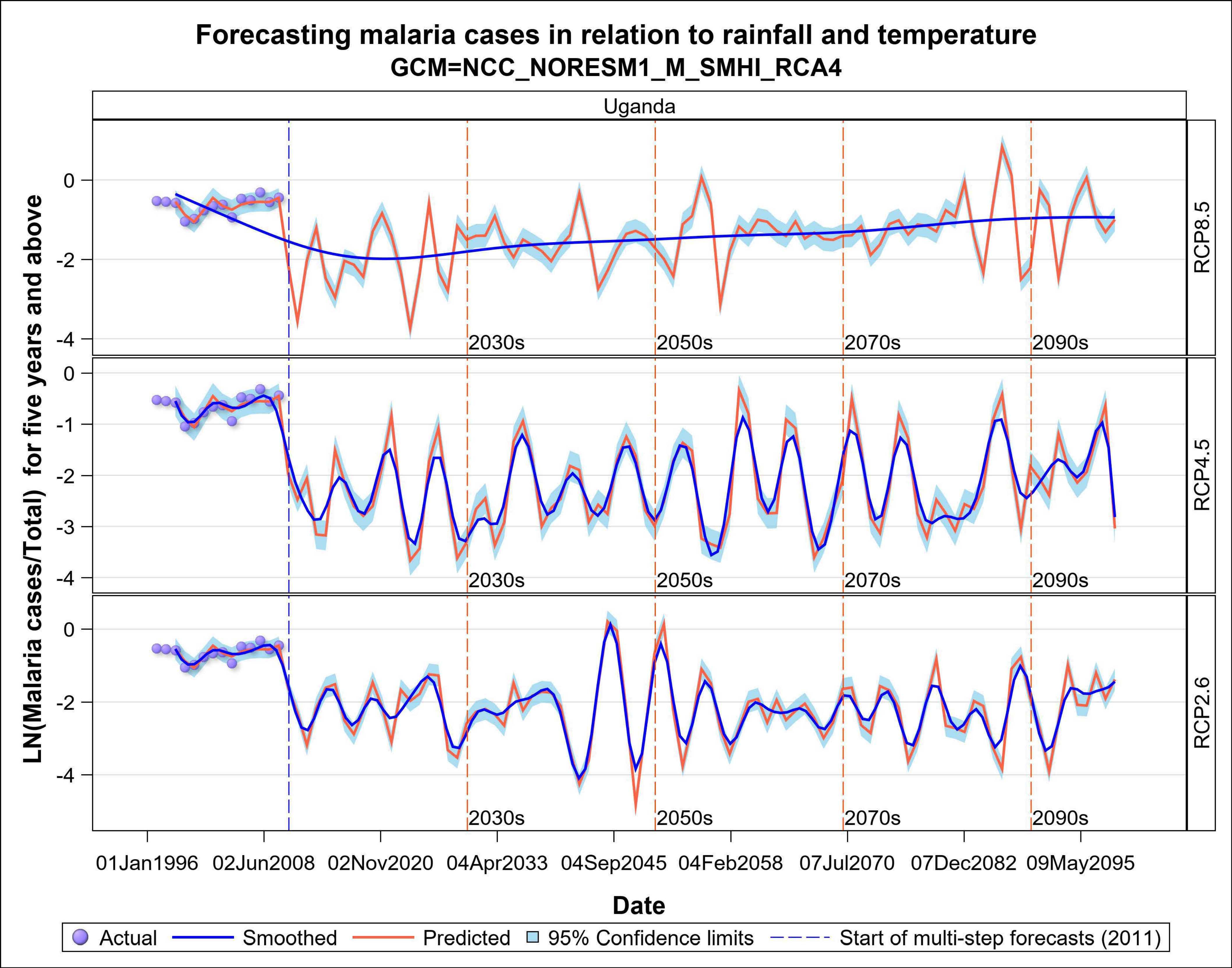

Forecasting malaria cases in relation to rainfall and temperature
GCM=NCC_NORESM1_M_SMHI_RCA4
Uganda
RCP8.5
RCP4.5
RCP2.6
2030s
2050s
2070s
2090s
LN(Malaria cases/Total) for five years and above
0
-1
-2
-3
-4
01Jan1996
02Jun2008
02Nov2020
04Apr2033
04Sep2045
04Feb2058
07Jul2070
07Dec2082
09May2095
Date
Actual
Smoothed
Predicted
95% Confidence limits
Start of multi-step forecasts (2011)
